# Supplementary material for: Isolation, (bio)synthetic studies and evaluation of antimicrobial properties of drimenol-type sesquiterpenes of Termitomyces fungi
Source: Commun Chem. 2023 Apr 24;6:79. doi: 10.1038/s42004-023-00871-z (PMC10126200; doi:10.1038/s42004-023-00871-z)
Supplement: Supplementary file 2 — Supplementary Information [file 42004_2023_871_MOESM2_ESM.pdf]

Supplementary Information to

Isolation, (bio)synthetic studies and evaluation of antimicrobial properties of drimenol-type sesquiterpenes of *Termitomyces* fungi

Nina B. Kreuzenbeck,[1] Seema Dhiman,[2] Dávid Roman,[1] Immo Burkhardt,[3] Benjamin H. Conlon,[4] Janis Fricke,[1] Huijuan Guo,[1] Janis Blume,[2] Helmar Görls,[5] Michael Poulsen,[4] Jeroen S. Dickschat,[3] Tobias G. Köllner,[6] Hans-Dieter Arndt,[2] and Christine Beemelmanns*[1,7,8]

[1] N. B. Kreuzenbeck, D. Roman, J. Fricke, H. Guo, C. Beemelmanns,
Chemical Biology of Microbe-Host Interactions
Leibniz Institute for Natural Product Research and Infection Biology – Hans Knöll-Institute (HKI),
Beutenbergstraße 11a, 07745, Jena, Germany,

[2] S. Dhiman, J. Blume, H.-D. Arndt
Institute for Organic and Macromolecular Chemistry
Friedrich-Schiller-University,
Humboldtstr. 10, 07743 Jena, Germany

[3] I. Burkhardt, J. S. Dickschat
Kekulé-Institute of Organic Chemistry and Biochemistry
University of Bonn, Gerhard-Domagk-Straße 1
53121 Bonn, Germany Department

[4] B. H. Conlon, M. Poulsen
Section for Ecology and Evolution, Department of Biology,
University of Copenhagen, Universitetsparken 15
2100 Copenhagen, Denmark

[5] H. Görls
Institute for Inorganic and Analytical Chemistry
Friedrich-Schiller University
07743 Jena, Germany

[6] T. G. Köllner
Max Planck Institute for Chemical Ecology
Department of Natural Product Biosynthesis
Hans-Knöll-Straße 8, 07745 Jena

[7] C. Beemelmanns
Helmholtz-Institut für Pharmazeutische Forschung Saarland (HIPS),
Helmholtz Zentrum für Infektionsforschung (HZI),
Campus E8, 66123 Saarbrücken, Germany,

[8] Universität des Saarlandes, Campus E8, 66123 Saarbrücken, Germany,
E-mail: Christine.Beemelmanns@helmholtz-hips.de

Table of Figures 4

Table of Tables 8

'Supplementary Methods' 10

1. General Experimental Procedures 10

2. Synthetic Procedures 12

Supplementary Note 1: Synthesis of drimenol derivatives **12**

1-((1*R*,2*R*,8a*S*)-2-Hydroxy-2,5,5,8a-tetramethyldecahydronaphthalen-1-yl)propan-2-one (28) 12

((1*S*,2*R*,8a*S*)-2-Hydroxy-2,5,5,8a-tetramethyldecahydronaphthalen-1-yl)methylacetate (8) 12

(1*S*,2*R*,8a*S*)-1-(Hydroxymethyl)-2,5,5,8a-tetramethyldecahydronaphthalen-2-ol (9) 13

((1*S*,8a*S*)-2,5,5,8a-Tetramethyl-1,4,4a,5,6,7,8,8a-octahydronaphthalen-1-yl)methanol (1) 15

(1*S*,8a*S*)-2,5,5,8a-Tetramethyl-1,4,4a,5,6,7,8,8a-octahydronaphthalene-1-carbaldehyde (11) 15

((1*S*,2*S*,8a*S*)-2,5,5,8a-Tetramethyldecahydronaphthalen-1-yl)methanol (12) 16

(*R*,1E,5*E*)-9,10-Dihydroxy-2,6,10-trimethylundeca-1,5-dien-1-yl acetate (14) 17

(2*E*,6*E*)-9-((*S*)-3,3-Dimethyloxiran-2-yl)-3,7-dimethylnona-2,6-dien-1-ol (29). 18

(2*E*,6*E*)-9-((*S*)-3,3-Dimethyloxiran-2-yl)-3,7-dimethylnona-2,6-dien-1-yl acetate (15) 18

((1*S*,4a*R*,6*S*,8a*S*)-6-Hydroxy-5,5,8a-trimethyl-2-methylenedecahydronaphthalen-1-yl)methyl acetate (16) 19

((1*S*,2*S*,6*S*,8a*S*)-6-Hydroxy-2,5,5,8a-tetramethyldecahydronaphthalen-1-yl)methyl acetate (17) 20

(2*S*,4a*S*,5*S*,6*S*,8a*R*)-5-(Hydroxymethyl)-1,1,4a,6-tetramethyldecahydronaphthalen-2-ol (18) 20

(4a*S*,5*S*,6*S*,8a*R*)-5-(Hydroxymethyl)-1,1,4a,6-tetramethyloctahydronaphthalen-2(1H)-one (19) 21

Supplementary Note 2: Synthesis of FPP and derivatives **22**

Farnesyl pyrophosphate (20) 22

10,11-Epoxyfarnesyl pyrophosphate ammonium salt (21) 23

(*2E,6E*)-1-Chloro-3,7,11-trimethyldodeca-2,6,10-triene (24) 23

9-(3,3-Dimethyloxiran-2-yl)-3,7-dimethylnona-2,6-dien-1-ol (25) 24

3-(9-Chloro-3,7-dimethylnona-3,7-dien-1-yl)-2,2-dimethyloxirane (26) 24

3,7,11-Trimethyldodeca-2,6,10-trien-1-yl acetate (27) 24

3. GC-MS-based Volatile Analysis of *Termitomyces* and Fungus Comb Samples 26

4. Cultivation and GC-MS-based Volatile Analysis of *Termitomyces* cultures 34

5. Isolation and Structure Elucidation of Drimenol Derivatives 41

6. Genome Mining of Putative Drimenol Synthases 55

7. Cloning and Heterologous Expression 67

8. Enzyme assay and compound purification 72

10. Agar Diffusion Test against *Termitomyces* sp. T153 79

11. Appendix HRMS and GC-MS Spectra 80

12. Supplementary References 84

Table of Figures

Figure S1. Synthesis of *trans,trans*-farnesyl pyrophosphate ammonium salt and its epoxy derivative as a mixture of isomers. 22
Figure S2. GC-MS chromatograms of volatiles emitted from a Termitomyces mushroom (without fungus comb, n = 2). 26
Figure S3. Sketch of the closed-loop stripping apparatus (CLSA)......................................27
Figure S4. GC-MS chromatograms of volatiles emitted from a Termitomyces mushroom emerging from fungus comb material (n=2). 28
Figure S5. GC-MS chromatograms of volatiles emitted from fungus comb material of Macrotermes natalensis colony MN187 (n =3)...30
Figure S6. GC-MS chromatograms of volatiles emitted from fungus comb material from Macrotermes natalensis colony MN188 (n = 3)..32
Figure S7. GC-MS chromatograms of volatile time study from Termitomyces strain T153 growing on PDA for 1-4 weeks. 35
Figure S8. GC-MS chromatograms of volatile time study from Termitomyces strain T153 growing **on PDA** for 5-8 weeks. Detected compounds are listed above...............................36
Figure S9. GC-MS chromatograms of volatile time study from Termitomyces strain T153 growing on cellulose medium after one (IBN 1117) and two weeks (IBN 1132). 38
Figure S10. GC-MS chromatograms of volatile time study from Termitomyces strain T153 growing on fungus comb medium after one (IBN 1118) and two (BN1131) weeks...............39
Figure S11. HR-MS spectra of drimenol derivatives **2-6** ([M+H] marked in green) detected from extracts of Termitomyces sp. T153 grown on ^{13}C-enriched medium. 41
Figure S12. Extracted ion chromatograms of drimenol derivatives **2-6** detected in extracts of *Termitomyces* sp. T153. 52
Figure S13. Extracted ion chromatograms of drimenol derivatives **2-6** of extracts obtained from Termitomyces sp. T153, T112 and P5-S after growth on PDA..52
Figure S14. Putative drimenol synthase (DS1) was identified by BLAST search of the AstC protein sequence (Accession Gene ID AORIB40_05908) against the respective Termitomyces predicted protein sequences of eight Termitomyces genomes. All identified sequences were aligned according to their classification by MUSCLE in Geneious Prime (2020.2.5) Putative active motifs are highlighted in orange boxes. 57
Figure S15. Identified DS2 sequences were aligned according to their classification by MUSCLE in Geneious Prime (2020.2.5). Putative active motifs are highlighted in orange boxes. 59
Figure S16. Alignment of DS3 protein sequences identified in six Termitomyces strains. Putative active motifs are highlighted in orange boxes. 60

Figure S17. Alignment of DS1-3 protein sequences of different lengths. Conserved motifs are highlighted in orange boxes. 61

Figure S18. Protein sequence alignment of putative drimenol synthases encoded in *Termitomyces* sp. T153. Active motifs (light blue) and hydrophobic tails (light brown) are colour coded. 62

Figure S19. Heatmap of expression levels (displayed as $\log_{10}$ values) of putative DS in *Termitomyces* sp. T153 in comparison to comb (of different age) and nodules of fungus growing termites (*M. natalensis* colony Mn156). DS1 was predicted as two separate transcripts but combined to one gene after reannotation. B) Table of transcript counts of RNAseq data from fresh/ old comb/ nodules (M*. natalensis* colony Mn156) and a plate culture of *Termitomyces* sp. T153. 66

Figure S20. SDS-PAGEs of heterologously expressed DS1-DS3 proteins of different length. O= originally predicted sequence, S1= shorter protein without C-terminal hydrophobic patch, S2= shorter protein without C-terminal hydrophobic patch and shorter N-terminus starting at an alternative start codon, 2xHis version of DS3-O with a His-tag at the N- and C-terminus. C= control sample, empty pET28a(+) vector expressed in E. coli BL21 (DE3). 71

Figure S21. SDS-Pages and Western Blots of heterologously produced DS1 and DS2 proteins tagged with MBP at the N-Terminus. S, soluble fraction; P, pellet fraction. 72

Figure S22. SDS-PAGE of selected FPLC fractions from purification of big scale DS3 protein expression. 72

Figure S23. GC-chromatogram comparison of enzyme assays with native DS1-DS3 E. coli soluble protein extracts incubated with FPP as substrate. As negative control served a soluble protein extract of an induced E. coli empty pET28 vector culture. Cyclization products of reaction with DS3 are marked with 1 = compound **22**, 2 = compound **23**, and * represents farnesol. 73

Figure S24. GC-chromatogram comparison of enzyme assays with Ni-NTA chromatographically purified DS1-MBP, DS2-MBP, DS3 or a mixture of proteins proteins incubated with FPP as substrate. As negative control a soluble protein extract of an induced E. coli empty pET28 vector culture was purified by Ni-NTA affinity chromatography and used in the assay instead of heterologous protein. Cyclization products of enzymatic reaction are marked with 1 = compound **22**, 2 = compound **23**, and * represents farnesol. 73

Figure S25. GC-chromatogram comparison of enzyme assays with purified DS3 protein incubated with FPP as substrate in various buffers adjusted to different pH values. Cyclization products of reaction are marked with 1 = compound **22**, 2 = compound **23**, and * represents farnesol. 74

Figure S26. Comparison of GC-chromatogram sections obtained from enzyme assays with purified DS3 protein incubated with FPP as substrate and different divalent metal cofactors.

As negative control a soluble protein extract of an induced E-coli empty pET28 vector culture was purified by Ni-NTA affinity chromatography and used in the assay instead of heterologous protein. Cyclization products of reaction with DS3 are marked with 1 = compound **22**, 2 = compound **23**, and * represents farnesol. Yet unidentified enzyme products are highlighted in grey and marked with "?". 74

Figure S27. Comparison of GC-chromatogram sections from enzyme assays with purified DS3 protein incubated with different substrates. Cyclization products of reaction with DS3 are marked with 1 = compound **22** and 2 = compound **23**. Dephosphorylated products are marked with asterisks (* farnesol, ** geraniol, *** geranylgeraniol, **** farnesolepoxide). 75

Figure S28. a Two steps HPLC separation chromatograms to remove dephosphorylated farnesol **13** from mixture of target compounds and the final separation of enzymatically formed compounds **22** and **23**. **b** ^{1}H-^{1}H COSY and key HMBC correlations in compounds **22** and **23**. The ^{1}H NMR spectroscopy showed proton resonances, which include a doublet methyl at δ_H 0.86 (3H, d, *J* = 6.7 Hz, H-13), singlet methyl at δ_H 0.87 attached to a quarternary carbon (3H, s, H-14), two methyls at δ_H 1.61 (3H, m, H-12) and δ_H 1.68 (3H, s, H-15) attached to olefinic system, two olefinic methines at δ_H 5.41 (m, H-2) and δ_H 5.42 (m, H-10). Furthermore, the undeniable chemical shift at δ_H 4.15 (2H, d, *J* = 6.8 Hz, H-1) suggests the presence of allylic alcohol. These proton resonances correlate with existence of substituted cyclohexene ring. Our theory of two double bonds was supported by ^{13}C NMR signals at δ_C 122.4 (C-10), 122.8 (C-2), 139.7 (C-11) and 141.1 (C-3). The presence of alcohol was endorsed by appeared signal at δ_C 59.6 (C-1). The existence of main cyclohexene core was supported by the finding of reciprocal ^{1}H-^{1}H COSY correlations between H-7, H-8, H-9 and H-10. Comprehensive HMBC analysis revealed the correlation from H-1 to C-2 and C-15 that indicates the attachment of C-15 methyl group to allylic alcohol. The position of a second double bond was also assigned by HMBC correlation of H-12 to olefinic C-10 and C-11. In addition, H-12 correlation to sp^3 quaternary carbon C-6 indicates the presence of aliphatic chain in *α* position. Additional HMBC correlations from H-7 to C-8 and C-9, from H-8 to C-9, C-7 and C-6, from H-9 to C-8, C-7 and C-10 confirmed that the main core of alcohol **22** is formed by 6,7,11-trimethylcyclohexene substituted at C-6 position. Comparative NMR analysis identified a very similar chemical shift pattern for the structurally related compound **23**, which differ only in position of C-14 methyl group and a new characteristic CH signal at δ_H 1.42...76

Figure S29. Pictures of disc diffusion assay of isolated and synthesized drimenol derivatives against Termitomyces sp. T153. Substances were tested in triplicates. Termitomyces sp. T153 was inoculated on small PDA plates (200 µl mycelium suspension) and incubated for one day at room temperature. The next day, paper discs soaked with 10 µL stock solutions (1 mg/mL in MeOH) were placed in the middle of inoculated Termitomyces plates and growth was monitored every day. Pictures were taken after 12 days..79

Figure S30. ESI-HRMS (+) spectrum of compound **2**. 80
Figure S31. GC-MS spectrum of compound **2**. 80
Figure S32. ESI-HRMS (+) spectrum of compound **3**. 81
Figure S33. GC-MS spectrum of compound **3**. 81
Figure S34. ESI-HRMS (+) spectrum of compound **4**. 82
Figure S35. GC-MS spectrum of compound **4**. 82
Figure S36. ESI-HRMS (+) spectrum of compound **5**. 83
Figure S37. GC-MS spectrum of compound **5**. 83
Figure S38. ESI-HRMS (+) spectrum of compound **6**. 84
Figure S39. GC-MS spectrum of compound **6**. 84

Table of Tables

Table S1. GC-MS analysis of volatiles emitted by a Termitomyces mushroom (without fungus comb, n = 2). Numbers of compounds are in accordance with those from the GC-MS chromatogram. Intensity scores indicated as **x**. 27
Table S2. GC-MS analysis of volatiles emitted by a Termitomyces mushroom emerging from fungus comb (n=2). Numbers of compounds are in accordance with those from the GC-MS chromatogram. Intensity scores indicated as **x**. 29
Table S3. GC-MS analysis of volatiles emitted by fungus comb material *Macrotermes natalensis* colony MN187 (n =3). Numbers of compounds are in accordance with those from the GC-MS chromatogram. Intensity scores indicated as x....31
Table S4. GC-MS analysis of volatiles emitted by fungus comb material from *Macrotermes natalensis* colony MN188 (n = 3). Numbers of compounds are in accordance with those from the GC-MS chromatogram Figure S5, Figure S6). *Intensity scores indicated as **x**.* 33
Table S5. Composition of cultivation media (for solid agar: 20.0 g/L agar-agar was added) .34
Table S6. GC-MS analysis of volatiles emitted by *Termitomyces* sp. T153 growing on PDA plates over 8 weeks. Numbers of compounds are in accordance with those from the GC-MS chromatogram. 37
Table S7. GC-MS analysis of volatiles from *Termitomyces* on different media after one and two weeks of growth. Numbers of compounds are in accordance with those from the GC-MS chromatogram ***Figure S9***, ***Figure S10***. 40
Table S8. NMR spectral data ($CDCl_3$, at 300 K) for compound **2**....42
Table S9. Comparison of NMR spectral data of compound **2** with literature data reported for compound **3** and **31a**.[12] 43
Table S10. NMR spectral data ($CDCl_3$, at 300 K) for compound **3**....44
Table S11. Comparison of NMR spectral data of compound **3** with literature data. 45
Table S12. NMR spectral data ($CDCl_3$, at 300 K) for compound **4**....46
Table S13. Comparison of NMR spectral data of compound **4** with literature data. 47
Table S14. NMR spectral data ($CDCl_3$, at 300 K) for compound **5**....48
Table S15. Comparison of NMR spectral data of compound **5** with literature data. 49
Table S16. NMR spectral data ($CDCl_3$, at 300 K) for compound **6**....50
Table S17. Comparison of NMR spectral data of compound **6** with literature data. 51
Table S18. Comparison of ^{1}H-NMR chemical shift values of isolated and synthesized compounds **1**, **2**, **3**, **12**, **18** and **19** and reported literature values for **1** ([a] 400 MHz in $CDCl_3$, [b] 300 MHz in CD_3OD). 53

Table S19. Comparison of ^{13}C chemical shift values of isolated and synthesized compounds **1**, **2**, **3**, **12**, **18** and **19** and reported literature values for **1** ([a] 400 MHz in $CDCl_3$, [b] 300 MHz in CD_3OD). 54
Table S20. List of Termitomyces strains used for genome mining. 55
Table S21. Identified terpenes from Termitomyces sp. mushrooms and the identified or putatively assigned enzymes responsible for biosynthesis of the respective compounds along with their proposed cyclization intermediates. Terpenes with unusual biosynthesis are marked in orange. 56
Table S22. Table of genes identified 30.000 bp up- and downstream of DS1 gene in Termitomyces sp. T153 with predicted domains of translated protein sequence....................63
Table S23. Table of genes identified 30.000 bp up- and downstream of DS2 gene in Termitomyces sp. T153 with predicted domains of translated protein sequences. 64
Table S24. Table of genes identified 30.000 bp up- and downstream of DS3 gene in Termitomyces sp. T153 with predicted domains of translated protein sequences. 65
Table S25. List of identified native gene, transcript, and protein sequences of DS1-3 in Termitomyces strain T153. 67
Table 26. Codon optimized transcript sequences. Codon optimization did not change the amino acid composition of the translated proteins. 70
Table S27. List of primer sequences for heterologous expression of DS1-3 proteins with varying length. Restriction sites are underlined....................71
Table S28. NMR spectral data ($CDCl_3$, at 300 K) for compound **22**....................77
Table S29. Comparison of NMR spectral data of compound **22** with literature data. 77
Table S30. NMR spectral data ($CDCl_3$, at 300 K) for compound **23**....................78
Table S31. Comparison of NMR spectral data of compound **23** with literature data. 78

'Supplementary Methods'

1. General Experimental Procedures

NMR measurements were performed on a Bruker AVANCE III 500 MHz and 600 MHz spectrometer, equipped with a Bruker Cryoplatform. The chemical shifts are reported in parts per million (ppm) relative to the solvent residual peak of $CDCl_3$ (^{1}H: 7.26 ppm, singlet; ^{13}C: 77.16 ppm, triplet). Synthesis of drimenol derivatives: ^{1}H- and ^{13}C-NMR-spectra were recorded at 297 K on one of the following Bruker spectrometers: AC 250, AC 300, or AC 400. Chemical shifts (δ) are expressed in parts per million (ppm) with respect to the solvent signal (^{13}C-NMR, δ: C_6D_6 = 128.06, $CDCl_3$ = 77.16, CD_3OD = 49.00; ^{1}H-NMR, δ: C_6D_6 = 7.16, $CDCl_3$ = 7.26, CD_3OD = 3.31) and were assigned based on 2D NMR-experiments.

GC-MS measurements for enzyme assay: A Hewlett-Packard model 6890 gas chromatograph was employed with the carrier gas He at 2 mL min 1, splitless injection (injector temperature, 230 °C; injection volume, 1 µL), a ZB5 column (Phenomenex, Aschaffenburg, Germany, 30 m x 0.25 mm x 0.25 µm) and a temperature program from 45 °C (2-min hold) at 6 °C min-1 to 200 °C, and 60 °C min-1 to 320 °C (1 min hold). The coupled mass spectrometer was a Hewlett-Packard model 5973 with a quadrupole mass selective detector (transfer line temperature, 270 °C; source temperature, 230 °C; quadrupole temperature, 150 °C; ionization potential, 70 eV; scan range, 44-300 atomic mass units). Compound identification was performed by comparison with NIST 2017 database.

LC-ESI-HRMS based metabolomics were performed on a Dionex Ultimate3000 system coupled with a Luna Omega C18 column (100 x 2.1 mm, particle size 1.6 µm, pore diameter 100 Å, Phenomenex) combined with Q-Exactive Pulse mass spectrometer (Thermo Scientific) equipped with an electrospray ion (HESI) source. Column oven was set to 40 °C; scan range of full MS was set to m/z 150 to 2,000 with resolution of 70,000 and AGC target 3e6 and maximum IT 100 ms under positive and negative mode with centroid data type. The spray voltage (+) was set to 4000 Volt, and (–) was set to 3300 Volt. The capillary temperature (+–) was set to 340 °C and probe heater temperature (+–) was set to 200 °C. The sheath gas flow (+–) was set to 35 L/min and Aux gas flow (+–) to 5 L/min. Max spray current (+) and (–) was set to 100 Volt. S-Lens RF level was set to 50. Synthesis of drimenol derivatives: High-resolution mass spectra (HRMS) were recorded on one of the following machines: Bruker Maxis Impact (QTOF) in ESI mode, Thermo Scientific Q Exactive Plus (Orbitrap) in ESI or APCI mode or Thermo Q Exactive GC (Orbitrap) in EI mode.

UHPLC-MS measurements were performed on a Shimadzu LCMS-2020 system equipped with single quadrupole mass spectrometer using a Phenomenex Kinetex C18 column (50 x 2.1 mm, particle size 1.7 µm, pore diameter 100 Å). Column oven was set to 40 °C; scan range of MS

was set to m/z 150 to 2,000 with a scan speed of 10,000 u/s and event time of 0.25 s under positive and negative mode. DL temperature was set to 250 °C with an interface temperature of 350 °C and a heat block of 400 °C. The nebulizing gas flow was set to 1.5 L/min and dry gas flow to 15 L/min.

Preparative HPLC was performed on a Shimadzu HPLC system using Luna C18(2) column 250 x 30 mm (particle size 5 µm, pore diameter 100 Å, Phenomenex) and **Semi-preparative HPLC** was performed on a Shimadzu HPLC system using a Phenomenex Luna C18(2) 250 x 10 mm column (particle size 5 µm, pore diameter 100 Å) and Phenomenex Luna5u Phenyl-Hexyl column 250x10 mm.

Flash chromatography was performed on Biotage Isolera Prime system. Normal phase purifications were run on Biotage SNAP KP-Sil or Chromabond cartridges packed with Normasil 60 silica gel (partical size 40 – 63 µm) using cyclohexane and EtOAc as solvents, unless otherwise stated.

Ion exchange chromatography was performed using DOWEX 50WX8 resin (Carl Roth; CAS: 11119-67-8, 100-200 mesh, H^+ form).

IR spectra were recorded on an FT/IR-4100 ATR spectrometer (JASCO). Synthesis of drimenol derivatives: FT-IR spectra were recorded on a Shimadzu IRAffinity-1 machine in ATR mode. The following notations indicate the intensity of the absorption bands: *s*=strong, *m*=medium, *w*=weak.

Optical rotations were recorded in $CHCl_3$ on a P-1020 polarimeter (JASCO).

Melting points were determined with a Büchi B-540 apparatus, and optical rotations were recorded with a Jasco P-2000 polarimeter at 589 nm. The path length of the cuvette was d = 100 mm. Specific rotations (α) are expressed in deg · mL · g^{-1} · dm^{-1}, but reported without the unit. Corresponding concentrations (c) are given in g/(100mL).

Analytical thin-layer chromatography (TLC) was performed on silica gel on aluminum sheets (60 F254) from Merck. Substances were detected by UV quenching (254 nm) or staining (PMA in EtOH solution).

Chemicals: Methanol, acetonitrile, dichlormethane (Th. Geyer); water for analytical and preparative HPLC (Millipore, Germany); formic acid (Carl Roth, Germany); acetonitrile (VWR as LC-MS grade); media ingredients (Carl Roth, Germany). Synthesis of FPP and derivatives: All reagents and solvents for synthesis were purchased from Acros Organics, Alfa Aesar, Carbolution Chemicals, Carl Roth, Fluorochem, Sigma Aldrich, TCI, Th. Geyer, VWR in the highest commercial quality and used without further purification, unless otherwise stated. Synthesis of drimenol derivatives: Reagents available from commercial sources were used without further purification.

2. Synthetic Procedures

Supplementary Note 1: Synthesis of drimenol derivatives

1-((1*R*,2*R*,8a*S*)-2-Hydroxy-2,5,5,8a-tetramethyldecahydronaphthalen-1-yl)propan-2-one (28)

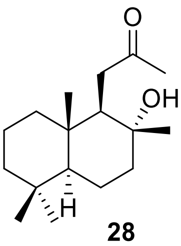

28

To a stirred solution of sclareolide **7** (505 mg, 1.96 mmol) in anhydrous Et_2O (20 mL) at -78 °C was added MeLi (1.96 mL, 1.6 M in Et_2O 3.13 mmol, 1.6 eq.) dropwise over a time period of 10 min under N_2 atmosphere. The reaction mixture was stirred for 30 min at this temperature before warming to 25 ˚C. The reaction mixture was slowly quenched by addition of sat. aq. NH_4Cl solution (10 mL). The layers were separated, and the aqueous phase was extracted with Et_2O (3 x 20 mL). The combined organic layers were dried over Na_2SO_4, filtered and the volatiles were evaporated under reduced pressure. The crude mixture was subjected to column chromatography (190 mL SiO_2, 10-30% EtOAc in PE). The ketone **28** (439 mg, 1.65 mmol, 84%) was afforded as a colourless oil.

^{1}H-NMR (300 MHz, $CDCl_3$, 297 K): δ = 2.59 – 2.40 (m, 2H), 2.21 (s, 3H), 1.98 – 1.89 (m, 2H), 1.74 – 1.65 (m, 1H), 1.62 – 1.50 (m, 1H), 1.48 – 1.32 (m, 5H), 1.31 – 1.23 (m, 1H), 1.22 – 1.13 (m, 1H), 1.11 (s, 3H), 1.02 (dd, *J* = 12.0, 2.2 Hz, 1H), 0.97 – 0.89 (m, 1H), 0.88 (s, 3H), 0.79 (s, 6H) ppm.

^{13}C-NMR (75 MHz, $CDCl_3$, 297 K): δ = 210.2, 73.1, 55.9, 55.8, 44.6, 41.7, 39.5, 39.2, 38.29, 33.3, 33.2, 30.3, 23.0, 21.3, 20.5, 18.3, 15.6 ppm.

IR (ATR): $\tilde{\nu}$ = 3410 (*w*, *br*), 2960 *(m)*, 1704 (*s*), 1462 (*m*), 1384 (*m*), 1045 (*s*), 1001 (*s*), 940 (*m*), 827 (*m*) cm^{-1}.

The ^{1}H- and ^{13}C-NMR data are matching with the reported literature data.[1]

((1*S*,2*R*,8a*S*)-2-Hydroxy-2,5,5,8a-tetramethyldecahydronaphthalen-1-yl)methylacetate (8)

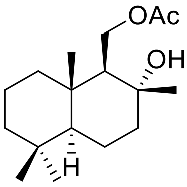

8

To a stirred solution of TFAA (3.46 mL, 24.9 mmol, 15.5 eq.) in anhydrous CH_2Cl_2 (14 mL), hydrogen peroxide (1.19 mL, 30% in H_2O, 11.7 mmol, 7.3 eq.) was added at 0 °C. After 10 min stirring, the reaction mixture was treated with $NaHCO_3$ (1.25 g) and stirred for an additional 10 min. Methyl ketone **28** (428 mg, 1.61 mmol) was dissolved in anhydrous CH_2Cl_2 (15 mL) and dropwise added to the reaction mixture at 25 °C. The reaction mixture was stirred for 45 min at 25 °C until the full conversion of methyl ketone was observed by TLC (TLC control EtOAc/PE, 30:70). The reaction mixture was quenched by adding water (10 mL), and pH was basified by the

addition of $NaHCO_3$ over a period of 30 min. The layers were separated, and the aqueous phase was extracted with CH_2Cl_2 (3 x 10 mL), dehydrated with Na_2SO_4, filtered, and the volatile compounds were evaporated under reduced pressure. The crude product was purified by silica gel column chromatography (150 mL SiO_2, 15-30% EtOAc in PE) to yield ester **8** (299 mg, 1.06 mmol, 66%) as a colourless oil.

$[\alpha]_D^{23}$ = -7.0 (*c* = 0.11, $CHCl_3$).

^{1}H-NMR (400 MHz, $CDCl_3$, 297 K): δ = 4.44 – 4.23 (m, 2H), 2.29 (s, 1H, broad), 2.07 (s, 3H), 1.91 (dt, *J* = 12.6, 3.2 Hz, 1H), 1.73 – 1.66 (m, 2H), 1.65 – 1.56 (m, 1H), 1.54 (t, *J* = 4.8 Hz, 1H), 1.52 – 1.39 (m, 3H), 1.28 (dd, *J* = 12.3, 3.2 Hz, 1H), 1.20 (s, 3H), 1.19 – 1.14 (m, 1H), 1.07 (td, *J* = 13.0, 3.3 Hz, 1H), 0.98 (dd, *J* = 12.1, 2.1 Hz, 1H), 0.90 (s, 3H), 0.88 (s, 3H), 0.83 (s, 3H) ppm.

^{13}C-NMR (101 MHz, $CDCl_3$, 297 K) δ = 171.3, 72.5, 62.5, 59.9, 55.7, 43.9, 41.7, 39.7, 38.0, 33.4, 33.1, 24.5, 21.5, 21.3, 20.2, 18.3, 15.8 ppm.

IR (ATR): $\tilde{\nu}$ = 3560 (*w*, *br*), 3483 (*w*, *br*), 2963 *(m)*, 2871 (*m*), 1724 (*s*), 1462 (*m*), 1384 (*m*), 1030 (*s*), 941 (*m*), 825 (*m*) cm^{-1}.

The optical rotation[2] and ^{1}H, ^{13}C-NMR data are matching with the reported literature data.

(1*S*,2*R*,8*aS*)-1-(Hydroxymethyl)-2,5,5,8a-tetramethyldecahydronaphthalen-2-ol (9)

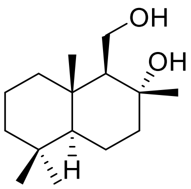

9

To a stirred solution of **8** (180 mg, 0.63 mmol) in MeOH (5 mL) at 25 °C was added 10% methanolic KOH solution (5 mL). After 10 min stirring, the reaction mixture was concentrated under reduced pressure. The residue was partitioned between Et_2O (30 mL), and water (5 mL). The organic layer was dehydrated over Na_2SO_4, filtered and the volatile compounds were evaporated under reduced pressure. The diol **9** (149 mg, 0.62 mmol, 98%) was afforded as colorless solid.

$[\alpha]_D^{23}$ = +1.59 (*c* = 0.63, $CHCl_3$).

^{1}H-NMR (400 MHz, $CDCl_3$, 297 K) *δ* = 3.94 (d, *J* = 7.3 Hz, 2H), 3.23 (s, 1H, OH), 2.73 (s, 1H, OH), 1.91 (dt, *J* = 12.4, 3.2 Hz, 1H), 1.77 (dd, *J* = 13.2, 2.6 Hz, 1H), 1.73 – 1.61 (m, 2H), 1.61 – 1.52 (m, 2H), 1.52 – 1.38 (m, 2H), 1.37 (s, 3H), 1.30 (ddd, *J* = 13.5, 12.2, 3.2 Hz, 1H), 1.23 – 1.18 (m, 1H), 1.18 – 1.10 (m, 1H), 0.99 (dd, *J* = 12.2, 2.1 Hz, 1H), 0.90 (s, 3H), 0.81 (s, 6H) ppm.

^{13}C-NMR (101 MHz, $CDCl_3$, 297 K) *δ* = 75.1, 61.1, 60.5, 55.9, 44.5, 41.7, 40.0, 37.5, 33.5, 33.2, 24.3, 21.6, 20.2, 18.6, 16.0 ppm.

IR (ATR): $\tilde{\nu}$ = 3360 (*w*, *br*), 3481 (*w*, *br*), 2941 *(m)*, 2845 (*m*), 1452 (*m*), 1384 (*m*), 943 (*m*), 813 (*m*) cm^{-1}.

Melting point: T_m = 118 °C.

The optical rotation and ^1H, ^{13}C-NMR data are matching with the reference literature data.

(4a*S*)-1,1,4a,6-Tetramethyl-5-methylene-1,2,3,4,4a,5,8,8a-octahydronaphthalene (10)

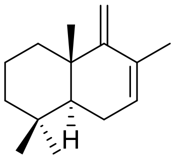

10

To a stirred solution of PPh_3 (54.1 mg, 0.20 mmol, 1.2 eq.) in anhydrous CH_2Cl_2 (1 mL) at 25 °C was added iodine (52.3 mg, 0.20 mmol, 1.2 eq.). After stirring for 10 min a solution of diol **9** (41.3 mg, 0.17 mmol) in anhydrous CH_2Cl_2 (1 mL) was added to the mixture. After 4 h at this temperature was added 5% aq. $NaHSO_3$ solution (5 mL) and stirred for 10 min. Reaction mixture was diluted with CH_2Cl_2 (10 mL), and the phases were separated. The aqueous phase was extracted with CH_2Cl_2 (15 x 2 mL), and combined organic phases were washed with water (20 mL) and brine (20 mL). The organic phase was dehydrated with Na_2SO_4, filtered, and the volatiles were evaporated under reduced pressure. The crude product was purified by column chromatography (15 mL SiO_2 with $AgNO_3$, 0 - 2% EtOAc in PE) to yield alkene **10** (21.3 mg, 104 µmol, 61%) as a colorless liquid.

$[\alpha]_D^{23}$ = -135.6 (*c* = 0.14, $CHCl_3$).

^{1}H-NMR (300 MHz, C_6D_6, 297 K) δ = 5.67 (s, 1H), 4.85 (s, 1H), 4.81 (s, 1H), 2.23 – 2.01 (m, 2H), 1.88 (dq, *J* = 12.6, 3.0 Hz, 1H), 1.81 (s, 3H), 1.65 (dt, *J* = 13.4, 3.1 Hz, 1H), 1.61 – 1.50 (m, 1H), 1.49 – 1.38 (m, 2H), 1.30 (dd, *J* = 11.4, 5.0 Hz, 1H), 1.20 (dd, *J* = 13.0, 4.1 Hz, 1H), 0.98 (s, 3H), 0.94 (s, 3H), 0.88 (s, 3H) ppm.

^{13}C-NMR (75 MHz, C_6D_6, 296 K) δ = 157.8, 131.1, 126.3, 103.8, 48.4, 42.0, 37.7, 37.6, 33.0, 32.7, 24.2, 21.9, 21.0, 20.4, 19.0 ppm.

IR (ATR): $\tilde{\nu}$ = 2924 (*m*), 2845 (*w*), 1605 (*w*), 1458 (*w*), 1369 (*w*), 883 (*s*), 818 (*w*) cm^{-1}.

HRMS [EI]: m/z calculated $C_{15}H_{24}O$ $[M]^+$ 204.1878, observed 204.1873.

The ^1H- and ^{13}C-NMR data are matching with the reported literature data.[3]

((1*S*,8a*S*)-2,5,5,8a-Tetramethyl-1,4,4a,5,6,7,8,8a-octahydronaphthalen-1-yl)methanol (1)

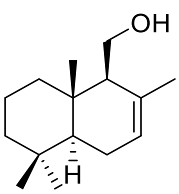

1

To a stirred solution of diol **9** (110.5 mg, 0.46 mmol) in anhydrous CH_2Cl_2 (5 mL) at 25 °C was added *p*-TsOH*H_2O (87.4 mg, 0.46 mmol, 1.0 eq.). After stirring the reaction mixture for 6 h at the same temperature, sat. aq. $NaHCO_3$ solution (2 mL) was added, and the two phases were separated. The aqueous phase was extracted with CH_2Cl_2 (3 x 15 mL), and the combined organic phase was dehydrated with Na_2SO_4, filtered, and the volatile compounds were evaporated under reduced pressure. The crude product was purified by column chromatography (50 mL SiO_2, 5 - 20% EtOAc in PE) to give alcohol **1** (61.9 mg, 0.31 mmol, 61%) as colorless solid.

$[\alpha]_D^{23}$ = -20.3 (*c* = 0.11, $CHCl_3$).

^{1}H-NMR (400 MHz, $CDCl_3$, 297 K) δ = 5.69 – 5.47 (m, 1H), 3.95 – 3.67 (m, 2H), 2.08 – 1.95 (m, 2H), 1.95 – 1.84 (m, 2H), 1.81 (s, 3H), 1.66 – 1.40 (m, 3H), 1.27 – 1.08 (m, 4H), 0.91 (s, 3H), 0.89 (s, 3H), 0.88 (s, 3H) ppm.

^{13}C-NMR (101 MHz, $CDCl_3$, 297 K) δ = 132.8, 124.1, 60.9, 57.3, 49.9, 42.1, 39.9, 36.0, 33.3, 32.9, 23.5, 22.0, 21.9, 18.7, 14.9 ppm.

IR (ATR): $\tilde{\nu}$ = 3314 (*w*, *br*), 2920 (*m*), 2847 (*w*), 1740 (*w*), 1452 (*m*), 1389 (*m*), 1030 (*s*), 1003 (*s*), 964 (*m*), 818 (*m*) cm^{-1}.

Melting point: T_m = 86 °C.

HRMS [EI]: m/z calculated $C_{15}H_{26}O$ $[M]^+$ 222.1984, observed 222.1972.

The optical rotation, ^{1}H- and ^{13}C-NMR data are matching with the reported literature data.[4]

(1*S*,8a*S*)-2,5,5,8a-Tetramethyl-1,4,4a,5,6,7,8,8a-octahydronaphthalene-1-carbaldehyde (11)

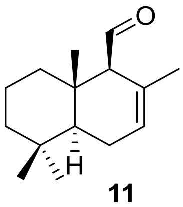

11

A stirred solution of oxalyl chloride (120 µL, 1.40 mmol, 5.2 eq.) in anhydrous CH_2Cl_2 (3 mL) was cooled to -78 °C. A solution of anhydrous DMSO (186 µL, 2.61 mmol, 9.7 eq.) in anhydrous CH_2Cl_2 (1 mL) was added to the solution under the N_2 atm. and the mixture was stirred for 10 min. A solution of alcohol **1** (60.1 mg, 0.27 mmol) in anhydrous CH_2Cl_2 (1 mL) was added dropwise to the reaction mixture and was stirred for 45 min at the same temperature. After 45 minutes, NEt_3 (899 µL, 6.49 mmol, 24.0 eq.) was added dropwise at -78 °C and the reaction mixture was allowed to warm to 25 °C in 2 h. Water (5 mL) was added to the reaction mixture, phases were separated, and the aqueous phase was extracted with CH_2Cl_2 (4 x 15 mL). The combined organic phases were washed with saturated aq. $NaHCO_3$ solution (5 mL) and water (20 mL).

Organic layer was dehydrated over Na_2SO_4, filtered, and the volatile compounds were evaporated under reduced pressure. The crude product was purified by column chromatography (20 mL SiO_2, 0 - 5% EtOAc in PE), to give aldehyde **11** (45.6 mg, 207 µmol, 77%) as a colorless oil with matching analytical data.[5]

$[\alpha]_D^{23}$ = +15.6 (*c* = 0.15, $CHCl_3$).

^{1}H-NMR (300 MHz, $CDCl_3$, 297 K) δ = 9.70 (d, *J* = 5.2 Hz, 1H), 5.85 – 5.58 (m, 1H), 2.60 (s, 1H), 2.16 – 1.88 (m, 2H), 1.63 (dd, *J* = 2.5, 1.3 Hz, 3H), 1.74 – 1.39 (m, 4H), 1.36 – 1.12 (m, 3H), 1.08 (s, 3H), 0.93 (s, 3H), 0.88 (s, 3H) ppm.

^{13}C-NMR (101 MHz, $CDCl_3$, 297 K) δ = 206.7, 127.7, 125.4, 67.6, 49.0, 42.0, 40.3, 37.0, 33.3, 33.0, 23.6, 22.0, 21.6, 18.2, 15.7 ppm.

IR (ATR): $\tilde{\nu}$ = 2924 (*m*), 2851 (*w*), 1720 (s), 1443 (*w*), 1385 (*w*), 1115 (*w*) cm^{-1}.

HRMS [EI]: m/z calculated $C_{15}H_{24}O$ $[M]^+$ 220.1827, observed 220.1822.

((1*S*,2*S*,8a*S*)-2,5,5,8a-Tetramethyldecahydronaphthalen-1-yl)methanol (12)

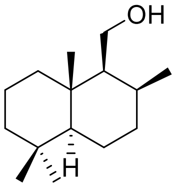

12

To a stirred solution of alcohol **1** (30.0 mg, 0.13 mmol) in anhydrous EtOAc (6 mL) at 25 °C was added PtO_2*H_2O (0.04 mmol). The reaction mixture was flushed with H_2 gas for 20 min and then reaction mixture was stirred for 2 h under the H_2-atmosphere (1 atm). After complete conversion, reaction mixture was filtered over celite and washed with EtOAc (20 mL). The solvent was evaporated under reduced pressure, and the resulted crude product was purified by column chromatography (25 mL SiO_2, 5 - 15% EtOAc in PE). The alcohol **12** (28.7 mg, 128 µmol, 95%) was afforded as white solid.

$[\alpha]_D^{23}$ = +16.5 (*c* = 0.13, $CHCl_3$).

^{1}H-NMR (400 MHz, $CDCl_3$, 297 K) δ = 3.88 (dt, *J* = 8.3, 3.8 Hz, 1H), 3.62 (td, *J* = 10.2, 3.5 Hz, 1H), 2.17 (dtd, *J* = 9.8, 4.9, 2.3 Hz, 1H), 1.75 – 1.66 (m, 2H), 1.65 – 1.47 (m, 3H), 1.47 – 1.36 (m, 4H), 1.18 (td, *J* = 13.4, 13.0, 4.3 Hz, 1H), 1.10 – 1.02 (m, 2H), 0.99 (d, *J* = 7.6 Hz, 3H), 0.89 (s, 6H), 0.84 (s, 3H) ppm.

^{13}C-NMR (101 MHz, $CDCl_3$, 297 K) δ = 61.0, 56.5, 55.7, 41.9, 39.9, 37.5, 34.4, 33.5, 33.2, 28.5, 21.6, 18.4, 17.5, 17.0, 15.6 ppm.

IR (ATR): $\tilde{\nu}$ = 3333 (*w*, *br*), 2904 (s), 2851 (*m*), 1740 (*w*), 1439 (*m*), 1385 (*m*), 1026 (*s*) cm^{-1}.

Melting point: T_m = 102 °C.

HRMS [EI]: m/z calculated $C_{15}H_{28}O$ $[M]^+$ 224.2140, observed 224.2134.

The optical rotation, ^{1}H- and ^{13}C-NMR data are matching with the reported literature data.[6]

(*R*,1E,5*E*)-9,10-Dihydroxy-2,6,10-trimethylundeca-1,5-dien-1-yl acetate (14)

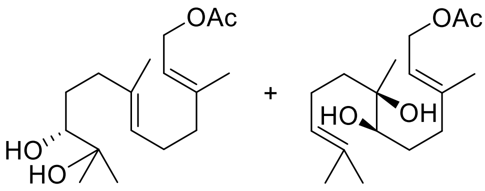

14
30%, 97% *ee*

15%, **14a**

To a stirred suspension of AD-mix-β (3.75 g) in 20 mL *t*-BuOH and water (1:1) under N_2 atm. at 0 °C was added $MeSO_2NH_2$ (296 mg, 3.12 mmol, 1.0 eq.). After stirring 10 min, a solution of farnesyl acetate (830.1 mg, 3.12 mmol) in *t*-BuOH, and water (1:1) (7 mL) was added dropwise to the suspension at 0 °C. The mixture was stirred at the same temperature for 24 h. Reaction was quenched by the addition of aqueous Na_2SO_3 solution (10 mL). The reaction mixture was extracted with EtOAc (3 x 20 mL), and the combined organic phases were washed with brine (15 mL) and dehydrated with Na_2SO_4. The volatiles were evaporated under reduced pressure, and the crude product was purified by column chromatography (200 mL SiO_2, 30 - 50% EtOAc in PE) to give diol **14** (285 mg, 955 µmol, 30%) as a colorless liquid. Diol **14s** (143 mg, 479 µmol, 15%) was also isolated as a colorless liquid. Farnesyl acetate (402 mg, 1.52 mmol, 49%) was recovered.

Analytics for diol **14:**

$[\alpha]_D^{23}$ = +20.9 (*c* = 1.0, MeOH).

^{1}H-NMR (300 MHz, $CDCl_3$, 297 K) δ = 5.34 (dt, *J* = 7.0, 3.4 Hz, 1H), 5.17 (dt, *J* = 7.0, 3.4 Hz, 1H), 4.60 (d, *J* = 7.1 Hz, 2H), 3.36 (d, *J* = 10.4 Hz, 1H), 2.31 – 1.98 (m, 8H), 2.07 (s, 3H), 1.71 (s, 3H), 1.63 (s, 3H), 1.67 – 1.53 (m, 1H), 1.50 – 1.33 (m, 1H), 1.21 (s, 3H), 1.17 (s, 3H) ppm.

^{13}C-NMR (101 MHz, $CDCl_3$, 297 K) δ = 204.3, 171.2, 141.9, 135.3, 124.4, 118.5, 78.0, 72.9, 61.4, 39.4, 36.6, 29.5, 26.4, 25.9, 23.2, 21.0, 16.4, 15.9 ppm.

IR (ATR): $\tilde{\nu}$ = 3446 (*w*, *br*), 3247 (*w*, *br*), 2974 (*w*), 2916 (*w*), 1734 (s), 1708 (s), 1423 (w), 1458 (*w*), 1361 (*w*), 1209 (*w*), 1045 (*w*), 883 (*s*), 818 (*w*) cm^{-1}.

HRMS [EI]: m/z calculated $C_{17}H_{31}O_4$ $[M+H]^+$ 299.2222, observed 299.2216.

The ^{1}H- and ^{13}C-NMR data are matching with the reported literature data.

The enantiomeric excess of **14** was determined as 97% by ^{1}H NMR analysis of its corresponding *mono*-(*S*)-MTPA ester: ^{1}H NMR (500 MHz, $CDCl_3$) δ 3.54 (s, 3H) for the *R* enantiomer; δ 3.47 (s, 3H) for the *S* enantiomer, corresponding to the methyl group on the *α*-methoxy group of the ester. The following is the procedure used for the conversion of **14** to its *mono*-MTPA ester: A mixture of **14** (3 mg, 0.01 mmol), (*S*)-(+)methoxy-α-(trifluoromethyl) phenylacetyl chloride (3.75 µL, 0.042 mmol) and DMAP (5 mg, 0.04 mmol) in dry CH_2Cl_2 (1.0 mL) was stirred at 25 °C for 1 h. The reaction mixture was then passed through a short column of silica gel (10 mL SiO_2, MTBE). The eluent was concentrated to give the desired (*S*)-MTPA ester as a colorless oil, which is used directly for ^{1}H NMR analysis.

(2*E*,6*E*)-9-((*S*)-3,3-Dimethyloxiran-2-yl)-3,7-dimethylnona-2,6-dien-1-ol (29).

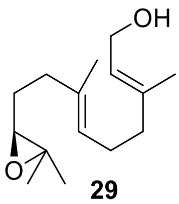
29

The diol **14** (20.2 mg, 68 µmol) was dissolved in anhydrous CH_2Cl_2 (2 mL) under N_2 atm. and cooled down to 0 °C. NEt_3 (14 µL, 0.10 mmol, 1.5 eq.) and MsCl (6 µL, 0.75 mmol, 1.1 eq.) were added at 0 °C, and the solution was stirred for 20 min. The reaction progress was monitored by TLC and reaction mixture was quenched with aq. $NaHCO_3$ solution (2 mL). The aqueous phase was extracted with CH_2Cl_2 (3 x 5 mL) and the combined organic phase was washed with brine (10 mL) and dehydrated with Na_2SO_4. The volatile compounds were evaporated under reduced pressure. The crude product was dissolved in 5 mL MeOH, and K_2CO_3 (94 mg, 0.67 mmol, 10.0 eq.) was added at 25 °C. The reaction mixture was stirred for 30 min at 25 °C before adding a saturated $NaHCO_3$ solution (10 mL). The resulting mixture was extracted with EtOAc (3 x 10 mL), and the combined organic phases were washed with aq. NaCl solution (15 mL). The organic phase was dehydrated with Na_2SO_4, filtered, and the volatiles were evaporated under reduced pressure. The crude product was purified by column chromatography (10 mL SiO_2, 20 - 33% EtOAc in PE) to give epoxide **29** (7.2 mg, 30 µmol, 45%) as a colourless liquid.

$[\alpha]_D^{23}$ = -5.19 (*c* = 1.0, MeOH).

^{1}H-NMR (300 MHz, $CDCl_3$, 297 K) δ = 5.42 (tq, *J* = 7.0, 1.3 Hz, 1H), 5.16 (tq, *J* = 5.5, 1.4 Hz, 1H), 4.15 (d, *J* = 6.9 Hz, 2H), 2.71 (t, *J* = 6.2 Hz, 1H), 2.25 – 1.94 (m, 6H), 1.68 (s, 3H), 1.71 – 1.59 (m, 2H), 1.63 (s, 3H), 1.31 (s, 3H), 1.27 (s, 3H) ppm.

^{13}C-NMR (75 MHz, $CDCl_3$, 297 K) δ = 138.9, 134.2, 124.5, 123.6, 64.1, 59.1, 58.4, 39.3, 36.2, 27.2, 26.1, 24.8, 18.7, 16.1, 15.9 ppm.

IR (ATR): $\tilde{\nu}$ = 3417 (*w*, *br*), 2956 (*w*), 2934 (*w*), 2871 (*w*), 1465 (w), 1400 (*w*), 1384 (*w*), 1249 (*w*), 1038 (*w*), 883 (*s*), 818 (*w*) cm^{-1}.

(2*E*,6*E*)-9-((*S*)-3,3-Dimethyloxiran-2-yl)-3,7-dimethylnona-2,6-dien-1-yl acetate (15)

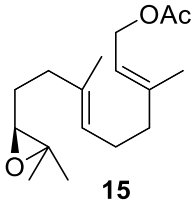
15

To a stirred solution of epoxide **29** (253 mg, 1.06 mmol) in anhydrous CH_2Cl_2 (8 mL) under N_2 atmosphere at 25 °C were added DMAP (162 mg, 1.33 mmol, 1.25 eq.) and Ac_2O (110 µL, 1.17 mmol, 1.1 eq.). After stirring for 1 h at 25 °C were added Et_2O (15 mL) and water (15 mL). The aqueous phase was extracted with Et_2O (2 x 15 mL), and the combined organic phases were washed with 2N HCl solution (20 mL), saturated aq. $NaHCO_3$ solution (20 mL) and brine (20 mL). The organic phase was dehydrated with Na_2SO_4, filtered, and the volatile compounds were evaporated under reduced pressure. The crude product was purified by column chromatography (30 mL SiO_2, 5-10% EtOAc in PE) to give acetate **15** (278 mg, 0.99 mmol, 93%) as a colourless liquid.

$[\alpha]_D^{23}$ = -4.5 (*c* = 1.0, MeOH).

¹H-NMR (300 MHz, $CDCl_3$, 297 K) δ = 5.35 (tq, *J* = 7.0, 1.4 Hz, 1H), 5.14 (qt, *J* = 5.5, 1.4 Hz, 1H), 4.59 (d, *J* = 7.3 Hz, 2H), 2.71 (td, *J* = 6.3, 3.4 Hz, 1H), 2.22 – 2.01 (m, 6H), 2.06 (s, 3H), 1.71 (s, 3H), 1.68 – 1.56 (m, 2H), 1.62 (s, 3H), 1.31 (d, *J* = 2.6 Hz, 3H), 1.27 (d, *J* = 3.2 Hz, 3H) ppm.

¹³C-NMR (75 MHz, $CDCl_3$, 297 K) δ = 171.0, 142.0, 134.5, 124.2, 118.3, 64.1, 61.3, 58.2, 39.3, 36.2, 27.4, 26.1, 24.8, 21.0, 18.7, 16.4, 15.9 ppm.

IR (ATR): $\tilde{\nu}$ = 2936 (*w*), 2834 (*w*), 1734 (*s*), 1449 (w), 1398 (*w*), 1345 (*w*), 1243 (*w*), 1027 (*w*), 863 (*s*), 808 (*w*) cm^{-1}.

The ^{1}H-NMR data are matching with the reported literature data.[7]

((1*S*,4*aR*,6*S*,8*aS*)-6-Hydroxy-5,5,8a-trimethyl-2-methylenedecahydronaphthalen-1-yl)methyl acetate (16)

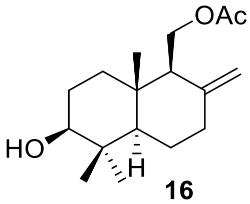

16

To a stirred solution of $TiCp_2Cl_2$ (48 mg, 0.19 mmol, 0.2 eq.) in anhydrous THF (7 mL) at 25 °C was added Mn powder (420 mg, 7.65 mmol, 8.0 eq.) under Ar-atmosphere. After stirring the mixture for 20 min, the colour of the suspension changed to light green. A solution of epoxide **15** (268 mg, 0.95 mmol) and 2,4,6-collidine (1.01 mL, 7.65 mmol, 8.0 eq.) in anhydrous THF (1.5 mL) was added dropwise to the reaction mixture at 25 °C. After stirring 10 min, Me_3SiCl (2.86 mL, 3.82 mmol, 4.0 eq.) was added and reaction mixture was further stirred for 7.5 h at the same temperature. The reaction was stopped by adding 2N aq. HCl (10 mL), and the aqueous phase was extracted with MTBE (2 x 15 mL). The combined organic phases were washed with brine (20 mL), dehydrated with Na_2SO_4, filtered, and the volatile compounds were evaporated under reduced pressure.

The crude product was dissolved in anhydrous THF (2 mL) and TBAF (8.82 mL, 1M in THF, 3.82 mmol, 4.0 eq.) was added at room temperature. After stirring the reaction mixture for 2 h at room temperature, MTBE (15 mL) and H_2O (5 mL) was added. Layers were separated and the aqueous phase was extracted with MTBE (2 x 15 mL). The combined organic phases were washed with brine (20 mL), dehydrated with Na_2SO_4, filtered, and the volatile compounds were evaporated under reduced pressure. The crude product was purified by column chromatography (150 mL SiO_2, 10 - 30% EtOAc in PE) to give polycycle **16** (56 mg, 200 µmol, 21%) as a colourless liquid.

$[\alpha]_D^{23}$ = +8.0 (*c* = 1.0, $CHCl_3$).

^{1}H-NMR (300 MHz, $CDCl_3$, 297 K): δ = 4.88 (d, *J* = 1.5 Hz, 1H), 4.54 (d, *J* = 1.5 Hz, 1H), 4.33 (dd, *J* = 11.3, 3.9 Hz, 1H), 4.19 (dd, *J* = 11.3, 8.9 Hz, 1H), 3.28 (dd, *J* = 11.5, 4.4 Hz, 1H), 2.43 (ddd, *J* = 13.2, 4.3, 2.4 Hz, 1H), 2.10 – 1.97 (m, 2H), 2.02 (s, 3H), 1.81 – 1.64 (m, 4H), 1.63 – 1.56 (m, 1H), 1.41 (dd, *J* = 12.8, 4.2 Hz, 2H), 1.13 (dd, *J* = 12.5, 2.7 Hz, 1H), 1.01 (s, 3H), 0.79 (s, 3H), 0.76 (s, 3H) ppm.

^{13}C-NMR (75 MHz, $CDCl_3$, 297 K): δ = 171.4, 146.1, 107.5, 78.5, 61.4, 54.4, 54.2, 39.1, 38.6, 37.3, 36.9, 28.3, 27.6, 23.4, 21.1, 15.4, 15.0 ppm.

IR (ATR): $\tilde{\nu}$ = 3241 (br, *w*), 2937 (*w*), 1729 (*s*), 1494 (w), 1367 (*w*), 1308 (*w*), 1290 (*w*), 1027 (*w*), 863 (*s*), 808 (*w*) cm^{-1}.

HRMS [EI]: m/z calculated $C_{17}H_{28}O_3$ $[M+H]^+$ 281.2117, observed 281.2111.

The ^{1}H- and ^{13}C-NMR data are matching with the reported literature data.[8]

((1*S*,2*S*,6*S*,8a*S*)-6-Hydroxy-2,5,5,8a-tetramethyldecahydronaphthalen-1-yl)methyl acetate (17)

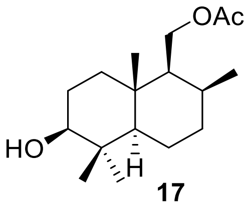

17

To a stirred solution of alcohol **16** (30 mg, 0.10 mmol, 1.0 eq.) in MeOH (5 mL) and Pd/C (0.04 mmol) was added to the solution. The reaction mixture was flushed with H_2 gas for 20 min and stirred for 2 h under the H_2-atmosphere (balloon pressure). The reaction mixture was filtered over celite and washed with MeOH (20 mL). The solvent was evaporated under reduced pressure, and the resulted crude product **17** (28 mg) was used for the next steps without purification.

(2*S*,4a*S*,5*S*,6*S*,8a*R*)-5-(Hydroxymethyl)-1,1,4a,6-tetramethyldecahydronaphthalen-2-ol (18)

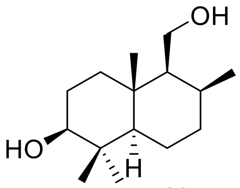

18

To a stirred solution of **17** (28 mg, 0.09 mmol) in MeOH (5 ml) at 25 °C was added 5% KOH solution in MeOH (5 mL) and mixture was allowed to stir at temperature for 4 h. Reaction mixture was concentrated under reduced pressure. The residue was partitioned between Et_2O (30 mL), and water (5 mL). The organic layer was dehydrated over Na_2SO_4, filtered and the volatile compounds were evaporated under reduced pressure. The crude product was purified by column chromatography (40 mL SiO_2, 30 - 50% EtOAc in PE) to give the diol **18** (90%) as a colourless oil.

$[\alpha]_D^{23}$ = +9.5 (*c* = 0.08, $CHCl_3$).

^{1}**H-NMR** (300 MHz, CD_3OD, 297 K) δ = 3.78 (dd, *J* = 10.8, 4.3 Hz, 1H), 3.53 (t, *J* = 10.3 Hz, 1H), 3.16 (dd, *J* = 10.6, 5.8 Hz, 1H), 2.17 (s, 1H), 1.86 – 1.69 (m, 2H), 1.68 – 1.47 (m, 5H), 1.38 – 1.26 (m, 2H), 1.17 (td, *J* = 12.7, 5.1 Hz, 1H), 0.98 (s, 3H), 0.98 (d, *J* = 7.5 Hz, 3H), 0.89 (s, 3H), 0.87 – 0.81 (m, 1H), 0.79 (s, 3H) ppm.

^{13}**C-NMR** (75 MHz, CD_3OD, 297 K) δ = 78.0, 59.1, 55.7, 55.3, 38.6, 38.0, 36.8, 34.3, 28.0, 27.3, 26.3, 16.9, 16.1, 14.7, 14.4 ppm.

IR (ATR): $\tilde{\nu}$ = 3294 (*w*, *br*), 2924 (*m*), 2851 (*w*), 1443 (*w*), 1385 (*w*), 1022 (*s*), 961 (*w*) cm^{-1}.

HRMS [EI]: m/z calculated $C_{15}H_{28}O_2$ $[M-H_2O]^+$ 222.1995, observed 222.1978.

(4a*S*,5*S*,6*S*,8a*R*)-5-(Hydroxymethyl)-1,1,4a,6-tetramethyloctahydronaphthalen-2(1H)-one (19)

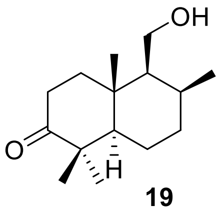

19

To a stirred solution of **17** (20 mg, 0.07 mmol, 1.0 eq.) in anhydrous CH_2Cl_2 (5 mL) at 25 °C was added Dess-Martin-Periodinane (45.0 mg, 0.10 mmol, 1.5 eq.) and mixture was allowed to stir at same temperature for 3 h under N_2 atmosphere. The organic phase was washed with brine (30 mL) and dehydrated with Na_2SO_4. The mixture was filtered, and the volatile compounds were evaporated under reduced pressure. The crude residue (37 mg) was dissolved in MeOH (2 mL) at 25 °C and 5% methanolic K_2CO_3 solution (3 mL) was added. After stirring for 4 h at the same temperature, reaction mixture was concentrated under reduced pressure. Residue was partitioned between Et_2O (30 mL), and water (5 mL). The organic layer was dehydrated over Na_2SO_4, filtered and the volatile compounds were evaporated under reduced pressure. The crude product was purified by column chromatography (40 mL SiO_2, 30 - 50% EtOAc in PE) to give the diol **19** (68%, over two steps) as a colourless oil.

$[\alpha]_D^{23}$ = –156.5 (*c* = 0.08, $CHCl_3$).

^{1}**H-NMR** (300 MHz, CD_3OD, 297 K) δ = 3.79 (dd, *J* = 10.9, 4.4 Hz, 1H), 3.59 (dd, *J* = 10.9, 9.7 Hz, 1H), 2.65 – 2.36 (m, 2H), 2.22 (td, *J* = 4.9, 2.5 Hz, 1H), 2.03 (ddd, *J* = 13.3, 7.4, 4.5 Hz, 1H), 1.78 (d, *J* = 9.0 Hz, 1H), 1.63 (m, 3H), 1.58 – 1.40 (m, 3H), 1.10 (s, 3H), 1.06 (s, 3H), 1.02 (d, *J* = 7.5 Hz, 3H), 0.98 (s, 3H) ppm.

^{13}**C-NMR** (75 MHz, CD_3OD, 297 K) δ = 218.8, 59.2, 55.2, 54.4, 47.2, 38.6, 36.3, 33.5, 33.5, 27.9, 25.7, 20.1, 18.1, 15.8, 13.9 ppm.

IR (ATR): $\tilde{\nu}$ = 3395 (*w*, *br*), 2936 (*m*), 2855 (*w*), 1701 (*s*), 1454 (*m*), 1385 (*m*), 1022 (*m*) cm^{-1}.

HRMS [EI]: m/z calculated $C_{15}H_{26}O_2$ $[M]^+$ 238.1933, observed 238.1927.

Supplementary Note 2: Synthesis of FPP and derivatives

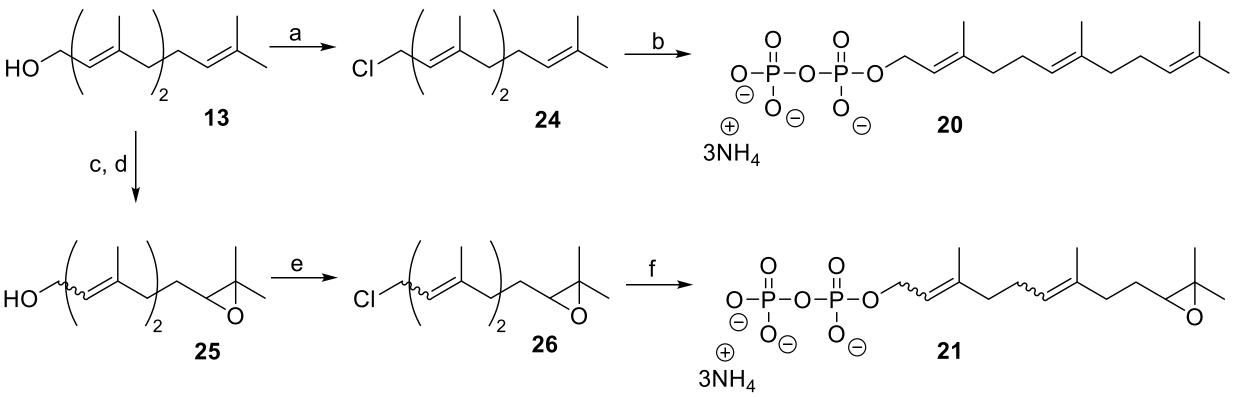

Figure S1. Synthesis of *trans,trans*-farnesyl pyrophosphate ammonium salt and its epoxy derivative as a mixture of isomers.

Farnesyl pyrophosphate (20)

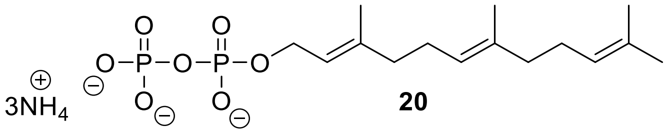

Freshly prepared chloride **24** (325 mg, 1.35 mmol) was dissolved in anhydrous MeCN (7.5 mL) under Ar at room temperature. Tris(tetrabutylammonium) hydrogen pyrophosphate (2435.6 mg, 2.7 mmol, 2.0 eq.) was added in one portion. Reaction was stirred overnight. Afterwards, the reaction mixture was concentrated in *vacuo* and loaded onto ion exchange chromatography.

Resin activation: 60 g of DOWEX 50WX8 resin (Carl Roth; CAS: 11119-67-8, 100-200 mesh, H^+ form) was placed into 1L frit funnel. Resin was transferred into ammonium form by washing (5-times) with 50% NH_4OH solution. Then, it was washed with 100 mL portions of deionized water until pH of filtrate drops to 7. Finally, it was 3-times washed with ion exchange buffer. Resin in ammonium form was suspended in 50 mL of ion exchange buffer and transferred into a glass column.

Ion exchange buffer: Ion exchange buffer (1 L) was prepared by dissolving NH_4HCO_3 (2 g) in deionized water (980 mL) and adding *i*-PrOH (20 mL).

Ion exchange chromatography: Glass column was packed with resin (13.5 x 12.7 cm). Freshly prepared **20** was dissolved in ion exchange buffer (5 mL) and loaded onto a column. First 4 column volumes were collected, transferred into a round bottom flask and freeze-dried to give a white powder.

Precipitation: White powder was dissolved in 50mM NH_4HCO_3 buffer (5 mL) and transferred into falcon tubes. A mixture of MeCN/*i*-PrOH (1:1, 20 mL) was added. The resultant mixture was stirred on vortex for a few minutes until very fine white precipitate appears. The mixture was centrifuged down (5 min, 3000 rpm). Supernatant was collected. Pellet was dissolved

again in 50mM NH_4HCO_3 buffer (5 mL) and the whole procedure was 3-times repeated. Supernatants were collected and freeze dried to give a white powder of *trans,trans*-farnesyl pyrophosphate ammonium salt **20** (390 mg, 67% yield).

^{1}H-NMR (500 MHz, D_2O): δ = 5.49 (t, *J* = 7.1 Hz, 1H), 5.27 – 5.18 (m, 2H), 4.49 (t, *J* = 6.6 Hz, 2H), 2.22 – 2.09 (m, 6H), 2.05 (t, *J* = 7.2 Hz, 2H), 1.75 (s, 3H), 1.71 (s, 3H), 1.65 (s, 6H) ppm.

^{13}C-NMR (125 MHz, D_2O): δ = 144.3, 135.5, 131.5, 124.4, 124.2, 119.9 (d), 62.5, 39.3 (d), 26.4, 26.2, 25.2, 17.2, 15.9, 15.5 ppm.

The analytical data are consistent with literature reports.[9]

10,11-Epoxyfarnesyl pyrophosphate ammonium salt (21)

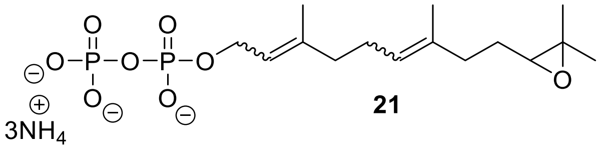

10,11-epoxyfarnesyl pyrophosphate ammonium salt **21** was prepared from epoxyfarnesyl chloride **26** (78 mg, 0.3 mmol) following the same procedure described for **20** and after ion exchange chromatography yielded a white powder (80 mg, 62%).

^{1}H-NMR (500 MHz, D_2O): δ = 5.41 (t, *J* = 6.7 Hz, 1H), 5.22 (t, *J* = 6.4 Hz, 1H), 4.41 (t, *J* = 6.6 Hz, 2H), 2.99 – 2.90 (m, 1H), 2.20 – 2.20 (m, 6H), 1.68 – 1.63 (m, 5H), 1.59 (s, 3H), 1.28 – 1.23 (m, 6H) ppm.

The analytical data are consistent with literature reports.[10]

(*2E,6E*)-1-Chloro-3,7,11-trimethyldodeca-2,6,10-triene (24)

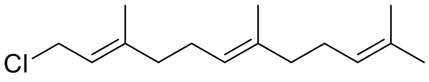

24

Trans,trans-farnesol (300 mg, 1.35 mmol) was dissolved in anhydrous DCM (12.5 mL) under Ar atmosphere and cooled to -40 °C. Subsequently, Et_3N (204.8 mg, 2.02 mmol, 1.5 eq.) and MsCl (201 mg, 1.75 mmol, 1.3 eq.) were added slowly. Resultant mixture was stirred for 60 min at -40 °C, let to warm to room temperature and LiCl (148.7 mg, 3.51 mmol, 2.6 eq.) in anhydrous THF (14 mL) was added dropwise. Mixture was stirred for further 2 h at room temperature. Ice-cooled water was added and the suspension was stirred for 10 min. Layers were separated. Aqueous layer was 3-times extracted with cyclohexane. Organic layers were collected, combined, dried over $MgSO_4$, filtered and concentrated in *vacuo* to afford light yellow oil (325 mg, 1.35 mmol, quant.). The product was immediately introduced into the next step without further purification or characterisation.

Note: The reaction was performed under exclusion of light.

9-(3,3-Dimethyloxiran-2-yl)-3,7-dimethylnona-2,6-dien-1-ol (25)

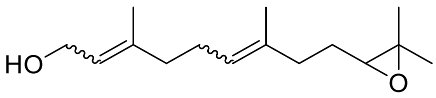

25

Acyl farnesol **27** (3.0 g, 11.35 mmol) was dissolved in THF (14.7 mL). Deionized water (2.6 mL) was added dropwise under vigorous stirring to avoid biphasic system. Forming cloudy solution was cooled to 0 °C. *N*-Bromosuccinimide (2.2 g, 12.5 mmol, 1.1 eq.) was added in one portion. The reaction was let to warm to room temperature and stirred for 1 h. The whole mixture was 3-times extracted with TBME. Organic layers were collected, washed with brine, dried over Na_2SO_4, filtered and concentrated under reduces pressure to give colourless oil. The crude oil was dissolved in MeOH (17.3 mL) and K_2CO_3 (3.1 g, 22.7 mmol, 2.0 eq.) was added in one portion. Formed milky suspension was stirred at room temperature for 48 h. Afterwards, the mixture was diluted wit Et_2O (100 mL) and quenched with sat. aq. NH_4Cl. The suspension was 3-times extracted with EtOAc. Organic layers were collected, combined, extensively washed with brine, dried over Na_2SO_4, filtered and concentrated in *vacuo* to give the crude product, which was purified by flash chromatography to afford an epoxy-farnesol **25** as a colourless oil (2069 mg, 76%).

^{1}H-NMR (300 MHz, $CDCl_3$): δ = 5.36 (t, *J* = 6.4 Hz, 1H), 5.13 (t, *J* = 7.2 Hz, 1H), 4.10 (d, *J* = 6.5 Hz, 2H), 2.73 – 2.63 (m, 1H), 2.21 – 1.94 (m, 7H), 1.72 – 1.65 (m, 2H), 1.63 (s, 3H), 1.59 (s, 3H), 1.27 (d, *J* = 2.3 Hz, 3H), 1.23 (d, *J* = 3.4 Hz, 3H) ppm.

^{13}C-NMR (75 MHz, $CDCl_3$): δ = 138.9 (d), 134.3 (d), 125.3, 124.5, 123.7 (d), 64.1 (d), 59.1 (d), 39.6 (d), 36.3, 28.5, 27.3 (d), 26.2, 24.8 (d), 23.3, 18.7 (d), 16.2 (d) ppm.

3-(9-Chloro-3,7-dimethylnona-3,7-dien-1-yl)-2,2-dimethyloxirane (26)

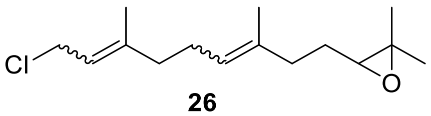

26

Following the same procedure for **24**, an epoxy-farnesyl chloride **26** was prepared from epoxy-farnesol **25** (60 mg, 0.25 mmol) as a light-yellow oil (65 mg, quant.) and was introduced into the next step without further purification and characterisation.

3,7,11-Trimethyldodeca-2,6,10-trien-1-yl acetate (27)

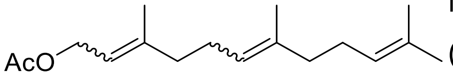

27

Mixture of farnesol isomers (5.0 g, 22.5 mmol) and Et_3N (3.4 g, 33.7 mmol, 1.5 eq.) were dissolved in DCM (50 mL) and cooled to 0 °C. Acetic anhydride (2.98 g, 29.2 mmol, 1.3 eq.) followed by a DMAP (137.4 mg, 1.1 mmol, 0.05 eq.) was added. Resultant mixture was stirred at 0 °C for 2 h. The reaction was quenched with 1M HCl (100 mL) and stirred for further 10 min. Whole was 3-times extracted with EtOAc. Organic layers were collected,

combined, washed with brine, dried over Na_2SO_4, filtered and concentrated in *vacuo* to afford colourless oil (5.87 g, 80%).

^{1}H-NMR (300 MHz, $CDCl_3$,): δ = 5.34 (t, *J* = 6.4 Hz, 1H), 5.15 – 5.04 (m, 2H), 4.58 (d, *J* = 7.2 Hz, 2H), 2.18 – 1.93 (m, 12H), 1.70 (s 3H), 1.68 (s, 3H), 1.59 (s, 3H) ppm.

^{13}C-NMR (75 MHz, $CDCl_3$): δ = 171.1, 142.2, 135.5 (d), 131.5 (d), 124.4, 124.3 (d), 123.6, 118.3 (d), 61.4, 39.7 (d), 39.5, 31.9, 26.6 (d), 26.1 (d), 25.7 (d), 23.3, 21.0, 17.6 (d), 16.4, 16.0 ppm.

3. GC-MS-based Volatile Analysis of *Termitomyces* and Fungus Comb Samples

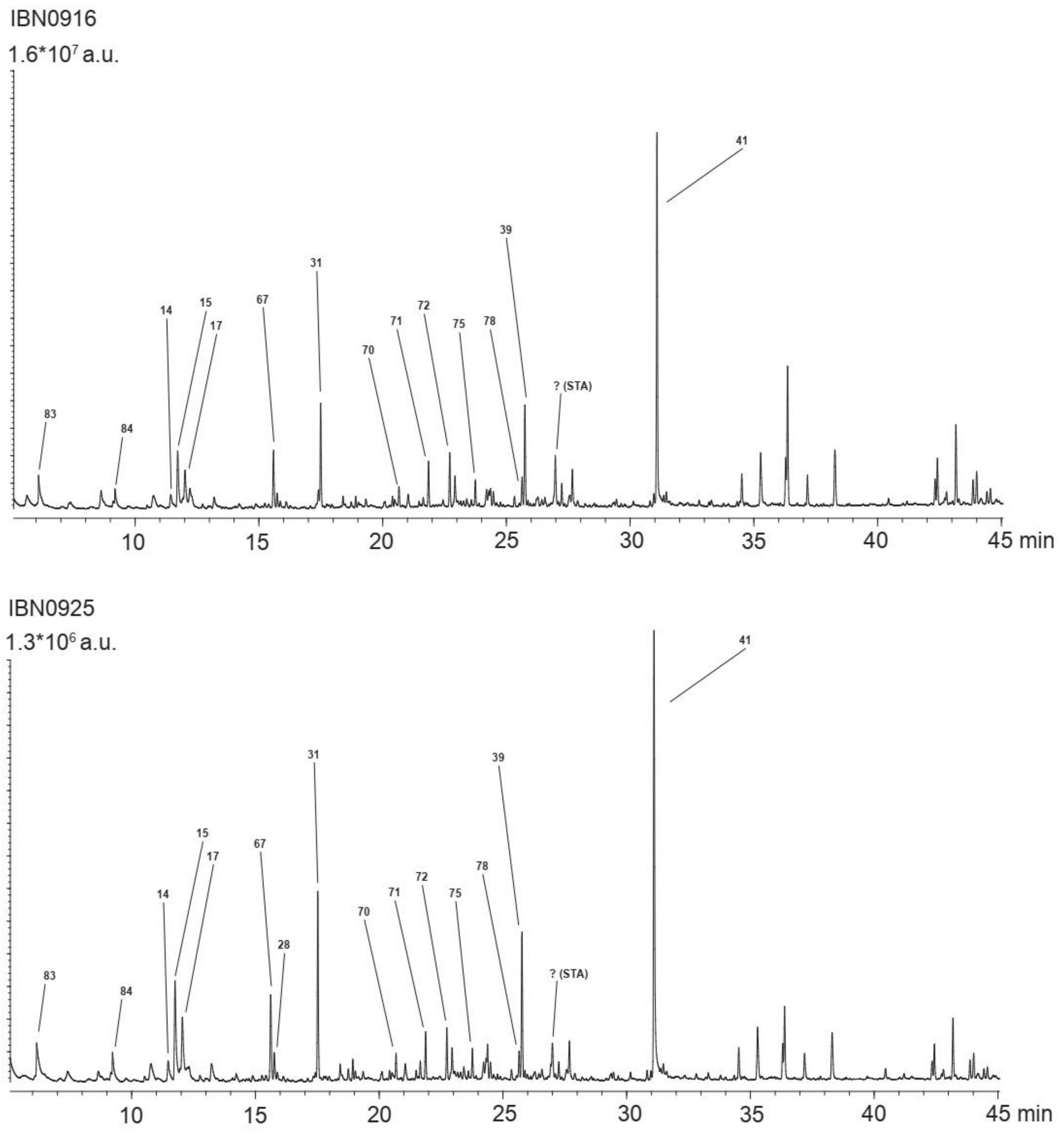

Figure S2. GC-MS chromatograms of volatiles emitted from a *Termitomyces* mushroom (without fungus comb, n = 2).

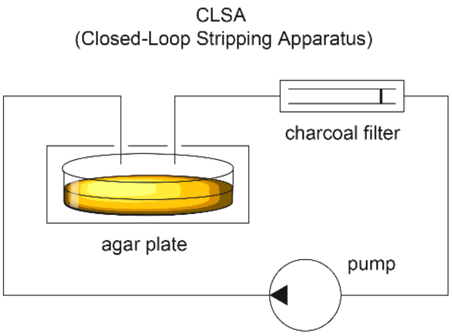

Figure S3. Sketch of the closed-loop stripping apparatus (CLSA).

Table S1. GC-MS analysis of volatiles emitted by a *Termitomyces* mushroom (without fungus comb, n = 2). Numbers of compounds are in accordance with those from the GC-MS chromatogram. Intensity scores indicated as **x**.

Nr. in GC	Compound	RT [min]	*I*	*I* (Lit.)	IBN0916	IBN0925
**83**	dimethylsulfoxid	6,10	839	829	x	xx
**84**	dimethylsulfone	9,19	922	926	x	xx
**12**	benzaldehyde	10,76	964	966	x	x
**14**	oct-1-en-3-ol	11,45	983	986	x	x
**17**	3-octanone	11,73	988	986	xx	xx
**19**	octan-3-ol	12,02	998	996	xx	xx
**67**	linalool	15,60	1102	1100	xx	xx
**28**	nonanal	15,74	1105	1102	x	x
**31**	2-nonenal	17,50	1160	1162	xxx	xxx
**48**	decanal	18,92	1207	1206	x	x
**70**	5-oxocamphor	20,66	1266	1264	x	x
**71**	brasila-1,10-diene	21,86	1308	1307	xx	xx
**72**	brasila-5,10-diene	22,72	1340	1335	xx	xx
**74**	α-cubebene	23,15	1356	1354	x	x
**56**	african-1-ene	23,27	1361	1356	x	x
**75**	brasila-5(10),6-diene	23,75	1378	1370	xx	x
**76**	α-copaene	23,89	1383	1382	x	x
**77**	β-cubebene	24,30	1397	1390	x	x
**57**	α-barbatene	24,87	1420	1415	x	x
**78**	brasila-1(6),5(10)-diene	25,64	1451	1442	xx	xx
**39**	β-barbatene	25,75	1455	1459	xxx	xxx
**80**	α-amorphene	27,10	1508	1506	x	x
**81**	intermedeol	30,98	1672	1669	x	x
**41**	1-tetradecanol	31,08	1678	1667	xxx	xxx
**82**	drimenol	33,29	1778	1777	x	x

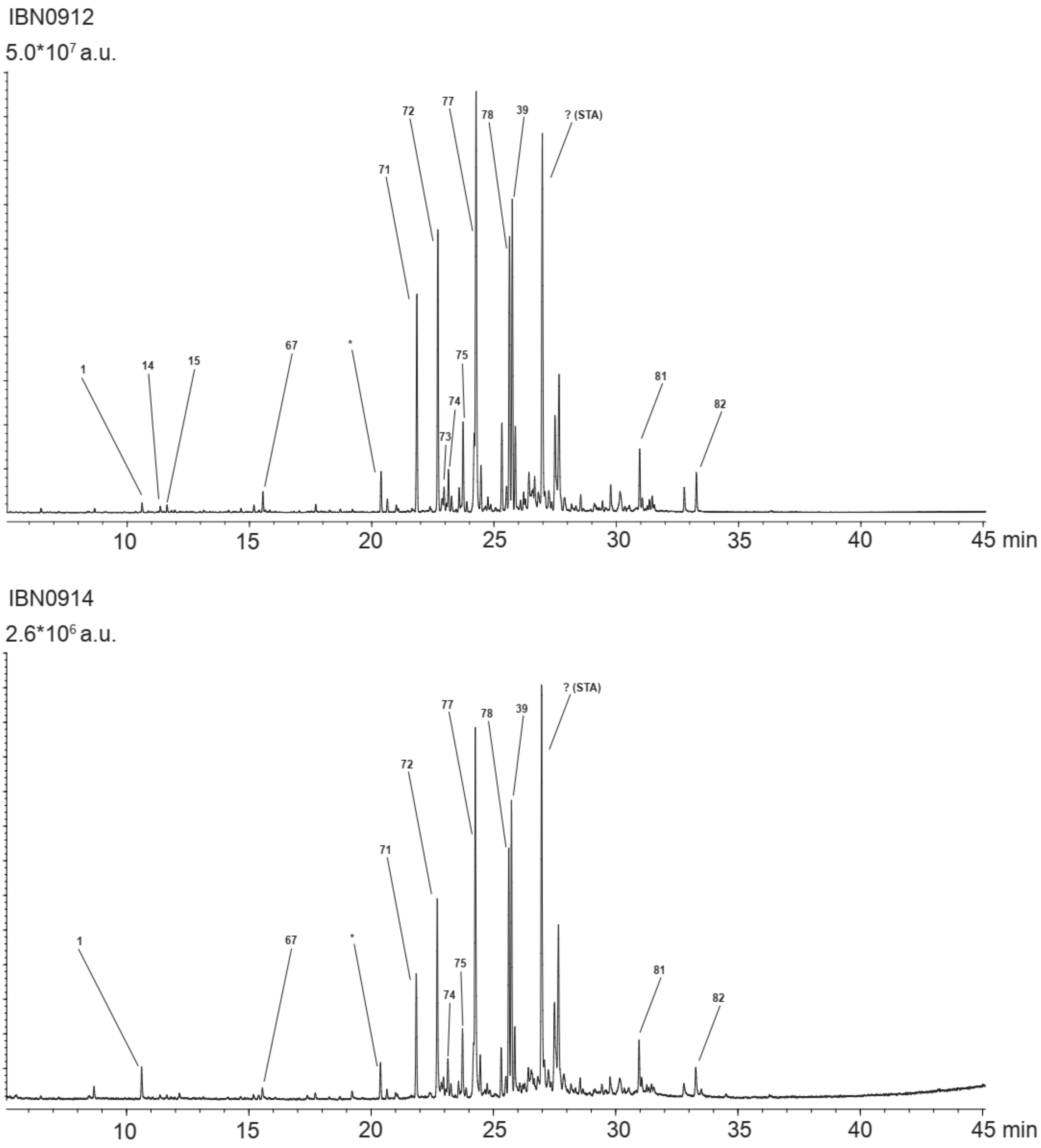

Figure S4. GC-MS chromatograms of volatiles emitted from a *Termitomyces* mushroom emerging from fungus comb material (n=2).

Table S2. GC-MS analysis of volatiles emitted by a *Termitomyces* mushroom emerging from fungus comb (n=2). Numbers of compounds are in accordance with those from the GC-MS chromatogram. Intensity scores indicated as **x**.

Nr. in GC	Compound	RT [min]	*I*	*I* (Lit.)	IBN0912	IBN0914
**12**	benzaldehyde	10,62	960	966	x	x
**14**	oct-1-en-3-ol	11,36	980	986	x	x
**15**	3-octanone	11,65	988	986	x	x
**16**	2-pentylfuran	11,82	992	992	x	
**17**	octan-3-ol	11,97	997	996	x	
**63**	limonene	13,14	1030	1030	x	
**64**	cis-linalool oxide	14,66	1074	1067	x	x
**65**	trans-linalool oxide	15,19	1090	1084	x	x
**66**	methyl benzoate	15,40	1096	1091	x	x
**67**	linalool	15,56	1100	1100	xx	x
**68**	camphor	17,05	1148	1148	x	
**69**	borneol	17,72	1169	1167	x	x
**70**	5-oxocamphor	20,64	1266	1264	x	x
**71**	brasila-1,10-diene	21,85	1308	1307 (!)	xxx	xx
**72**	brasila-5,10-diene	22,71	1340	1335	xxx	xxx
**73**	african-5-ene	22,96	1349	1347	xx	x
**74**	α-cubebene	23,14	1356	1354	xx	x
**56**	african-1-ene	23,27	1361	1356	x	x
**75**	brasila-5(10),6-diene	23,74	1378	1370	xx	x
**76**	α-copaene	23,89	1383	1382	x	x
**77**	β-cubebene	24,27	1397	1390	xxx	xxx
**57**	α-barbatene	24,87	1420	1415	x	x
**78**	brasila-1(6),5(10)-diene	25,64	1451	1442	xxx	xxx
**39**	β-barbatene	25,75	1455	1459	xxx	xxx
**79**	germacrene D	26,67	1491	1487	x	x
**80**	α-amorphene	27,09	1508	1506	x	x
**81**	intermedeol	30,96	1672	1669	xx	xx
**82**	drimenol	33,28	1778	1777	xx	x

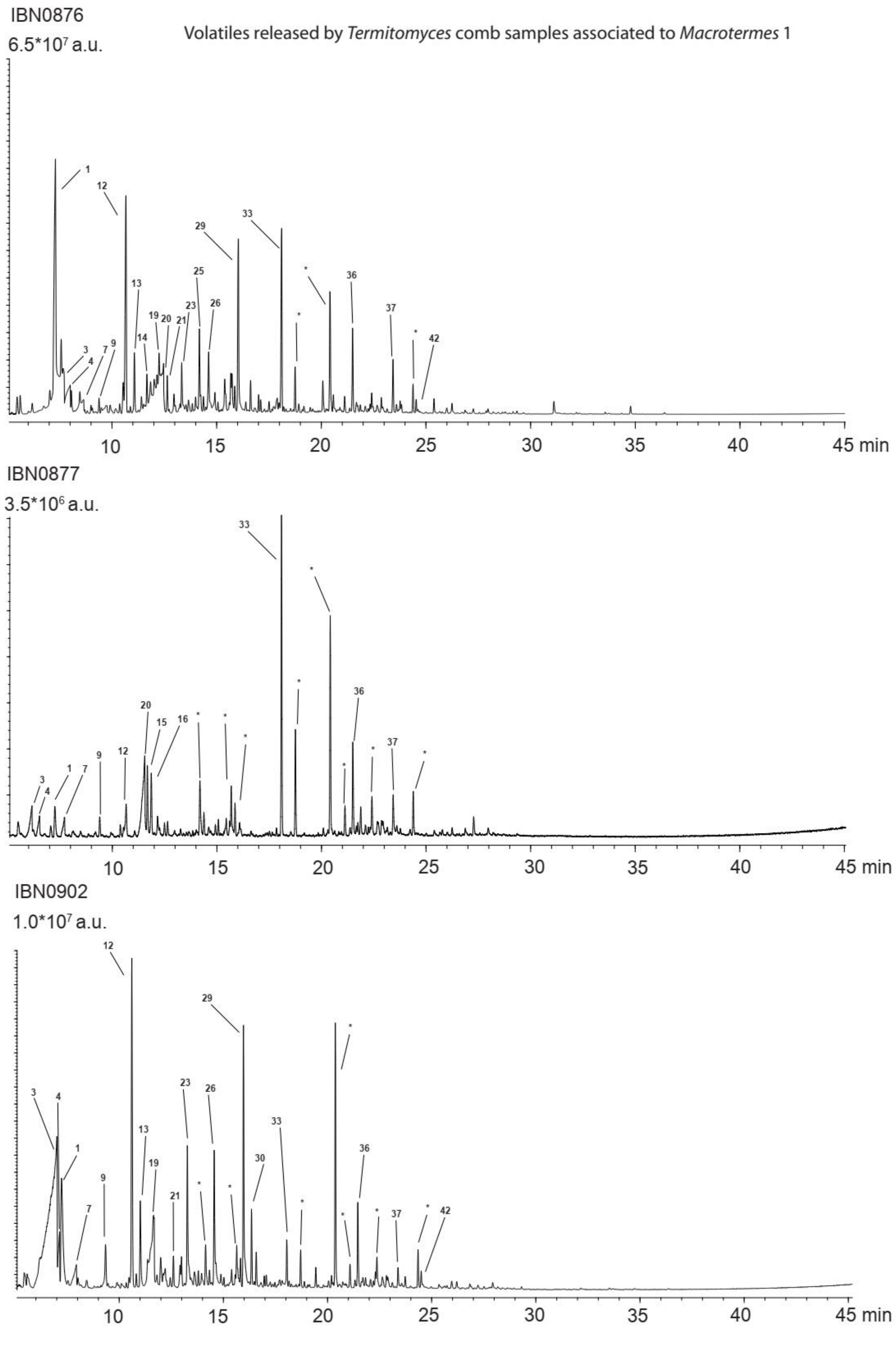

Figure S5. GC-MS chromatograms of volatiles emitted from fungus comb material of *Macrotermes natalensis* colony MN187 (n =3).

Table S3. GC-MS analysis of volatiles emitted by fungus comb material *Macrotermes natalensis* colony MN187 (n =3). Numbers of compounds are in accordance with those from the GC-MS chromatogram. Intensity scores indicated as x.

Nr. in GC	Compound	RT [min]	*I*	*I* (Lit.)	IBN0876	IBN0877	IBN902	IBN903
**1**	hexanol	7,30	871	867	xxx	x	xx	x
**2**	3-methylbutanol acetate	7,58	879	876	x		x	x
**3**	isovaleric acid	7,68	882	888	xxx	x	xxx	x
**4**	2-methyl-butanoic acid	8,00	890	886	xxx	x	x	x
**5**	2-heptanone	8,06	892	889	x			
**6**	heptanal	8,47	903	899	x			x
**7**	pentanoic acid	8,65	908	908	x	x	x	x
**8**	pentyl acetate	9,00	917	916	x			
**9**	methyl hexanoate	9,39	927	924	x	x	x	x
**10**	γ-pentalactone	10,37	954	950	x	x	x	
**11**	(*E*)-2-heptenal	10,48	957	956	x			x
**12**	benzaldehyde	10,67	962	966	xxx	x	xxx	xxx
**13**	heptanol	11,07	972	969	xxx	x	xx	x
**14**	oct-1-en-3-ol	11,41	981	986	x		x	x
**15**	3-octanone	11,67	988	986	xxx	xx	x	
**16**	2-pentylfuran	11,85	993	992	x	xx	x	x
**17**	octan-3-ol	12,02	998	996	x	x	x	
**18**	ethyl hexanoate	12,15	1000	998	x		x	x
**19**	octanal	12,25	1004	1001	x	x	x	x
**20**	hexanoic acid	12,46	1010	981	xxx	xxx	xx	xxx
**21**	hexyl acetate	12,65	1016	1013	xxx		x	
**22**	methyl heptanoate	13,00	1026	1026			x	
**23**	benzyl alcohol	13,34	1036	1034	xxx		xx	x
**24**	γ-hexalactone	14,00	1055	1056	x	x	x	x
**25**	(*E*)-2-octenal	14,18	1060	1056	xx	xx	x	xxx
**26**	octanol	14,62	1073	1074	xx		xx	
**27**	heptanoic acid	14,93	1080	1080	x	x		xx
**28**	nonanal	15,74	1106	1102	x			x
**29**	2-phenylethanol	16,04	1116	1113	xxx		x	x
**30**	methyl octanoate	16,40	1127	1126	x		xx	x
**31**	(*E*)-2-nonenal	17,51	1160	1162	x	x		x
**32**	octanoic acid	17,89	1172	1175	x	x		x
**33**	methyl phenylacetate	18,11	1181	1179	xxx	xxx	x	x
**34**	methyl nonanoate	19,44	1225	1227			x	x
**35**	(*E*)-2-decenal	20,60	1262	1262	x			+
**36**	2-undecanone	21,50	1296	1294	xxx	xx	xx	xxx
**37**	γ-nonalactone	23,43	1367	1362	xx	x	x	x
**38**	6,10-dimethylundecan-2-one	24,54	1408	1407	x		x	
**39**	β-barbatene	25,73	1454	1459				x
**40**	dihydroactinidiolide	27,88	1538	1538	x			
**41**	tetradecanol	31,12	1679	1676	x			
**42**	6,10,14-trimethylpentadecan-2-one	34,78	1849	1847	x		x	

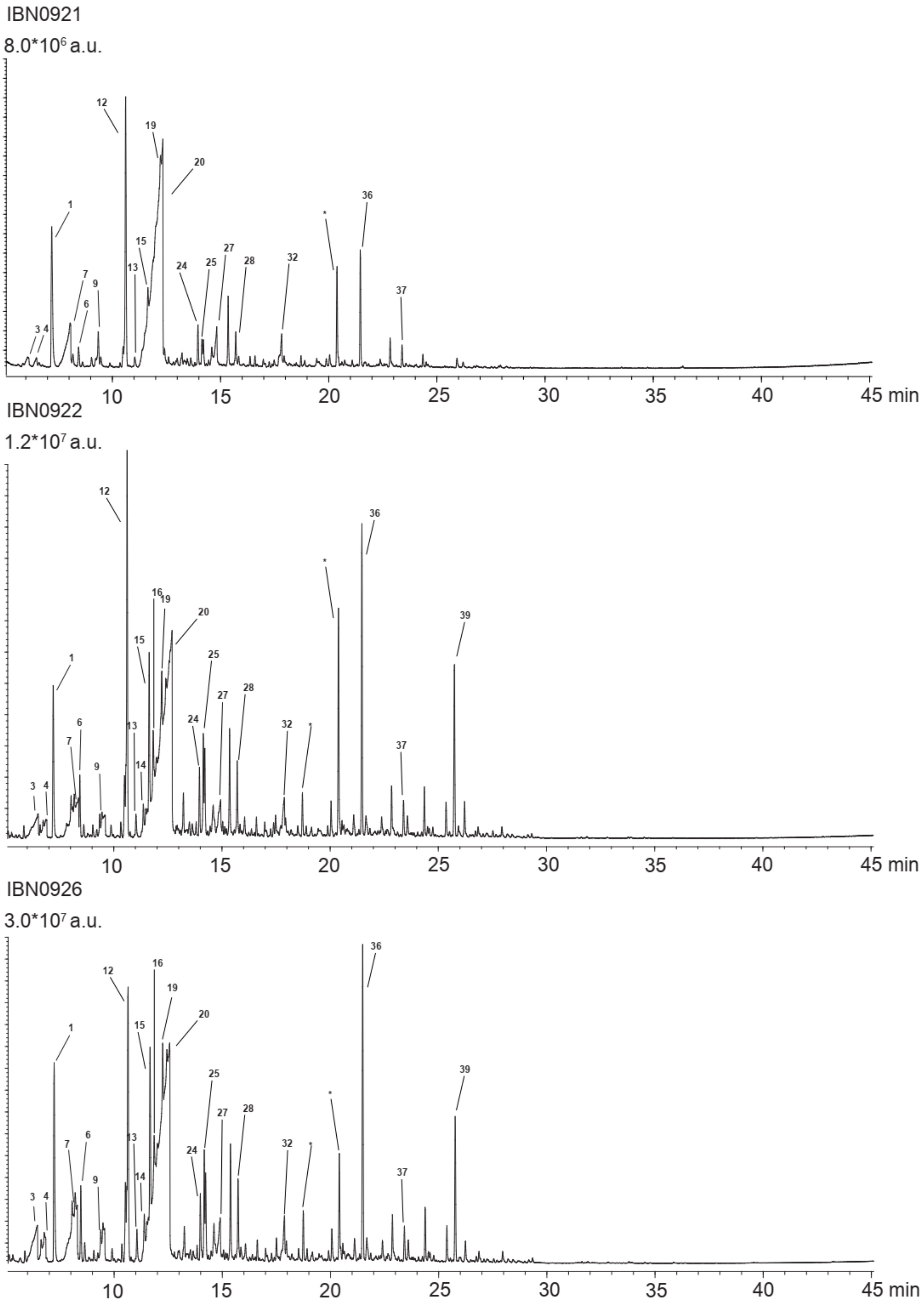

Figure S6. GC-MS chromatograms of volatiles emitted from fungus comb material from *Macrotermes natalensis* colony MN188 (n = 3).

Table S4. GC-MS analysis of volatiles emitted by fungus comb material from *Macrotermes natalensis* colony MN188 (n = 3). Numbers of compounds are in accordance with those from the GC-MS chromatogram Figure S5, Figure S6). *Intensity scores indicated as **x**.*

Nr. in GC	Compound	RT [min]	*I*	*I* (Lit.)	IBN0921	IBN0922	IBN0926
**43**	Furfural	5,85	832	835	x	x	x
**44**	(*E*)-2-hexenal	6,60	852	855	x	x	x
**3**	isovaleric acid	6,09	839	888	x	xx	xx
**4**	2-methylbutanoic acid	6,49	849	886	x	x	x
**1**	Hexanol	7,20	869	867	xxx	xxx	xxx
**7**	pentanoic acid	8,05	892	908	xxx	xxx	xxx
**6**	Heptanal	8,44	902	899	x	xx	xx
**9**	methyl hexanoate	9,35	926	924	xxx	x	x
**45**	2-methylpentanoic acid	9,60	933	935	x	x	x
**10**	γ- pentalactone	10,36	954	950	x	x	x
**11**	(*E*)-2-heptenal	10,50	957	956	xx	xx	xx
**12**	Benzaldehyde	10,62	960	966	xxx	xxx	xxx
**13**	Heptanol	11,04	972	969	x	x	x
**14**	oct-1-en-3-ol	11,37	980	986		x	x
**15**	3-octanone	11,65	988	986	x	xxx	xxx
**16**	2-pentylfuran	11,82	992	992	x	xx	xx
**17**	octan-3-ol	11,98	997	996		x	x
**19**	Octanal	12,23	1003	1001	x	xx	xx
**20**	hexanoic acid	12,33	1006	981	xxx	xxx	xxx
**24**	γ-hexalactone	13,95	1054	1056	x	xx	xx
**25**	(*E*)-2-octenal	14,14	1059	1056	x	xx	xx
**27**	heptanoic acid	14,83	1079	1080	xxx	xx	x
**28**	Nonanal	15,71	1105	1102	xx	xx	xx
**30**	methyl octanoate	16,37	1126	1126	x	x	x
**46**	γ-heptalactone	17,25	1154	1163	x	x	x
**32**	octanoic acid	17,83	1172	1175	x	x	xx
**47**	2-decanone	18,51	1193	1191		x	x
**48**	Decanal	18,90	1207	1206	x	x	x
**34**	methyl nonaoate	19,45	1226	1227	x		x
**35**	(*E*)-2-decenal	20,55	1263	1265	x	x	x
**36**	2-undecanone	21,47	1295	1294	xxx	xxx	xxx
**37**	γ-nonalactone	23,39	1365	1362	xx	xx	x
**39**	β-barbatene	25,75	1455	1459		xxx	xx

4. Cultivation and GC-MS-based Volatile Analysis of *Termitomyces* cultures

Table S5. Composition of cultivation media (for solid agar: 20.0 g/L agar-agar was added)

	Chemical	Company	Amount
**MA**	Malt extract	Carl Roth	30.0 g/L
	Peptone	Carl Roth	5.0 g/L
**PDB**		Carl Roth	26.5 g/L
**YM**	Dextrose	Carl Roth	10.0 g/L
	Peptone	Carl Roth	5.0 g/L
	Yeast extract	Carl Roth	3.0 g/L
	Malt extract	Carl Roth	3.0 g/L
**Minimal medium**	$MgSO_4$ x 7 H_2O	Carl Roth	0.5 g/L
	L-Asparagine x H_2O	Carbolution	1.5 g/L
	Solution A	See below	1.0 mL/L
	$FeCl_3$ x 6 H_2O	Alfa Aesar	3.0 mg/L
	Solution C	See below	1 mL/l
	Solution D	See below	2.5 mL/l
	Agar-Agar	Carl Roth	20 g/L
	Urea	Carl Roth	20 mM
	Carbon source	See below	
**Carbon source**	PDB	Carl Roth	26.5 g/L
	Cellulose	Alfa Aesar	30 g/L
	Fungus comb		30 g/L

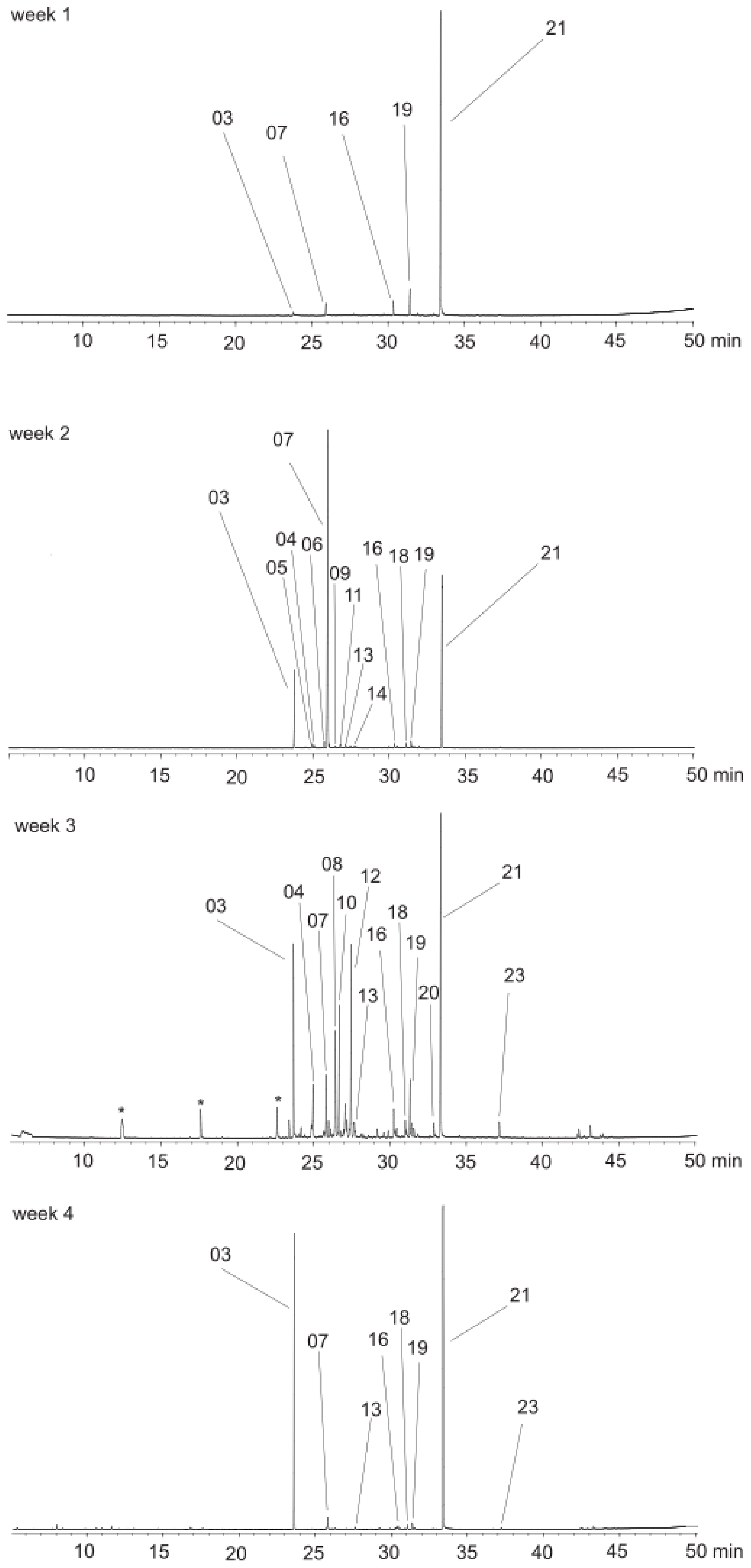

Figure S7. GC-MS chromatograms of volatile time study from *Termitomyces* strain T153 growing on PDA for 1-4 weeks.

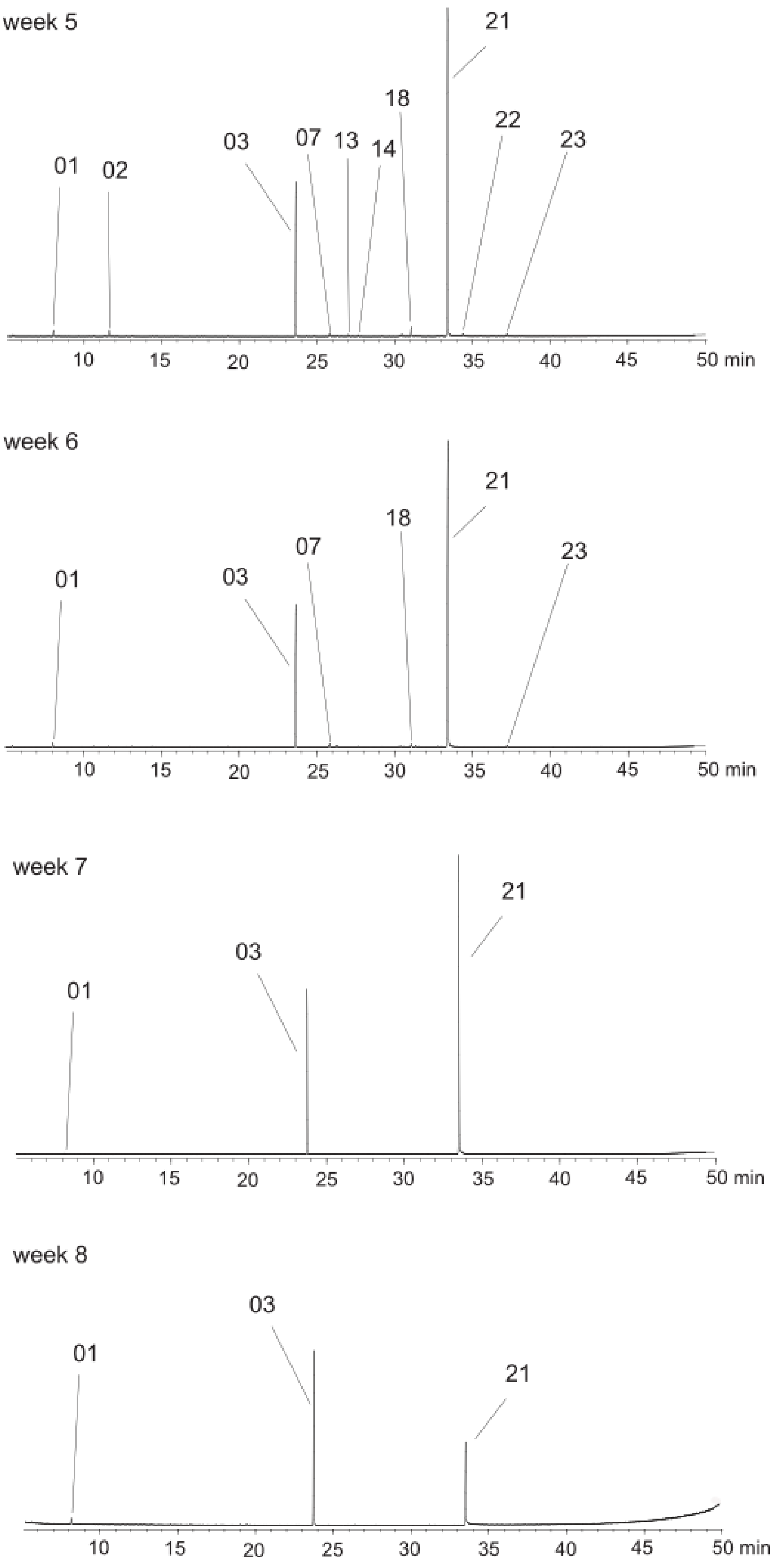

Figure S8. GC-MS chromatograms of volatile time study from *Termitomyces* strain T153 growing **on PDA** for 5-8 weeks. Detected compounds are listed above.

Table S6. GC-MS analysis of volatiles emitted by *Termitomyces* sp. T153 growing on PDA plates over 8 weeks. Numbers of compounds are in accordance with those from the GC-MS chromatogram.

Nr. in GC	Compound	RT [min]	Week							
			1	2	3	4	5	6	7	8
01	Styrene	8.80					x	x	x	x
02	3-octanone	12.39					x			
03	1,2,4-trimethoxybenzene	23.77	x	xx	xx	xxx	xx	xx	xx	xxx
04	α-Barbatene	24.84		x	x					
05	Longifolene	24.95		x	x					
06	isobazzanene	25.73		x						
07	β-barbatene	25.93	xx	xxx	x	x	x	x		
08	best database hit: himachalene	26.29			x					
09	β-acoradiene	26.48		x						
10	best database hit: himachalene	26.55			x					
11	β-chamigrene	26.79		x						
12	best database hit: eremophilene	27.32			xx					
13	unknown	27.79		x	x	x	x	x		
14	unknown	28.41		x			x			
15	unknown	29.99				x				
16	oxidised terpenoid; similar to drimenol	30.35	xx	x	x	x				
17	unknown	31.23					x			
18	Intermedeol	31.12		x	x	x	xx	x	x	
19	oxidised terpenoid; similar to drimenol	31.49	xx	x	x	x	x	x	-	-
20	oxidised terpenoid; similar to albaflavenol	32.76			x					
21	Drimenol	33.44	xxxx	xx	xxx	xxx	xxx	xxx	xxx	xx
22	unknown	35.14					x			
23	unknown	37.05			x	x	x	x		

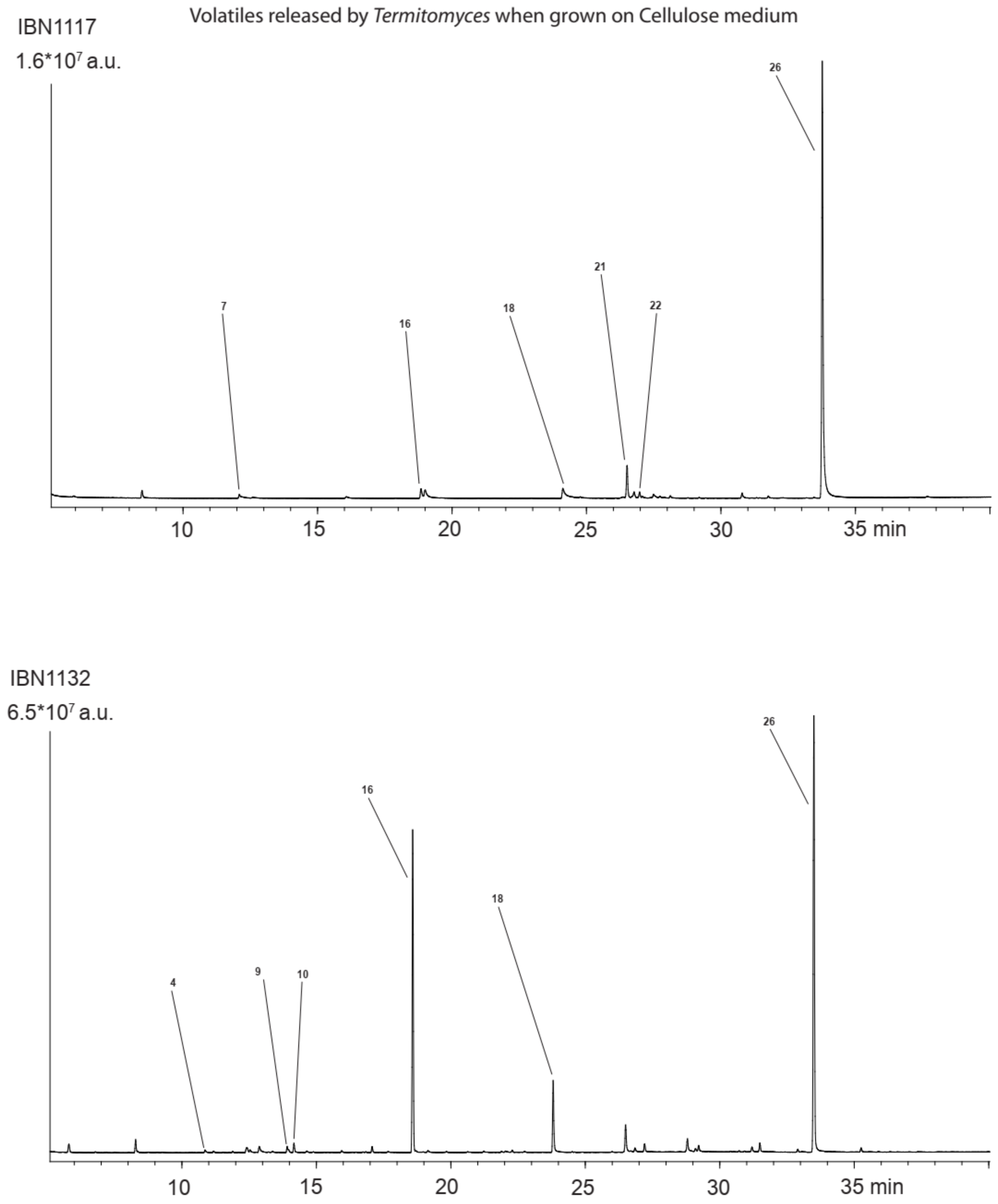

Figure S9. GC-MS chromatograms of volatile time study from *Termitomyces* strain T153 growing on cellulose medium after one (IBN 1117) and two weeks (IBN 1132).

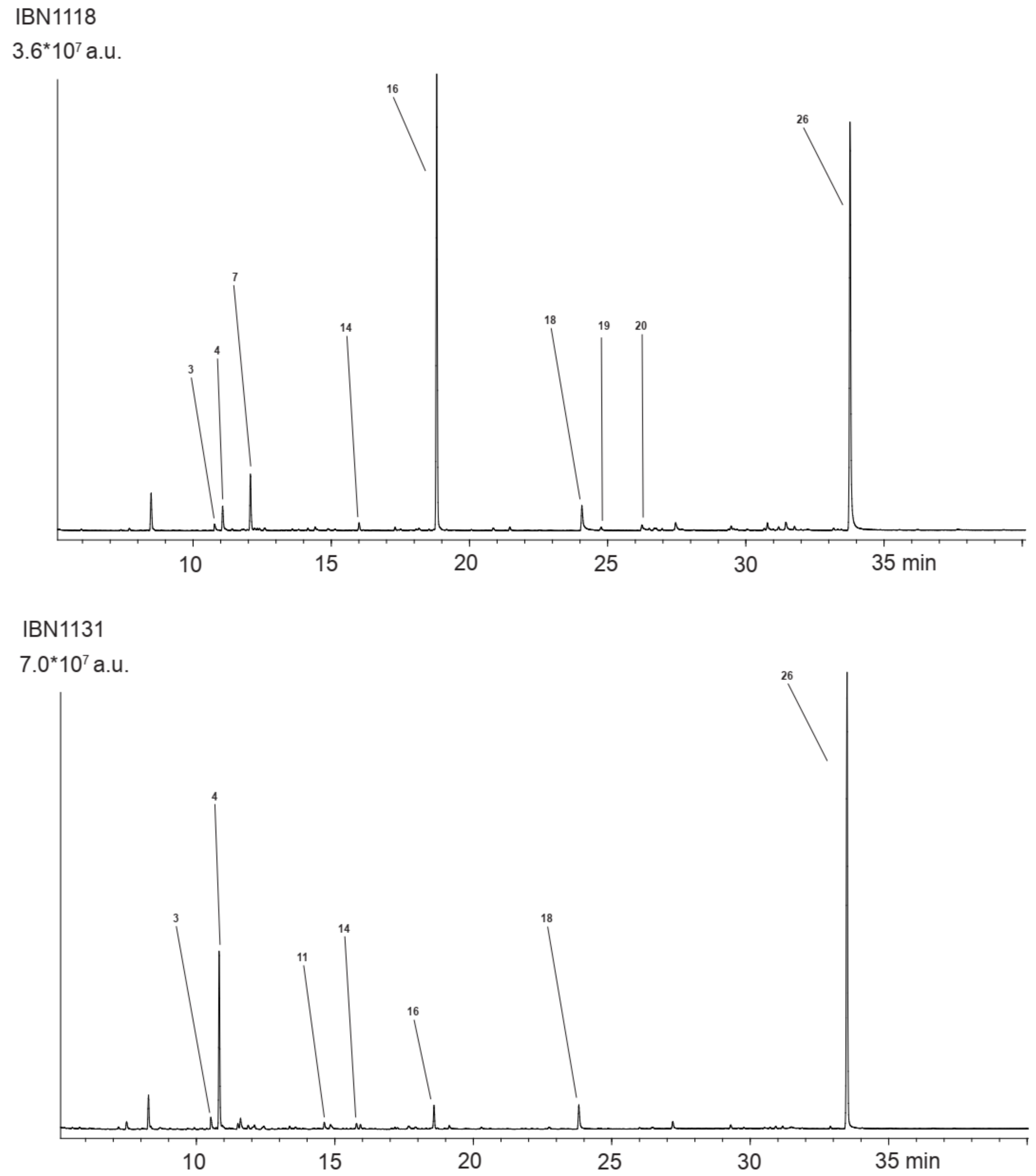

Figure S10. GC-MS chromatograms of volatile time study from *Termitomyces* strain T153 growing on fungus comb medium after one (IBN 1118) and two (BN1131) weeks.

Table S7. GC-MS analysis of volatiles from *Termitomyces* on different media after one and two weeks of growth. Numbers of compounds are in accordance with those from the GC-MS chromatogram ***Figure S9***, ***Figure S10***.

					**PDA**		**Celllose**		**FCA**	
Nr. in GC	Compound	RT [min]	*I*	*I* (Lit.)	IBN1116 (1 week)	IBN1122 (2 weeks)	IBN1117 (1 week)	IBN1132 (2 weeks)	IBN1118 (1 week)	IBN1131 (2 weeks)
**1**	α-pinene	10,12	932	932	x	x			x	x
**2**	2-methyl-γ-butyrolactone	10,62	946	957		x				
**3**	5,5-dimethyl-2(5H)-furanone	10,78	950	952					x	x
**4**	benzaldehyde	11,06	958	966		x		x	xx	xx
**5**	isopropylpyrazine	11,18	961					x		
**6**	3-octenol	11,83	978	976					x	x
**7**	3-octanone	12,07	985	986	xx	xx	x	x	xx	x
**8**	3-octanol	12,38	993	996		x			x	
**9**	2-methyl-3-isopropyl-pyrazine	14,15	1044			x		x	x	
**10**	2-methyl-5-isopropyl-pyrazine	14,17	1045	1059				x		
**11**	acetophenone	14,65	1059	1065				x	x	x
**12**	linalool oxide	15,12	1072	1078		x			x	x
**13**	nonanal	15,95	1096	1102				x		x
**14**	linalool	16,00	1098	1100	x	x	x		x	x
**15**	2,6,6-trimethyl-2-cyclohexene-1,4-dione	17,18	1134	1140						x
**16**	2,5-diisopropylpyrazine	18,81	1186		xx	xx	x	xxx	xxx	xx
**17**	4-phenyl-2-butanone	20,29	1235							x
**18**	1,2,4-trimethoxybenzene	24,07	1368		xx	x	xx	xx	xx	xx
**19**	isolongifolene	24,77	1394	1386					x	x
**20**	geranylacetone	26,23	1451	1454	x			x	x	x
**21**	amorpha,4-11-diene	26,49	1461	1482	x	x	xx		x	
**22**	α-neocallitropsene	26,96	1480	1493	x	x	x		x	
**23**	β-bisabolene	27,74	1511	1506			x			
**24**	β-sesquiphellandrene	28,12	1527	1525			x			
**25**	intermedeol	31,45	1669	1666					x	x
**26**	drimenol	33,76	1775	1761	xxx	xxx	xxx	xxx	xxx	xxx

5. Isolation and Structure Elucidation of Drimenol Derivatives

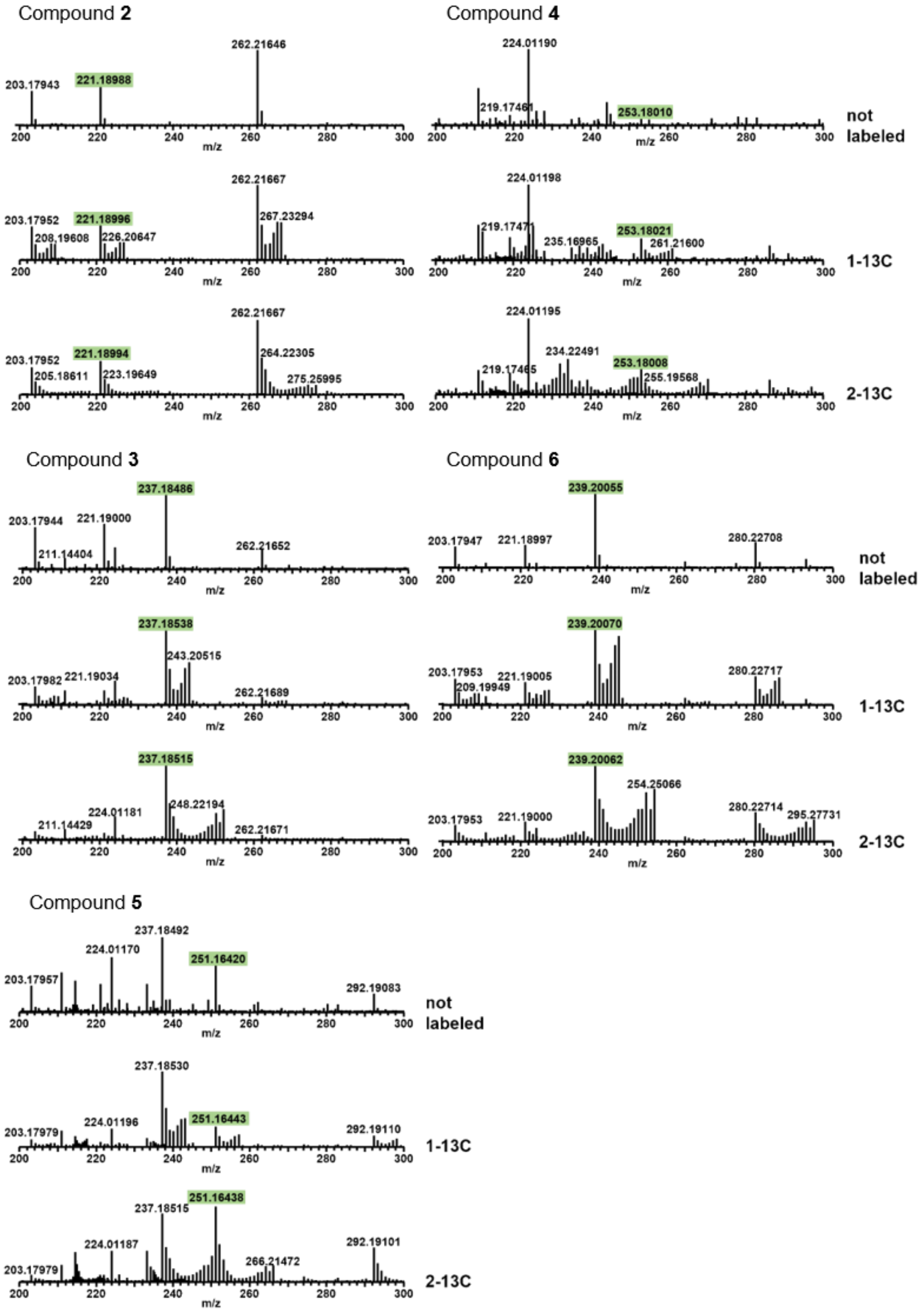

Figure S11. HR-MS spectra of drimenol derivatives **2-6** ([M+H] marked in green) detected from extracts of *Termitomyces* sp. T153 grown on ^{13}C-enriched medium.

Table S8. NMR spectral data ($CDCl_3$, at 300 K) for compound **2**.

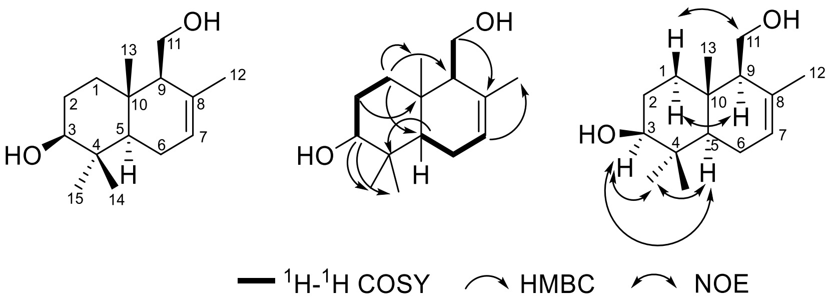

Pos.		δ$_H$, mult. (*J* in Hz)[a]	δ$_C$, type[b]	COSY[a]	HMBC[a]	TOCSY[a]	NOESY[a]
1	a	2.01, m	38.1, t	1b, 2a	2, 3, 4, 5, 13		
	b	1.24, m		1a ,2b	2, 3, 4, 9, 13	1a, 2a, 3	9
2	a	1.65, m	27.4, t	1a	1, 3, 10	1b	
	b	1.63, m		1b, 3	1, 3		13
3		3.25, dd (*11.0, 4.8*)	79.2, d	2b	1, 14, 15	1a, 1b, 2a	2b, 5, 15
4			36.0, s				
5		1.18, d (*5.2*)	49.5, d	6b	1, 2, 4, 6, 7, 9, 13, 14, 15	6a	15
6	a	1.98, m	23.4, t			5, 7	14
	b	1.96, m		5			
7		5.53, m	124.1, d	6a, 12	9, 12	6b	6a, 12
8			132.9, s				
9		1.84, br. m.	57.2, d	11a, 11b		11a	1b
10			38.8, s				
11	a	3.85, dd (*11.3, 3.6*)	60.9, t	9, 11b	4, 8, 9	9, 11b	1a, 9
	b	3.73, dd (*11.3, 5.2*)		9, 11a	4, 8, 9	9, 11a	
12		1.77, s	21.9, q	7	7, 8, 9	5	
13		0.85, s	15.1, q		9, 10		
14		0.86, s	15.5, q		3, 4, 5		6b
15		0.98, s	28.2, q		3, 4, 5		6a

[a] 600 MHz for ^{1}H NMR, COSY, TOCSY, HMBC, HSQC, [b] 150 MHz for ^{13}C, DEPT

[b] numbers of attached protons were determined by analysis of DEPT and 2D spectra.

Table S9. Comparison of NMR spectral data of compound **2** with literature data reported for compound **3**[11] and **31a**.[12]

		**Literature**		**Compound 2**	
**Pos.**		**δ_H, mult. (*J* in Hz)**[12]	**δ_C, type**[13]	**δ_H, mult. (*J* in Hz)**	**δ_C, type**
1	a		38.9	1.29, m	39.0, t
	b			1.28, td *(13.5, 4.0)*	
2	a		28.1	1.66, m	28.1, t
	b			1.62, m	
3		3.26, dd (*10.5, 5.4*)	79.6	3.19, dd (*11.3, 4.6*)	79.7, d
4			39.7		39.8, s
5			51.0	1.19, q (*5.5*)	51.0, d
6	a		24.2	2.05, dt (*13.5, 3.4*)	24.4, t
	b			1.98, m	
7		5.54, m	123.7	5.46, m	123.8, d
8			134.9		135.0, s
9			58.2	1.81, m	58.3, d
10			36.8		36.9, s
11	a	3.86, dd (*11.1, 3.4*)	61.2	3.80, dd (*11.1, 3.2*)	61.2, t
	b	3.75, dd (*11.5, 5.0*)		3.57, dd (*11.1, 6.5*)	
12		1.78, br s	22.2	1.75, s	22.1, q
13		0.98, s	28.7	0.96, s	28.7, q
14		0.87, s	15.9	0.85, s	15.9, q
15		0.86, s	14.9	0.81, s	14.9, q

Table S10. NMR spectral data ($CDCl_3$, at 300 K) for compound **3**.

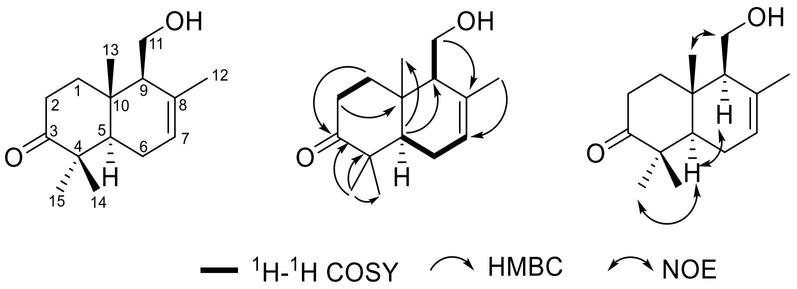

Position		δ_H, mult. (*J* in Hz)[a]	δ_C, type[b]	COSY[a]	HMBC[c]	NOE[c]
1	a	2.74, td (*15.31, 5.96*)	34.7, t	1b, 2a, 2b	2, 3	
	b	2.28, m		1a, 2b	10	
2	a	2.29, m	38.6, t	2b	10	
	b	1.56, td (*12.07, 4.43*)		1a, 1b, 2a		
3			216.7, s			
4			47.6, s			
5		1.61, dd (*11.87, 3.76*)	51.4, d	6a, 6b	9, 10, 13	2a, 9, 15
6	a	2.08, m	24.0, t	5, 6b, 7		
	b	1.95, m		5, 6a, 7		
7		5.58, m	123.9, d	6a, 6b, 12		
8			133.1, s			
9		1.92, m	56.2, d	11a, 11b, 12		
10			36.0, s			
11	a	3.88, dd (*11.16, 3.79*)	60.8, t	9	8, 10	
	b	3.81, dd (*11.16, 3.70*)				
12		1.79, s	21.9, q	7,9	7, 8, 9	
13		1.100, s	14.7, q		2, 9, 10	1a, 2a, 6a, 11a, 11b
14		1.104, s	22.5, q		3, 4, 5, 15	
15		1.06, s	25.4, q		3, 4, 5, 14	5, 6b

[a] 600 MHz for ^{1}H NMR, COSY, [b] 150 MHz for ^{13}C, [c] 500 MHz for HSQC, HMBC, NOE

[b] numbers of attached protons were determined by analysis of 2D spectra.

Table S11. Comparison of NMR spectral data of compound **3** with literature data.[14]

		**Literature**		**Compound 3**	
**Position**		**δ_H, mult. (*J* in Hz)**	**δ_C, type**	**δ_H, mult. (*J* in Hz)[a]**	**δ_C, type[b]**
1	a	2.74 dd (*13.9, 6.1*)	34.5, t	2.74, td (*15.31, 5.96*)	34.7, t
	b	2.28, m		2.28, m	
2	a	1.56, m	38.5, t	2.29, m	38.6, t
	b	2.30, m		1.56, td (*12.07, 4.43*)	
3			216.7, s		216.7, s
4			47.5, s		47.6, s
5		1.60, m	51.1, s d	1.61, dd (*11.87, 3.76*)	51.4, d
6	a	1.94, m	23.8, t	2.08, m	24.0, t
	b	2.08, m		1.95, m	
7		5.58, m	123.7, d	5.58, m	123.9, d
8			132.9, s		133.1, s
9		1.92, m	56.0 d	1.92, m	56.2, d
10			35.8 s		36.0, s
11		3.82, dd (*10.9, 3.9*)	60.6 t	3.88, dd (*11.16, 3.79*)	60.8, t
		3.87, dd (*10.9, 3.9*)		3.81, dd (*11.16, 3.70*)	
12		1.78, d (*1.5*)	21.7 q	1.79, s	21.9, q
13		1.06, s	25.2 q	1.100, s	14.7, q
14		1.10, s	22.3 q	1.104, s	22.5, q
15		1.09, s	14.5 q	1.06, s	25.4, q

Table S12. NMR spectral data ($CDCl_3$, at 300 K) for compound **4**.

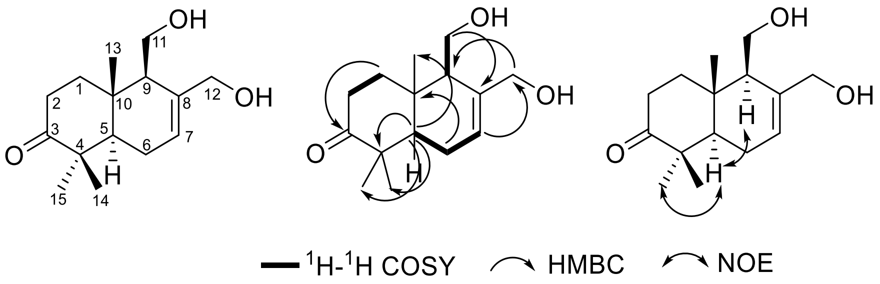

Pos.		δH, mult. (*J* in Hz)[a]	δC, type[b]	COSY[a]	HMBC[a]	NOE[a]	TOCSY[a]
1	a	2.75, m	34.6, t	1b, 2b	2, 3		
	b	2.29, m		1a	2, 5, 10		
2	a	2.22, m	38.1, t	2b	1		
	b	1.57, m		1a, 1b, 2a	1		1b
3			215.8, s				
4			47.6, s				
5		1.68, m	50.5, d	6a, 6b	4, 6, 9, 10, 13, 14, 15	9, 15	6a, 14, 15
6	a	2.13, m	23.9, t	5, 7	5		5, 7
	b	2.08, m		7	5, 10		
7		5.87, s	127.4, d	6a, 6b	5, 6, 12		6a
8			134.5, s				
9		2.27, m	54.5, d	11a	10	1a, 5	11a, 11b
10			35.5, s				
11		4.19, m	61.7, t	9,12b	8, 10		9
		4.11, m		9	8, 9, 10		9
12	a	4.43, dd (*36.76, 12.47*)	67.5, t	12b	7, 8, 9		
	b	4.38 dd (*20.25, 8.78*)		11a, 12a	7 ,8, 9		
13		1.07, s	14.5, s		2, 9, 10		
14		1.11, s	22.2, s		3, 4, 5, 15	1a, 6a	
15		1.07, s	25.1, s		3, 4, 5, 14		

[a] 600 MHz for ^{1}H NMR, COSY, [b] 150 MHz for ^{13}C, DEPT

[b] numbers of attached protons were determined by analysis of 2D spectra and DEPT.

Table S13. Comparison of NMR spectral data of compound **4** with literature data.[15]

		Literature		Compound 4	
Pos.		δ_H, mult. (*J* in Hz)	δ_C, type	δ_H, mult. (*J* in Hz)	δ_C, type
1	a	2.76, ddd (*14.5, 14.5, 5.3*)	35.1, t	2.75, m	34.6, t
	b	2.14, ddd (*14.5, 3.7, 3.7*)		2.29, m	
2	a	2.34, ddd (*13.3, 5.3, 3.7*)	38.7, t	2.22, m	38.1, t
	b	1.59, ddd (*14.5, 13.3, 3.7*)		1.57, m	
3			215.1, s		215.8, s
4			47.9, s		47.6, s
5		1.63, dd (*11.8, 4.6*)	51.8, d	1.68, m	50.5, d
6	a	2.14, overlapped	24.4, t	2.13, m	23.9, t
	b	2.01, m		2.08, m	
7		5.78, d (*5.1*)	125.0, d	5.87, s	127.4, d
8			139.3, s		134.5, s
9		2.14, overlapped	54.7, d	2.27, m	54.5, d
10			36.2, s		35.5, s
11		3.70, ddd (*11.1, 5.0, 2.5*)	60.9, t	4.19, m	61.7, t
		3.90, ddd (*11.1, 6.9, 5.0*)		4.11, m	
12	a	3.96, dd (*12.2, 6.9*)	66.7, t	4.43, dd (*36.76, 12.47*)	67.5, t
	b	4.27, dd (*12.2, 5.3*)		4.38 dd (*20.25, 8.78*)	
13		1.04, s	14.4, q	1.07, s	25.1, s
14		1.08, s	22.5, q	1.11, s	22.1, s
15		1.02, s	25.7, q	1.07, s	14.54, s

Table S14. NMR spectral data ($CDCl_3$, at 300 K) for compound **5**.

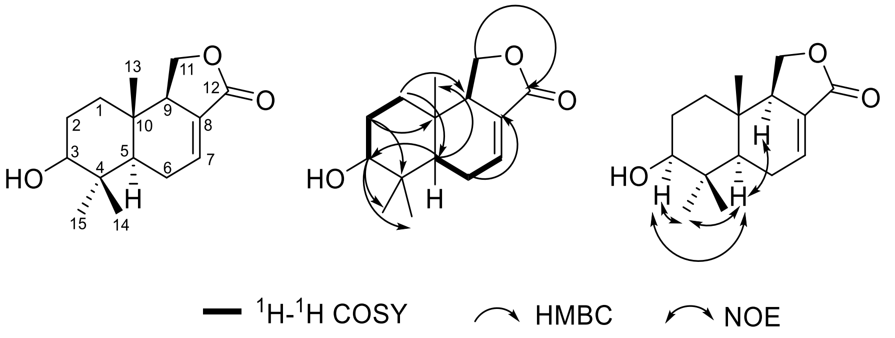

Position		δ$_H$, mult. (*J* in Hz)[a]	δ$_C$, type[b]	COSY[a]	HMBC[a]	NOE[c]
1	a	1.64, m	37.4, t	1b, 2b, 5	3, 5, 10	
	b	1.35, td (*13.86, 4.12*)		1a, 2b	2, 3, 9	
2	a	1.70, m	27.1, t		1, 3, 4, 10	
	b	1.66, m		1b, 3	3, 4, 10	
3		3.31, dd (*11.04, 4.29)*	78.8, d	2b	14, 15	5, 15
4			38.9, s			
5		1.38, dd (*11.57, 5.38*)	49.3, d	6a, 6b	3, 4, 6, 9, 10, 13, 14, 15	2b, 3, 9, 15
6	a	2.44, dq (*20.25, 3.98*)	25.0, t	5, 6b, 7	5, 7, 8, 10	
	b	2.22, m		5, 6a, 7	5	
7		6.90, dd (*7.06*, *3.50*)	136.3, d	6a, 6b, 9		
8			127.2, s			
9		2.79, m	50.9, d	7, 11a, 11b	7, 10, 11	5
10			34.3, s			
11	a	4.39, t (*9.15*)	67.2, t	9, 11b	8, 9, 12	
	b	4.05, t (*9.15*)		9, 11a	9, 10	
12			170.1, s			
13		0.81, s	13.6, q		1, 5, 9, 10	
14		0.92, s	15.0, q		3, 4, 5, 15	
15		1.05, s	28.0, q		3, 4, 5, 14	

[a] 600 MHz for ^{1}H NMR, COSY, HSQC, HMBC [b] 150 MHz for ^{13}C, DEPT, [c] 500 MHz for 1D NOE

[b] Numbers of attached protons were determined by analysis of 2D spectra and DEPT.

Table S15. Comparison of NMR spectral data of compound **5** with literature data.[16]

		Literature		Compound 5	
Pos.		δ$_H$, mult. (*J* in Hz)	δ$_C$, type	δ$_H$, mult. (*J* in Hz)	δ$_C$, type
1	a	n. d.	37.3	1.64, m	37.4, t
	b	n. d.		1.35, td (*13.86, 4.12*)	
2	a	n. d.	27.0	1.70, m	27.1, t
	b	n. d.		1.66, m	
3		3.31, dd (*10.5, 4.5*)	78.7	3.31, dd (*11.04, 4.29)*	78.8, d
4			38.8		38.9, s
5		1.38, dd (*11.5, 5.5*)	49.2	1.38, dd (*11.57, 5.38*)	49.3, d
6	a	2.20, dddd (*20.2, 11.5, 5.0, 3.5*)	24.9	2.44, dq (*20.25, 3.98*)	25.0, t
	b	2.44, ddt (20.2, 5.5, 3.5)		2.22, m	
7		6.90, dt (*3.5, 3.5*)	136.2	6.90, dd (*7.06*, *3.50*)	136.3, d
8			127.2		127.2, s
9		2.79	50.8	2.79, m	50.9, d
10			34.2		34.3, s
11		4.39, t (*9.1*)	67.0	4.39, t (*9.15*)	67.2, t
		4.05, t (*9.1*)		4.05, t (*9.15*)	
12			170.0		170.1, s
13		0.81	13.5	0.81, s	13.6, q
14		0.92	14.9	0.92, s	15.0, q
15		1.05	27.9	1.05, s	28.0, q

Table S16. NMR spectral data ($CDCl_3$, at 300 K) for compound **6**.

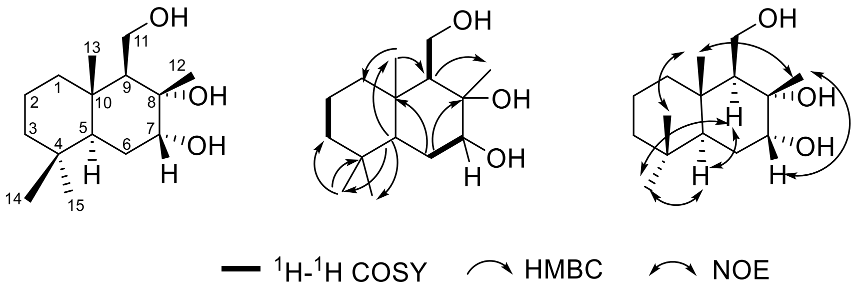

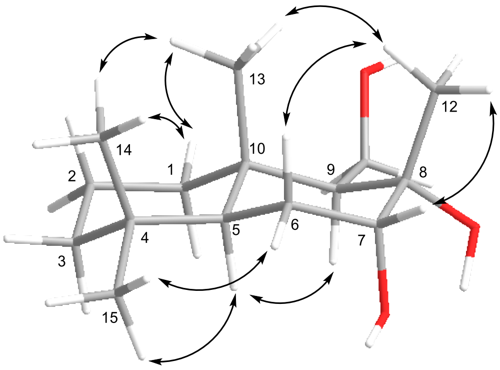

Position		δH, mult. (*J* in Hz)[a]	δC, type[b]	COSY[a]	HMBC[a]	NOE[c]
1	a	1.54, m	39.3, t			
	b	1.42, m				
2	a	1.606, m	18.6, t			
	b	1.47, m		3b		
3	a	1.41, m	41.8, t			
	b	1.22, m		2b		
4			32.7, s			
5		1.612, m	46.7, d		6, 10, 13, 14, 15	6a, 9, 15
6	a	1.92, m	25.3, t	6b, 7	5, 7, 8, 10	
	b	1.52, m		6a, 7		
7		3.76, m	72.9, d	6	5	
8			76.0, s			
9		1.96, dd (*12.39*, *5.11*)	50.1, d	11	8, 10, 11, 12, 13	2a, 5, 13, 15
10			36.4, s			
11	a	4.04, m	60.1, t	9	8, 9	
	b	3.97, m		9	8, 9	
12		1.31, s	22.8, s		7, 8, 9	6b, 7, 13
13		0.85, s	15.5, s		1, 5, 9, 10	12
14		0.80, s	21.6, s		3, 4, 5, 15	13, 15
15		0.89, s	33.2, s		3, 4, 5, 14	3a, 5, 6a, 14

[a] 600 MHz for ^1H NMR, COSY, HSQC, HMBC [b] 150 MHz for ^{13}C, DEPT, [c] 500 MHz for NOE

[b] numbers of attached protons were determined by analysis of 2D spectra and DEPT.

Table S17. Comparison of NMR spectral data of compound **6** with literature data.[17]

		**Literature**		**Compound 6**	
**Position**		**$δ_H$, mult. (*J* in Hz)**	**$δ_C$, type**	**$δ_H$, mult. (*J* in Hz)[a]**	**$δ_C$, type[b]**
1	a	1.12, td (*12.5, 12.9, 3.7*)	39.62	1.54, m	39.3, t
	b	1.67, dtd (*12.5, 3.5, 3.2, 1.3*)		1.42, m	
2	a	1.58, qt (*14.0, 13.6, 13.6, 3.5*)	18.59	1.606, m	18.6, t
	b	1.46, dquint (*14.0, 3.5, 3.5, 3.2, 3.2*)		1.47, m	
3		1.39, dtd (*13.6, 3.5, 3.2, 1.3*)	40.79	1.41, m	41.8, t
		1.22, td (*13.6, 13.6, 3.5*)		1.22, m	
4			32.70		32.7, s
5		1.57, m (*11.0*)	45.08	1.612, m	46.7, d
6	a	1.86, m	25.70	1.92, m	25.3, t
	b	1.53, m (*13.2*)		1.52, m	
7		3.67, (*3.6*)	24.03	3.76, m	72.9, d
8		n.a.	75.28		76.0, s
9		n.a.	54.41	1.96, dd (*12.39*, *5.11*)	50.1, d
10			36.99		36.4, s
11	a	3.95	60.76	4.04, m	60.1, t
	b	3.99		3.97, m	
12		1.33, s	23.07	1.31, s	22.8, s
13		0.90, s	21.60	0.85, s	15.5, s
14		0.885, s	32.31	0.80, s	21.6, s
15		0.79, s	15.64	0.89, s	33.2, s

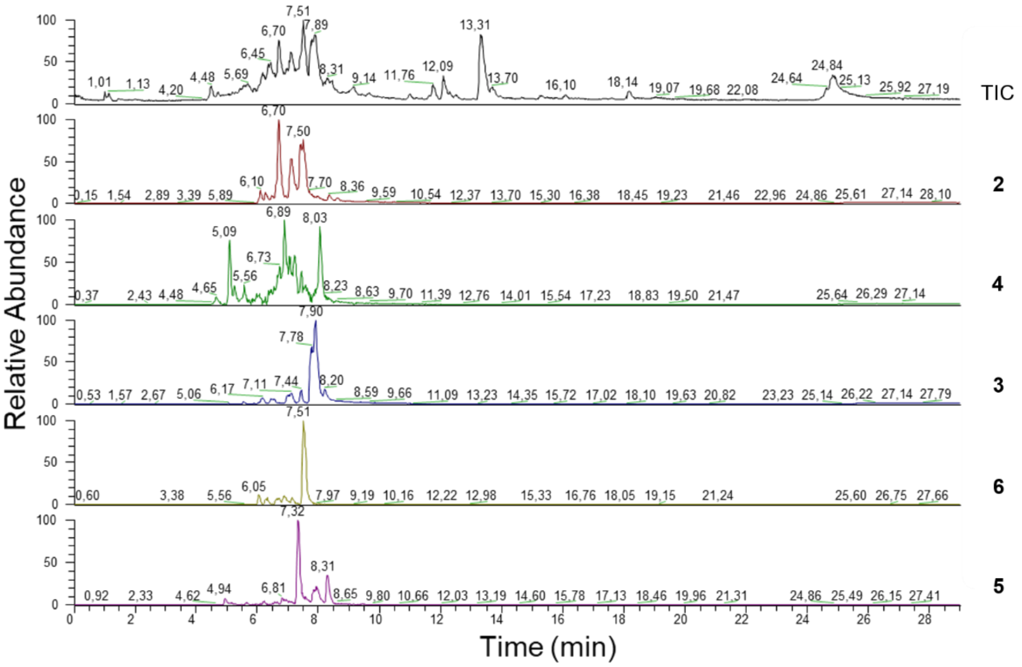

Figure S12. Extracted ion chromatograms of drimenol derivatives **2-6** detected in extracts of *Termitomyces* sp. T153.

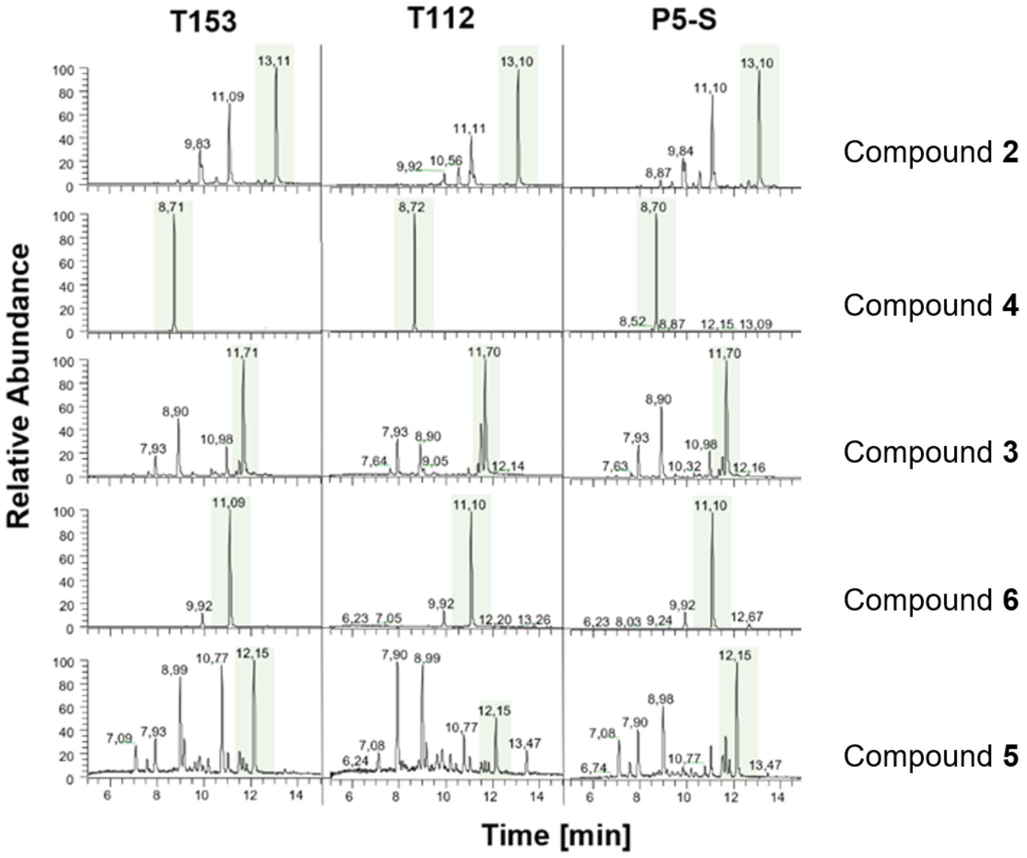

Figure S13. Extracted ion chromatograms of drimenol derivatives **2-6** of extracts obtained from *Termitomyces* sp. T153, T112 and P5-S after growth on PDA.

Table S18. Comparison of ^{1}H-NMR chemical shift values of isolated and synthesized compounds **1**, **2**, **3**, **12**, **18** and **19** and reported literature values for **1** ([a] 400 MHz in $CDCl_3$, [b] 300 MHz in CD_3OD).

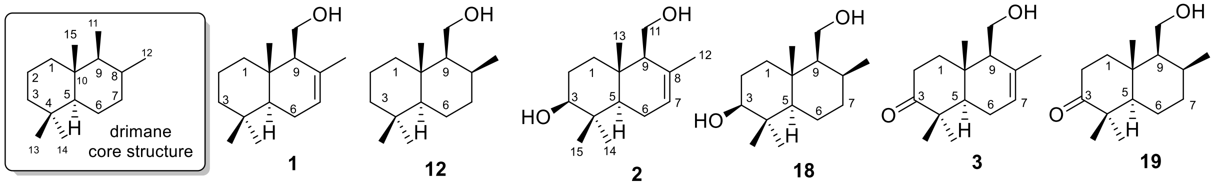

Position	1[a18]	Literature report: 1[a,4]	12[a,19,20]	2[a]	18[b]	3[a]	19[b]
	δ$_H$, mult. (*J* in Hz)						
**1**	1.27 - 1.08, m	1.26 - 1.01, m	1.47 - 1.36, m<br>1.10 - 1.02, m	1.29, m<br>1.28, td *(13.5, 4.0)*	1.38 - 1.26, m<br>1.68 - 1.47, m	2.74, td (*15.3, 6.0*)<br>2.28, m	2.65 - 2.36, m<br>1.78, d (*9.0*)
**2**	1.27 - 1.08, m	1.26 - 1.01, m	1.65 - 1.47, m	1.66-1.62, m		2.29, m<br>1.56, td (*12.1, 4.4*)	2.22, td (*4.9, 2.5*)<br>1.63, m
**3**	1.66 - 1.40, m	1.63 - 1.39, m		3.19, dd (*11.3, 4.6*)	3.16, dd (*10.6, 5.8*)	-	-
**4**	-	-	-	-	-	-	-
**5**	1.66 - 1.40, m	1.63 - 1.39, m	1.65 - 1.47, m	1.19, q (*5.5*)	1.17, td (*12.7, 5.1*)	1.61, dd (*11.9, 3.8*)	
**6**	2.08 - 1.95, m	2.05 - 1.84, m	1.75 - 1.66, m<br>1.65 - 1.47, m	2.05, dt (*13.5, 3.4*)<br>1.98, m	1.86 - 1.69, m<br>1.68 - 1.47, m	2.08, m<br>1.95, m	1.58 – 1.40, m<br>1.63, m
**7**	5.69 - 5.47, m	5.54, br d (*3.9*)		5.46, m		5.58, m	
**8**	-	-	1.65 - 1.47, m	-	1.68 - 1.47, m	-	2.03, ddd (*13.3, 7.4, 4.5*)
**9**	1.95 - 1.84, m	2.05 - 1.84, m	2.17, dtd (*9.8, 4.9, 2.3*)	1.81, m	2.17, m	1.92, m	2.22, td (*4.9, 2.5*)
**10**	-	-	-	-	-	-	-
**11**	3.95 - 3.67, m	3.86, d (*10.7*)<br>3.74, d (*8.3*)	3.88, dt (*8.3, 3.8*)<br>3.62, td (*10.2, 3.5*)	3.80, dd (*11.1, 3.2*)<br>3.57, dd (*11.1, 6.5*)	3.78, dd (*10.8, 3.5*)<br>3.53, t (*10.3*)	3.88, dd (*11.2, 3.8*)<br>3.81, dd (*11.2, 3.8*)	3.79, dd (*10.9, 4.4*)<br>3.59, dd (*10.9, 9.7*)
**12**	1.81, s	1.79, s	0.99, d (*7.6*)	1.75, s	0.98, d (*7.5*)	1.79, s	1.02, d (*7.5*)
**13**<br>**14**<br>**15**	0.88, s<br>0.89, s<br>0.91, s	0.86, s<br>0.87 s<br>0.89, s	0.84, s<br>0.89, s<br>0.89, s	0.96, s<br>0.85, s<br>0.89, s	0.98, s<br>0.89 s,<br>0.79, s	1.10, s<br>1.104, s<br>1.06, s	1.10, s<br>1.06, s<br>0.98, s

Table S19. Comparison of ^{13}C chemical shift values of isolated and synthesized compounds **1**, **2**, **3**, **12**, **18** and **19** and reported literature values for **1** ([a] 400 MHz in $CDCl_3$, [b] 300 MHz in CD_3OD).

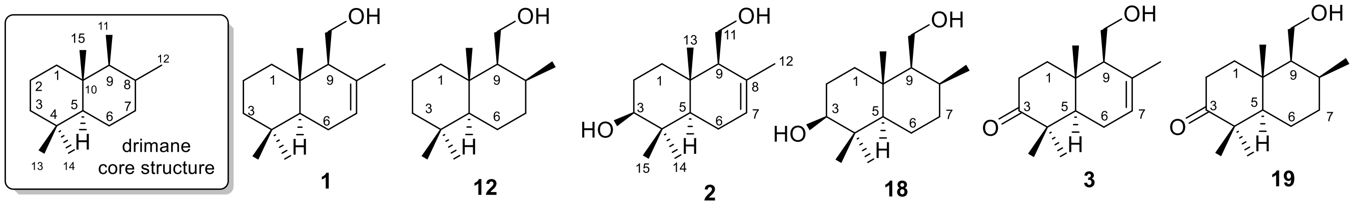

Position	1[a]	Literature report 1[a4,21]	12[a,21]	2	18[b22]	3	19[b,23]
**1**	39.9	39.9	39.9	39.0	38.0	34.7, t	38.6
**2**	18.7	18.8	18.4	28.1	27.3	38.6, t	36.3
**3**	42.1	42.1	41.9	79.7	78.0	216.7, s	218.8
**4**	33.3	33.4	33.2	39.8	38.6	47.6, s	47.2
**5**	49.9	49.9	56.5	51.0	55.3	51.4, d	54.4
**6**	23.5	23.6	17.5	24.4	16.9	24.0, t	15.8
**7**	124.1	124.2	34.4	123.8	34.3	123.9, d	33.5
**8**	132.8	132.9	28.5	135.0	28.0	133.1, s	27.9
**9**	57.3	57.3	55.7	58.3	55.7	56.2, d	55.2
**10**	36.0	36.1	37.5	36.9	36.8	36.0, s	36.3
**11**	60.9	60.9	61.0	61.2	59.1	60.8, t	59.2
**12**	22.0	22.1	15.6	22.1,	26.3,	21.9, q	25.7
**13**	32.9	32.9	33.5	28.7	16.1,	14.7, q	20.1,
**14**	21.9	21.9	21.6	15.9	14.7	22.5, q	18.1,
**15**	14.9	14.9	17.0	14.9	14.4	25.4, q	13.9

6. Genome Mining of Putative Drimenol Synthases

Table S20. List of *Termitomyces* strains used for genome mining.

Strain	Genus	Species	NCBI Accession
J132	*Termitomyces*	sp.	GCA_001263195.1
T112	*Termitomyces*	sp.	GCA_018296085.1
T153	*Termitomyces*	sp.	GCA_018296165.1
JCM 13351	*Termitomyces*	sp.	GCA_001972325
MG145	*Termitomyces*	sp.	GCA_003313055
MG16	*Termitomyces*	sp.	GCA_003313075
	Termitomyces	*heimii*	GCA_003313675
MG148	*Termitomyces*	sp.	GCA_003313785

Table S21. Identified terpenes from *Termitomyces* sp. mushrooms and the identified or putatively assigned enzymes responsible for biosynthesis of the respective compounds along with their proposed cyclization intermediates. Terpenes with unusual biosynthesis are marked in orange.

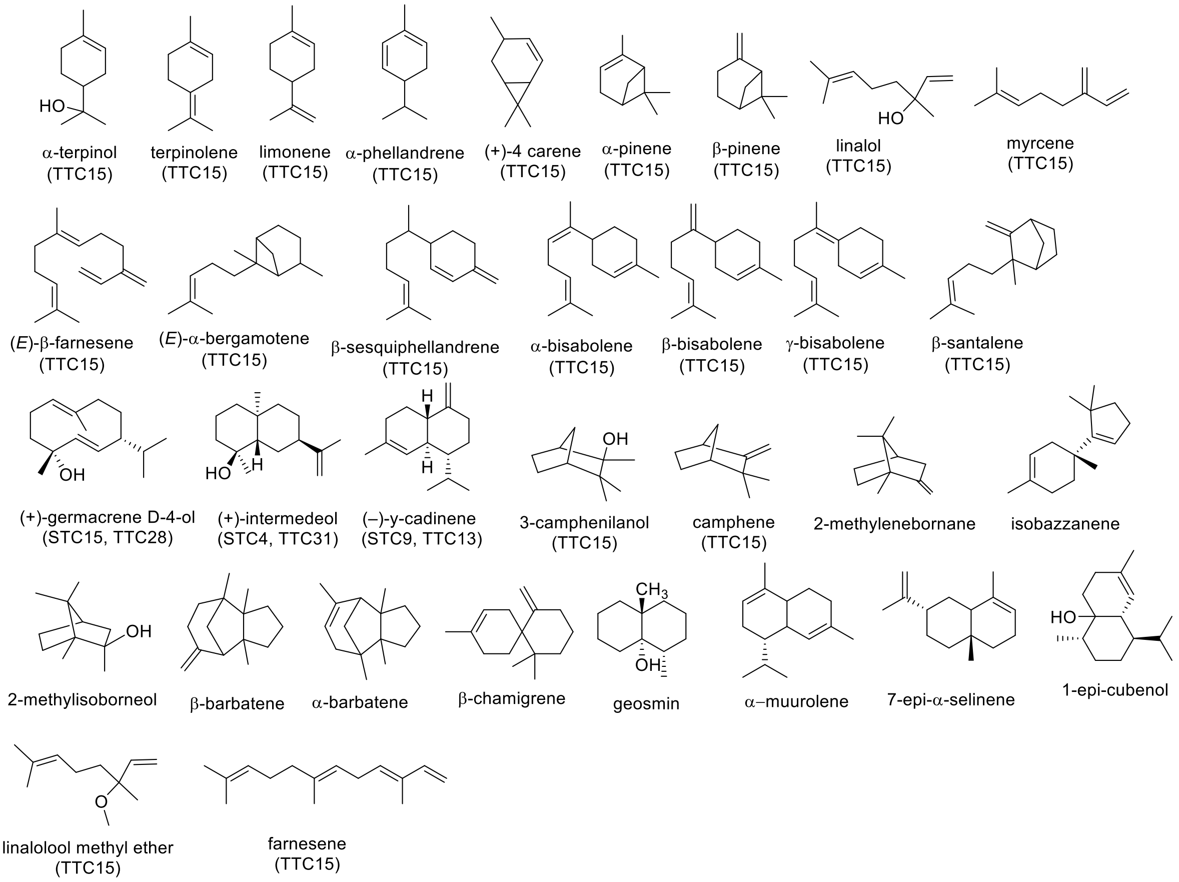

Compound	Biosynthetic Classification	Cyclization Intermediate	Identified/ *Putative* Enzymes	Ref.
African-1-ene	Clade III	Humulyl Cation	*TTC2-10*	24
α-Amorphene	Clade II	Germacradienyl Cation	*TTC1*	25
α-Barbatene	Clade IV	Bisabolyl Cation	*TTC14,16-20*	26
Amorpha,4-11-diene	Clade IV	Bisabolyl Cation	*TTC14,16-20*	27
β-Barbatene	Clade IV	Bisabolyl Cation	*TTC14,16-20*	26
Brasila-5(10),6-diene<br>Brasila-1,10-diene<br>Brasila-5,10-diene<br>Brasila-1(6),5(10)-diene	Clade III	Humulyl Cation	*TTC2-10*	28
5-Oxocamphor	Monoterpene			
α-Copaene<br>α-Cubebene	Putative Clade II	Germacradienyl Cation	*TTC1*	2926
β-Cubebene	Clade II	Germacradienyl Cation	*TTC1*	30
Drimenol	No Clade	not canonical sesquiterpene cyclization		31
Geranylacetone	linear			
Intermedeol	Clade V	Germacradienyl Cation	**TTC31**	3233
Linalool	Monoterpene		**TTC15**	32
Linalool Oxide	Monoterpene			
Isolongifolene	Unknown			34
α-Neocallitropsene	Clade IV	Bisabolyl Cation	*TTC14,16-20*	35
α-Pinene	Monoterpene		**TTC15**	32

Figure S14. Putative drimenol synthase (DS1) was identified by BLAST search of the AstC protein sequence (Accession Gene ID AORIB40_05908) against the respective *Termitomyces* predicted protein sequences of eight *Termitomyces* genomes. All identified sequences were aligned according to their classification by MUSCLE in Geneious Prime (2020.2.5) Putative active motifs are highlighted in orange boxes.

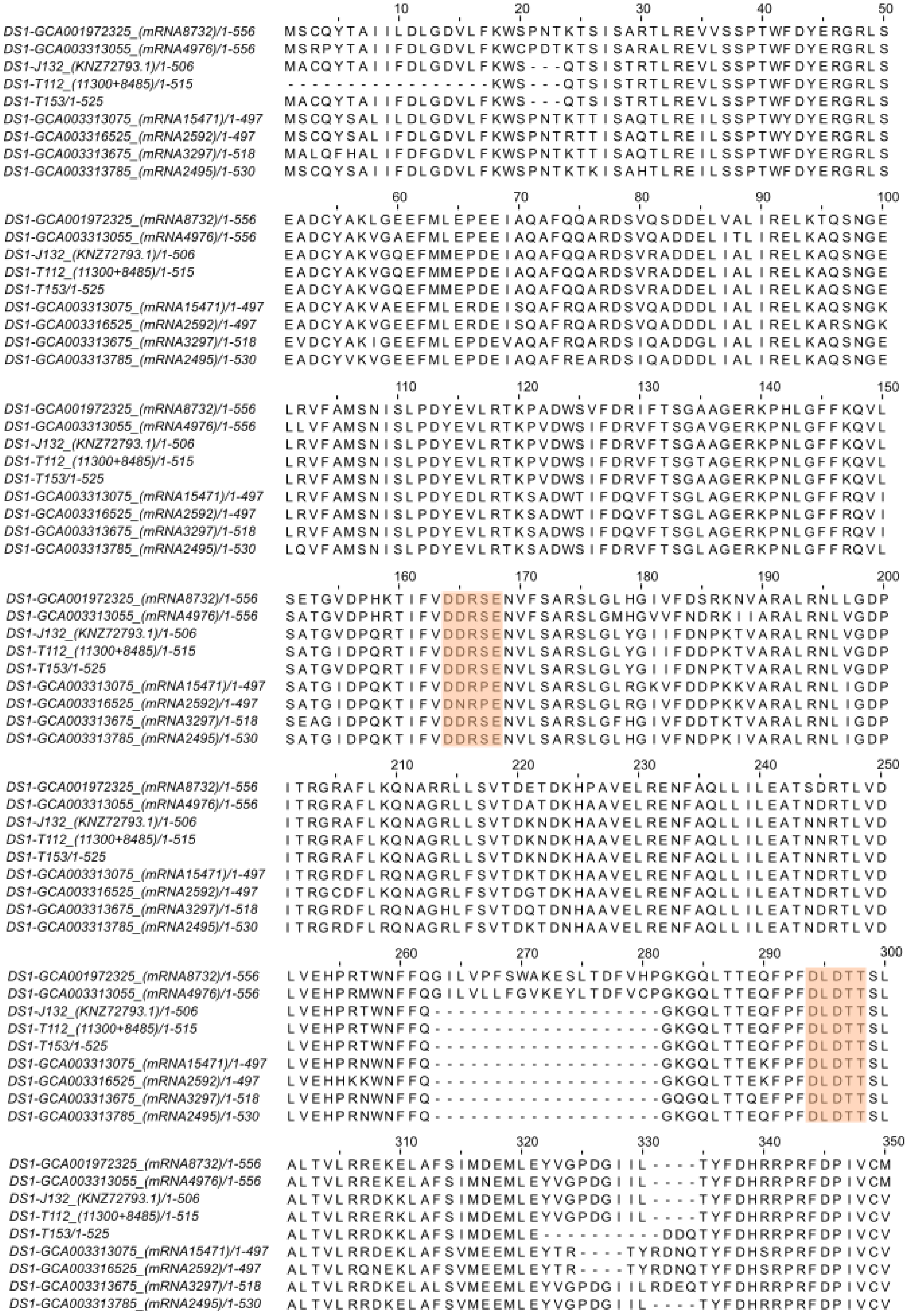

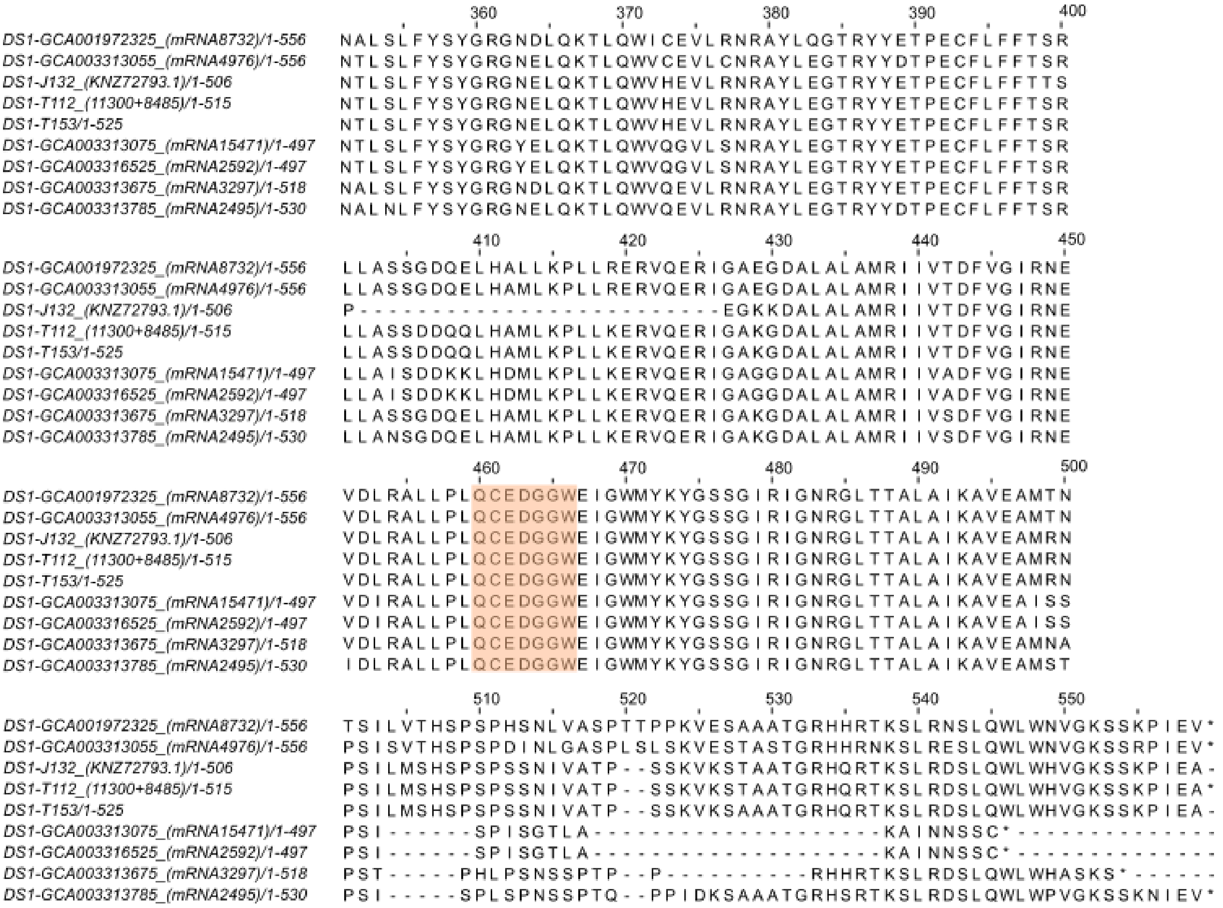
360 370 380 390 400
DS1-GCA001972325_(mRNA8732)/1-556 NALSLFYSYGRGNDLQKTLQWICEVLRNRAYLQGTRYYETPECFLFFTSR
DS1-GCA003313055_(mRNA4976)/1-556 NTLSLFYSYGRGNELQKTLQWVCEVLCNRAYLEGTRYYDTPECFLFFTSR
DS1-J132_(KNZ72793.1)/1-506 NTLSLFYSYGRGNELQKTLQWVHEVLRNRAYLEGTRYYETPECFLFFTTS
DS1-T112_(11300+8485)/1-515 NTLSLFYSYGRGNELQKTLQWVHEVLRNRAYLEGTRYYETPECFLFFTSR
DS1-T153/1-525 NTLSLFYSYGRGNELQKTLQWVHEVLRNRAYLEGTRYYETPECFLFFTSR
DS1-GCA003313075_(mRNA15471)/1-497 NTLSLFYSYGRGYELQKTLQWVQGVLSNRAYLEGTRYYETPECFLFFTSR
DS1-GCA003316525_(mRNA2592)/1-497 NTLSLFYSYGRGYELQKTLQWVQGVLSNRAYLEGTRYYETPECFLFFTSR
DS1-GCA003313675_(mRNA3297)/1-518 NALSLFYSYGRGNDLQKTLQWVQEVLRNRAYLEGTRYYETPECFLFFTSR
DS1-GCA003313785_(mRNA2495)/1-530 NALNLFYSYGRGNELQKTLQWVQEVLRNRAYLEGTRYYDTPECFLFFTSR
410 420 430 440 450
DS1-GCA001972325_(mRNA8732)/1-556 LLASSGDQELHALLKPLLRERVQERIGAEGDALALAMRIIVTDFVGIRNE
DS1-GCA003313055_(mRNA4976)/1-556 LLASSGDQELHAMLKPLLRERVQERIGAEGDALALAMRIIVTDFVGIRNE
DS1-J132_(KNZ72793.1)/1-506 P-------------------------EGKKDALALAMRIIVTDFVGIRNE
DS1-T112_(11300+8485)/1-515 LLASSDDQQLHAMLKPLLKERVQERIGAKGDALALAMRIIVTDFVGIRNE
DS1-T153/1-525 LLASSDDQQLHAMLKPLLKERVQERIGAKGDALALAMRIIVTDFVGIRNE
DS1-GCA003313075_(mRNA15471)/1-497 LLAISDDKKLHDMLKPLLKERVQERIGAGGDALALAMRIIVADFVGIRNE
DS1-GCA003316525_(mRNA2592)/1-497 LLAISDDKKLHDMLKPLLKERVQERIGAGGDALALAMRIIVADFVGIRNE
DS1-GCA003313675_(mRNA3297)/1-518 LLASSGDQELHAMLKPLLKERVQERIGAKGDALALAMRIIVSDFVGIRNE
DS1-GCA003313785_(mRNA2495)/1-530 LLANSGDQELHAMLKPLLKERVQERIGAKGDALALAMRIIVSDFVGIRNE
460 470 480 490 500
DS1-GCA001972325_(mRNA8732)/1-556 VDLRALLPLQCEDGGWEIGWMYKYGSSGIRIGNRGLTTALAIKAVEAMTN
DS1-GCA003313055_(mRNA4976)/1-556 VDLRALLPLQCEDGGWEIGWMYKYGSSGIRIGNRGLTTALAIKAVEAMTN
DS1-J132_(KNZ72793.1)/1-506 VDLRALLPLQCEDGGWEIGWMYKYGSSGIRIGNRGLTTALAIKAVEAMRN
DS1-T112_(11300+8485)/1-515 VDLRALLPLQCEDGGWEIGWMYKYGSSGIRIGNRGLTTALAIKAVEAMRN
DS1-T153/1-525 VDLRALLPLQCEDGGWEIGWMYKYGSSGIRIGNRGLTTALAIKAVEAMRN
DS1-GCA003313075_(mRNA15471)/1-497 VDIRALLPLQCEDGGWEIGWMYKYGSSGIRIGNRGLTTALAIKAVEAISS
DS1-GCA003316525_(mRNA2592)/1-497 VDIRALLPLQCEDGGWEIGWMYKYGSSGIRIGNRGLTTALAIKAVEAISS
DS1-GCA003313675_(mRNA3297)/1-518 VDLRALLPLQCEDGGWEIGWMYKYGSSGIRIGNRGLTTALAIKAVEAMNA
DS1-GCA003313785_(mRNA2495)/1-530 IDLRALLPLQCEDGGWEIGWMYKYGSSGIRIGNRGLTTALAIKAVEAMST
510 520 530 540 550
DS1-GCA001972325_(mRNA8732)/1-556 TSILVTHSPSPHSNLVASPTTPPKVESAAATGRHHRTKSLRNSLQWLWNVGKSSKPIEV*
DS1-GCA003313055_(mRNA4976)/1-556 PSISVTHSPSPDINLGASPLSLSKVESTASTGRHHRNKSLRESLQWLWNVGKSSRPIEV*
DS1-J132_(KNZ72793.1)/1-506 PSILMSHSPSPSSNIVATP--SSKVKSTAATGRHQRTKSLRDSLQWLWHVGKSSKPIEA-
DS1-T112_(11300+8485)/1-515 PSILMSHSPSPSSNIVATP--SSKVKSTAATGRHQRTKSLRDSLQWLWHVGKSSKPIEA*
DS1-T153/1-525 PSILMSHSPSPSSNIVATP--SSKVKSAAATGRHQRTKSLRDSLQWLWHVGKSSKPIEA-
DS1-GCA003313075_(mRNA15471)/1-497 PSI------SPISGTLA--------------------KAINNSSC*--------------
DS1-GCA003316525_(mRNA2592)/1-497 PSI------SPISGTLA--------------------KAINNSSC*--------------
DS1-GCA003313675_(mRNA3297)/1-518 PST-----PHLPSNSSPTP--P----------RHHRTKSLRDSLQWLWHASKS*------
DS1-GCA003313785_(mRNA2495)/1-530 PSI-----SPLSPNSSPTQ--PPIDKSAAATGRHSRTKSLRDSLQWLWPVGKSSKNIEV*

Figure S15. Identified DS2 sequences were aligned according to their classification by MUSCLE in Geneious Prime (2020.2.5). Putative active motifs are highlighted in orange boxes.

```
                                          10        20        30        40        50
DS2-GCA003313055_(mRNA7931)/1-494   MTLSNIHHTSSDSRLYSTIIFDLGDVLFTWSLSIPNSPLGPKILRKILRS
DS2-J132_(KNZ77741.1)/1-550         MTPTDVHNTSSHSNLYTAIIFDLGDVLFTWSLS-SNPPLPEKLLRRILSC
DS2-T112_(mRNA2941)/1-551           MTPTDVHNTSSHSNLYTAIIFDLGDVLFTWSLS-SNPPLPEKLLRRILSC
DS2-T153/1-550                      MTPTDVHNTSSHSNLYTAIIFDLGDVLFTWSLS-SNPPLPEKLLRRILSC

                                          60        70        80        90       100
DS2-GCA003313055_(mRNA7931)/1-494   SHWFEYEKGNLDEDEVYLLIGKEISVDPAAIKDASKAARDSLRSNKEMLE
DS2-J132_(KNZ77741.1)/1-550         SHWFEYEKGNINEAEVYSRVAKDFLVDPAAVKDTLQVAQDSLQSNTKMLE
DS2-T112_(mRNA2941)/1-551           SHWFEYEKGNINEAEVYSRVAKDFLVDPAAVKDTLQVAQDSLQSNTKMLE
DS2-T153/1-550                      SHWFEYEKGNINEAEVYSRVAKDFLVDPAAVKDTLQVAQDSLQSNTKMLE

                                         110       120       130       140       150
DS2-GCA003313055_(mRNA7931)/1-494   VIQELKEAGLSVFAMSNISAPDWEVLRTKATPTEWALFDHIFTSASARQC
DS2-J132_(KNZ77741.1)/1-550         VIQELKEAGLMIYAMSNISAPSWEILERKATPSHWALFDHVFTSASAHQR
DS2-T112_(mRNA2941)/1-551           VIQELKEAGLMIYAMSNISAPSWEILERKATPSQWALFDHVFTSASAHQR
DS2-T153/1-550                      VIQELKEAGLMIYAMSNISAPSWEILERKATPSQWALFDHVFTSASAHQR

                                         160       170       180       190       200
DS2-GCA003313055_(mRNA7931)/1-494   KPNTGFFKHVIEKTGIDPSRTIFVDDKLENVLTARSFGMHGIIFDDQSKV
DS2-J132_(KNZ77741.1)/1-550         KPNLGFFRHVIERTGIDPSRTILVDDKLENVLTARSFGMHGIIFDNESKV
DS2-T112_(mRNA2941)/1-551           KPNLGFFRHVIERTGIDPSRTILVDDKLENVLTARSFGMHGIIFDNESKV
DS2-T153/1-550                      KPNLGFFRHVIERTGIDPSRTILVDDKLENVLTARSFGMHGIIFDNESKV

                                         210       220       230       240       250
DS2-GCA003313055_(mRNA7931)/1-494   IKSLRNLCYDPILRGKKFLASH-QSLNSVTSDNIALLENFSRLIILLAAG
DS2-J132_(KNZ77741.1)/1-550         IKDLKNLCYDPVLRGKRFLTSHKKNLKTVTSNGIEFMDDYSQLIILLATG
DS2-T112_(mRNA2941)/1-551           IKDLKNLCYDPVLRGKRFLTSHKKNLKTVTSNGIEFMDDYSQLIILLATG
DS2-T153/1-550                      IKDLKNLCYDPVLRGKRFLTSHKKNLKTVTSNGIEFMDDYSQLIILLATG

                                         260       270       280       290       300
DS2-GCA003313055_(mRNA7931)/1-494   NGSLQVEHMVKSPGHYNVFQDSGVLTDEVYPNDLDMISIYHTVYEHVDMD
DS2-J132_(KNZ77741.1)/1-550         DDSL-VDY-VKSPGQFNVFPDGTLFTTEVYPNDLDTTAIGLTVTDHVDVG
DS2-T112_(mRNA2941)/1-551           DDSL-VDY-VKSPGQFNVFPDGTLFTTEVYPNDLDTTAIGLTVTDHVDVG
DS2-T153/1-550                      DDSL-VDY-VKSPGQFNVFPDGTLFTTEVYPNDLDTTAIGLTVTDHVDVG

                                         310       320       330       340       350
DS2-GCA003313055_(mRNA7931)/1-494   TKHGFMDKVIGYQDPNGIVQVYFDHSRPRIDPIVCINVLNLFYKNGRGHE
DS2-J132_(KNZ77741.1)/1-550         TKHKIMDEMLEYRDSDAIIQVYFDHSRPRIDPVICINVLNLFYENGRGHE
DS2-T112_(mRNA2941)/1-551           TKHKIMDEMLEYRDSDAIIQVYFDHSRPRIDPVICINVLNLFYENGRGHE
DS2-T153/1-550                      TKHKIMDEMLEYRDSDAIIQVYFDHSRPRIDPVICINVLNLFYENGRGHE

                                         360       370       380       390       400
DS2-GCA003313055_(mRNA7931)/1-494   LSGTLDWVEQVLVNRAYISGTYYYVSADHYLFFLSRLLQTSAEVRQRLGT
DS2-J132_(KNZ77741.1)/1-550         LPETLDWVEQVLTHRAYISGTTYYIGADVFLFFLSRLLQTSAEVRRRLGS
DS2-T112_(mRNA2941)/1-551           LPETLDWVEQVLTHRAYISGTTYYIGADVFLFFLSRLLQTSAEVRRRLGS
DS2-T153/1-550                      LPETLDWVEQVLTHRAYISGTTYYIGADVFLFFLSRLLQTSAEVRRRLGS

                                         410       420       430       440       450
DS2-GCA003313055_(mRNA7931)/1-494   VFKERVVERFGAEGDSLSLAARIIAATVVDLVDERDLMTLFSMQCEDGSW
DS2-J132_(KNZ77741.1)/1-550         IFKERVIERFGVEGDSLSLSARIIAATVAGVIDERALKNLLSMQCEDGSW
DS2-T112_(mRNA2941)/1-551           IFKERVIERFGVEGDSLSLSARIIAATVAGVIDERALKNLLSMQCEDGSW
DS2-T153/1-550                      IFKERVIERFGVEGDSLSLSARIIAATVAGVIDERALKNLLSMQCEDGSW

                                         460       470       480       490
DS2-GCA003313055_(mRNA7931)/1-494   DDSWFWRYGLSRILVKNDGVTTALAICSIEQVQLLR--------------
DS2-J132_(KNZ77741.1)/1-550         DNSWFWKWGMSSIMAKNDGVTTALAIWAIEEVQSLRKEQSETNGCTQNAL
DS2-T112_(mRNA2941)/1-551           DNSWFWKWGMSSIMAKNDGVTTALAIWPIEEVQSLRKEQSETNGCTQNAL
DS2-T153/1-550                      DNSWFWKWGMSSIMAKNDGVTTALAIWPIEEVQSLRKEQSETNGCTQNAL

                                  500        510       520       530       540       550
DS2-GCA003313055_(mRNA7931)/1-494   --------------RGRLNPPA*------------------------------
DS2-J132_(KNZ77741.1)/1-550         PRNQGGIGHIQSWLDRGTVRGPKIPQITEEWEYADCAWEMMESITTTPKLYLR-
DS2-T112_(mRNA2941)/1-551           PRNQGGIGHIQSWLDRGTVRGPKIPQITEEWEYADCAWEMMESITTTPKLYLR*
DS2-T153/1-550                      PRNQGGIGHIQSWLDRGTVRGPKIPQITEEWEYADCAWEMMESITTTPKLYLR-
```

Figure S16. Alignment of DS3 protein sequences identified in six *Termitomyces* strains. Putative active motifs are highlighted in orange boxes.

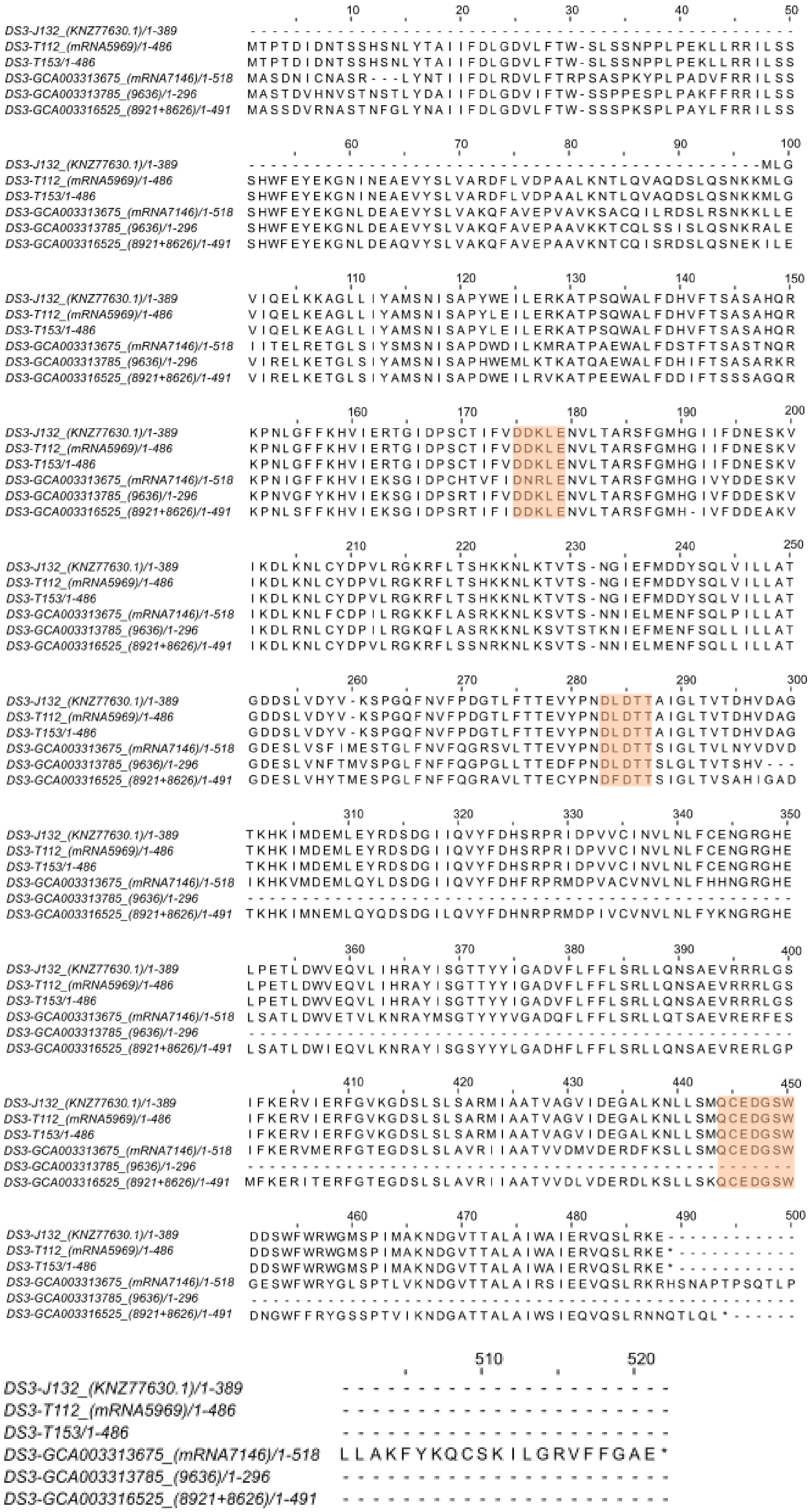

Figure S17. Alignment of DS1-3 protein sequences of different lengths. Conserved motifs are highlighted in orange boxes.

```
                  10        20        30        40        50
DS1-O   -----------MACQYTAIIFDLGDVLFKWSQTS---ISTRTLREVLSSP
DS2-O   MTPTDVHNTSSHSNLYTAIIFDLGDVLFTWSLSSNPPLPEKLLRRILSCS
DS1-S1  -----------MACQYTAIIFDLGDVLFKWSQTS---ISTRTLREVLSSP
DS2-S1  MTPTDVHNTSSHSNLYTAIIFDLGDVLFTWSLSSNPPLPEKLLRRILSCS
DS3-O   MTPTDIDNTSSHSNLYTAIIFDLGDVLFTWSLSSNPPLPEKLLRRILSSS
DS1-S2  --------------------------------------------------
DS2-S2  --------------------------------------------------
DS3-S2  --------------------------------------------------

                  60        70        80        90       100
DS1-O   TWFDYERGRLSEADCYAKVGQEFMMEPDEIAQAFQQARDSVRADDELIAL
DS2-O   HWFEYEKGNINEAEVYSRVAKDFLVDPAAVKDTLQVAQDSLQSNTKMLEV
DS1-S1  TWFDYERGRLSEADCYAKVGQEFMMEPDEIAQAFQQARDSVRADDELIAL
DS2-S1  HWFEYEKGNINEAEVYSRVAKDFLVDPAAVKDTLQVAQDSLQSNTKMLEV
DS3-O   HWFEYEKGNINEAEVYSLVARDFLVDPAALKNTLQVAQDSLQSNKKMLGV
DS1-S2  ------------------------MEPDEIAQAFQQARDSVRADDELIAL
DS2-S2  ----------------------------------------------MLEV
DS3-S2  ----------------------------------------------MLGV

                 110       120       130       140       150
DS1-O   IRELKAQSNGELRVFAMSNISLPDYEVL--RTKPVDWSIFDRVFTSGAAG
DS2-O   IQELK---EAGLMIYAMSNISAPSWEILERKATPSQWALFDHVFTSASAH
DS1-S1  IRELKAQSNGELRVFAMSNISLPDYEVL--RTKPVDWSIFDRVFTSGAAG
DS2-S1  IQELK---EAGLMIYAMSNISAPSWEILERKATPSQWALFDHVFTSASAH
DS3-O   IQELK---EAGLLIYAMSNISAPYLEILERKATPSQWALFDHVFTSASAH
DS1-S2  IRELKAQSNGELRVFAMSNISLPDYEVL--RTKPVDWSIFDRVFTSGAAG
DS2-S2  IQELK---EAGLMIYAMSNISAPSWEILERKATPSQWALFDHVFTSASAH
DS3-S2  IQELK---EAGLLIYAMSNISAPYLEILERKATPSQWALFDHVFTSASAH

                 160       170       180       190       200
DS1-O   ERKPNLGFFKQVLSATGVDPQRTIFVDDRSENVLSARSLGLYGIIFDNPK
DS2-O   QRKPNLGFFRHVIERTGIDPSRTILVDDKLENVLTARSFGMHGIIFDNES
DS1-S1  ERKPNLGFFKQVLSATGVDPQRTIFVDDRSENVLSARSLGLYGIIFDNPK
DS2-S1  QRKPNLGFFRHVIERTGIDPSRTILVDDKLENVLTARSFGMHGIIFDNES
DS3-O   QRKPNLGFFKHVIERTGIDPSCTIFVDDKLENVLTARSFGMHGIIFDNES
DS1-S2  ERKPNLGFFKQVLSATGVDPQRTIFVDDRSENVLSARSLGLYGIIFDNPK
DS2-S2  QRKPNLGFFRHVIERTGIDPSRTILVDDKLENVLTARSFGMHGIIFDNES
DS3-S2  QRKPNLGFFKHVIERTGIDPSCTIFVDDKLENVLTARSFGMHGIIFDNES

                 210       220       230       240       250
DS1-O   TVARALRNLVGDPITRGRAFLKQNAGRLLSVTDKNDKHAAVELRENFAQL
DS2-O   KVIKDLKNLCYDPVLRGKRFLTSHKKNLKTVTSN-----GIEFMDDYSQL
DS1-S1  TVARALRNLVGDPITRGRAFLKQNAGRLLSVTDKNDKHAAVELRENFAQL
DS2-S1  KVIKDLKNLCYDPVLRGKRFLTSHKKNLKTVTSN-----GIEFMDDYSQL
DS3-O   KVIKDLKNLCYDPVLRGKRFLTSHKKNLKTVTSN-----GIEFMDDYSQL
DS1-S2  TVARALRNLVGDPITRGRAFLKQNAGRLLSVTDKNDKHAAVELRENFAQL
DS2-S2  KVIKDLKNLCYDPVLRGKRFLTSHKKNLKTVTSN-----GIEFMDDYSQL
DS3-S2  KVIKDLKNLCYDPVLRGKRFLTSHKKNLKTVTSN-----GIEFMDDYSQL

                 260       270       280       290       300
DS1-O   LILEATNNRTLVDLVEHPRTWNFFQGKGQLTTEQFPFDLDTTSLALTVLR
DS2-O   IILLATGDDSLVDYVKSPGQFNVFPDGTLFTTEVYPNDLDTTAIGLTVTD
DS1-S1  LILEATNNRTLVDLVEHPRTWNFFQGKGQLTTEQFPFDLDTTSLALTVLR
DS2-S1  IILLATGDDSLVDYVKSPGQFNVFPDGTLFTTEVYPNDLDTTAIGLTVTD
DS3-O   VILLATGDDSLVDYVKSPGQFNVFPDGTLFTTEVYPNDLDTTAIGLTVTD
DS1-S2  LILEATNNRTLVDLVEHPRTWNFFQGKGQLTTEQFPFDLDTTSLALTVLR
DS2-S2  IILLATGDDSLVDYVKSPGQFNVFPDGTLFTTEVYPNDLDTTAIGLTVTD
DS3-S2  VILLATGDDSLVDYVKSPGQFNVFPDGTLFTTEVYPNDLDTTAIGLTVTD

                 310       320       330       340       350
DS1-O   R-DKKLAFSIMDEMLEDD------QTYFDHRRPRFDPIVCVNTLSLFYSY
DS2-O   HVDVGTKHKIMDEMLEYRDSDAIIQVYFDHSRPRIDPVICINVLNLFYEN
DS1-S1  R-DKKLAFSIMDEMLEDD------QTYFDHRRPRFDPIVCVNTLSLFYSY
DS2-S1  HVDVGTKHKIMDEMLEYRDSDAIIQVYFDHSRPRIDPVICINVLNLFYEN
DS3-O   HVDAGTKHKIMDEMLEYRDSDGIIQVYFDHSRPRIDPVVCINVLNLFCEN
DS1-S2  R-DKKLAFSIMDEMLEDD------QTYFDHRRPRFDPIVCVNTLSLFYSY
DS2-S2  HVDVGTKHKIMDEMLEYRDSDAIIQVYFDHSRPRIDPVICINVLNLFYEN
DS3-S2  HVDAGTKHKIMDEMLEYRDSDGIIQVYFDHSRPRIDPVVCINVLNLFCEN

                 360       370       380       390       400
DS1-O   GRGNELQKTLQWVHEVLRNRAYLEGTRYYETPECFLFFTSRLLASSDDQQ
DS2-O   GRGHELPETLDWVEQVLTHRAYISGTTYYIGADVFLFFLSRLLQTSAE--
DS1-S1  GRGNELQKTLQWVHEVLRNRAYLEGTRYYETPECFLFFTSRLLASSDDQQ
DS2-S1  GRGHELPETLDWVEQVLTHRAYISGTTYYIGADVFLFFLSRLLQTSAE--
DS3-O   GRGHELPETLDWVEQVLIHRAYISGTTYYIGADVFLFFLSRLLQNSAE--
DS1-S2  GRGNELQKTLQWVHEVLRNRAYLEGTRYYETPECFLFFTSRLLASSDDQQ
DS2-S2  GRGHELPETLDWVEQVLTHRAYISGTTYYIGADVFLFFLSRLLQTSAE--
DS3-S2  GRGHELPETLDWVEQVLIHRAYISGTTYYIGADVFLFFLSRLLQNSAE--
```

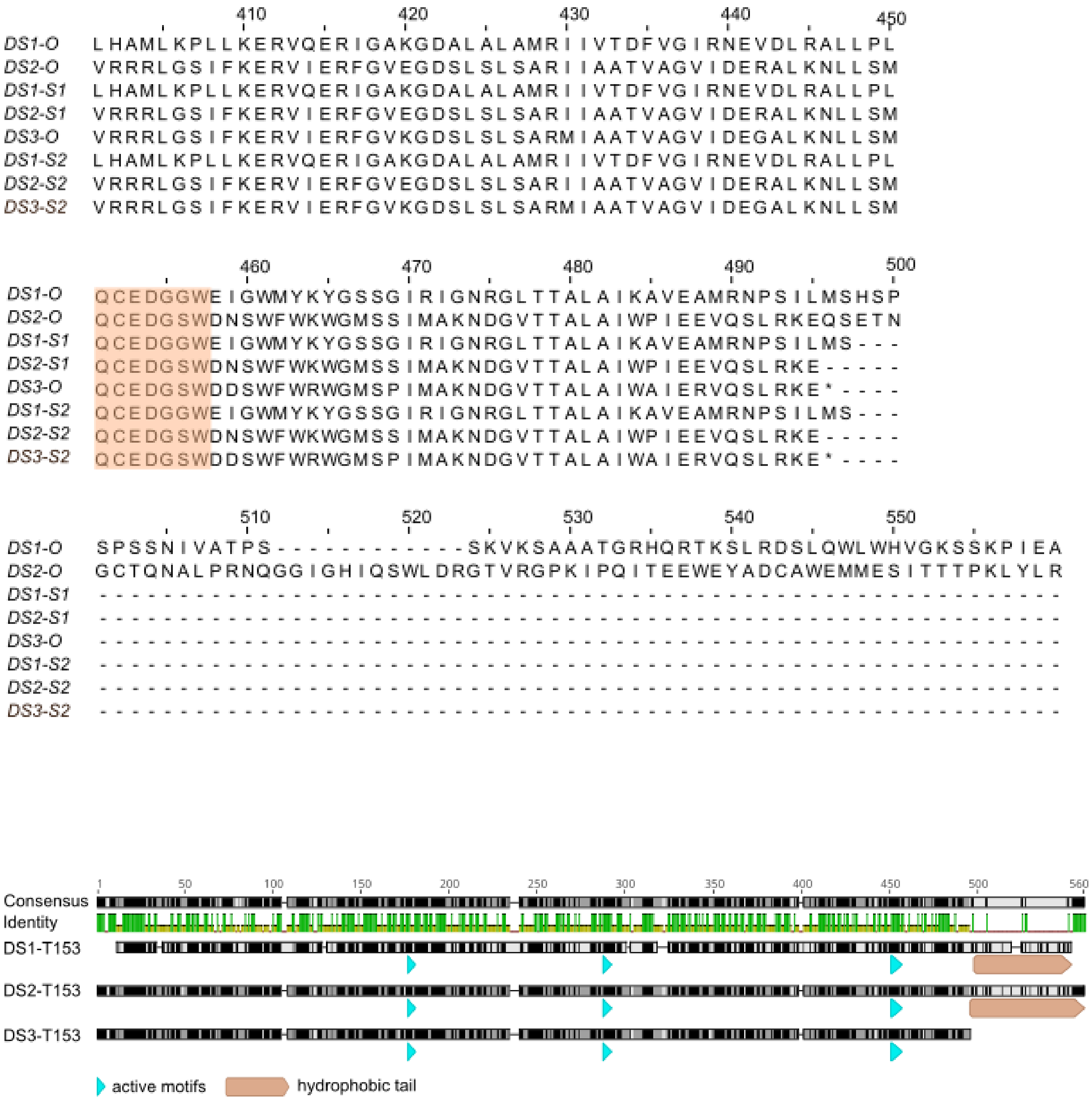

Figure S18. Protein sequence alignment of putative drimenol synthases encoded in *Termitomyces* sp. T153. Active motifs (light blue) and hydrophobic tails (light brown) are colour coded.

Table S22. Table of genes identified 30.000 bp up- and downstream of DS1 gene in *Termitomyces* sp. T153 with predicted domains of translated protein sequence.

Gene	Position	Annotation/ NCBI-BLAST of translated Protein Sequence	Identified Domains
g373	1,355,411-1,360,653	CAZyme family GH78	Trehalase, α-rhamnosidase, Autophagy related protein, Virus related protein
g374	1,361,230-1,362,205	Hypothetical Protein	DASH Complex Subunit
g375	1,362,871-1,365,878	ATP-dependent RNA Helicase	DEAD-box Helicase Domain
g376	1,366,003-1,368,847	Endomembrane Protein 70	Endomembrane Protein 70
g377	1,369,137-1,370,824	Queuine tRNA-Ribosyltransferase	Tgt Superfamily
g378	1,370,993-1,374,535	Queuine tRNA-Ribosyltransferase	Abhydrolase Superfamily, Tyrosyl-tRNA Synthetase
g379	1,374,791-1,376,979	40S Ribosomal Protein S23	40S Ribosomal Protein S23, Transglutaminase-like Superfamily, Cytokinesis Protein 3
g380	1,377,069-1,379,195	Phosphatidate Cytidylyltransferase	CTP transf Superfamily
g381	1,379,374-1,382,576	DNA Topoisomerase	DNA Topoisomerase 2-like Protein, Viral DNA-topo-I Superfamily
**g382.t1**	**1,385,044-1,386,794**	**DS1; Phosphatase**	**HAD-like Hydrolase**
g383	1,386,957-1,389,041	Threonine Dehydratase (?)	PRK09224 Superfamily
g384	1,389,196-1,391,320	PHD Zinc Finger; Histone Deacetylase Complex SU	PHD Zinc Finger
g385	1,391,591-1,396,509	Golgi CORVET Complex Core Vacuolar Protein 8	Vps8
g386	1,396,605-1,399,883	DUF3510; Golgi Complex SU2	COG2, DUF3510
g387	1,399,966-1,400,663	DUF1168; PRKR-interacting Protein 1 like	DUF1168
g388	1,401,296-1,404,580	DNA Replicative Helicase MCM Subunit	Minichromosome Maintenance Protein
g389	1,404,768-1,407,039	Arginine N-Nethyltransferase	Viral Ankyrin Protein, Longin-like Domains
g390	1,407,144-1,411,094	SNF2 Family N-Terminal Domain-Containing Protein	PHA03307, Helix Hairpin Helix Domain, HepA Superfamily Helicase
g391	1,412,064-1,413,378	Mitochondrial Carrier Protein	
g392	1,413,512-1,415,395	Hypothetical Protein	DUF1365; Exodeoxyribonuclease V SU Gamma
g393	1,415,507-1,418,652	Splicing Factor	PTZ00121; RNA Recognition Motif

Table S23. Table of genes identified 30.000 bp up- and downstream of DS2 gene in *Termitomyces* sp. T153 with predicted domains of translated protein sequences.

**Gene**	**Position**	**Annotation/ NCBI-BLAST of translated Protein Sequence**	**Identified Domains**
143	671,727-682,337	Calcium-Channel Protein	Aldose-Epimerase
g144	684,410-685,072	Hypothetical Protein	DUF1349, Concanvalin A like Superfamily
g145	685,946-686,552	Hypothetical Protein; PLAC8-domain containing Protein	PLAC8 Superfamily
g146	687,676-689,660	Putative NRPS-like	Myxochelin-NRPS, Adenylate Forming Domain, Lys2b Superfamily
g147	690,469-691,085	Hypothetical Protein	No Domains
g148	696,757-697,589	Hypothetical Protein	No Domains
g149	699,044-700,862	Putative NRPS-like	Copia type Retrotransposon Protein, AFD class Superfamily
g150	702,489-703,385	DUF636, Glutathione-dep Formaldehyde Activating Enzyme	GFA Superfamily
**g151**	**707,590-709,534**	**DS2, HAD-like Protein**	**HAD-like Superfamily**
g152	711,744-712,245	Hypothetical Protein; Retrovirus related Protein	No Domains
g153	717,483-718,695	Hypothetical Protein	No Domains
g154	731,257-731,759	Hypothetical Protein	No Domains
g155	732,060-732,290	Hypothetical Protein	No Domains

Table S24. Table of genes identified 30.000 bp up- and downstream of DS3 gene in *Termitomyces* sp. T153 with predicted domains of translated protein sequences.

Gene	Position	Annotation/ NCBI-BLAST of translated Protein Sequence	Identified Domains
g112	555,897-557,835	O-methyltransferase	C20 Methyltransferase
g113	562,058-564,135	Hypothetical protein	No Domains
g114	566,996-569,273	Laccase	Cupredoxin
g115	570,644-570,980	Hypothetical Protein	No Domains
**g116**	**577,263-579,015**	**DS3, HAD-like Protein/ Acyl-CoA Dehydrogenase Family**	**HAD-like Hydrolase**
g117	580,346-584,885	Integrase/ Recombinase	RT-like, RNase H; Recombinase
g118	589,934-590,224	Reverse Transcriptase-RNase H Integrase	Reverse Transcriptase
g119	590,750-591,373	E3 Ubiquitin-Protein Ligase	No Conserved Domains
g120	593,530-595,269	Hypothetical Protein	No Conserved Domains
g120-2	595,560-595,603	No Similarities	No Similarities
g121	598,765-599,463	ATP-dependent DNA Helicase	DEAD-like Helicase, Exonuclease V
g122	600,636-601,912	Reverse Transcriptase	Pepsin-retropepsin-like Superfamily, Reverse Transcriptase
g123	602,186-603,058	Reverse Transcriptase	RNase H-like
g124	603,404-604,516	Reverse Transcriptase	Integrase, Chromatin organization Modifier
g124-2	604,863-606,022	Hypothetical Protein	No Domains
g125	608,602-608,982	Hypothetical Protein	No Domains

Figure S19. Heatmap of expression levels (displayed as log_{10} values) of putative DS in *Termitomyces* sp. T153 in comparison to comb (of different age) and nodules of fungus growing termites (*M. natalensis* colony Mn156). DS1 was predicted as two separate transcripts but combined to one gene after reannotation. B) Table of transcript counts of RNAseq data from fresh/ old comb/ nodules (M*. natalensis* colony Mn156)[36] and a plate culture of *Termitomyces* sp. T153.[37]

A

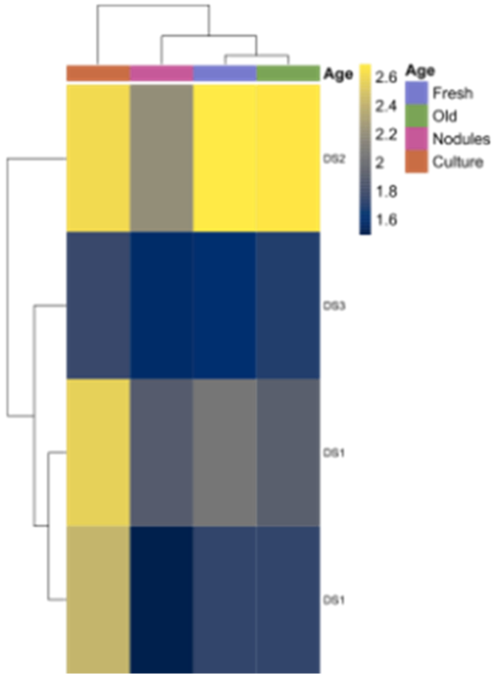

B

Gene	Fresh Comb	Nodules	Old Comb	T153 Culture
DS2	489	156	442	409
DS3	39	37	49	59
DS1-1	112	75	80	357
DS1-2	54	31	56	254

7. Cloning and Heterologous Expression

Table S25. List of identified native gene, transcript, and protein sequences of DS1-3 in *Termitomyces* strain T153.

Name	Strain	Sequence Type	Sequence
**DS1**	T153	Gene	ATGGCTTGTCAGTATACCGCTATCATCTTCGACCTCGGAGACGTGTTATTTAAATGGTC<br>TCAGACAAGTATATCAACCCGGACTCTTCGTGAAGTCCTTTCCTCACCTACATGGTTCG<br>ACTACGAGAGAGGACGTCTGTCTGAGGCTGATTGTTACGCCAAAGTTGGGCAGGAGTT<br>CATGATGGAACCTGACGAAATTGCACAGGCGTTTCAACAGGCTCGGGATTCCGTCCGG<br>GCTGACGACGAACTTATTGCACTAATTCGGGAACTCAAGGCTCAGTCCAATGGCGAAC<br>TCCGAGTCTTCGCGATGTCGAATATCTCCCTGCCTGATTATGAGGTCCTCCGCACGAA<br>GCCAGTAGATTGGTCGATATTTGACCGAGTTTTCACTTCTGGCGCTGCTGGTGAACGTA<br>AGCCAAATCTTGGCTTCTTCAAACAAGTCTTGTCGGCAACAGGTGTTGACCCTCAAAGA<br>ACAATCTTTGTGGACGATCGTTCAGAGAATGTTCTGTCTGCGCGTTCTCTTGGTCTGTA<br>CGGTATAATCTTTGATAATCCGAAGACCGTCGCGCGTGCTCTCCGCAACCTCGTTGGC<br>GACCCAATTACGAGAGGCCGCGCATTTCTCAAGCAAAACGCCGGCCGTCTATTATCCG<br>TGACAGACAAGAACGACAAGCACGCCGCTGTGGAGCTTCGAGAAAACTTTGCCCAGCT<br>TCTGATACTTGAAGCCACCAATAACCGGTATGTACTACAAGTCGTGGTTTTGAACTTGC<br>TTGAACTTAACCTCATGTAGAACCCTTGTCGACCTAGTAGAGCACCCGAGAACGTGGAA<br>TTTTTTCCAAGGTATCCCGATCCTTCTCGTGGCAAAAGAATAGTTTTACTGACTTTGTTG<br>TCAGGAAAAGGACAGCTTACAACGGAGCAATTTCCATTCGATCTCGACACCACATCTTT<br>GGCCCTGACTGTGCTCCGCCGGGACAAAAAACTCGCATTCTCTATCATGGATGAAATG<br>CTTGAGTATGTAGGTCCAGACGGGATAATCCTGGTATGCTTTTCAATCAACTTATCCTAA<br>CTCGTACTTACAGAGATGATCAGACTTATTTCGATCACCGCAGACCCCGTTTCGATCCT<br>ATCGTTTGCGTGAACACCCTCAGCCTCTTCTACTCATATGGTCGCGGTAATGAGCTTCA<br>GAAGACGCTGCAATGGGTCCATGAAGTCCTTCGTAATCGGGCATATCTTGAGGGCACC<br>AGATACTATGAAACCCCGGAATGTTTCCTCTTCTTCACAAGTCGGTTATTGGCTAGCTC<br>AGATGACCAGCAATTGCACGCTATGCTGAAGCCTCTCTTGAAGGAAAGAGTGCAAGAG<br>AGGATCGGTGCCAAAGGAGATGCCCTAGCACTGGCGATGCGCATCATCGTCACGGAC<br>TTCGTTGGGATCAGGAACGAGGTCGATCTAAGAGCACTACTTCCTCTGCAATGCGAAG<br>ACGGTGGTTGGGAGATCGGCTGGATGTACAAATATGGATCGTCGGGAATTCGGATCGG<br>AAATCGTGGCCTCACGACTGCCCTTGCCATCAAGGCTGTTGAAGCCATGAGGAATCCA<br>TCAATCTTGATGAGTCACTCGCCATCTCCCAGCTCTAACATTGTTGCGACACCATCATC<br>AAAAGTCAAATCGGCTGCCGCCACAGGACGCCACCAACGCACCAAGTCACTGAGAGAT<br>TCGCTGCAGTGGCTCTGGCATGTTGGTAAAAGCAGCAAGCCTATCGAGGCTTGA
	T153	Transcript	ATGGCTTGTCAGTATACCGCTATCATCTTCGACCTCGGAGACGTGTTATTTAAATGGTC<br>TCAGACAAGTATATCAACCCGGACTCTTCGTGAAGTCCTTTCCTCACCTACATGGTTCG<br>ACTACGAGAGAGGACGTCTGTCTGAGGCTGATTGTTACGCCAAAGTTGGGCAGGAGTT<br>CATGATGGAACCTGACGAAATTGCACAGGCGTTTCAACAGGCTCGGGATTCCGTCCGG<br>GCTGACGACGAACTTATTGCACTAATTCGGGAACTCAAGGCTCAGTCCAATGGCGAAC<br>TCCGAGTCTTCGCGATGTCGAATATCTCCCTGCCTGATTATGAGGTCCTCCGCACGAA<br>GCCAGTAGATTGGTCGATATTTGACCGAGTTTTCACTTCTGGCGCTGCTGGTGAACGTA<br>AGCCAAATCTTGGCTTCTTCAAACAAGTCTTGTCGGCAACAGGTGTTGACCCTCAAAGA<br>ACAATCTTTGTGGACGATCGTTCAGAGAATGTTCTGTCTGCGCGTTCTCTTGGTCTGTA<br>CGGTATAATCTTTGATAATCCGAAGACCGTCGCGCGTGCTCTCCGCAACCTCGTTGGC<br>GACCCAATTACGAGAGGCCGCGCATTTCTCAAGCAAAACGCCGGCCGTCTATTATCCG<br>TGACAGACAAGAACGACAAGCACGCCGCTGTGGAGCTTCGAGAAAACTTTGCCCAGCT<br>TCTGATACTTGAAGCCACCAATAACCGAACCCTTGTCGACCTAGTAGAGCACCCGAGA<br>ACGTGGAATTTTTTCCAAGGAAAAGGACAGCTTACAACGGAGCAATTTCCATTCGATCT<br>CGACACCACATCTTTGGCCCTGACTGTGCTCCGCCGGGACAAAAAACTCGCATTCTCT<br>ATCATGGATGAAATGCTTGAAGATGATCAGACTTATTTCGATCACCGCAGACCCCGTTT<br>CGATCCTATCGTTTGCGTGAACACCCTCAGCCTCTTCTACTCATATGGTCGCGGTAATG<br>AGCTTCAGAAGACGCTGCAATGGGTCCATGAAGTCCTTCGTAATCGGGCATATCTTGA<br>GGGCACCAGATACTATGAAACCCCGGAATGTTTCCTCTTCTTCACAAGTCGGTTATTGG<br>CTAGCTCAGATGACCAGCAATTGCACGCTATGCTGAAGCCTCTCTTGAAGGAAAGAGT<br>GCAAGAGAGGATCGGTGCCAAAGGAGATGCCCTAGCACTGGCGATGCGCATCATCGT<br>CACGGACTTCGTTGGGATCAGGAACGAGGTCGATCTAAGAGCACTACTTCCTCTGCAA<br>TGCGAAGACGGTGGTTGGGAGATCGGCTGGATGTACAAATATGGATCGTCGGGAATTC<br>GGATCGGAAATCGTGGCCTCACGACTGCCCTTGCCATCAAGGCTGTTGAAGCCATGAG<br>GAATCCATCAATCTTGATGAGTCACTCGCCATCTCCCAGCTCTAACATTGTTGCGACAC<br>CATCATCAAAAGTCAAATCGGCTGCCGCCACAGGACGCCACCAACGCACCAAGTCACT<br>GAGAGATTCGCTGCAGTGGCTCTGGCATGTTGGTAAAAGCAGCAAGCCTATCGAGGCT<br>TGA
	T153	Protein	MACQYTAIIFDLGDVLFKWSQTSISTRTLREVLSSPTWFDYERGRLSEADCYAKVGQEFMM<br>EPDEIAQAFQQARDSVRADDELIALIRELKAQSNGELRVFAMSNISLPDYEVLRTKPVDWSIF<br>DRVFTSGAAGERKPNLGFFKQVLSATGVDPQRTIFVDDRSENVLSARSLGLYGIIFDNPKTV<br>ARALRNLVGDPITRGRAFLKQNAGRLLSVTDKNDKHAAVELRENFAQLLILEATNNRTLVDL<br>VEHPRTWNFFQGKGQLTTEQFPFDLDTTSLALTVLRRDKKLAFSIMDEMLEDDQTYFDHRR<br>PRFDPIVCVNTLSLFYSYGRGNELQKTLQWVHEVLRNRAYLEGTRYYETPECFLFFTSRLL<br>ASSDDQQLHAMLKPLLKERVQERIGAKGDALALAMRIIVTDFVGIRNEVDLRALLPLQCEDG<br>GWEIGWMYKYGSSGIRIGNRGLTTALAIKAVEAMRNPSILMSHSPSPSSNIVATPSSKVKSA<br>AATGRHQRTKSLRDSLQWLWHVGKSSKPIEA
**DS2**	T153	Gene	ATGACTCCTACTGATGTTCACAATACTTCTTCTCACTCCAACCTCTATACTGCCATCATA<br>TTTGATCTCGGTGACGTGCTCTTCACTTGGTCTCTCTCTTCGAACCCTCCTCTTCCAGA

			AAAGCTGCTTCGTAGGATTCTGTCTTGTTCTCACTGGTTTGAGTATGAAAAGGGTAATAT<br>TAACGAAGCCGAGGTATACTCTCGTGTTGCAAAGGATTTTCTAGTCGATCCTGCTGCGG<br>TGAAGGACACCTTGCAAGTTGCGCAGGACTCTTTGCAGAGCAACACGAAAATGCTGGA<br>GGTTATTCAGGAACTCAAGGAAGCCGGCCTTATGATTTATGCTATGTCCAACATATCAG<br>CACCCTCCTGGGAGATTTTGGAAAGAAAGGCGACCCCATCACAGTGGGCCCTTTTTGA<br>TCACGTATTCACATCGTATGTTTCCTCCTTCGACTCTATTTATGTTGGACGCTGCTTAAC<br>TAGAAGTCTATTCTCAGTGCTTCTGCCCACCAACGTAAACCAAATCTCGGTTTCTTCAG<br>GCATGTTATCGAGAGGACTGGGATTGATCCTTCGCGCACCATTCTGGTCGATGACAAG<br>CTGGAGAATGTCTTGACAGCTCGATCCTTTGGAATGCATGGTATTATTTTTGACAACGA<br>GTCAAAAGTTATCAAAGACCTCAAGAATCTTTGCTACGATCCTGTTCTGCGTGGAAAGA<br>GATTTTTAACCTCTCACAAGAAGAATTTGAAGACCGTGACCTCGAATGGCATTGAATTTA<br>TGGATGTCTGTTTCTTGAGTATTACTTTCTGCAAGTGCCAGATCTAACTGATGATTTATG<br>TCAGGACTATTCTCAACTTATAATTCTCCTAGCTACAGGTGATGAGTAAGTCCTACTCCT<br>GATTTGCATGGGGTCGGAATTGTTGATAGAGTTGAAAAAGCTCTCTTGTCGATTATGTA<br>AAGTCTCCTGGACAATTCAATGTTTTCCCGGATGGCACGTTGTTCACAACTGAAGTCTA<br>TCCGTGAGTTCAATTTGACGGTTGCAGAAAAACTGAATGGGACATTGCTCATCAGAAAT<br>GACCTCGATACAACCGCTATCGGCCTTACCGTCACGGACCATGTCGACGTGGGCACAA<br>AACATAAGATTATGGATGAAATGCTCGAGTATCGGGACTCCGATGCAATCATTCAAGTA<br>TATTTCGACCATTCCCGTCCTCGCATCGGTGAAACTTCCCTCTACAAATTGAGCTGATT<br>GACGTTGTTACTGATTTACAACCTCCTATATAGATCCAGTAATCTGTATCAATGTGTTGA<br>ACCTCTTTTACGAAAATGGGAGAGGTCATGAACTTCCCGAGACACTTGACTGGGTCGA<br>ACAGGTTCTTACACATCGCGCGTACATATCAGGGACAACCTATTATATTGGGGCCGAC<br>GTATTTTTGTTCTTCCTCTCCCGCCTCCTCCAAACTTCTGCGGAAGTTCGTCGACGTCT<br>CGGATCAATCTTCAAAGAACGGGTCATAGAACGTTTTGGCGTGGAAGGGGATTCCCTT<br>TCTCTTTCTGCTCGAATCATCGCTGCAACGGTAGCAGGCGTCATAGACGAACGTGCTTT<br>AAAGAATCTTCTATCAATGCAATGCGAAGATGGTTCATGGGATAATAGTTGGTTTTGGA<br>AATGGGGAATGTCTTCAATCATGGCGAAGAACGATGGCGTAACCACTGCCCTCGCTAT<br>TTGGCCTATTGAAGAGGTCCAATCATTGCGCAAGGAGCAGTCAGAAACGAACGGATGT<br>ACTCAAAATGCTCTTCCCCGCAATCAAGGAGGCATTGGCCATATACAATCTTGGCTTGA<br>TCGGGGTACGGTTCGTGGTCCGAAGATTCCTCAGATAACGGAGGAATGGGAATATGCT<br>GACTGTGCTTGGGAGATGATGGAAAGCATAACAACTACGCCCAAGCTCTACTTAAGGT<br>AG
	T153	Transcript	ATGACTCCTACTGATGTTCACAATACTTCTTCTCACTCCAACCTCTATACTGCCATCATA<br>TTTGATCTCGGTGACGTGCTCTTCACTTGGTCTCTCTCTTCGAACCCTCCTCTTCCAGA<br>AAAGCTGCTTCGTAGGATTCTGTCTTGTTCTCACTGGTTTGAGTATGAAAAGGGTAATAT<br>TAACGAAGCCGAGGTATACTCTCGTGTTGCAAAGGATTTTCTAGTCGATCCTGCTGCGG<br>TGAAGGACACCTTGCAAGTTGCGCAGGACTCTTTGCAGAGCAACACGAAAATGCTGGA<br>GGTTATTCAGGAACTCAAGGAAGCCGGCCTTATGATTTATGCTATGTCCAACATATCAG<br>CACCCTCCTGGGAGATTTTGGAAAGAAAGGCGACCCCATCACAGTGGGCCCTTTTTGA<br>TCACGTATTCACATCTGCTTCTGCCCACCAACGTAAACCAAATCTCGGTTTCTTCAGGC<br>ATGTTATCGAGAGGACTGGGATTGATCCTTCGCGCACCATTCTGGTCGATGACAAGCT<br>GGAGAATGTCTTGACAGCTCGATCCTTTGGAATGCATGGTATTATTTTTGACAACGAGT<br>CAAAAGTTATCAAAGACCTCAAGAATCTTTGCTACGATCCTGTTCTGCGTGGAAAGAGA<br>TTTTTAACCTCTCACAAGAAGAATTTGAAGACCGTGACCTCGAATGGCATTGAATTTATG<br>GATGACTATTCTCAACTTATAATTCTCCTAGCTACAGGTGATGACTCTCTTGTCGATTAT<br>GTAAAGTCTCCTGGACAATTCAATGTTTTCCCGGATGGCACGTTGTTCACAACTGAAGT<br>CTATCCAAATGACCTCGATACAACCGCTATCGGCCTTACCGTCACGGACCATGTCGAC<br>GTGGGCACAAAACATAAGATTATGGATGAAATGCTCGAGTATCGGGACTCCGATGCAA<br>TCATTCAAGTATATTTCGACCATTCCCGTCCTCGCATCGATCCAGTAATCTGTATCAATG<br>TGTTGAACCTCTTTTACGAAAATGGGAGAGGTCATGAACTTCCCGAGACACTTGACTGG<br>GTCGAACAGGTTCTTACACATCGCGCGTACATATCAGGGACAACCTATTATATTGGGGC<br>CGACGTATTTTTGTTCTTCCTCTCCCGCCTCCTCCAAACTTCTGCGGAAGTTCGTCGAC<br>GTCTCGGATCAATCTTCAAAGAACGGGTCATAGAACGTTTTGGCGTGGAAGGGGATTC<br>CCTTTCTCTTTCTGCTCGAATCATCGCTGCAACGGTAGCAGGCGTCATAGACGAACGT<br>GCTTTAAAGAATCTTCTATCAATGCAATGCGAAGATGGTTCATGGGATAATAGTTGGTTT<br>TGGAAATGGGGAATGTCTTCAATCATGGCGAAGAACGATGGCGTAACCACTGCCCTCG<br>CTATTTGGCCTATTGAAGAGGTCCAATCATTGCGCAAGGAGCAGTCAGAAACGAACGG<br>ATGTACTCAAAATGCTCTTCCCCGCAATCAAGGAGGCATTGGCCATATACAATCTTGGC<br>TTGATCGGGGTACGGTTCGTGGTCCGAAGATTCCTCAGATAACGGAGGAATGGGAATA<br>TGCTGACTGTGCTTGGGAGATGATGGAAAGCATAACAACTACGCCCAAGCTCTACTTAA<br>GGTAG
	T153	Protein	MTPTDVHNTSSHSNLYTAIIFDLGDVLFTWSLSSNPPLPEKLLRRILSCSHWFEYEKGNINEA<br>EVYSRVAKDFLVDPAAVKDTLQVAQDSLQSNTKMLEVIQELKEAGLMIYAMSNISAPSWEIL<br>ERKATPSQWALFDHVFTSASAHQRKPNLGFFRHVIERTGIDPSRTILVDDKLENVLTARSFG<br>MHGIIFDNESKVIKDLKNLCYDPVLRGKRFLTSHKKNLKTVTSNGIEFMDDYSQLIILLATGDD<br>SLVDYVKSPGQFNVFPDGTLFTTEVYPNDLDTTAIGLTVTDHVDVGTKHKIMDEMLEYRDS<br>DAIIQVYFDHSRPRIDPVICINVLNLFYENGRGHELPETLDWVEQVLTHRAYISGTTYYIGADV<br>FLFFLSRLLQTSAEVRRRLGSIFKERVIERFGVEGDSLSLSARIIAATVAGVIDERALKNLLSM<br>QCEDGSWDNSWFWKWGMSSIMAKNDGVTTALAIWPIEEVQSLRKEQSETNGCTQNALPR<br>NQGGIGHIQSWLDRGTVRGPKIPQITEEWEYADCAWEMMESITTTPKLYLR*
**DS3**	T153	Gene	ACTAACAGCATCACAGCATTCTCAAAGACAGTCCGAAATAAAGGTGCATTATTGCAGGA<br>AGGTGGAACTAAGGAATGAATGAATGAATGAATGAACTCCGTTTATACCGTCGGCAAGC<br>CATAATACAGGGCTAGGAGGCGGGGGTAAAGTAGTGACTCCACTCTGATAGTAAGTAC<br>TGGTAGTCAGTATACTGTGTCGTTCTCCATGAAATCATTGTTGTTAGAACTCTCCCCGC<br>AGACCTTTGTGTGGTGGATTTAAGGCGCGGTCACGTCGCAAATAACTCGAGTAAAACAT<br>TGGATGTTGGGTGTAGTTTCGGGGAACAAGGTGGGTGGCTTGTGACGGGCCTCTTTGT<br>GGAACTCAGGAAGATGCCAAATTAGGCGAGGAATATTCAGTTGTGCATCAGGAGACGG

			TTGGAATGAAGAAGCATGTGTATTTAAATCCGAAAGATGGATACAATCCAACGAAAAAA<br>TGACGCCCACTGATATTGACAATACTTCTTCTCACTCCAACCTCTATACTGCCATCATAT<br>TTGATCTCGGTGACGTGCTCTTCACTTGGTCTCTCTCTTCAAACCCTCCTCTTCCAGAA<br>AAACTGCTTCGTAGGATTCTGTCTTCTTCTCACTGGTTCGAGTATGAAAAGGGCAATATT<br>AACGAAGCCGAGGTATACTCCCTGGTTGCAAGGGATTTTCTAGTCGATCCTGCTGCATT<br>GAAGAACACCTTGCAAGTTGCGCAGGACTCTTTGCAGAGCAACAAGAAAATGCTGGGC<br>GTTATTCAGGAACTCAAGGAAGCCGGCCTCTTGATTTATGCTATGTCCAACATATCAGC<br>ACCCTACTTGGAGATTTTGGAAAGAAAGGCGACCCCATCACAGTGGGCACTTTTTGATC<br>ACGTATTCACATCGTATGTTTCCTCCTTCGACTCTATCTATGTTGGACGCTGCTTAACTA<br>GAAGTCTATTCTCAGTGCTTCTGCCCACCAACGTAAACCGAATCTCGGTTTCTTCAAGC<br>ATGTTATCGAGAGGACTGGGATTGATCCTTCGTGCACCATTTTTGTTGATGACAAGCTG<br>GAGAATGTCTTGACAGCTCGATCCTTTGGAATGCATGGTATTATTTTTGACAACGAGTC<br>AAAAGTTATCAAAGACCTCAAGAATCTTTGCTACGATCCTGTTCTGCGTGGAAAGAGAT<br>TTTTAACCTCACACAAGAAGAATCTGAAGACCGTGACCTCGAATGGCATTGAATTTATG<br>GATGTCTGTTTCTTGAGTATTACGTTCTTCAAATGCCACATCTAACTGATGATTTATATCA<br>GGACTATTCTCAACTTGTGATCCTCCTAGCTACAGGCGATGAGTAAGTCATGATGCTTC<br>TGATTTTCATAGGATCGGAATTGTTGATATAGTTGAAAAAGCTCTCTTGTCGATTATGTC<br>AAGTCTCCTGGACAATTCAATGTTTTCCCGGATGGCACGTTGTTCACAACTGAAGTCTA<br>TCCGTGAGTTTAATTTTACGGTTGCAGAAAACCTGAATGTGACATTTCCAATAGGAATGA<br>CCTCGATACAACCGCTATCGGCCTTACCGTCACGGACCATGTTGATGCGGGCACAAAA<br>CATAAGATTATGGATGAAATGCTCGAGTATCGGGACTCCGATGGGATCATTCAAGTATA<br>TTTTGACCATTCCCGTCCTCGCATTGGTAAGACTTGCCTCTACAAATTGAGCTGATTGAT<br>GGTTGTTACTGACTTACAACCTCTTATATAGATCCAGTAGTCTGTATCAATGTGTTGAAC<br>CTCTTTTGCGAAAATGGGAGAGGTCATGAACTTCCCGAGACACTTGACTGGGTCGAAC<br>AAGTTCTTATACATCGCGCGTACATCTCAGGGACAACCTATTATATTGGGGCCGACGTA<br>TTCTTGTTCTTCCTGTCCCGCCTCCTCCAAAATTCTGCGGAAGTTCGTCGACGTCTCGG<br>ATCGATATTCAAAGAACGGGTTATAGAACGTTTTGGCGTCAAAGGGGATTCCCTTTCTC<br>TTTCTGCTCGAATGATTGCTGCAACGGTAGCAGGCGTTATAGATGAAGGTGCTTTGAAG<br>AATCTTCTATCAATGCAGTGCGAGGATGGTTCATGGGATGATAGTTGGTTTTGGAGATG<br>GGGAATGTCTCCGATCATGGCGAAGAATGATGGCGTAACCACTGCCCTCGCTATTTGG<br>GCTATTGAACGGGTCCAATCATTGCGCAAGGAATAGTCAGAAACGAACAGATGTATTCA<br>AAATGCTCTTCCCCACAATCAGGGAGGTTTTGGCCATATACGATCTTGGCCTGATCGG<br>GGTGCTCCGAAAATTCCTCAGATAGCGGAGGAACGGGATAATATTGACCCTGCTTTTG<br>AGATGATGGAAAGCAAAACATAACAGCTACGGCTGGAGTCCTCTACCCAAGTCAGTTA<br>CCTTTCCAATTTTCTCTCTGCTAGGAATTTGTATGATATATATCAGTATACCTGTCCTCAA<br>CTCAGCCTCATATGGCACGCGCGTAGAAGTTGTTGGTACATATGTGCCGCTCACTAAC<br>ATCGAGAGCTACCGATCGATCGATCCCCTCCTCACGGGGGTCTCAGCTATCTATGATA<br>TTTGCCAGATGACGAGAATTATTCCGCAGAATATGGAGGTGCACAACAAGCTGGAAATC<br>AAGGTGTTAATCCTGAAATCCTTAACAATATGAGCCACTTCATCCATGCTATTGAGGAG<br>ATGCAGGATAATATATTATTGCAAACTTTGAATGAGAC
	T153	Transcript	ATGACGCCCACTGATATTGACAATACTTCTTCTCACTCCAACCTCTATACTGCCATCATA<br>TTTGATCTCGGTGACGTGCTCTTCACTTGGTCTCTCTCTTCAAACCCTCCTCTTCCAGAA<br>AAACTGCTTCGTAGGATTCTGTCTTCTTCTCACTGGTTCGAGTATGAAAAGGGCAATATT<br>AACGAAGCCGAGGTATACTCCCTGGTTGCAAGGGATTTTCTAGTCGATCCTGCTGCATT<br>GAAGAACACCTTGCAAGTTGCGCAGGACTCTTTGCAGAGCAACAAGAAAATGCTGGGC<br>GTTATTCAGGAACTCAAGGAAGCCGGCCTCTTGATTTATGCTATGTCCAACATATCAGC<br>ACCCTACTTGGAGATTTTGGAAAGAAAGGCGACCCCATCACAGTGGGCACTTTTTGATC<br>ACGTATTCACATCTGCTTCTGCCCACCAACGTAAACCGAATCTCGGTTTCTTCAAGCAT<br>GTTATCGAGAGGACTGGGATTGATCCTTCGTGCACCATTTTTGTTGATGACAAGCTGGA<br>GAATGTCTTGACAGCTCGATCCTTTGGAATGCATGGTATTATTTTTGACAACGAGTCAAA<br>AGTTATCAAAGACCTCAAGAATCTTTGCTACGATCCTGTTCTGCGTGGAAAGAGATTTTT<br>AACCTCACACAAGAAGAATCTGAAGACCGTGACCTCGAATGGCATTGAATTTATGGATG<br>ACTATTCTCAACTTGTGATCCTCCTAGCTACAGGCGATGACTCTCTTGTCGATTATGTCA<br>AGTCTCCTGGACAATTCAATGTTTTCCCGGATGGCACGTTGTTCACAACTGAAGTCTAT<br>CCGAATGACCTCGATACAACCGCTATCGGCCTTACCGTCACGGACCATGTTGATGCGG<br>GCACAAAACATAAGATTATGGATGAAATGCTCGAGTATCGGGACTCCGATGGGATCATT<br>CAAGTATATTTTGACCATTCCCGTCCTCGCATTGATCCAGTAGTCTGTATCAATGTGTTG<br>AACCTCTTTTGCGAAAATGGGAGAGGTCATGAACTTCCCGAGACACTTGACTGGGTCG<br>AACAAGTTCTTATACATCGCGCGTACATCTCAGGGACAACCTATTATATTGGGGCCGAC<br>GTATTCTTGTTCTTCCTGTCCCGCCTCCTCCAAAATTCTGCGGAAGTTCGTCGACGTCT<br>CGGATCGATATTCAAAGAACGGGTTATAGAACGTTTTGGCGTCAAAGGGGATTCCCTTT<br>CTCTTTCTGCTCGAATGATTGCTGCAACGGTAGCAGGCGTTATAGATGAAGGTGCTTTG<br>AAGAATCTTCTATCAATGCAGTGCGAGGATGGTTCATGGGATGATAGTTGGTTTTGGAG<br>ATGGGGAATGTCTCCGATCATGGCGAAGAATGATGGCGTAACCACTGCCCTCGCTATT<br>TGGGCTATTGAACGGGTCCAATCATTGCGCAAGGAATAG
	T153	Protein	MTPTDIDNTSSHSNLYTAIIFDLGDVLFTWSLSSNPPLPEKLLRRILSSSHWFEYEKGNINEA<br>EVYSLVARDFLVDPAALKNTLQVAQDSLQSNKKMLGVIQELKEAGLLIYAMSNISAPYLEILE<br>RKATPSQWALFDHVFTSASAHQRKPNLGFFKHVIERTGIDPSCTIFVDDKLENVLTARSFGM<br>HGIIFDNESKVIKDLKNLCYDPVLRGKRFLTSHKKNLKTVTSNGIEFMDDYSQLVILLATGDD<br>SLVDYVKSPGQFNVFPDGTLFTTEVYPNDLDTTAIGLTVTDHVDAGTKHKIMDEMLEYRDS<br>DGIIQVYFDHSRPRIDPVVCINVLNLFCENGRGHELPETLDWVEQVLIHRAYISGTTYYIGAD<br>VFLFFLSRLLQNSAEVRRRLGSIFKERVIERFGVKGDSLSLSARMIAATVAGVIDEGALKNLL<br>SMQCEDGSWDDSWFWRWGMSPIMAKNDGVTTALAIWAIERVQSLRKE*

Table 26. Codon optimized transcript sequences. Codon optimization did not change the amino acid composition of the translated proteins.

DS1 transcript sequence codon optimized (marked in red):
ATGGCATGTCAGTATACCGCCATTATTTTTGATCTGGGTGACGTTCTGTTTAAATGGAGTCAGACCAGCATTA<br>GTACCCGTACCCTGCGTGAAGTTCTGAGCAGTCCGACCTGGTTTGATTATGAACGTGGTCGCCTGAGCGA<br>AGCAGATTGCTATGCAAAAGTTGGCCAGGAATTCATGATGGAACCGGATGAAATTGCACAGGCATTTCAGC<br>AGGCACGTGATAGCGTGCGTGCCGATGATGAACTGATTGCCCTGATTCGCGAACTGAAAGCACAGAGTAAT<br>GGCGAACTGCGTGTTTTTGCCATGAGTAATATTAGCCTGCCGGATTATGAAGTTCTGCGTACCAAACCGGTT<br>GATTGGAGTATTTTTGATCGTGTGTTTACCAGCGGTGCAGCAGGCGAACGTAAACCGAATCTGGGCTTTTT<br>CAAACAGGTGCTGAGTGCAACCGGTGTTGATCCGCAGCGCACCATTTTTGTTGATGATCGTAGCGAAAATG<br>TGCTGAGTGCGCGTAGCCTGGGTCTGTATGGCATTATTTTTGACAATCCGAAAACCGTGGCCCGTGCCCTG<br>CGCAATCTGGTGGGCGATCCGATTACCCGTGGCCGCGCCTTTCTGAAACAGAATGCAGGCCGCCTGCTGA<br>GCGTTACCGATAAAAATGATAAACATGCAGCCGTGGAACTGCGCGAAAATTTTGCCCAGCTGCTGATTCTG<br>GAAGCAACCAATAATCGCACCCTGGTGGATCTGGTTGAACATCCGCGTACCTGGAATTTCTTTCAGGGCAA<br>AGGTCAGCTGACCACCGAACAGTTTCCGTTTGATCTGGATACCACCAGTCTGGCCCTGACCGTTCTGCGC<br>CGTGATAAAAAACTGGCATTTTCTATTATGGACGAAATGCTGGAAGATGATCAGACCTATTTTGATCATCGTC<br>GTCCGCGCTTTGATCCGATTGTGTGTGTTAATACCCTGAGTCTGTTTTATAGTTATGGCCGCGGTAATGAAC<br>TGCAGAAAACCCTGCAGTGGGTTCATGAAGTTCTGCGCAATCGTGCCTATCTGGAAGGTACCCGTTATTAT<br>GAAACCCCGGAATGTTTTCTGTTTTTCACCAGTCGTCTGCTGGCCAGTAGTGATGATCAGCAGCTGCATGC<br>CATGCTGAAACCGCTGCTGAAAGAACGCGTTCAGGAACGTATTGGTGCAAAAGGTGACGCCCTGGCCCTG<br>GCCATGCGTATTATTGTGACCGATTTTGTGGGTATTCGCAATGAAGTTGATCTGCGTGCACTGCTGCCGCTG<br>CAGTGTGAAGATGGTGGCTGGGAAATTGGTTGGATGTATAAATATGGTAGTAGCGGTATTCGTATTGGTAATC<br>GTGGTCTGACCACCGCCCTGGCAATTAAGGCCGTGGAAGCAATGCGCAATCCGAGTATTCTGATGAGTCAT<br>AGCCCGAGCCCGAGTAGCAATATTGTGGCAACCCCGAGTAGTAAAGTTAAAAGTGCAGCAGCCACCGGTC<br>GCCATCAGCGTACCAAAAGCCTGCGTGATAGCCTGCAGTGGCTGTGGCATGTTGGCAAAAGTAGTAAACC<br>GATTGAAGCATAA
DS3 transcript sequence codon optimized (marked in red):
ATGACCCCGACCGATATTGATAATACCAGCAGTCATAGTAATCTGTATACCGCAATTATTTTCGATCTGGG<br>CGATGTTCTGTTTACCTGGAGCCTGAGCAGCAATCCGCCGCTGCCGGAAAAACTGCTGCGTCGTATTCTG<br>AGCAGCAGTCATTGGTTTGAATATGAAAAAGGTAACATCAACGAGGCAGAAGTTTATAGTCTGGTTGCCCG<br>TGATTTTCTGGTGGATCCGGCCGCACTGAAAAATACCCTGCAGGTTGCCCAGGATAGTCTGCAGAGCAAT<br>AAGAAAATGCTGGGTGTGATTCAGGAACTGAAAGAAGCCGGTCTGCTGATCTATGCCATGAGCAATATTA<br>GCGCCCCGTATCTGGAAATTCTGGAACGTAAAGCAACCCCGAGTCAGTGGGCCCTGTTTGATCATGTGTT<br>TACCAGTGCCAGTGCCCATCAGCGTAAACCGAATCTGGGCTTTTTCAAACATGTGATTGAACGTACCGGTA<br>TTGATCCGAGCTGTACCATTTTTGTTGATGATAAACTGGAAAACGTGCTGACCGCCCGTAGTTTTGGTATG<br>CATGGTATTATTTTTGACAACGAAAGTAAGGTTATCAAGGATCTGAAAAATCTGTGTTATGATCCGGTGCTG<br>CGCGGTAAACGTTTTCTGACCAGTCATAAAAAGAATCTGAAAACCGTGACCAGCAATGGCATTGAATTCAT<br>GGATGATTATAGCCAGCTGGTTATTCTGCTGGCCACCGGTGACGATAGCCTGGTGGATTATGTTAAAAGT<br>CCGGGTCAGTTTAATGTTTTTCCGGATGGCACCCTGTTTACCACCGAAGTTTATCCGAATGATCTGGATAC<br>CACCGCCATTGGTCTGACCGTTACCGATCATGTGGATGCCGGCACCAAACATAAAATTATGGATGAAATG<br>CTGGAGTATCGCGATAGCGATGGCATTATTCAGGTTTATTTTGATCATAGCCGCCCGCGCATTGATCCGGT<br>GGTTTGCATTAATGTGCTGAATCTGTTTTGTGAAAACGGTCGCGGCCATGAACTGCCGGAAACCCTGGAT<br>TGGGTTGAACAGGTTCTGATTCATCGCGCATATATTAGCGGCACCACCTATTATATTGGTGCAGATGTGTT<br>TCTGTTTTTCCTGAGCCGCCTGCTGCAGAATAGCGCCGAAGTGCGCCGTCGTCTGGGCAGCATTTTTAAA<br>GAACGCGTGATTGAACGCTTTGGTGTTAAAGGTGACAGCCTGAGCCTGAGTGCACGCATGATTGCCGCCA<br>CCGTGGCAGGCGTTATTGATGAAGGTGCACTGAAAAACCTGCTGAGTATGCAGTGTGAAGATGGTAGCTG<br>GGATGATAGTTGGTTTTGGCGCTGGGGCATGAGCCCGATTATGGCAAAAAATGATGGCGTTACCACCGCC<br>CTGGCCATTTGGGCAATTGAACGCGTGCAGAGTCTGCGTAAAGAA

Table S27. List of primer sequences for heterologous expression of DS1-3 proteins with varying length. Restriction sites are underlined.

Name	Sequence (5'->3')
DS1 N-Term FW	CAGTCAGCTAGCATGGCATGTCAGTATACCGC
DS1 short FW	CAGTCAGCTAGCATGGAACCGGATGAAATTGCAC
DS1 short REV	CAGTCAAAGCTTTTAACTCATCAGAATACTCGGATTG
DS2-FW	CGCGGATCCATGCTCCTACTGATGTTAC
DS2-REV	CCCAAGCTTCTACCTTAAGTAGAGCTTGGGC
DS2 N-term FW	CAGTCAGCTAGCATGACTCCTACTGATGTTCACAAT
DS2 short FW	GCTAGCATGCTGGAGGTTATTCAGGAAC
DS2 short REV	CAGTCAAAGCTTCTACTCCTTGCGCAATGATTG
DS3 N-term FW	GCTAGCATGACCCCGACCGATATTG
DS3 short FW	CAGTCAGCTAGCATGCTGGGTGTGATTCAG
DS3 short REV	CAGTCAAAGCTTCTATTCTTTACGCAGACTCT

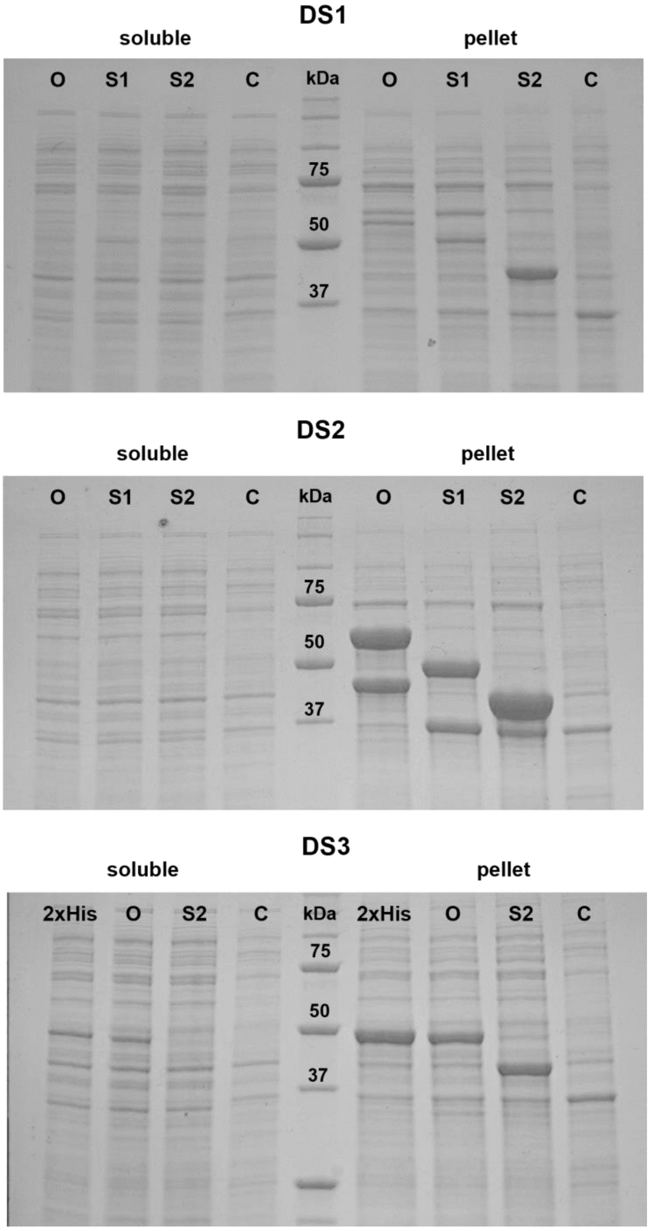

Figure S20. SDS-PAGEs of heterologously expressed DS1-DS3 proteins of different length. O= originally predicted sequence, S1= shorter protein without C-terminal hydrophobic patch, S2= shorter protein without C-terminal hydrophobic patch and shorter N-terminus starting at an alternative start codon, 2xHis version of DS3-O with a His-tag at the N- and C-terminus. C= control sample, empty pET28a(+) vector expressed in *E. coli* BL21 (DE3).

8. Enzyme assay and compound purification

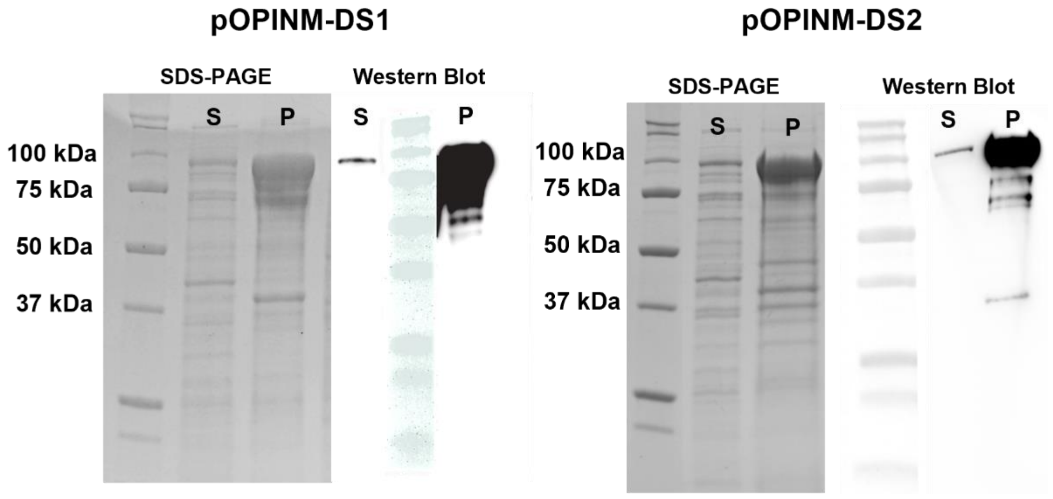

Figure S21. SDS-Pages and Western Blots of heterologously produced DS1 and DS2 proteins tagged with MBP at the N-Terminus. S, soluble fraction; P, pellet fraction.

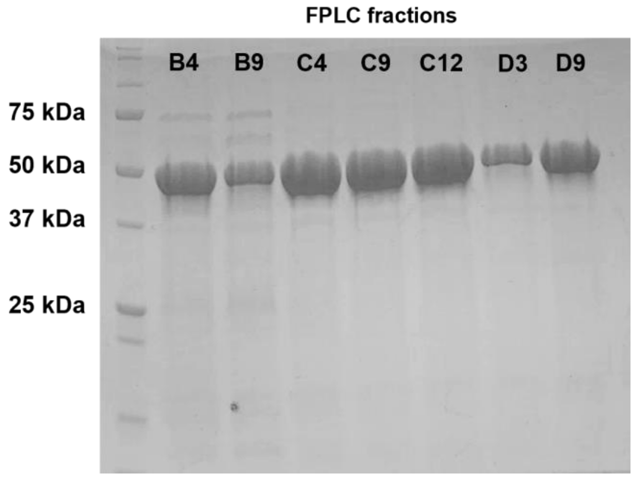

Figure S22. SDS-PAGE of selected FPLC fractions from purification of big scale DS3 protein expression.

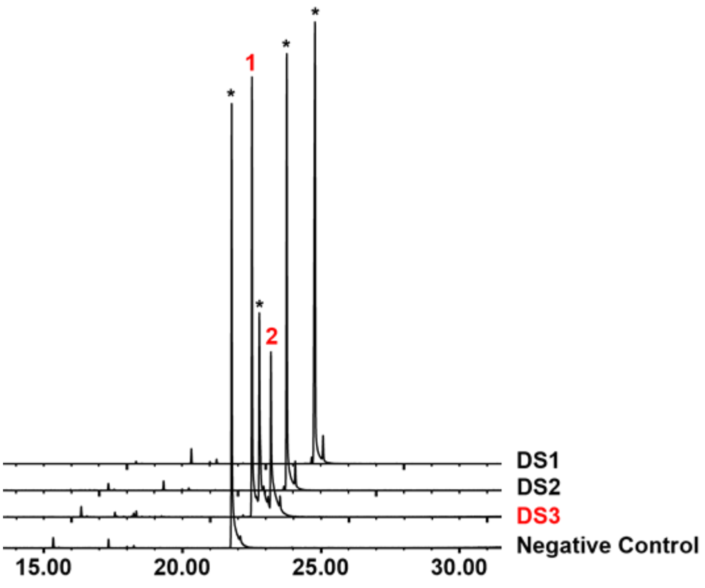

Figure S23. GC-chromatogram comparison of enzyme assays with native DS1-DS3 *E. coli* soluble protein extracts incubated with FPP as substrate. As negative control served a soluble protein extract of an induced *E. coli* empty pET28 vector culture. Cyclization products of reaction with DS3 are marked with 1 = compound **22**, 2 = compound **23**, and * represents farnesol.

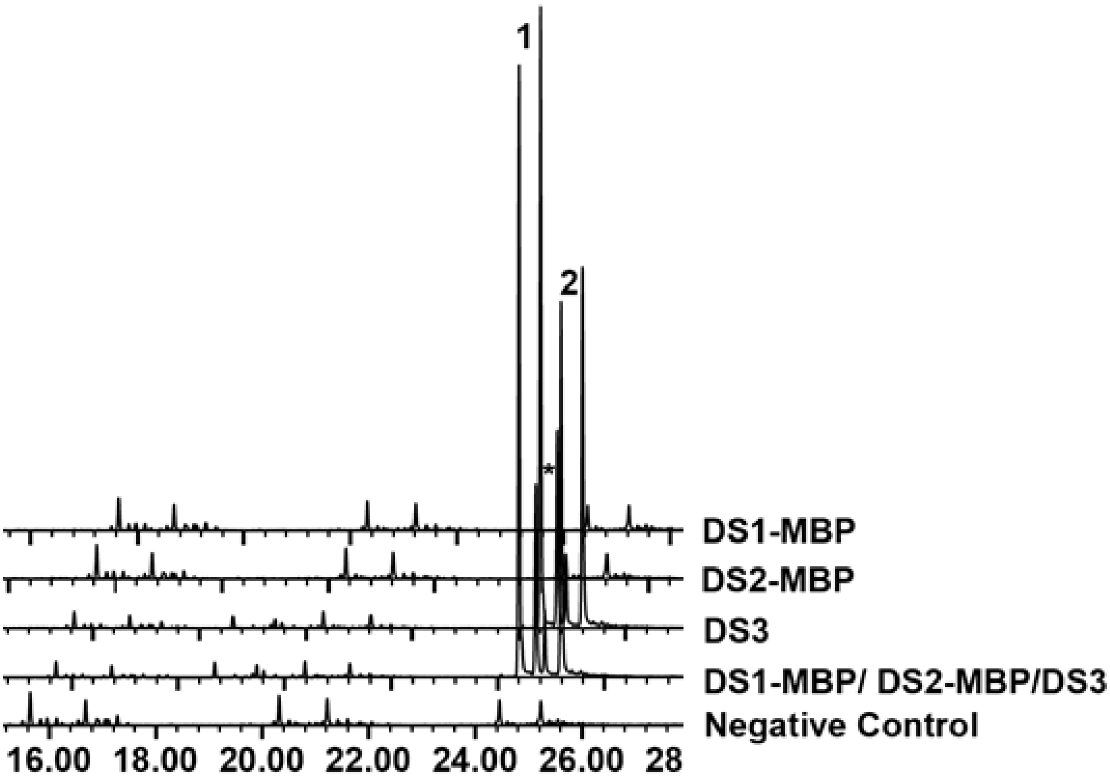

Figure S24. GC-chromatogram comparison of enzyme assays with Ni-NTA chromatographically purified DS1-MBP, DS2-MBP, DS3 or a mixture of proteins proteins incubated with FPP as substrate. As negative control a soluble protein extract of an induced *E. coli* empty pET28 vector culture was purified by Ni-NTA affinity chromatography and used in the assay instead of heterologous protein. Cyclization products of enzymatic reaction are marked with 1 = compound **22**, 2 = compound **23**, and * represents farnesol.

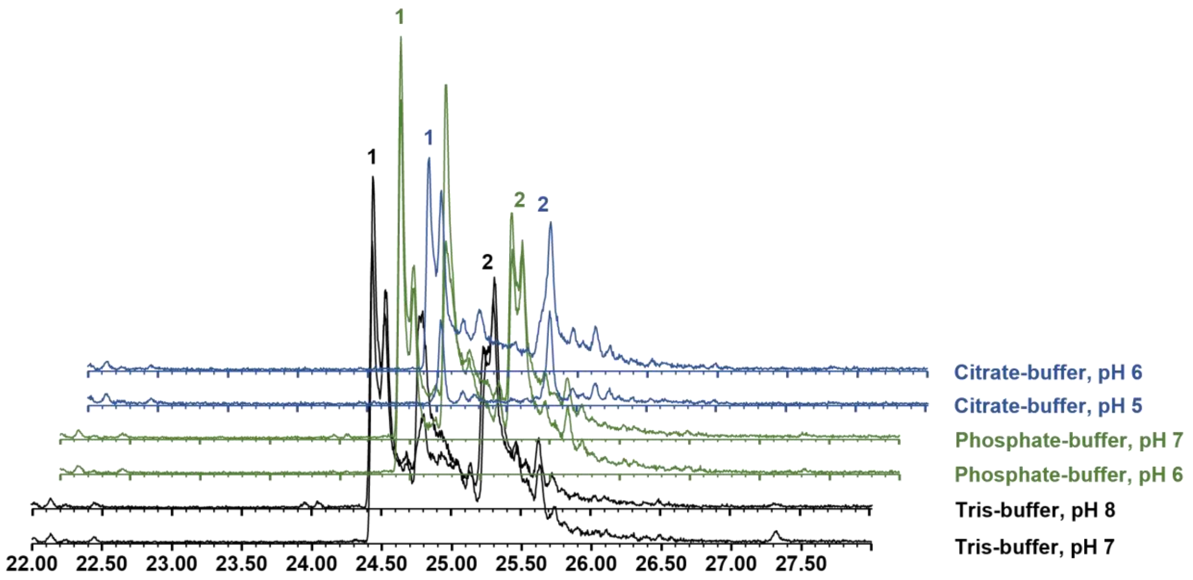

Figure S25. GC-chromatogram comparison of enzyme assays with purified DS3 protein incubated with FPP as substrate in various buffers adjusted to different pH values. Cyclization products of reaction are marked with 1 = compound **22**, 2 = compound **23**, and * represents farnesol.

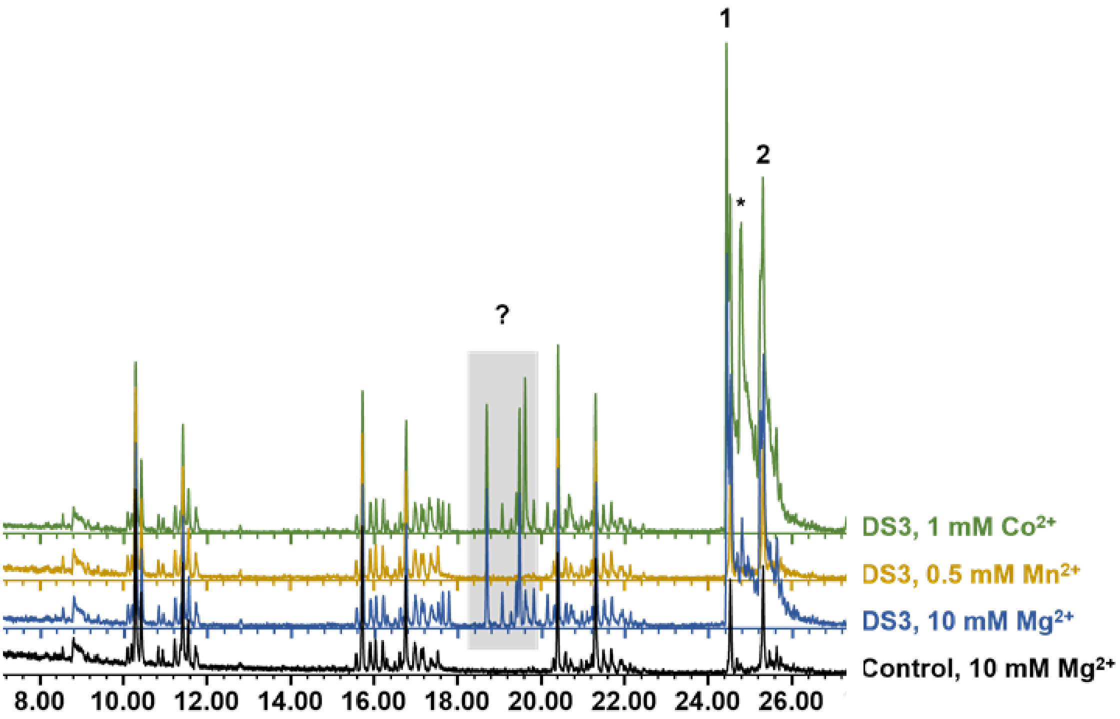

Figure S26. Comparison of GC-chromatogram sections obtained from enzyme assays with purified DS3 protein incubated with FPP as substrate and different divalent metal cofactors. As negative control a soluble protein extract of an induced *E-coli* empty pET28 vector culture was purified by Ni-NTA affinity chromatography and used in the assay instead of heterologous protein. Cyclization products of reaction with DS3 are marked with 1 = compound **22**, 2 = compound **23**, and * represents farnesol. Yet unidentified enzyme products are highlighted in grey and marked with “?”.

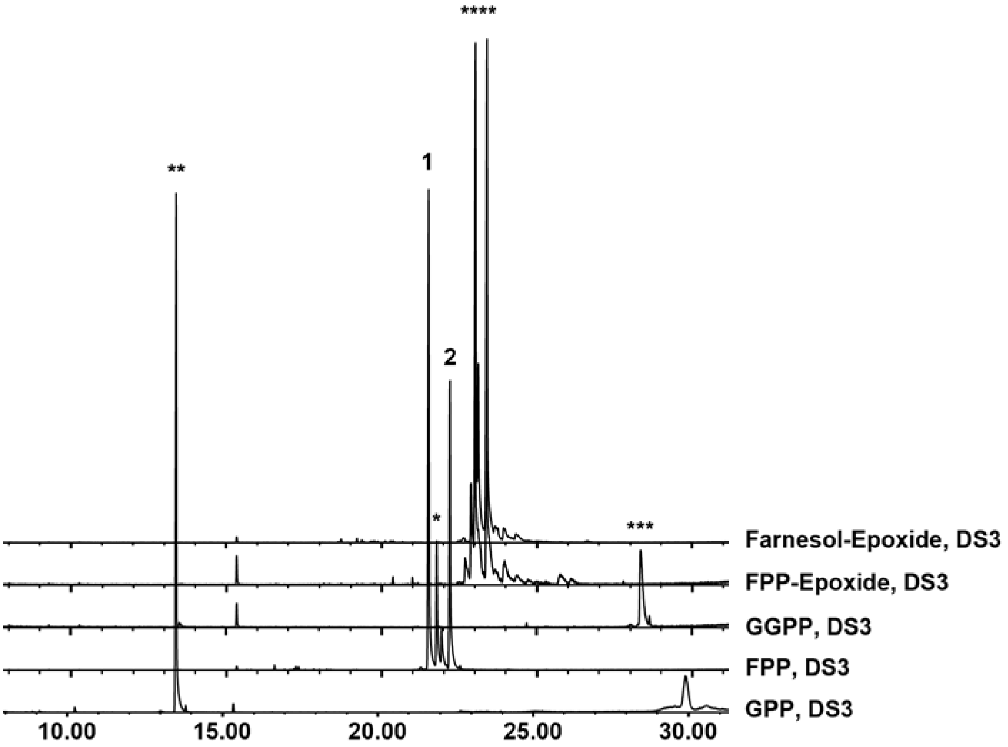

Figure S27. Comparison of GC-chromatogram sections from enzyme assays with purified DS3 protein incubated with different substrates. Cyclization products of reaction with DS3 are marked with 1 = compound **22** and 2 = compound **23**. Dephosphorylated products are marked with asterisks (* farnesol, ** geraniol, *** geranylgeraniol, **** farnesolepoxide).

9. Structure elucidation of compounds 22 and 23

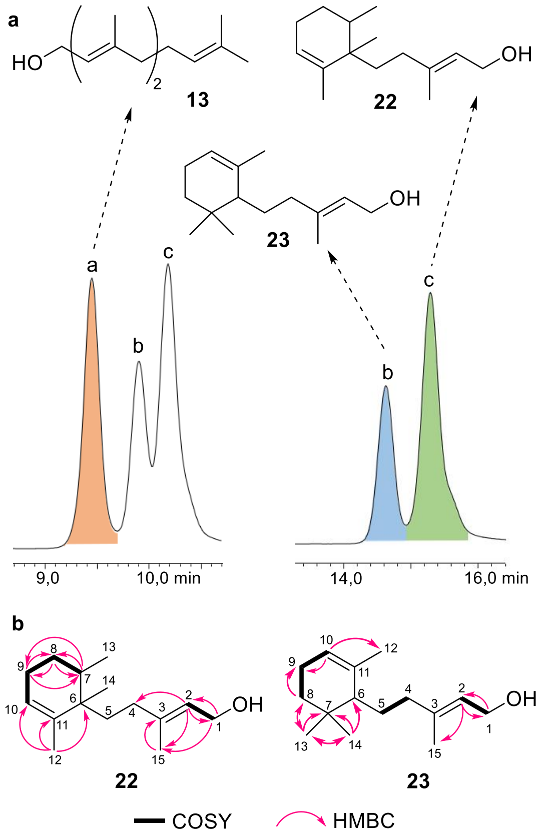

Figure S28. **a** Two steps HPLC separation chromatograms to remove dephosphorylated farnesol **13** from mixture of target compounds and the final separation of enzymatically formed compounds **22** and **23**. **b** ^{1}H-^{1}H COSY and key HMBC correlations in compounds **22** and **23**. The ^{1}H NMR spectroscopy showed proton resonances, which include a doublet methyl at δ_H 0.86 (3H, d, J = 6.7 Hz, H-13), singlet methyl at δ_H 0.87 attached to a quarternary carbon (3H, s, H-14), two methyls at δ_H 1.61 (3H, m, H-12) and δ_H 1.68 (3H, s, H-15) attached to olefinic system, two olefinic methines at δ_H 5.41 (m, H-2) and δ_H 5.42 (m, H-10). Furthermore, the undeniable chemical shift at δ_H 4.15 (2H, d, J = 6.8 Hz, H-1) suggests the presence of allylic alcohol. These proton resonances correlate with existence of substituted cyclohexene ring. Our theory of two double bonds was supported by ^{13}C NMR signals at δ_C 122.4 (C-10), 122.8 (C-2), 139.7 (C-11) and 141.1 (C-3). The presence of alcohol was endorsed by appeared signal at δ_C 59.6 (C-1). The existence of main cyclohexene core was supported by the finding of reciprocal ^{1}H-^{1}H COSY correlations between H-7, H-8, H-9 and H-10. Comprehensive HMBC analysis revealed the correlation from H-1 to C-2 and C-15 that indicates the attachment of C-15 methyl group to allylic alcohol. The position of a second double bond was also assigned by HMBC correlation of H-12 to olefinic C-10 and C-11. In addition, H-12 correlation to sp^3 quaternary carbon C-6 indicates the presence of aliphatic chain in *α* position. Additional HMBC correlations from H-7 to C-8 and C-9, from H-8 to C-9, C-7 and C-6, from H-9 to C-8, C-7 and C-10 confirmed that the main core of alcohol **22** is formed by 6,7,11-trimethylcyclohexene substituted at C-6 position. Comparative NMR analysis identified a very similar chemical shift pattern for the structurally related compound **23**, which differ only in position of C-14 methyl group and a new characteristic CH signal at δ_H 1.42.

Table S28. NMR spectral data ($CDCl_3$, at 300 K) for compound **22**.

Pos.	δ_H, mult. (*J* in Hz)[a]	δ_C, type[b]	COSY[a]	HMBC[a]
1	4.15, d (6.8)	59.6	2, 15	2, 3
2	5.41, m	122.8	1, 4, 15	15
3	-	141.1	-	-
4	1.72, m	34.2	14	-
5	1.50, m	35.0	-	11, 6, 15, 7
6	-	40.5	-	-
7	1.47, m	19.3	8, 9	6, 11, 14
8	1.44, m	27.2	7, 9	9, 6
9	1.97, m	25.7	8, 7, 12	10, 12, 13
10	5.42, m	124.4	9	12
11	-	139.7	-	-
12	1.61, m	16.7	-	6, 10, 11
13	0.86, d (6.7)	16.0	4	11, 6, 5, 8
14	0.87, s	21.2	4	11, 6, 5, 8
15	1.68, s	33.4	5	2, 3, 4

[a] 600 MHz for ^{1}H NMR, COSY, [b] 150 MHz for ^{13}C

Table S29. Comparison of NMR spectral data of compound **22** with literature data.

	Literature[38]		Compound 22	
Pos.	δ_H, mult. (*J* in Hz)[a]	δ_C, type[b]	δ_H, mult. (*J* in Hz)[c]	δ_C, type[d]
1	4.39, d (6.9)	58.9	4.15, d (6.8)	59.6
2	5.39, br s	124.5	5.41, m	122.8
3	-	139.6	-	141.1
4	1.88, m<br>1.91, m	34.3	1.72, m	34.2
5	1.41, m<br>1.46, m	35.1	1.50, m	35.0
6	-	40.5	-	40.5
7	1.62, m	33.4	1.47, m	19.3
8	1.31, m	27.2	1.44, m	27.2
9	1.82, m<br>1.88, m	25.8	1.97, m	25.7
10	5.69, m	125.7	5.42, m	124.4
11		137.5	-	139.7
12	1.52, s	19.3	1.61, m	16.7
13	0.73, d (6.1)	15.9	0.86, d (6.7)	16.0
14	0.76, s	2.1	0.87, s	21.2
15	1.67, s	16.6	1.68, s	33.4

[a] pyridine-d_5, 600 MHz; [b] pyridine-d_5, 150 MHz; [c] $CDCl_3$, 600 MHz; [d] $CDCl_3$, 150 MHz

Table S30. NMR spectral data ($CDCl_3$, at 300 K) for compound **23**.

Pos.	δ_H, mult. (*J* in Hz)[a]	δ_C, type[b]	COSY[a]	HMBC[a]
1	4.15, d (6.9)	59.6	2, 15	2, 3
2	5.41, m	123.2	1, 15	-
3	-	140.8	-	-
4	2.05, m	40.5	-	2, 3
5	1.55, m	29.7	4	-
6	1.42, m	49.2	-	9
7	-	32.7	-	-
8	1.42, m	31.8	9	9, 13, 14
9	1.96, m	23.2	-	-
10	5.29, m	120.3	9, 12	-
11	-	135.7	-	-
12	1.67, s	23.6	-	-
13	0.87, s	27.7	-	6, 7, 8, 14
14	0.93, s	27.6	-	6, 7, 8, 13
15	1.68, s	16.5	-	4, 6

[a] 600 MHz for ^1H NMR, COSY, [b] 150 MHz for ^{13}C

Table S31. Comparison of NMR spectral data of compound **23** with literature data.

	Literature[39]		Compound 23	
Pos.	δ_H, mult. (*J* in Hz)[a]	δ_C[b]	δ_H, mult. (*J* in Hz)[c]	δ_C[d]
1	4.10, d (7.0)	59.3	4.15, d (6.9)	59.6
2	5.38, m	123.1	5.41, m	123.2
3	-	140.3	-	140.8
4	2.05, m	40.3	2.05, m	40.5
5	1.3 – 1.6, m	29.4	1.55, m	29.7
6	1.20, m	48.9	1.42, m	49.2
7	-	32.5	-	32.7
8	1.3 – 1.6, m	31.5	1.42, m	31.8
9	1.93, m	22.9	1.96, m	23.2
10	5.27, s	120.0	5.29, m	120.3
11	-	136.4	-	135.7
12	1.66, m	23.4	1.67, s	23.6
13	0.83, s	27.5	0.87, s	27.7
14	0.90, s	27.4	0.93, s	27.6
15	1.66, m	15.9	1.68, s	16.5

[a] $CDCl_3$, 200 MHz; [b] $CDCl_3$, 50 MHz; [c] $CDCl_3$, 600 MHz; [d] $CDCl_3$, 150 MHz

10. Agar Diffusion Test against *Termitomyces* sp. T153

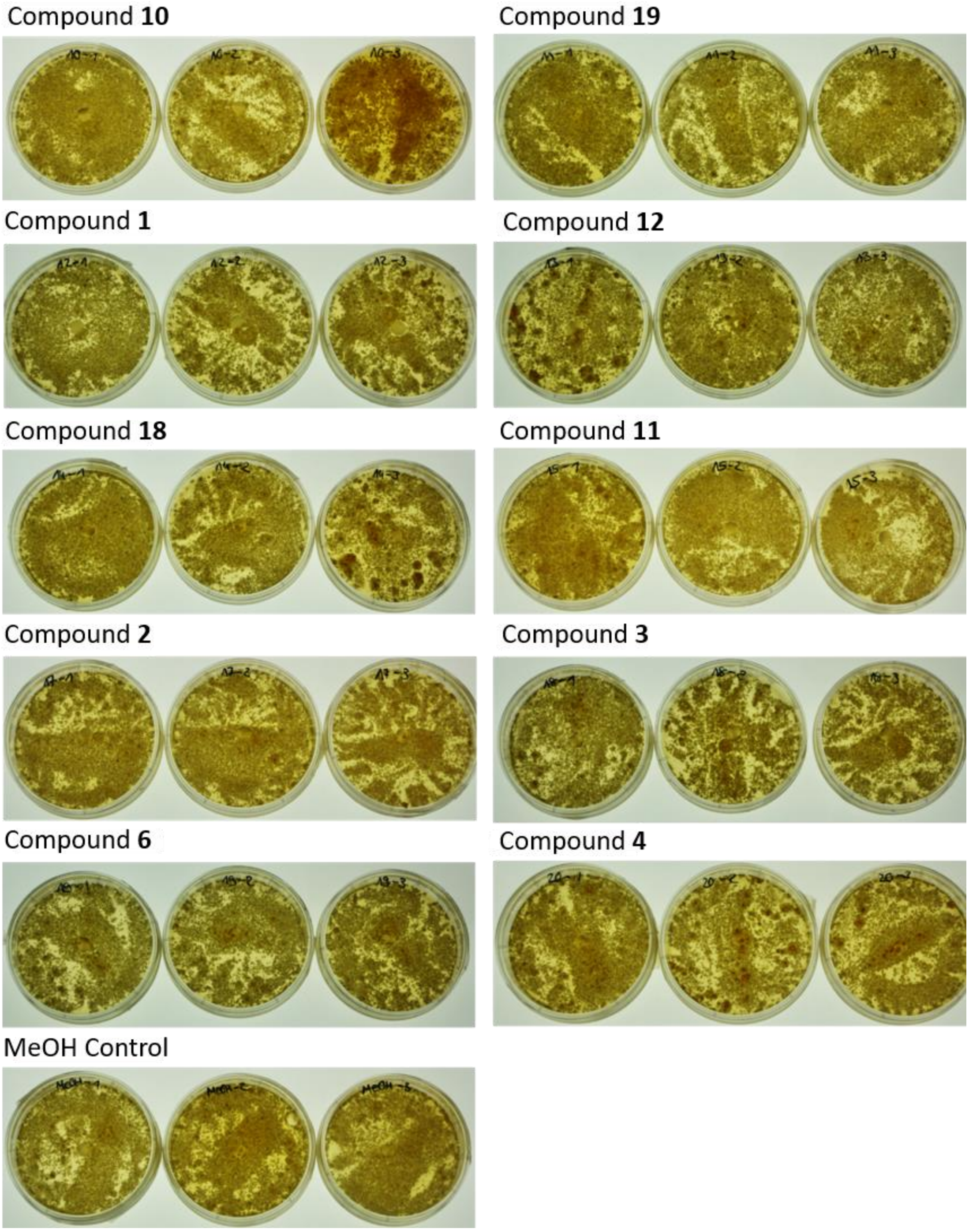

Figure S29. Pictures of disc diffusion assay of isolated and synthesized drimenol derivatives against *Termitomyces* sp. T153. Substances were tested in triplicates. *Termitomyces* sp. T153 was inoculated on small PDA plates (200 µl mycelium suspension) and incubated for one day at room temperature. The next day, paper discs soaked with 10 µL stock solutions (1 mg/mL in MeOH) were placed in the middle of inoculated *Termitomyces* plates and growth was monitored every day. Pictures were taken after 12 days.

11. Appendix HRMS and GC-MS Spectra

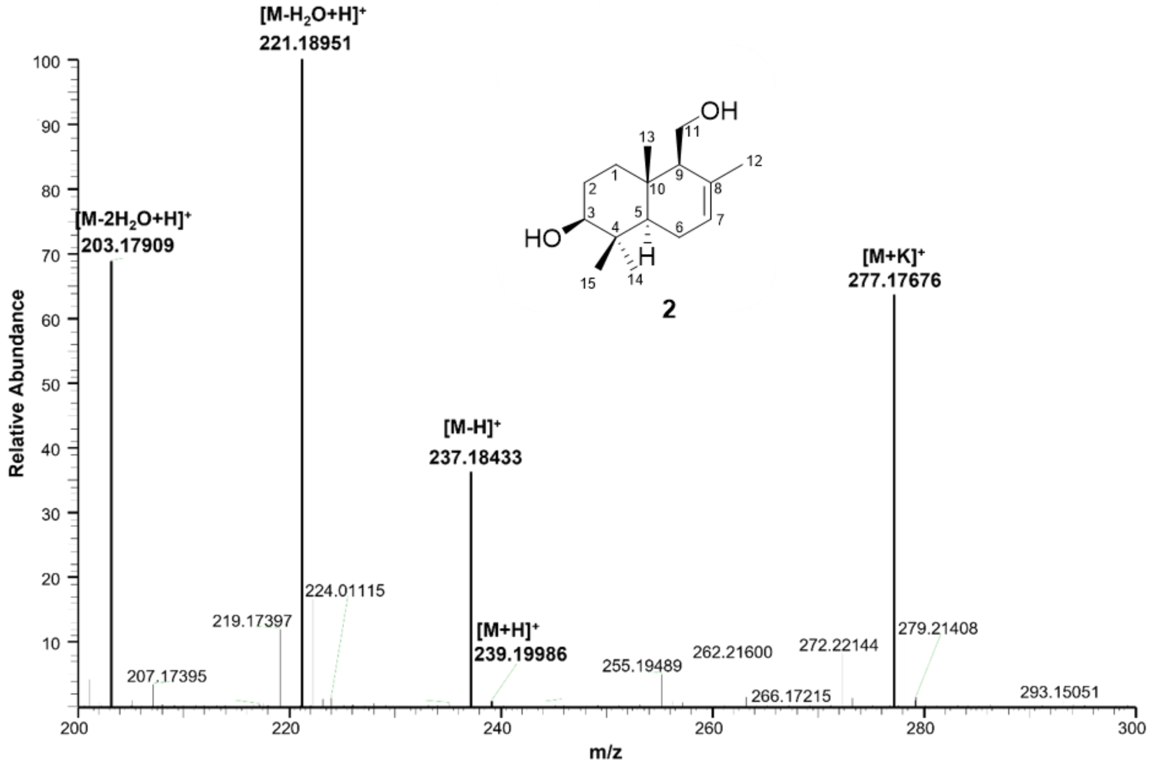

Figure S30. ESI-HRMS (+) spectrum of compound **2**.

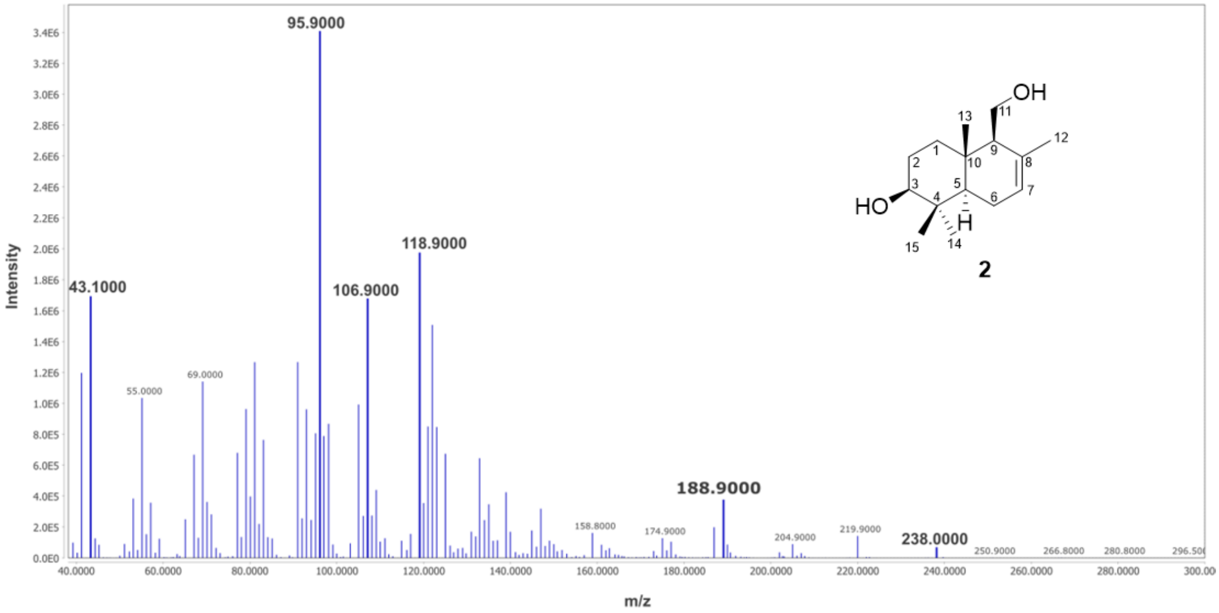

Figure S31. GC-MS spectrum of compound **2**.

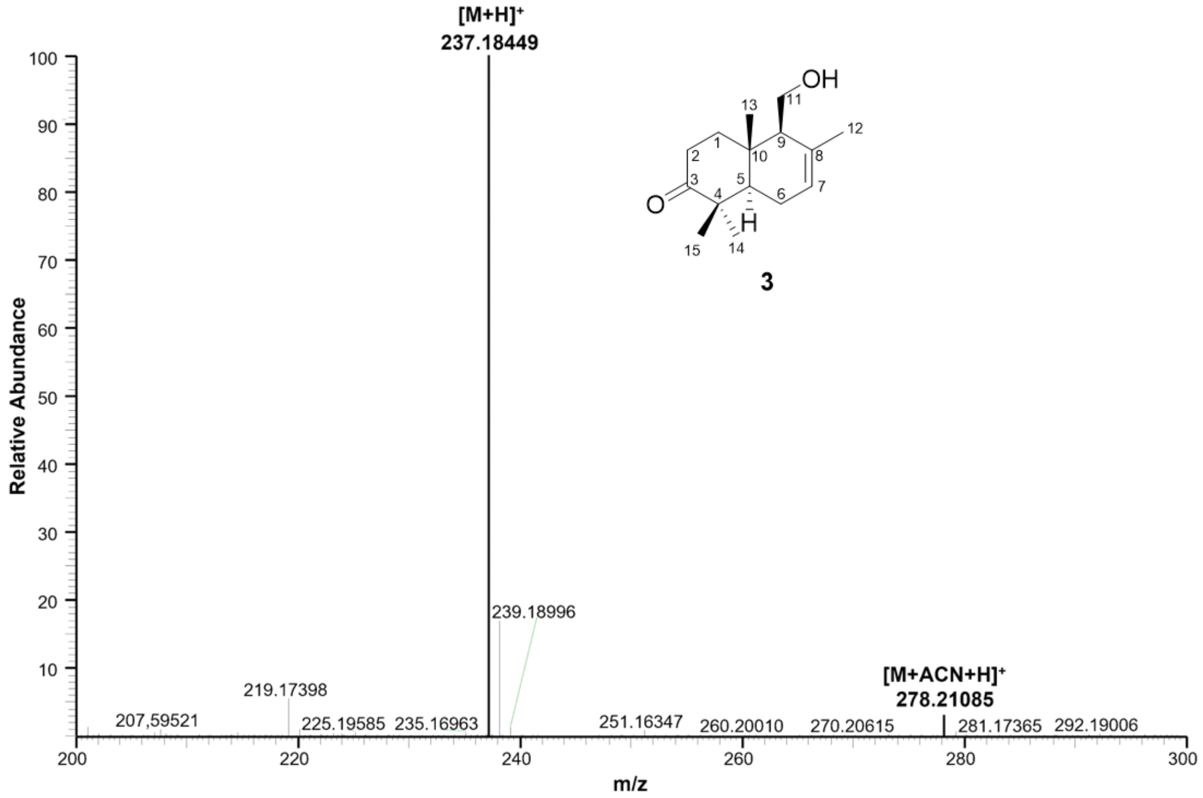

Figure S32. ESI-HRMS (+) spectrum of compound **3**.

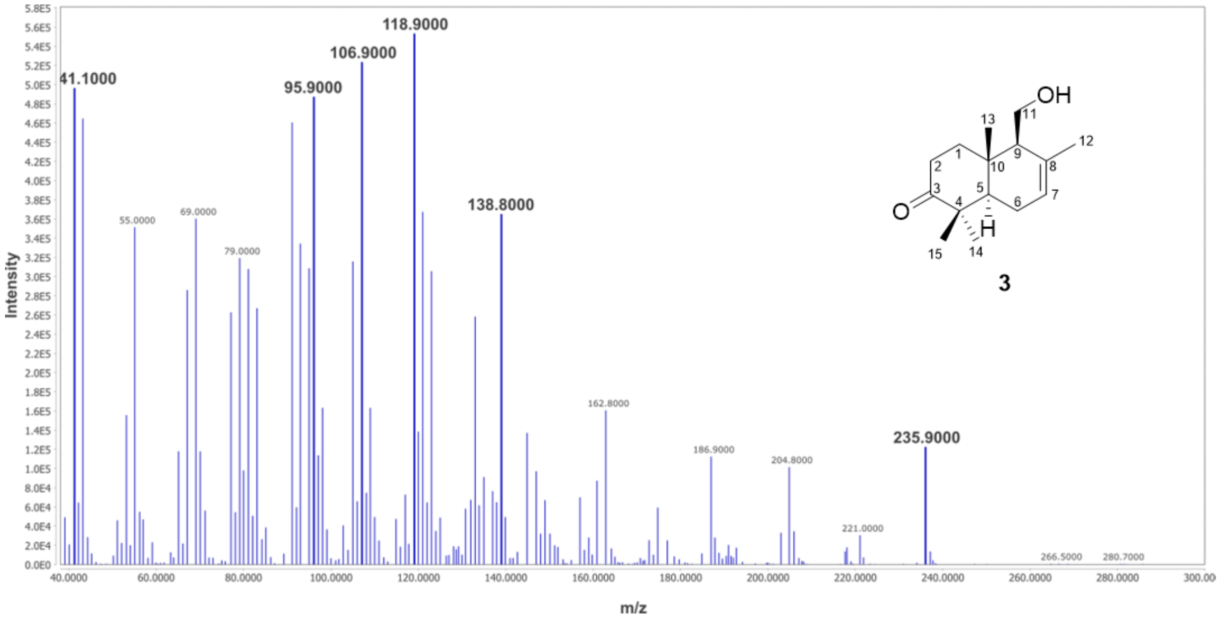

Figure S33. GC-MS spectrum of compound **3**.

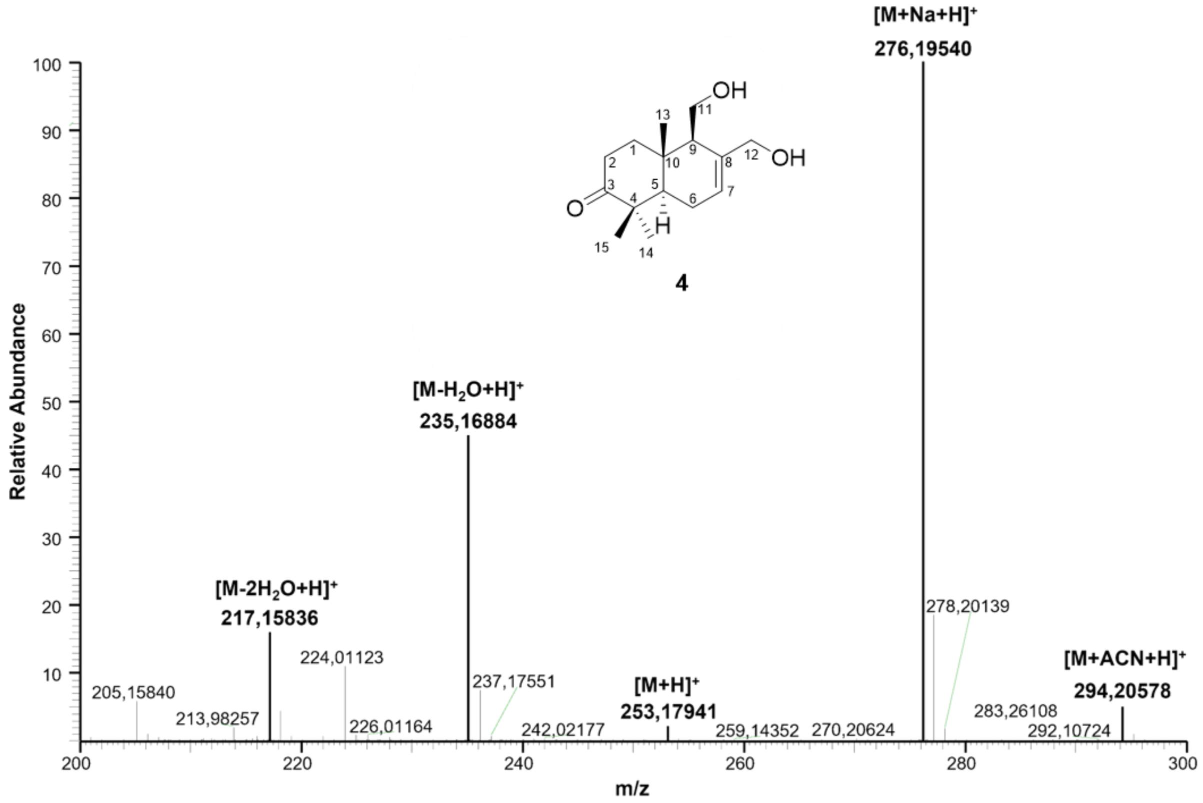

Figure S34. ESI-HRMS (+) spectrum of compound **4**.

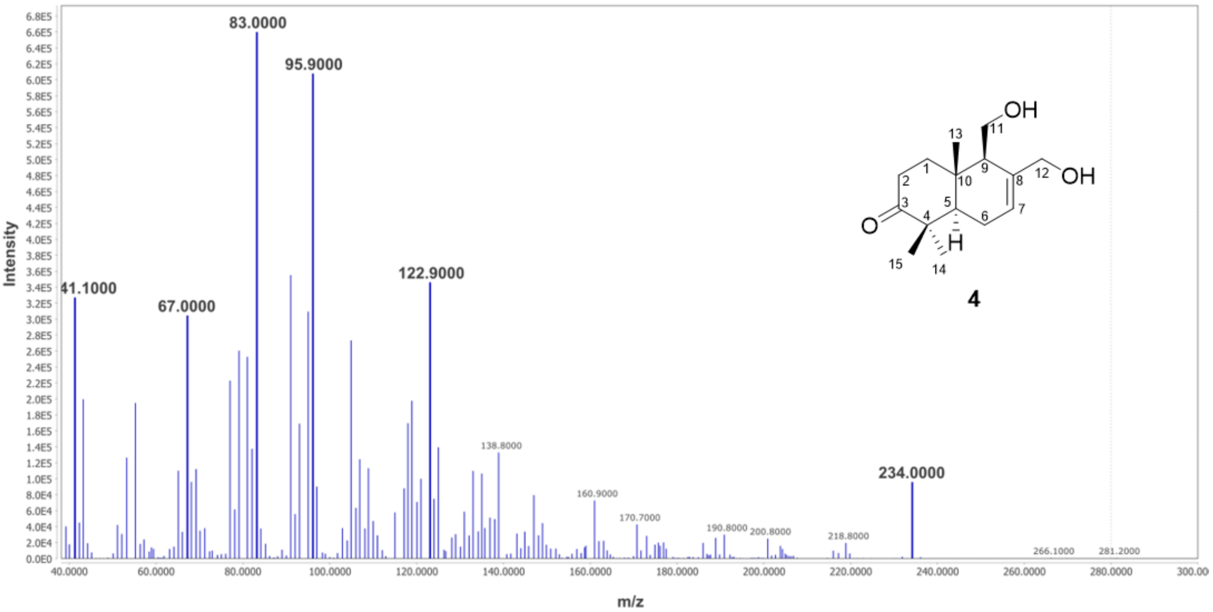

Figure S35. GC-MS spectrum of compound **4**.

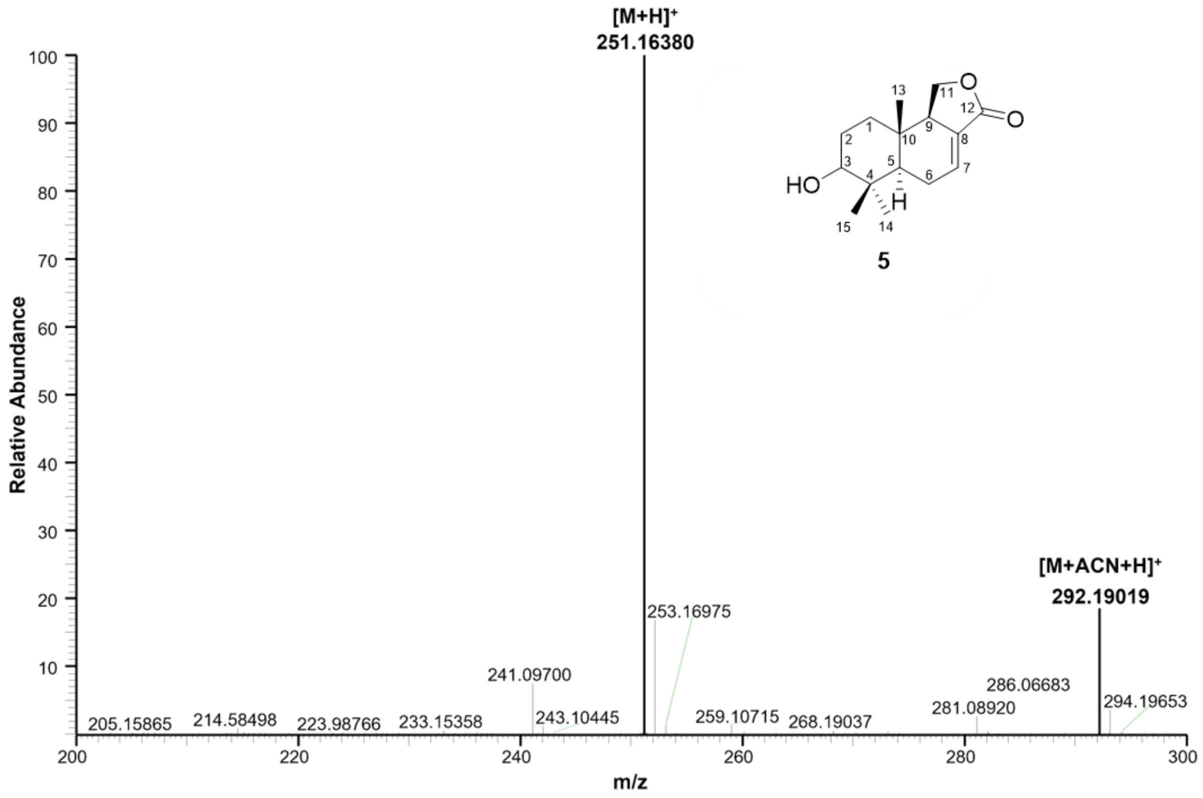

Figure S36. ESI-HRMS (+) spectrum of compound **5**.

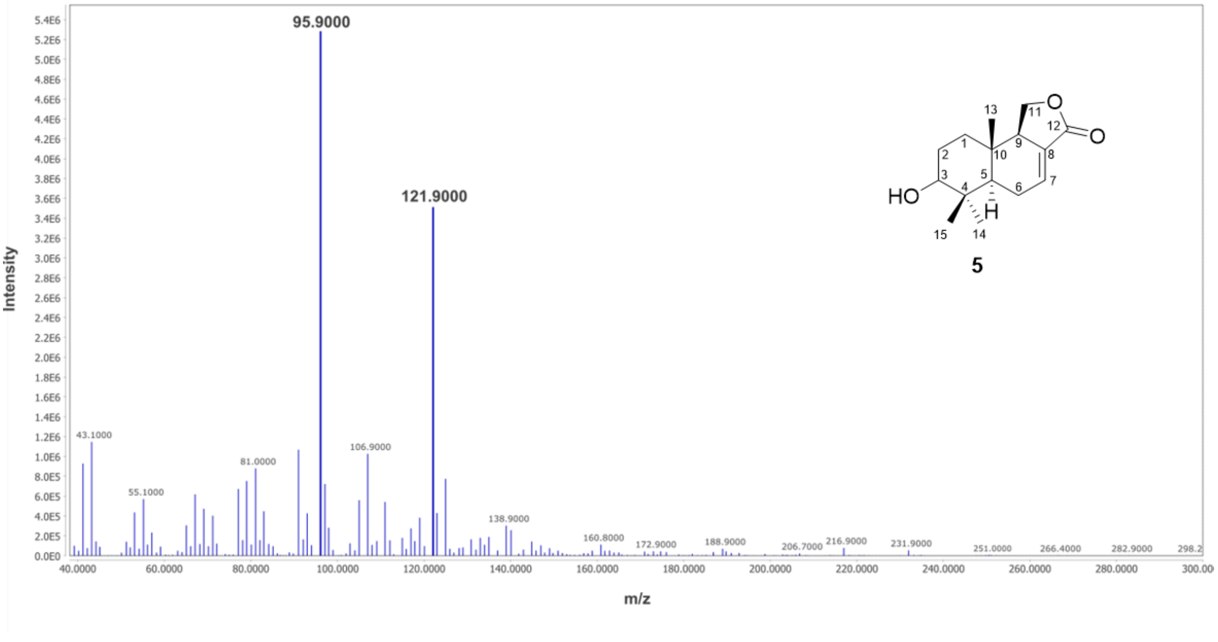

Figure S37. GC-MS spectrum of compound **5**.

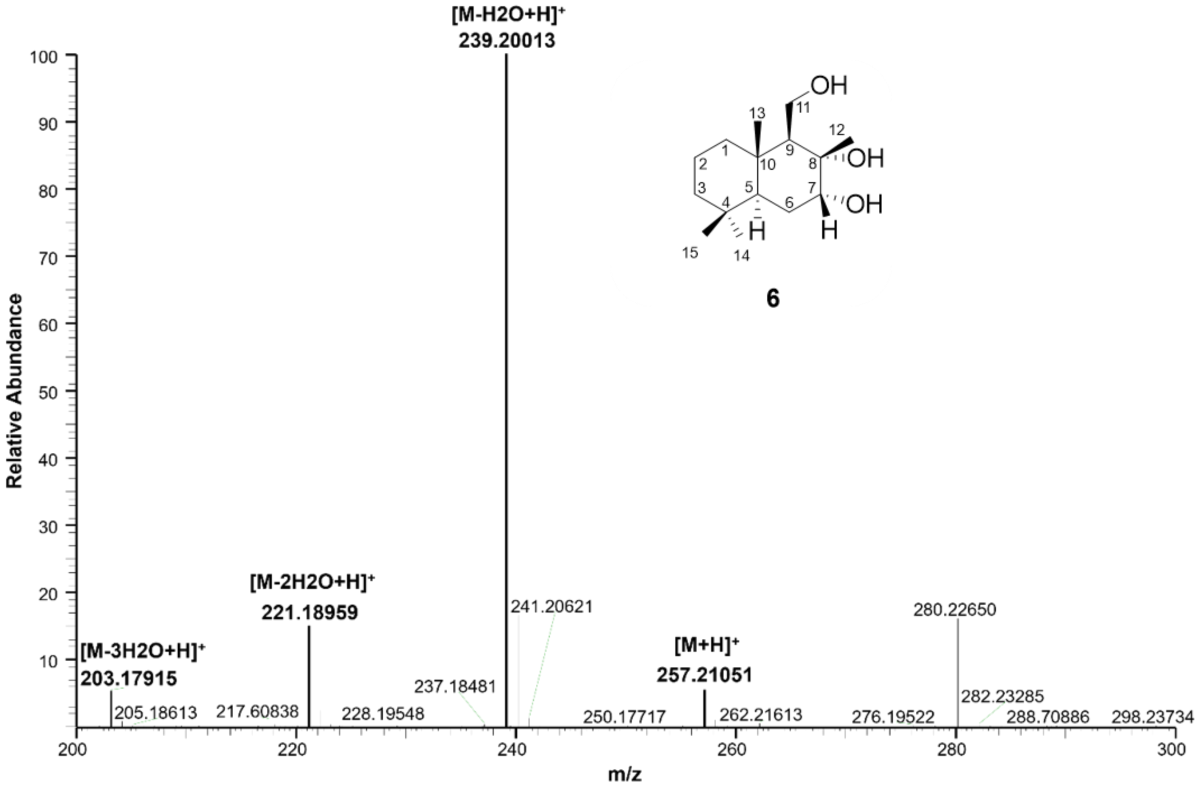

Figure S38. ESI-HRMS (+) spectrum of compound **6**.

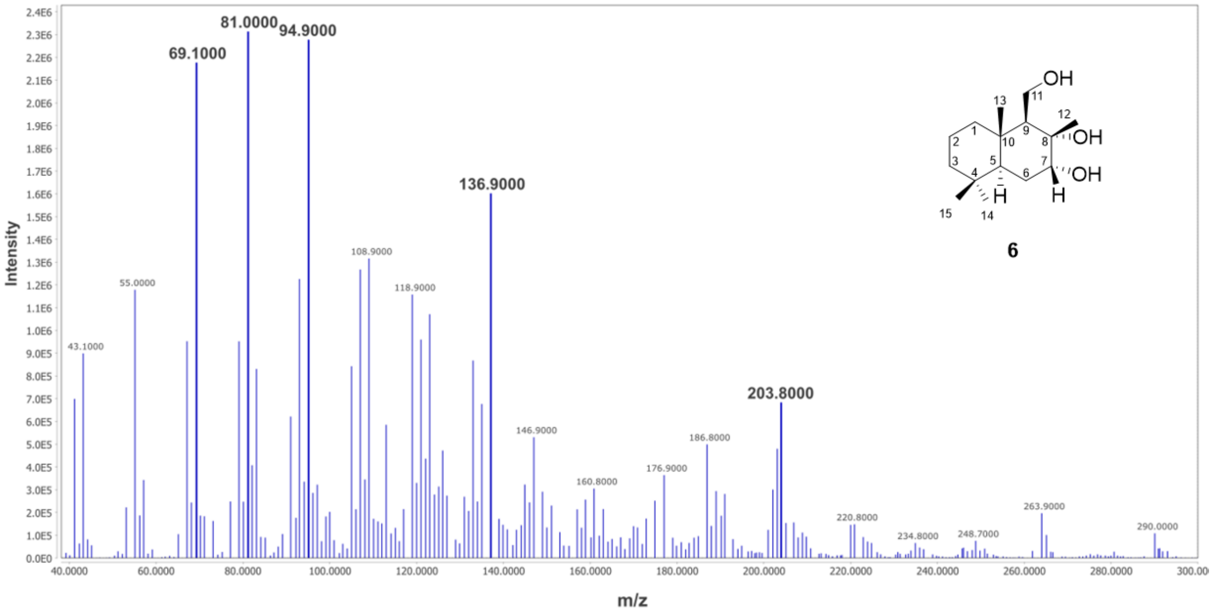

Figure S39. GC-MS spectrum of compound **6**.

12. Supplementary References

1 Skiredj A, Beniddir MA, Evanno L, Poupon E. Mimicking the Main Events of the Biosynthesis of Drimentines: Synthesis of ∆8'-Isodrimentine A and Related Compounds. Eur J Org Chem 2016(17):2954-2958. https://doi.org/10.1002/ejoc.201600444.

2 Hayakawa I, Nakamura T, Ohno O, Suenaga K, Kigoshi H. Synthesis and structure-activity relationships for cytotoxicity and apoptosis-inducing activity of (+)-halichonine B. Org Biomol Chem 2015, 13, 9969-9976; https://doi.org/10.1039/C5OB01488C.

3 Vlad PF, Aryku AN, Chokyrlan AG. Synthesis of (+)-drim-9(11)-en-8α-ol from sclareol. Russ Chem Bull 2004, 53, 443-446. https://doi.org/10.1023/b:rucb.0000030822.72251.ea.

4 Kamishima T, Kikuchi T, Narita K, Katoh T. 2014. Biogenetically Inspired Total Synthesis (+)-Liphagal: A Potent and Selective Phosphoinositide 3-Kinase α (PI3Kα) Inhibitor from the Marine Sponge Aka coralliphaga. Eur J Org Chem 2014, 3443-3450. https://doi.org/10.1002/ejoc.201402082.

5 Kuchkova KI, Aryku AN, Dragalin IP, Vlad PF. Synthesis of Drim-9(11)-en-8α- and -8β-ols from Drimenol. Chem Nat Compd 2005, 41, 190-193. https://doi.org/10.1007/s10600-005-0109-8.

6 Kikuchi T, Narita K, Saijo K, Ishioka C, Katoh T. 2016. Enantioselective Total Synthesis of (-)-Siphonodictyal B and (+)-8-epi-Siphonodictyal B with Phosphatidylinositol 3-Kinase α (PI3Kα) Inhibitory Activity. Eur J Org Chem 2016, 5659-5666. https://doi.org/10.1002/ejoc.201600949.

7 Göhl M, Seifert K. Synthesis of the Sesquiterpenes Albicanol, Drimanol and Drimanic Acid and the Marine Sesquiterpene Hydroquinone Deoxyspongiaquinol. Eur J Org Chem 2014, 31, 6975-6982. https://doi.org/10.1002/ejoc.201402873.

8 D'Acunto M, Monica CD, Izzo I, Petrocellis L, Marzo V, Spinella A. Enantioselective synthesis of 3-(*S*)-hydroxy polygodial derivatives and evaluation of their vanilloid activity. Tetrahedron 2010, 66, 9785-9789. https://doi.org/10.1016/j.tet.2010.11.002.

9 Phan, R. M.; Poulter, C. D., *J. Org. Chem.* 2001, 66, 20, 6705-6710.

10 Huynh, F.; Grundy, D. J.; Jenkins, R. L.; Miller, D. J.; Allemann, R.K. *ChemBioChem* 2018,19,1834 –1838

11 Xu D, Sheng Y, Zhou ZY, Liu R, Leng Y, Liu JK. Sesquiterpenes from cultures of the basidiomycete Clitocybe conglobata and their 11 beta-hydroxysteroid dehydrogenase inhibitory activity. Chem Pharm Bull (Tokyo). 2009, 57, 433-5. doi: 10.1248/cpb.57.433

12 Ayer WA, Craw P. Metabolites of the fairy ring fungus, *Marasmius oreades*. Part 2. Norsesquiterpenes, further sesquiterpenes and agrocybin. Can J Chem 1989, 67, 1371-1380. https://doi.org/10.1139/v89-210.

13 Aranda G, Facon I, Lallemand J-Y, Leclaire M, Azerad R, Cortes M, Lopez J, Ramirez H. Microbial hydroxylation in the drimane series. Tetrahedron Lett 1992, 33, 7845-7848. https://doi.org/10.1016/S0040-4039(00)74759-9.

14 Xu D, Sheng Y, Zhou Z-Y, Liu R, Leng Y, Liu J-K. Sesquiterpenes from cultures of the basidiomycete *Clitocybe conglobate* and their 11 beta-hydroxysteroid dehydrogenase inhibitory activity. Chem Pharm Bull 2009, 57, 433-435. https://doi.org/10.1248/cpb.57.433.

15 Zhao Z-Z, Chen H-P, Feng T, Li Z-H, Dong Z-J, Liu J-K. Four New Sesquiterpenoids from Cultures of the Fungus Phellinidium sulphurascens. Nat Prod Bioprospect 2014, 5, 23-28. https://doi.org/10.1007/s13659-014-0047-x.

16 Ayer WA, Trifonov LS. Drimane Sesquiterpene Lactones from Peniophora polygonia. J Nat Prod 1992, 55, 1454-1461. https://doi.org/10.1021/np50088a011.

17 Panasenko AA, Gorincioi EC, Aricu AN, Barcari EA, Deleanu K and Vlad PF. 2004. ^{1}H and ^{13}C NMR spectra of some drimanic sesquiterpenoids. Russ Chem Bull 53:2700-2705. https://doi.org/10.1007/s11172-005-0178-7.

18 G. D. Gamalevicha, V. N. Kulcitkib, N. D. Ungurb, P. F.Vladb, *Mendeleev Communications* 2002, 12, 59-60; https://doi.org/10.1070/MC2002v012n02ABEH001566

19 T. Kikuchi, K. Narita, K. Saijo, C. Ishioka, T. Katoh, Enantioselective Total Synthesis of (–)-Siphonodictyal B and (+)-8-epi-Siphonodictyal B with Phosphatidylinositol 3-Kinase α (PI3Kα) Inhibitory Activity, Eur. J. Org. Chem. 2016, 34, 5659-5666, https://doi.org/10.1002/ejoc.201600949

20 T. Kamishima, T. Kikuchi, K. Narita, T. Katoh, Biogenetically Inspired Total Synthesis of (+)-Liphagal: A Potent and Selective Phosphoinositide 3-Kinase α (PI3Kα) Inhibitor from the Marine Sponge Aka coralliphaga, Eur. J. Org. Chem. 2014, 16, 3443-3450 https://doi.org/10.1002/ejoc.201402082

21 Manuel González-Sierra, Maria de los Angeles Laborde, Edmundo A. Rúveda, Alternative and Stereoselective Synthesis of 8β(H)-Drimane, A Bicyclic Sesquiterpane of Widespread Occurrence in Petroleums, Synthetic Communications 1987, 17, 431-441, https://doi.org/10.1080/00397918708063921

22 Di Xu, Yu Sheng, Zhong-Yu Zhou, Rong Liu, Ying Leng, Ji-Kai Liu, Sesquiterpenes from Cultures of the Basidiomycete Clitocybe conglobata and Their 11β-Hydroxysteroid Dehydrogenase Inhibitory Activity, J-Stage 2009, 57, 433-435, https://doi.org/10.1248/cpb.57.433

23 Zhao, ZZ., Chen, HP., Feng, T. *et al.* Four New Sesquiterpenoids from Cultures of the Fungus *Phellinidium sulphurascens*. *Nat. Prod. Bioprospect.* 2015, **5**, 23–28 https://doi.org/10.1007/s13659-014-0047-x

24 Rabe P, Citron CA and Dickschat JS. Volatile Terpenes from Actinomycetes: A Biosynthetic Study Correlating Chemical Analyses to Genome Data. ChemBioChem 2013, 14, 2345-2354. https://doi.org/10.1002/cbic.201300329.

25 Rabe P and Dickschat JS. Rapid Chemical Characterization of Bacterial Terpene Synthases. Angew. Chem. Int. Ed. 2013, 52, 1810-1812. https://doi.org/10.1002/anie.201209103.

26 Quin MB, Flynn CM and Schmidt-Dannert C. Traversing the fungal terpenome. Nat. Prod. Rep. 2014, 31, 1449-1473. https://doi.org/10.1039/C4NP00075G.

27 Tang X, Allemann RK and Wirth T, Optimising Terpene Synthesis with Flow Biocatalysis. Eur. J. Org. Chem. 2017, 414-418.
https://doi.org/10.1002/ejoc.201601388.

28 Sato H, Hashishin T, Kanazawa J, Miyamoto K and Uchiyama M. DFT Study of a Missing Piece in Brasilane-Type Structure Biosynthesis: An Unusual Skeletal Rearrangement. J. Am. Chem. Soc. 2020, 142, 19830-19834.
https://doi.org/10.1021/jacs.0c09616.

29 Martin DM, Aubourg, S, Schouwey, MB et al. Functional Annotation, Genome Organization and Phylogeny of the Grapevine (Vitis vinifera) Terpene Synthase Gene Family Based on Genome Assembly, FLcDNA Cloning, and Enzyme Assays. BMC Plant Biol 2010, 10, 226. https://doi.org/10.1186/1471-2229-10-226.

30 López-Gallego F, Wawrzyn GT, Schmidt-Dannert C. Selectivity of Fungal Sesquiterpene Synthases: Role of the Active Site's H-1α Loop in Catalysis. Appl. Environ. Microbiol. 2010, 76. https://doi.org/10.1128/AEM.01811-10

31 Shinohara Y, Takahashi S, Osada H, Koyama Y. Identification of a novel sesquiterpene biosynthetic machinery involved in astellolide biosynthesis. Sci Rep. 2016, 6, 32865.
doi: 10.1038/srep32865.

32 Kreuzenbeck NB, Seibel E, Schwitalla JW, Fricke J, Conlon BH, Schmidt S, Hammerbacher A, Köllner TG, Poulsen M, Hoffmeister D, Beemelmanns C. Comparative Genomic and Metabolomic Analysis of *Termitomyces* Species Provides Insights into the Terpenome of the Fungal Cultivar and the Characteristic Odor of the Fungus Garden of *Macrotermes natalensis* Termites. mSystems 2022, 11, e0121421.
doi: 10.1128/msystems.01214-21.

33 Burkhardt I, Kreuzenbeck NB, Beemelmanns C, Dickschat JS. Mechanistic characterization of three sesquiterpene synthases from the termite-associated fungus *Termitomyces*. Org Biomol Chem 2019, 17, 3348-3355.
https://doi.org/10.1039/C8OB02744G.

34 Zhang Q, Rinkel J, Goldfuss B. et al. Sesquiterpene cyclizations catalysed inside the resorcinarene capsule and application in the short synthesis of isolongifolene and isolongifolenone. Nat Catal 2018,1, 609–615.
https://doi.org/10.1038/s41929-018-0115-4.

35 Lin X and Cane DE. Biosynthesis of the Sesquiterpene Antibiotic Albaflavenone in *Streptomyces coelicolor*. Mechanism and Stereochemistry of the Enzymatic Formation of Epi-isozizaene. J. Am. Chem. Soc. 2009, 131, 6332-6333.
https://doi.org/10.1021/ja901313v.

36 R. R. da Costa, H. Hu, B. Pilgaard, S. M. E. Vreeburg, J. Schückel, K. S. K. Pedersen, S. K. Kračun, P. K. Busk, J. Harholt, P. Sapountzis, L. Lange, D. K. Aanen, M. Poulsen, *Appl. Environ. Microbiol.* 2018, *84*, e01815-17.

37 N. B. Kreuzenbeck, E. Seibel, J. W. Schwitalla, J. Fricke, B. H. Conlon, S. Schmidt, A. Hammerbacher, T. G. Köllner, M. Poulsen, D. Hoffmeister, C. Beemelmanns, *mSystems* 2022, *7*, e01214-0121421.

38 Á. Proszenyák, M. Brændvang, C. Charnock, L. L. Gundersen, *Tetrahedron* 2009, *65*, 1

39 A. Fernández-Mateos, S. Encinas Madrazo, P. Herrero Teijón, R. Rabanedo Clemente, R. Rubio González, and F. Sanz González *J. Org. Chem.* 2013 *78*, 9571-9578
