# Supplementary material for: Isolation, (bio)synthetic studies and evaluation of antimicrobial properties of drimenol-type sesquiterpenes of Termitomyces fungi
Source: Commun Chem. 2023 Apr 24;6:79. doi: 10.1038/s42004-023-00871-z (PMC10126200; doi:10.1038/s42004-023-00871-z)
Supplement: Supplementary file 3 — Description of Additional Supplementary Files [file 42004_2023_871_MOESM3_ESM.pdf]

Description of Additional Supplementary Files

File name: Supplementary Data 1

Description: copies of all NMR spectra

File name: Supplementary Data 2

Description: Crystallographic Information File (CIF) for structure of compound 2.
