# Supplementary material for: Isolation, (bio)synthetic studies and evaluation of antimicrobial properties of drimenol-type sesquiterpenes of Termitomyces fungi
Source: Commun Chem. 2023 Apr 24;6:79. doi: 10.1038/s42004-023-00871-z (PMC10126200; doi:10.1038/s42004-023-00871-z)
Supplement: Supplementary file 4 — Supplementary Data 1 [file 42004_2023_871_MOESM4_ESM.pdf]

Supplementary Data 1

Isolation, (bio)synthetic studies and evaluation of antimicrobial properties of drimenol-type sesquiterpenes of *Termitomyces* fungi

Nina B. Kreuzenbeck,[1] Seema Dhiman,[2] Dávid Roman,[1] Immo Burkhardt,[3] Benjamin H. Conlon,[4] Janis Fricke,[1] Huijuan Guo,[1] Janis Blume,[2] Helmar Görls,[5] Michael Poulsen,[4] Jeroen S. Dickschat,[3] Tobias G. Köllner,[6] Hans-Dieter Arndt,[2] and Christine Beemelmanns*[1,7,8]

[1] N. B. Kreuzenbeck, D. Roman, J. Fricke, H. Guo, C. Beemelmanns,
Chemical Biology of Microbe-Host Interactions
Leibniz Institute for Natural Product Research and Infection Biology – Hans Knöll-Institute (HKI),
Beutenbergstraße 11a, 07745, Jena, Germany,

[2] S. Dhiman, J. Blume, H.-D. Arndt
Institute for Organic and Macromolecular Chemistry
Friedrich-Schiller-University,
Humboldtstr. 10, 07743 Jena, Germany

[3] I. Burkhardt, J. S. Dickschat
Kekulé-Institute of Organic Chemistry and Biochemistry
University of Bonn, Gerhard-Domagk-Straße 1
53121 Bonn, Germany Department

[4] B. H. Conlon, M. Poulsen
Section for Ecology and Evolution, Department of Biology,
University of Copenhagen, Universitetsparken 15
2100 Copenhagen, Denmark

[5] H. Görls
Institute for Inorganic and Analytical Chemistry
Friedrich-Schiller University
07743 Jena, Germany

[6] T. G. Köllner
Max Planck Institute for Chemical Ecology
Department of Natural Product Biosynthesis
Hans-Knöll-Straße 8, 07745 Jena

[7] C. Beemelmanns
Helmholtz-Institut für Pharmazeutische Forschung Saarland (HIPS),
Helmholtz Zentrum für Infektionsforschung (HZI),
Campus E8, 66123 Saarbrücken, Germany,

[8] Universität des Saarlandes, Campus E8, 66123 Saarbrücken, Germany,
E-mail: Christine.Beemelmanns@helmholtz-hips.de

Table of Figures

Figure NMR-S1. ^{1}H NMR spectrum of compound ((1S,8aS)-2,5,5,8a-tetramethyl-1,4,4a,5,6,7,8,8a-octahydronaphthalen-1-yl)methanol (**1**). 6
Figure NMR-S2. ^{13}C NMR spectrum of synthetic compound **1**. 7
Figure NMR-S3. ^{1}H NMR spectrum of compound **2** ($CDCl_3$, 300 K, 600 MHz). 8
Figure NMR-S4. ^{13}C NMR spectrum of compound **2** ($CDCl_3$, 300 K, 150 MHz). 9
Figure NMR-S5. ^{13}C NMR DEPT spectrum of compound **2** ($CDCl_3$, 300 K, 150 MHz). 10
Figure NMR-6. COSY spectrum of compound **2** ($CDCl_3$, 300 K, 600 MHz). 11
Figure NMR-S7. HSQC spectrum of compound **2** ($CDCl_3$, 300 K, 600 MHz). 12
Figure NMR-S8. HMBC spectrum of compound **2** ($CDCl_3$, 300 K, 600 MHz). 13
Figure NMR-S9. TOCSY spectrum of compound **2** ($CDCl_3$, 300 K, 600 MHz). 14
Figure NMR-S10. NOESY spectrum of compound **2** ($CDCl_3$, 300 K, 600 MHz). 15
Figure NMR-S11. ^{1}H NMR spectrum of compound **3** ($CDCl_3$, 300 K, 600 MHz). 16
Figure NMR-S12. ^{13}C NMR spectrum of compound **3** ($CDCl_3$, 300 K, 150 MHz) 17
Figure NMR-S13. COSY spectrum of compound **3** ($CDCl_3$, 300 K, 600 MHz). 18
Figure NMR-S14. HSQC spectrum of compound **3** ($CDCl_3$, 300 K, 500 MHz). 19
Figure NMR-S15. HMBC spectrum of compound **3** ($CDCl_3$, 300 K, 500 MHz). 20
Figure NMR-S16. Selective NOE spectrum of compound **3** at 1.100 ppm ($CDCl_3$, 300 K, 500 MHz). 21
Figure NMR-S17. Selective NOE spectrum of compound **3** at 1.06 ppm ($CDCl_3$, 300 K, 500 MHz). 22
Figure NMR-S18. Selective NOE spectrum of compound **3** at 1.61 ppm ($CDCl_3$, 300 K, 500 MHz). 23
Figure NMR-S19. ^{1}H NMR spectrum of compound **4** ($CDCl_3$, 300 K, 600 MHz). 24
Figure NMR-S20. ^{13}C NMR spectrum of compound **4** ($CDCl_3$, 300 K, 150 MHz) 25
Figure NMR-S21. ^{13}C NMR DEPT spectrum of compound **4** ($CDCl_3$, 300 K, 150 MHz). 26
Figure NMR-S22. COSY spectrum of compound **4** ($CDCl_3$, 300 K, 600 MHz). 27
Figure NMR-S23. HSQC spectrum of compound **4** ($CDCl_3$, 300 K, 600 MHz). 28
Figure NMR-S24. HMBC spectrum of compound **4** ($CDCl_3$, 300 K, 600 MHz). 29
Figure NMR-S25. TOCSY spectrum of compound **4** ($CDCl_3$, 300 K, 600 MHz). 30
Figure NMR-S26. Selective NOE spectrum of compound **4** at 1.68 ppm ($CDCl_3$, 300 K, 600 MHz). 31
Figure NMR-S27. Selective NOE spectrum of compound **4** at 2.27 ppm ($CDCl_3$, 300 K, 600 MHz). 32
Figure NMR-S28. Selective NOE spectrum of compound **4** at 1.11 ppm ($CDCl_3$, 300 K, 600 MHz). 33

Figure NMR-S29. ^{1}H NMR spectrum of compound **5** ($CDCl_3$, 300 K, 600 MHz). 34
Figure NMR-S30. ^{13}C NMR spectrum of compound **5** ($CDCl_3$, 300 K, 150 MHz).................35
Figure NMR-S31. ^{13}C NMR DEPT spectrum of compound **5** ($CDCl_3$, 300 K, 150 MHz). 36
Figure NMR-S32. COSY spectrum of compound **5** ($CDCl_3$, 300 K, 600 MHz).....................37
Figure NMR-S33. HSQC spectrum of compound **5** ($CDCl_3$, 300 K, 600 MHz). 38
Figure NMR-S34. HMBC spectrum of compound **5** ($CDCl_3$, 300 K, 600 MHz). 39
Figure NMR-S35. Selective NOE spectrum of compound **5** at 1.38 ppm ($CDCl_3$, 300 K, 500 MHz). 40
Figure NMR-S36. Selective NOE spectrum of compound **5** at 2.79 ppm ($CDCl_3$, 300 K, 500 MHz). 41
Figure NMR-S37. Selective NOE spectrum of compound **5** at 3.31 ppm ($CDCl_3$, 300 K, 500 MHz). 42
Figure NMR-S38. ^{1}H NMR spectrum of compound **6** ($CDCl_3$, 300 K, 600 MHz). 43
Figure NMR-S39. ^{13}C NMR spectrum of compound **6** ($CDCl_3$, 300 K, 150 MHz).................44
Figure NMR-S40. ^{13}C NMR DEPT spectrum of compound **6** ($CDCl_3$, 300 K, 150 MHz). 45
Figure NMR-S41. COSY spectrum of compound **6** ($CDCl_3$, 300 K, 600 MHz).....................46
Figure NMR-S42. HSQC spectrum of compound **6** ($CDCl_3$, 300 K, 600 MHz). 47
Figure NMR-S43. HMBC spectrum of compound **6** ($CDCl_3$, 300 K, 600 MHz). 48
Figure NMR-S44. Selective NOE spectrum of compound **6** at 0.89 ppm ($CDCl_3$, 300 K, 500 MHz). 49
Figure NMR-S45. Selective NOE spectrum of compound **6** at 0.85 ppm ($CDCl_3$, 300 K, 500 MHz). 50
Figure NMR-S46. Selective NOE spectrum of compound **6** at 0.80 ppm ($CDCl_3$, 300 K, 500 MHz). 51
Figure NMR-S47. Selective NOE spectrum of compound **6** at 1.31 ppm ($CDCl_3$, 300 K, 500 MHz). 52
Figure NMR-S48. Selective NOE spectrum of compound **6** at 1.61 ppm ($CDCl_3$, 300 K, 500 MHz). 53
Figure NMR-S49. Selective NOE spectrum of compound **6** at 1.96 ppm ($CDCl_3$, 300 K, 500 MHz). 54
Figure NMR-S50. ^{1}H NMR spectrum of compound ((1S,2R,8aS)-2-hydroxy-2,5,5,8a-tetramethyldecahydronaphthalen-1-yl)methyl acetate (**8**). 55
Figure NMR-S51. ^{13}C NMR spectrum of compound **8**...56
Figure NMR-S52. ^{1}H NMR spectrum of compound (1S,2R,8aS)-1-(hydroxymethyl)-2,5,5,8a-tetramethyldecahydronaphthalen-2-ol (**9**). 57
Figure NMR-S53. ^{13}C NMR spectrum of compound **9**...58

Figure NMR-S54. ^{1}H NMR spectrum of compound (4aS)-1,1,4a,6-tetramethyl-5-methylene-1,2,3,4,4a,5,8,8a-octahydronaphthalene (**10**).........59
Figure NMR-S55. ^{13}C NMR spectrum of compound **10**.........60
Figure NMR-S56. ^{1}H NMR spectrum of compound (1S,8aS)-2,5,5,8a-tetramethyl-1,4,4a,5,6,7,8,8a-octahydronaphthalene-1-carbaldehyde (**11**).........61
Figure NMR-S57. ^{13}C NMR spectrum of compound **11**.........62
Figure NMR-S58. ^{1}H NMR spectrum of compound ((1S,2S,8aS)-2,5,5,8a-tetramethyldecahydronaphthalen-1-yl)methanol (**12**).........63
Figure NMR-S59. ^{13}C NMR spectrum of compound **12**.........64
Figure NMR-S60. ^{1}H NMR spectrum of compound (R,1E,5E)-9,10-dihydroxy-2,6,10-trimethylundeca-1,5-dien-1-yl acetate (**14**).........65
Figure NMR-S61. ^{13}C NMR spectrum of compound **14**.........66
Figure NMR-S62. ^{1}H NMR spectrum of compound (2E,6E)-9-((S)-3,3-dimethyloxiran-2-yl)-3,7-dimethylnona-2,6-dien-1-yl acetate (**15**).........67
Figure NMR-S63. ^{13}C NMR spectrum of compound **15**.........68
Figure NMR-S64. ^{1}H NMR spectrum of compound ((1S,4aR,6S,8aS)-6-hydroxy-5,5,8a-trimethyl-2-methylenedecahydronaphthalen-1-yl)methyl acetate (**16**).........69
Figure NMR-S65. ^{13}C NMR spectrum of compound **16**.........70
Figure NMR-S66. ^{1}H NMR spectrum of compound (2S,4aS,5S,6S,8aR)-5-(hydroxymethyl)-1,1,4a,6-tetramethyldecahydronaphthalen-2-ol (**18**).........71
Figure NMR-S67. ^{13}C NMR spectrum of compound **18**.........72
Figure NMR-S68. ^{1}H NMR spectrum of compound (4aS,5S,6S,8aR)-5-(hydroxymethyl)-1,1,4a,6-tetramethyloctahydronaphthalen-2(1H)-one (**19**).........73
Figure NMR-S69. ^{13}C NMR spectrum of compound **19**.........74
Figure NMR-S70. ^{1}H NMR spectrum of **20** (D_2O, 500 MHz).........75
Figure NMR-S71. ^{13}C NMR spectrum of **20** (D_2O, 125 MHz).........76
Figure NMR-S72. ^{1}H NMR spectrum of **21** (D_2O, 500 MHz).........77
Figure NMR-S73. ^{1}H NMR spectrum of compound **22** ($CDCl_3$, 300 K, 600 MHz).........78
Figure NMR-S74. ^{13}C NMR spectrum of compound **22**.........79
Figure NMR-S75. ^{13}C NMR DEPT spectrum of compound **22** ($CDCl_3$, 300 K, 150 MHz).........80
Figure NMR-S76. COSY spectrum of compound **22** ($CDCl_3$, 300 K, 600 MHz).........81
Figure NMR-S77. HSQC spectrum of compound **22** ($CDCl_3$, 300 K, 600 MHz).........82
Figure NMR-S78. HMBC spectrum of compound **22** ($CDCl_3$, 300 K, 600 MHz).........83
Figure NMR-S79. ^{1}H NMR spectrum of compound **23** ($CDCl_3$, 300 K, 600 MHz).........84
Figure NMR-S80. ^{13}C NMR spectrum of compound **23**.........85
Figure NMR-S81. ^{13}C NMR DEPT spectrum of compound **23** ($CDCl_3$, 300 K, 150 MHz).........86
Figure NMR-S82. COSY spectrum of compound **23** ($CDCl_3$, 300 K, 600 MHz).........87

Figure NMR-S83. HSQC spectrum of compound **23** ($CDCl_3$, 300 K, 600 MHz). 88
Figure NMR-S84. HMBC spectrum of compound **23** ($CDCl_3$, 300 K, 600 MHz). 89
Figure NMR-S85. ^{1}H NMR spectrum of **25** ($CDCl_3$, 300 MHz). 90
Figure NMR-S86. ^{13}C NMR spectrum of **25** ($CDCl_3$, 75 MHz). 91
Figure NMR-S87. ^{1}H NMR spectrum of **27** ($CDCl_3$, 300 MHz). 92
Figure NMR-S88. ^{13}C NMR spectrum of **27** ($CDCl_3$, 75 MHz). 93
Figure NMR-S89. ^{1}H NMR spectrum of compound 1-((1R,2R,8aS)-2-hydroxy-2,5,5,8a-tetramethyldecahydronaphthalen-1-yl)propan-2-one (**28**). 94
Figure NMR-S90. ^{13}C NMR spectrum of compound **28**. 95
Figure NMR-S91. ^{1}H NMR spectrum of compound (2E,6E)-9-((S)-3,3-dimethyloxiran-2-yl)-3,7-dimethylnona-2,6-dien-1-ol (**29**)...96
Figure NMR-S92. ^{13}C NMR spectrum of compound **29**. 97

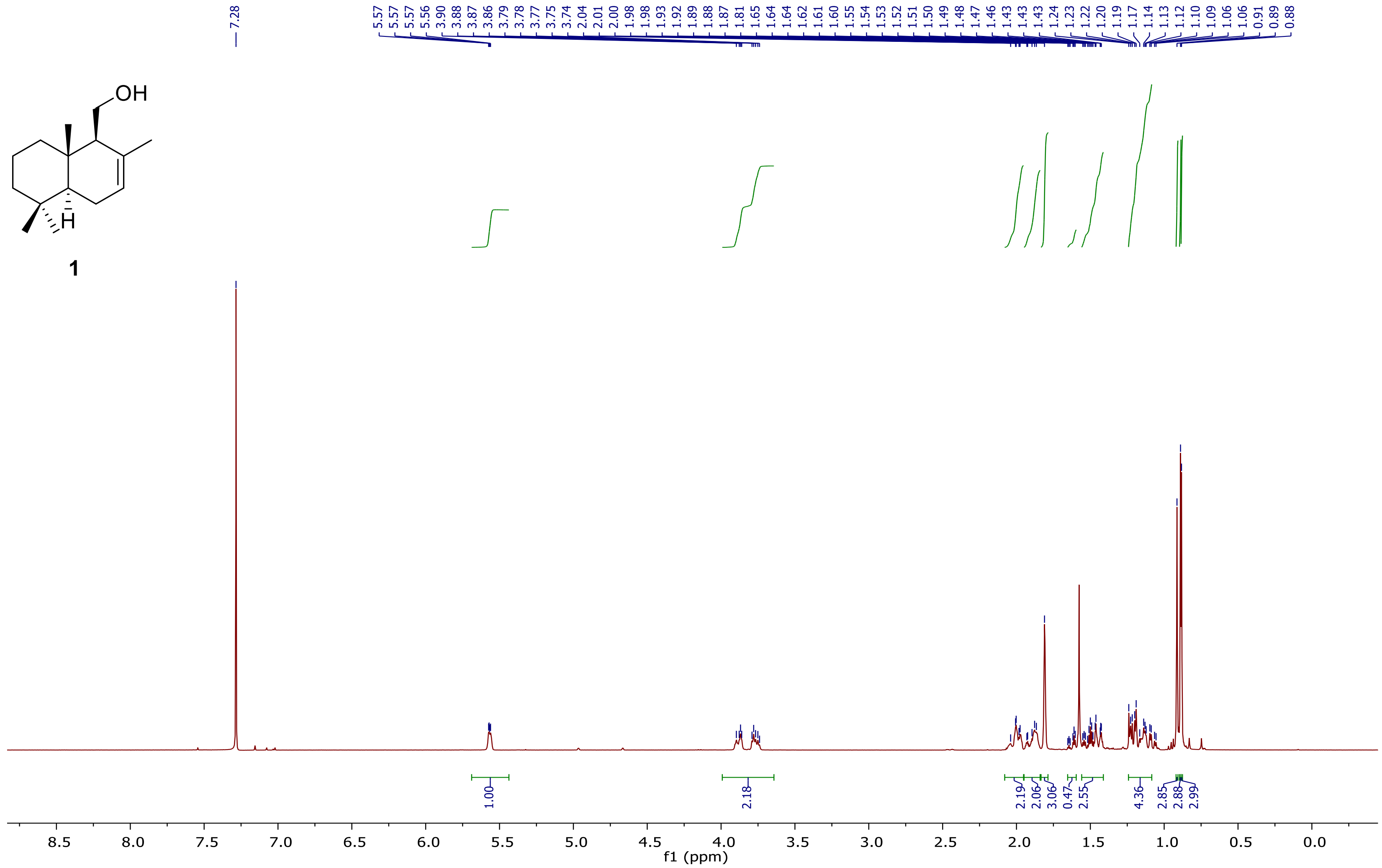

Figure NMR-S1. ^{1}H NMR spectrum of compound ((1*S*,8a*S*)-2,5,5,8a-tetramethyl-1,4,4a,5,6,7,8,8a-octahydronaphthalen-1-yl)methanol (**1**).

Supplementary Data 1

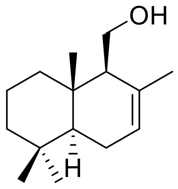

1

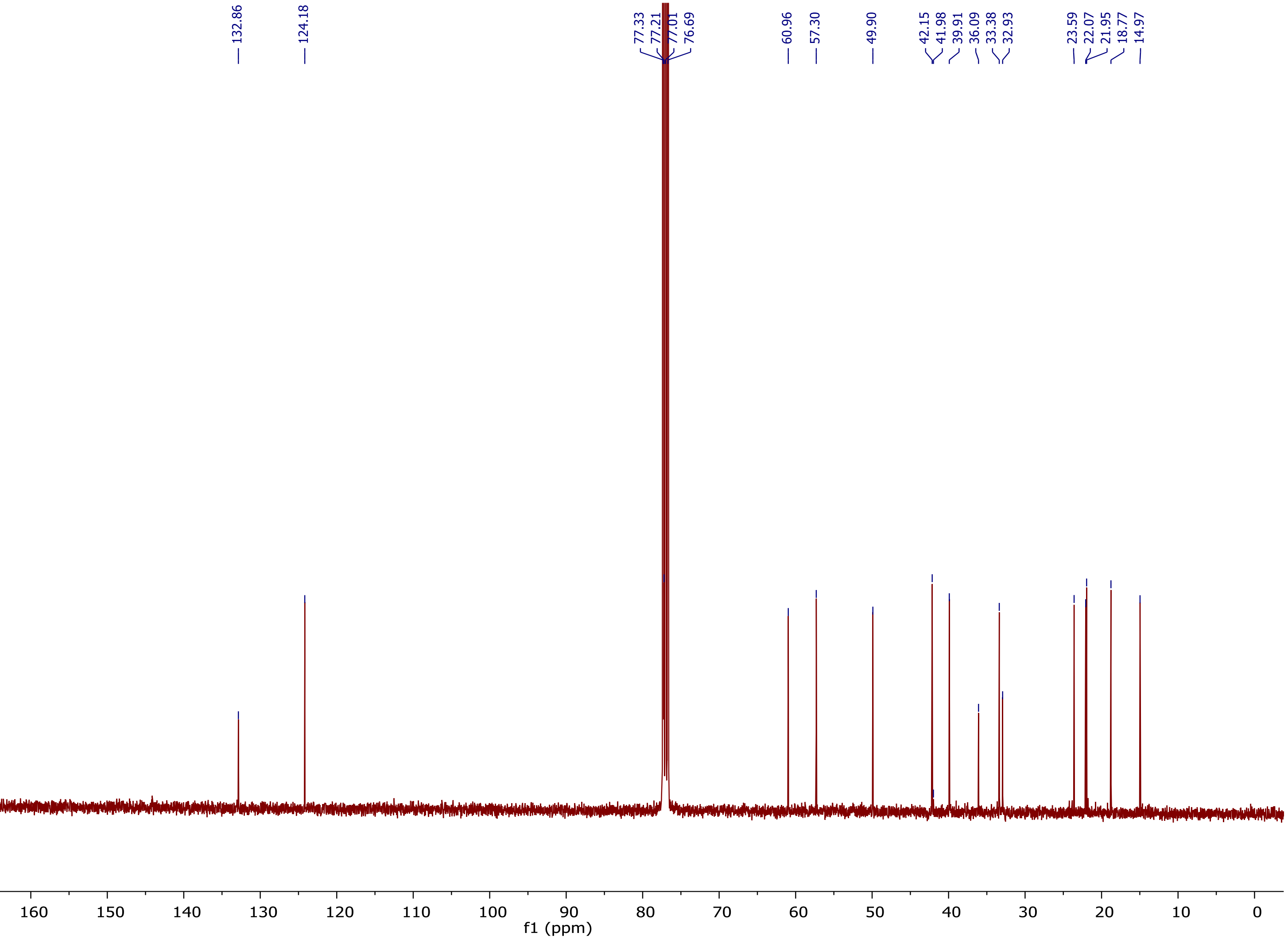

Figure NMR-S2. ^{13}C NMR spectrum of synthetic compound **1**.

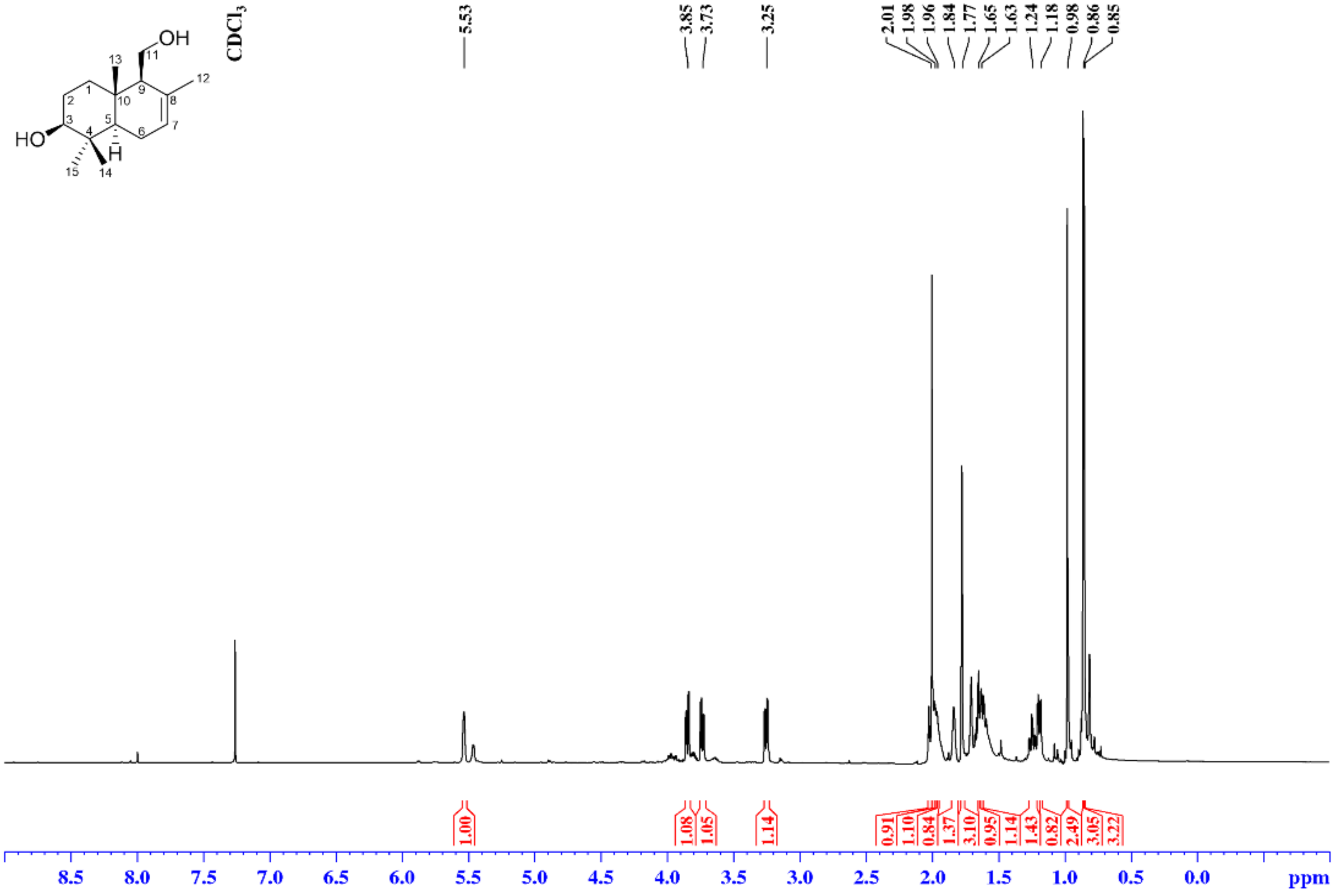

Figure NMR-S3. ^{1}H NMR spectrum of compound **2** ($CDCl_3$, 300 K, 600 MHz).

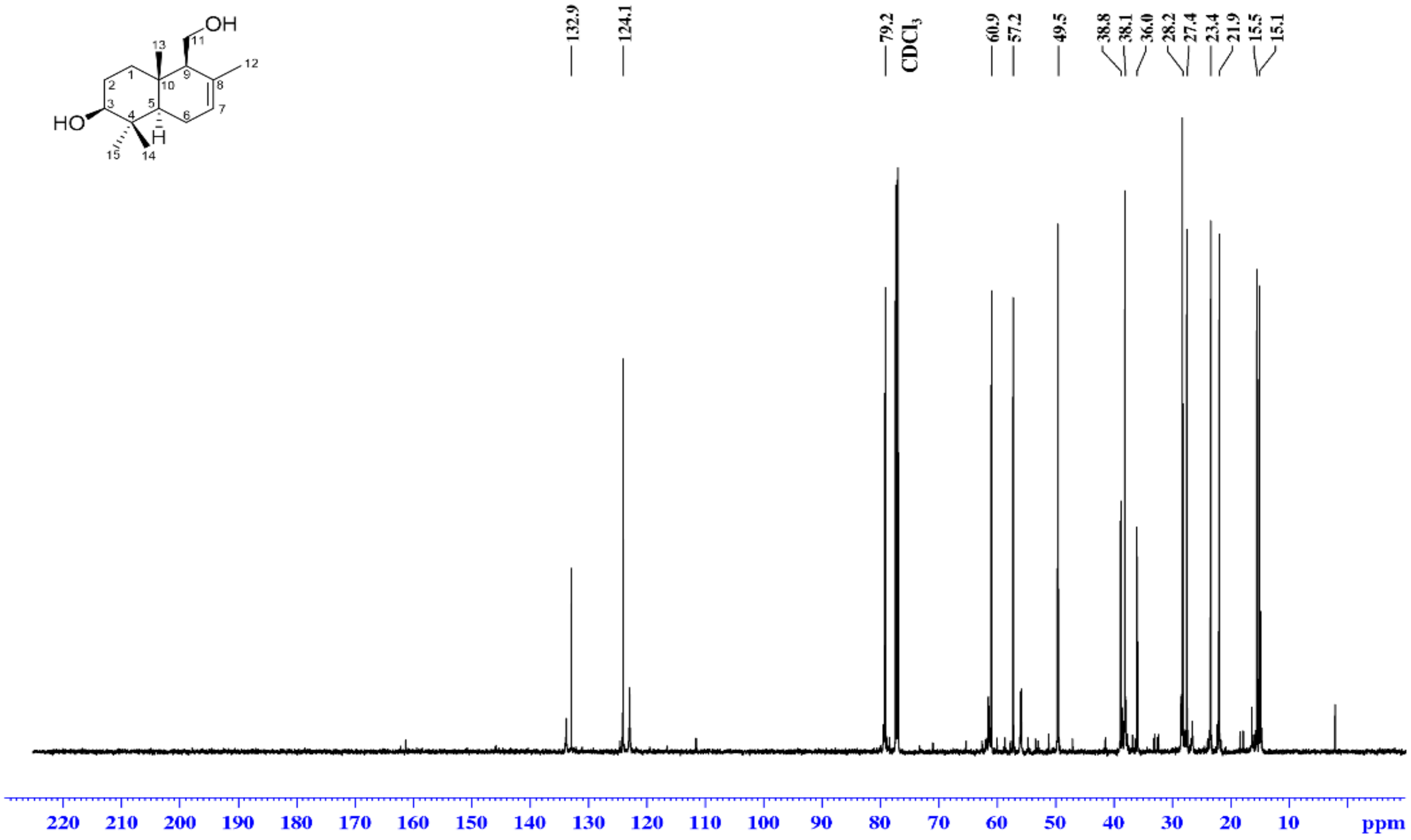

Figure NMR-S4. ^{13}C NMR spectrum of compound **2** ($CDCl_3$, 300 K, 150 MHz).

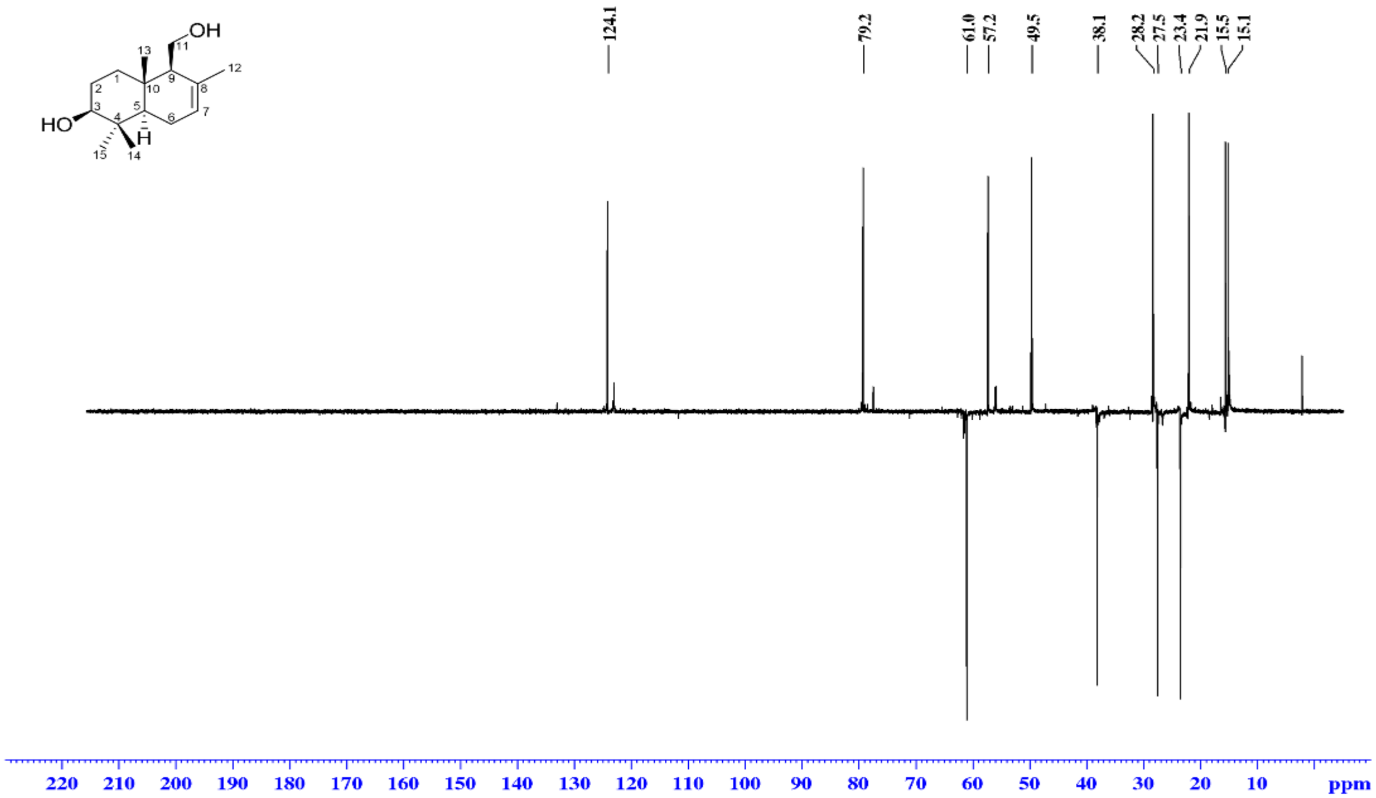

Figure NMR-S5. ^{13}C NMR DEPT spectrum of compound **2** ($CDCl_3$, 300 K, 150 MHz).

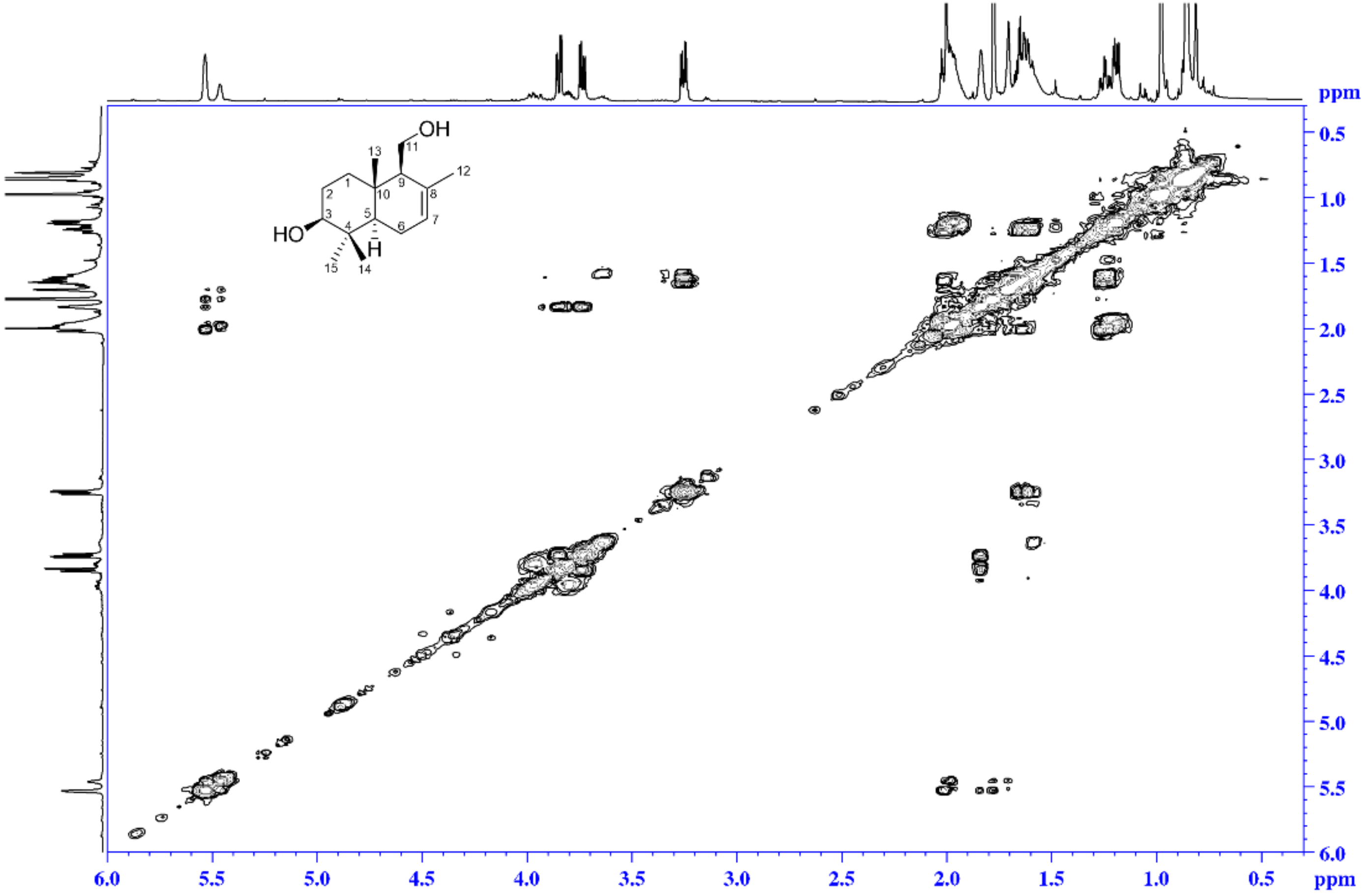

Figure NMR-6. COSY spectrum of compound **2** ($CDCl_3$, 300 K, 600 MHz).

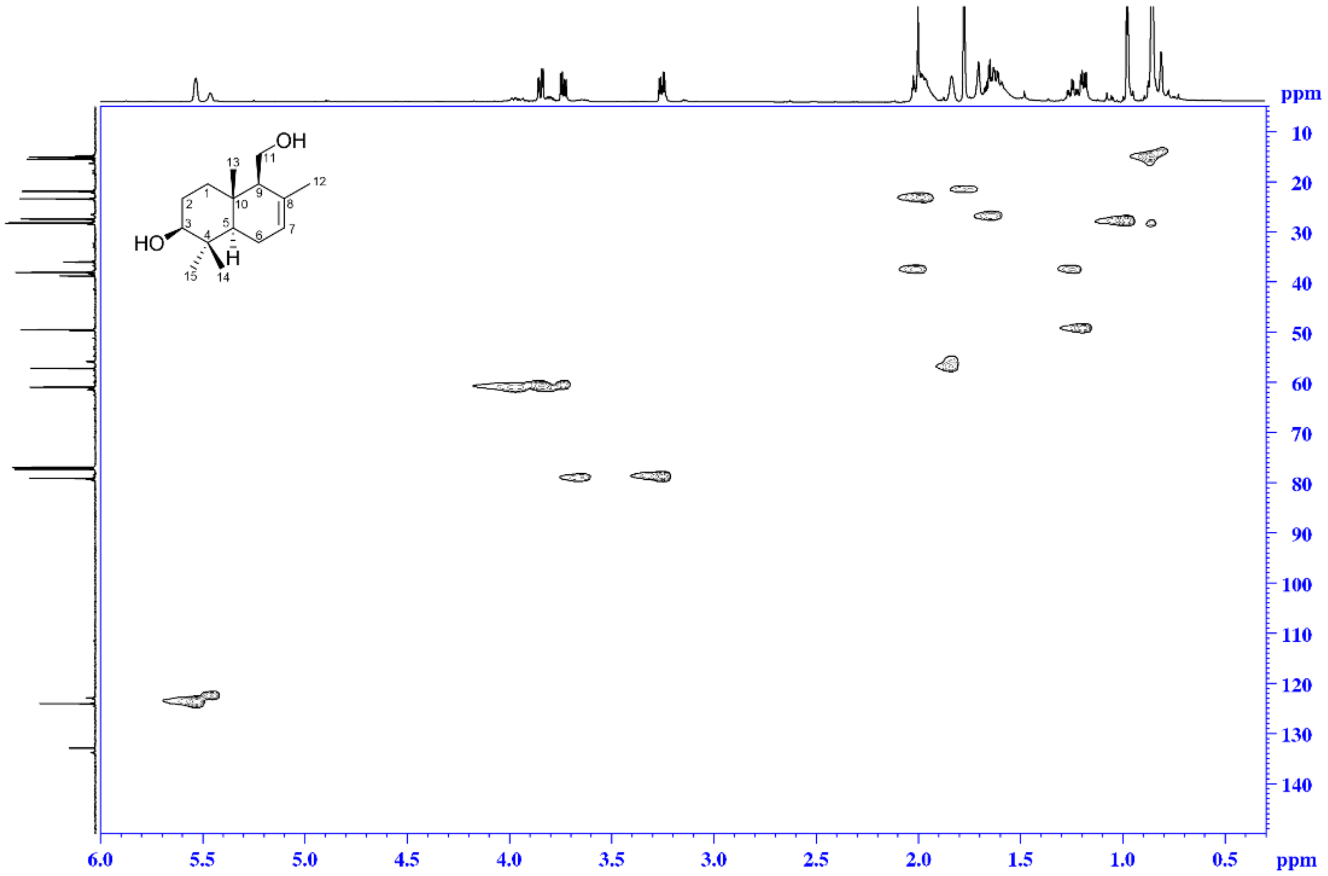

Figure NMR-S7. HSQC spectrum of compound **2** ($CDCl_3$, 300 K, 600 MHz).

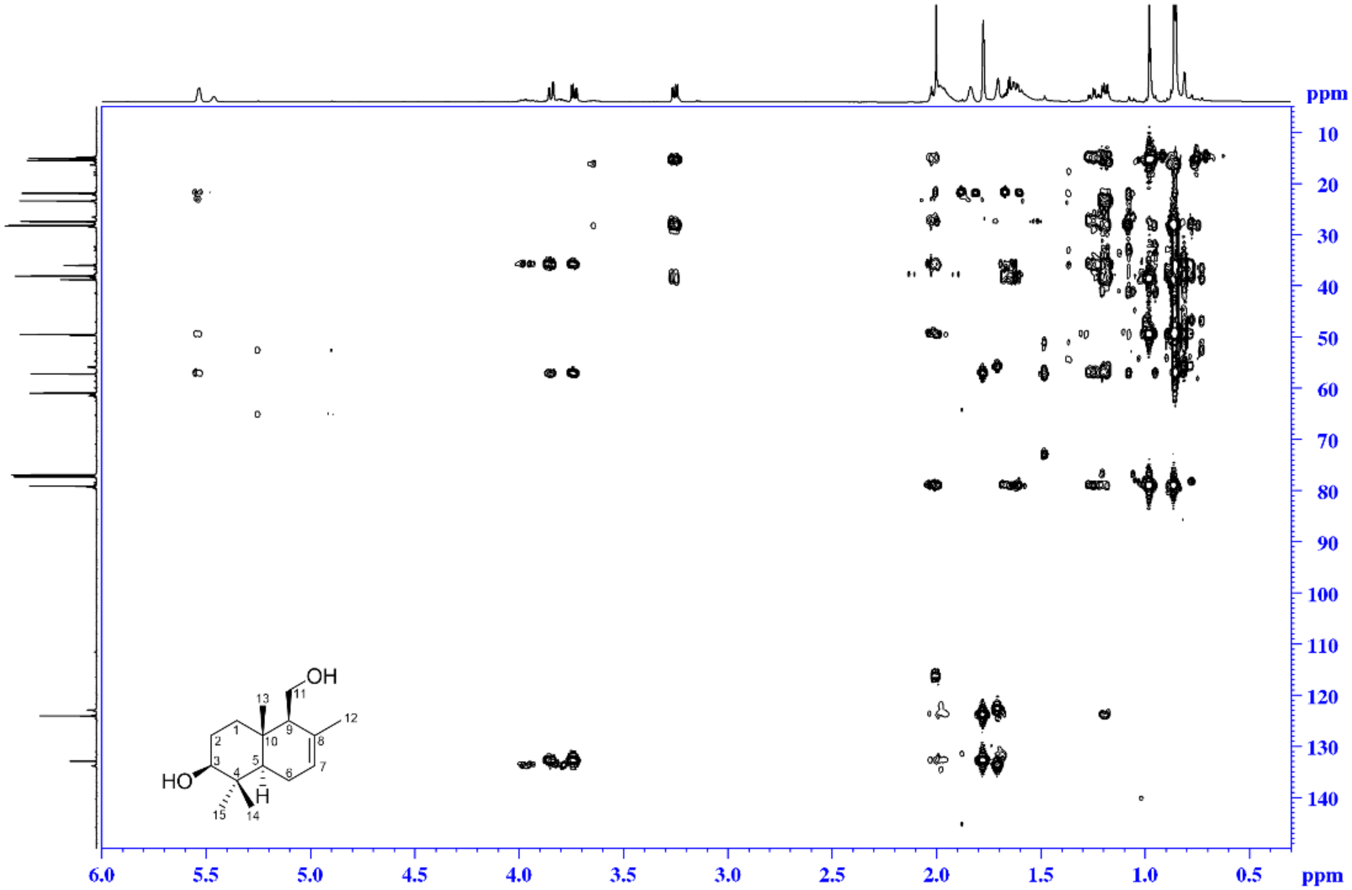

Figure NMR-S8. HMBC spectrum of compound **2** ($CDCl_3$, 300 K, 600 MHz).

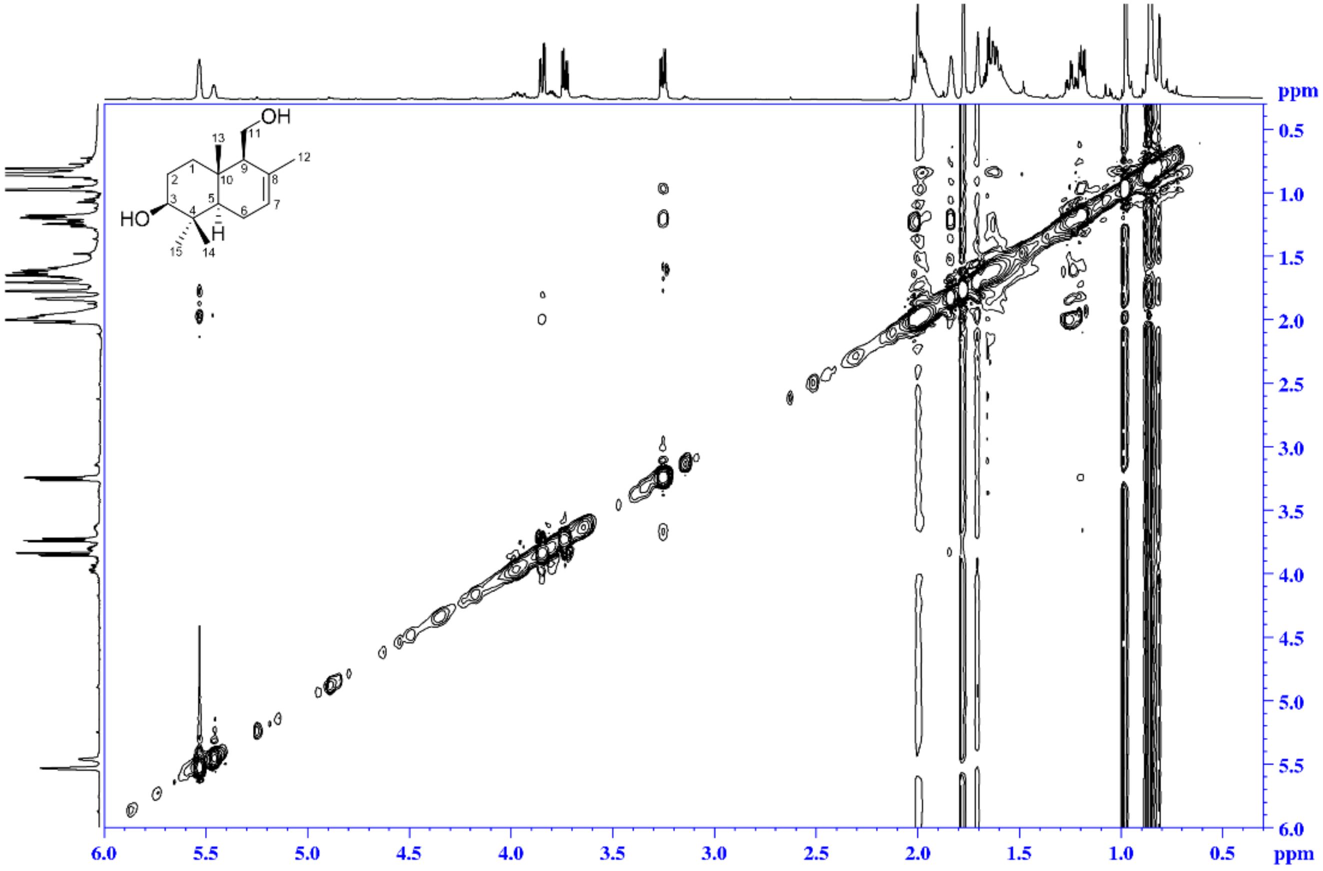

Figure NMR-S9. TOCSY spectrum of compound **2** ($CDCl_3$, 300 K, 600 MHz).

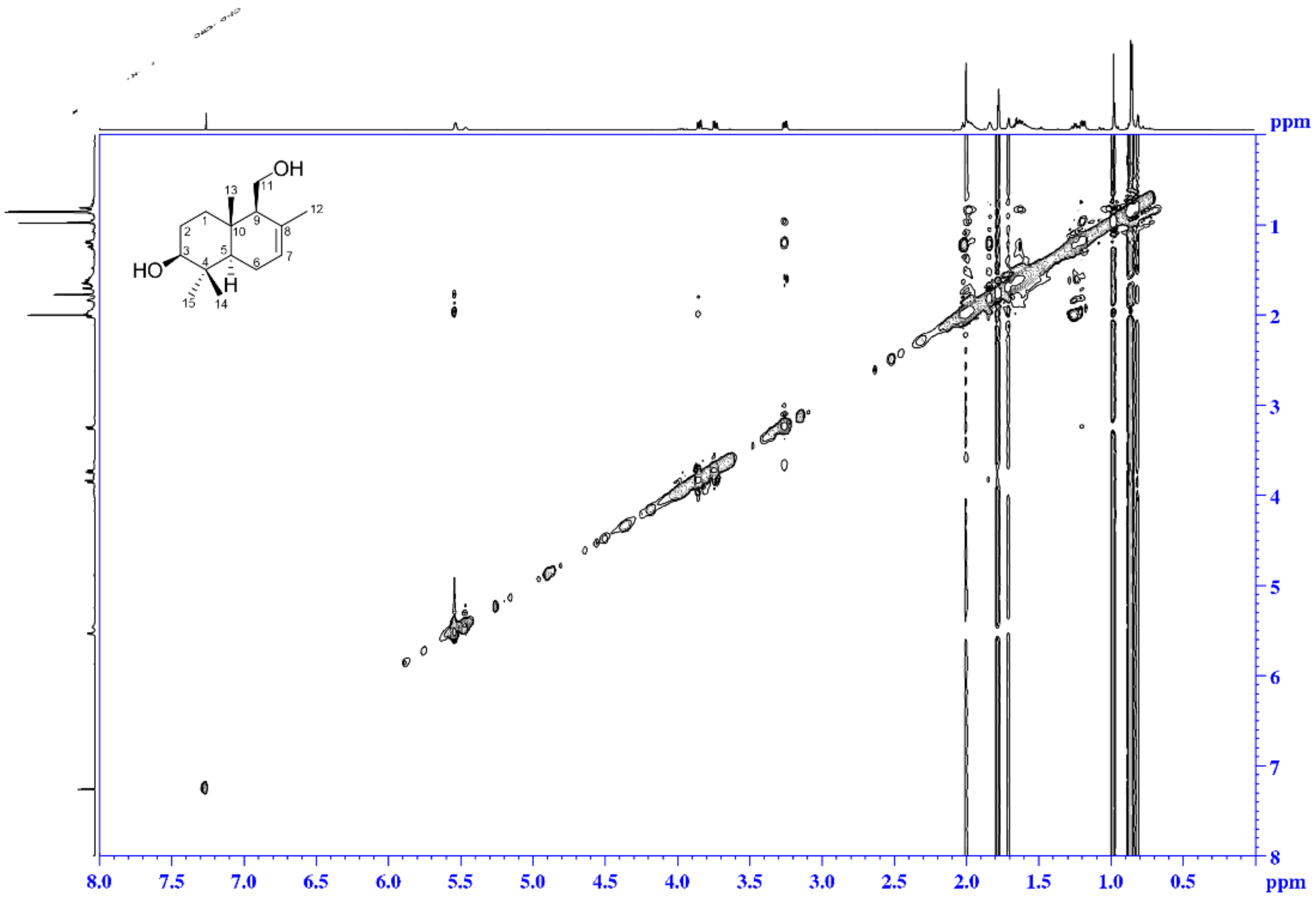

Figure NMR-S10. NOESY spectrum of compound **2** ($CDCl_3$, 300 K, 600 MHz).

Supplementary Data 1

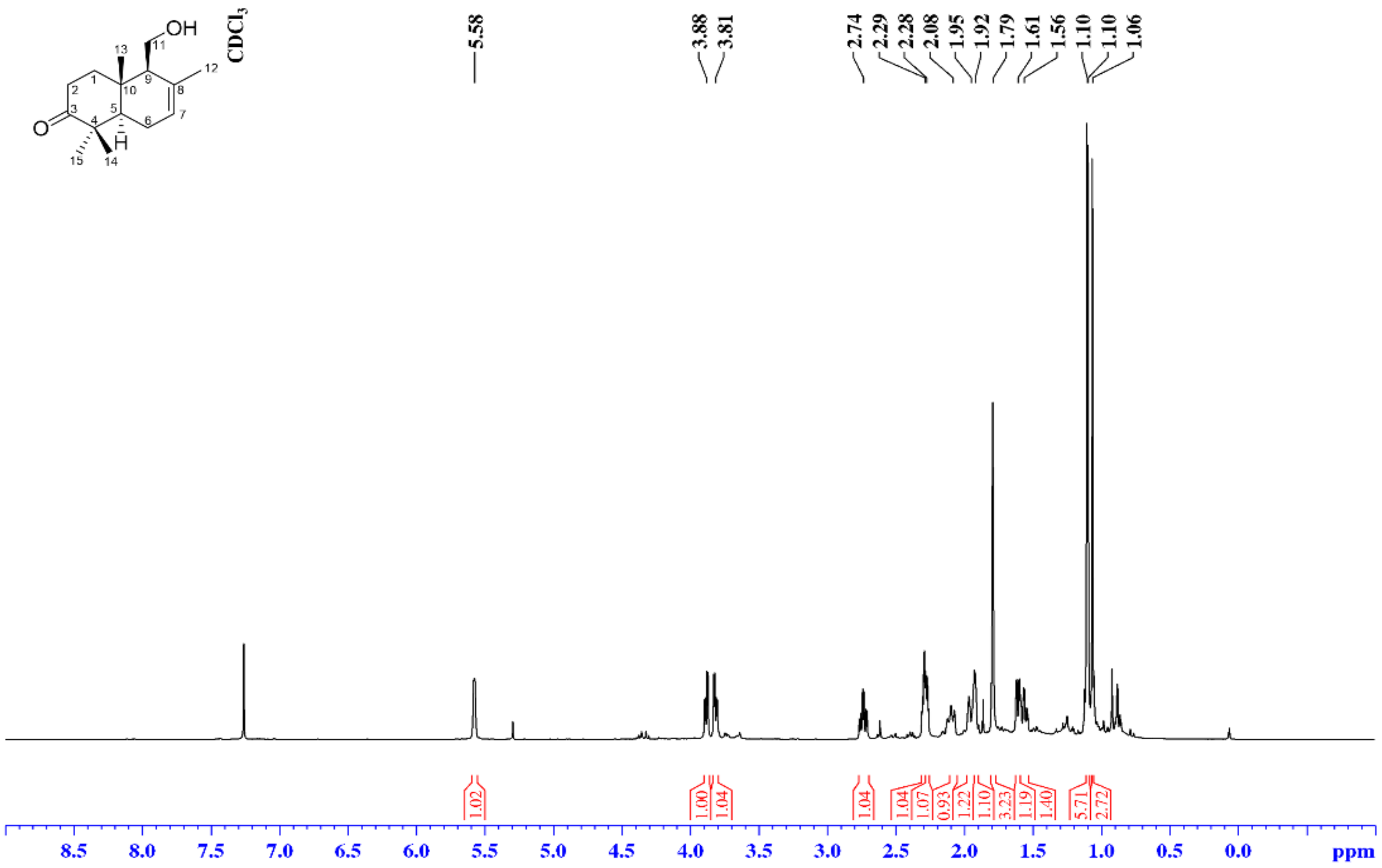

Figure NMR-S11. ^{1}H NMR spectrum of compound **3** ($CDCl_3$, 300 K, 600 MHz).

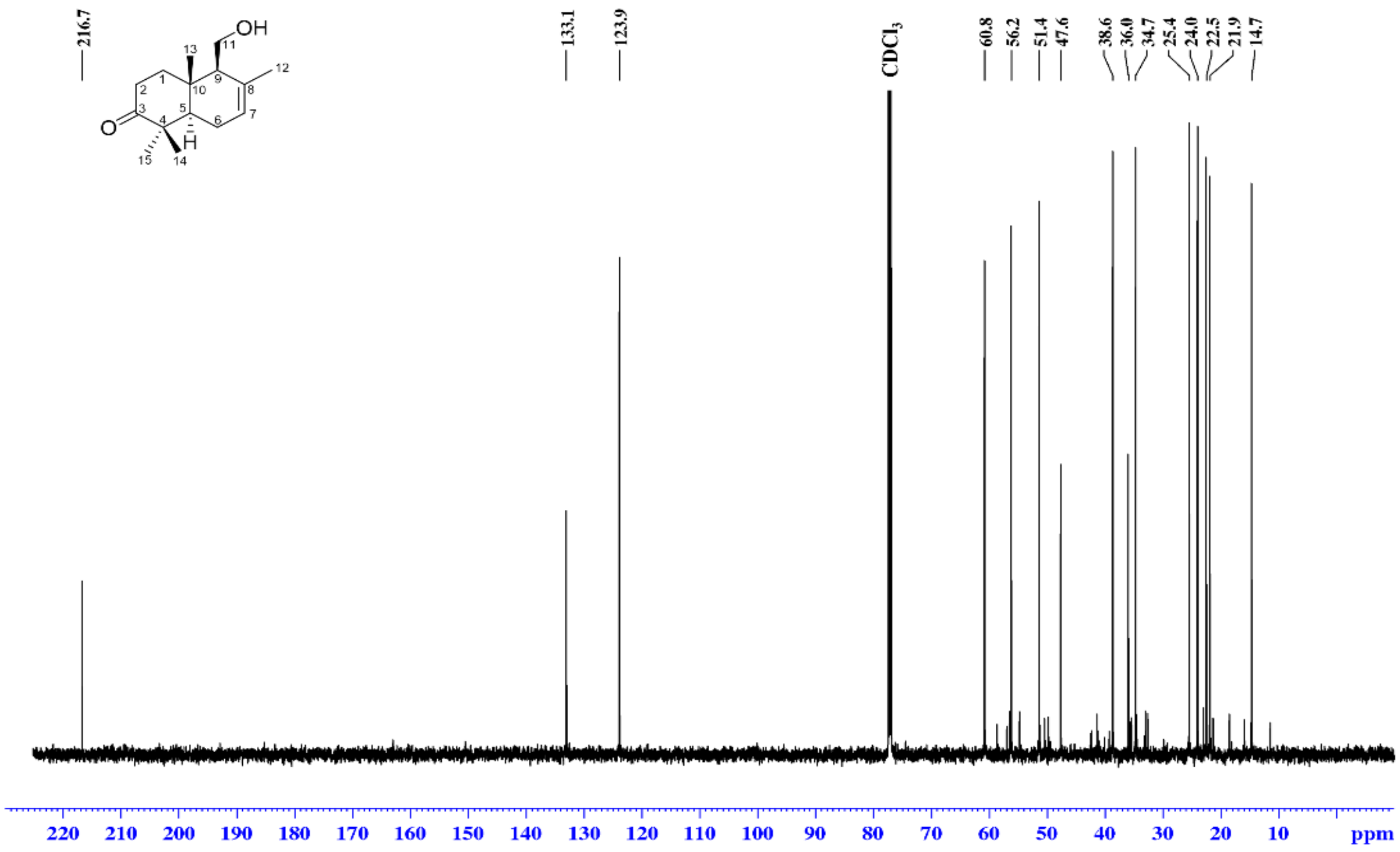

Figure NMR-S12. ^{13}C NMR spectrum of compound **3** ($CDCl_3$, 300 K, 150 MHz).

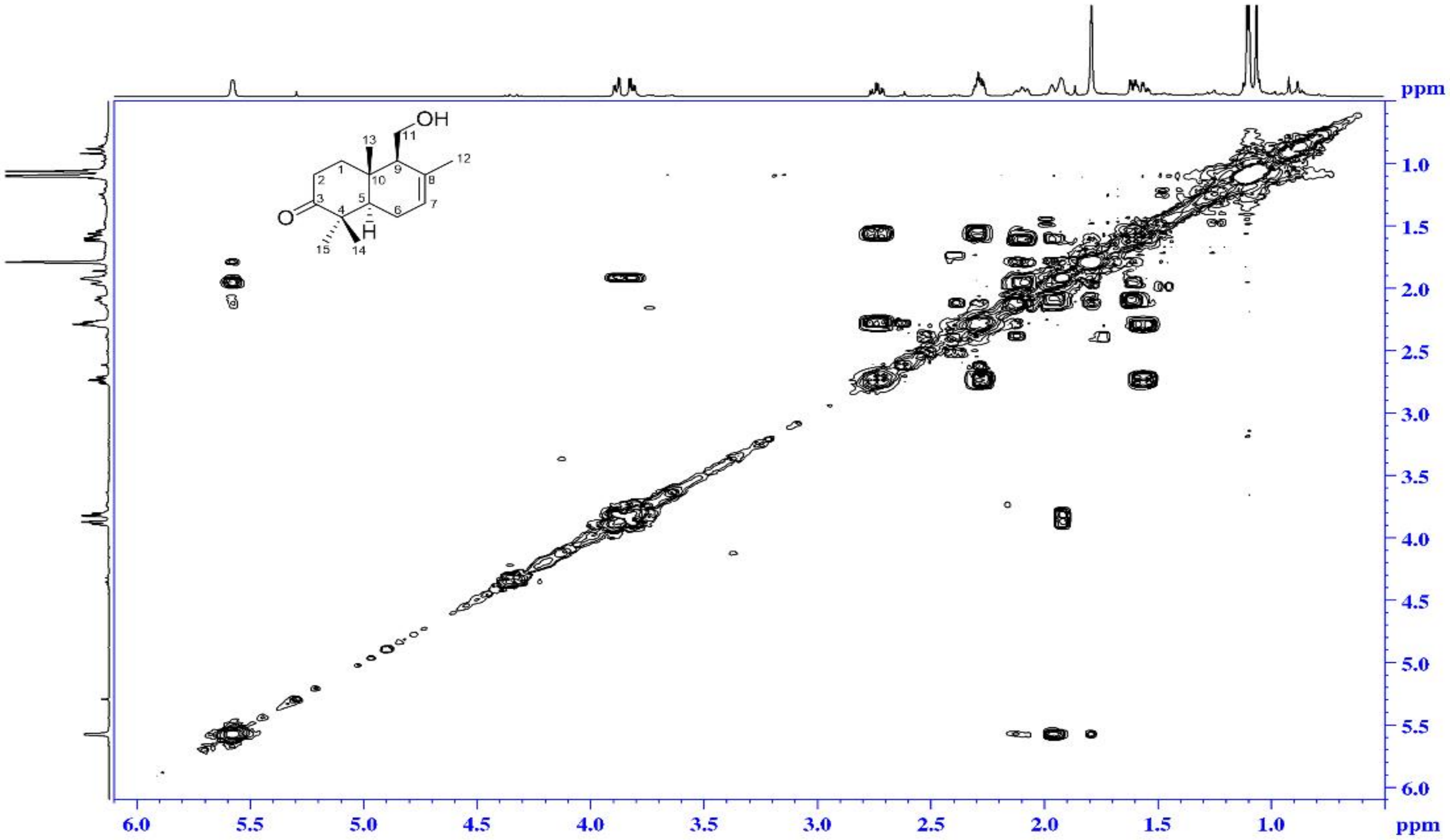

Figure NMR-S13. COSY spectrum of compound **3** ($CDCl_3$, 300 K, 600 MHz).

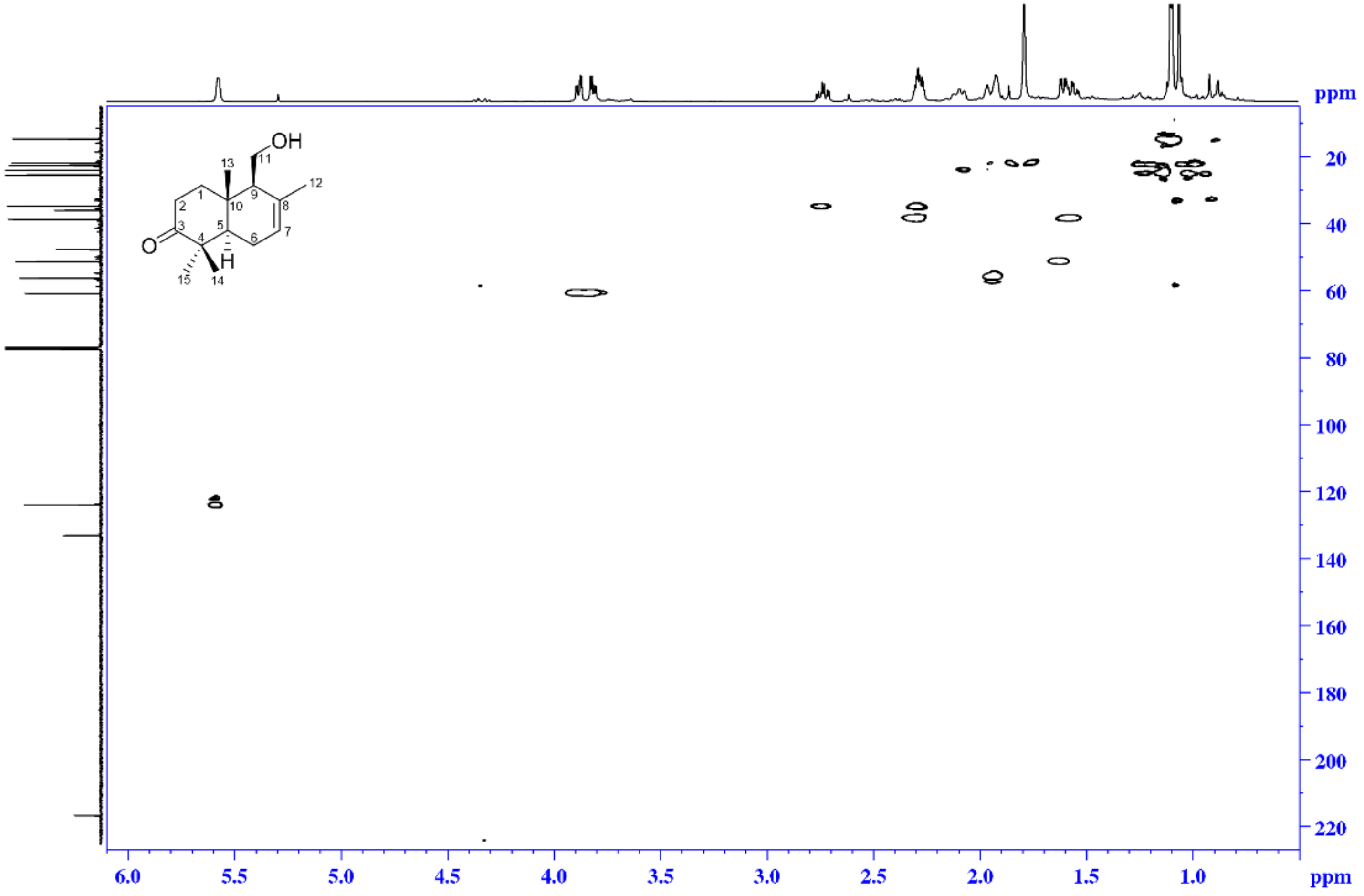

Figure NMR-S14. HSQC spectrum of compound **3** ($CDCl_3$, 300 K, 500 MHz).

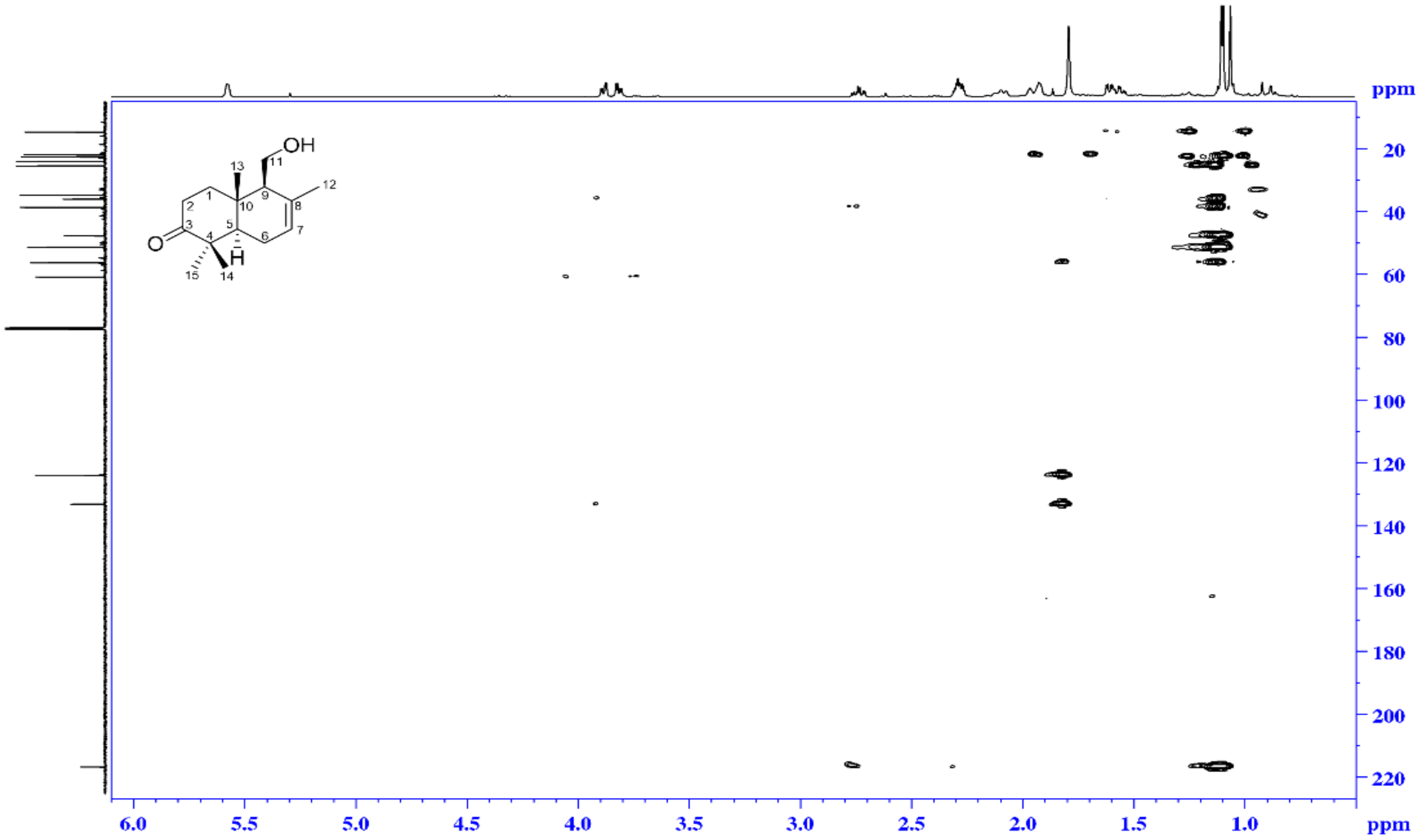

Figure NMR-S15. HMBC spectrum of compound **3** ($CDCl_3$, 300 K, 500 MHz).

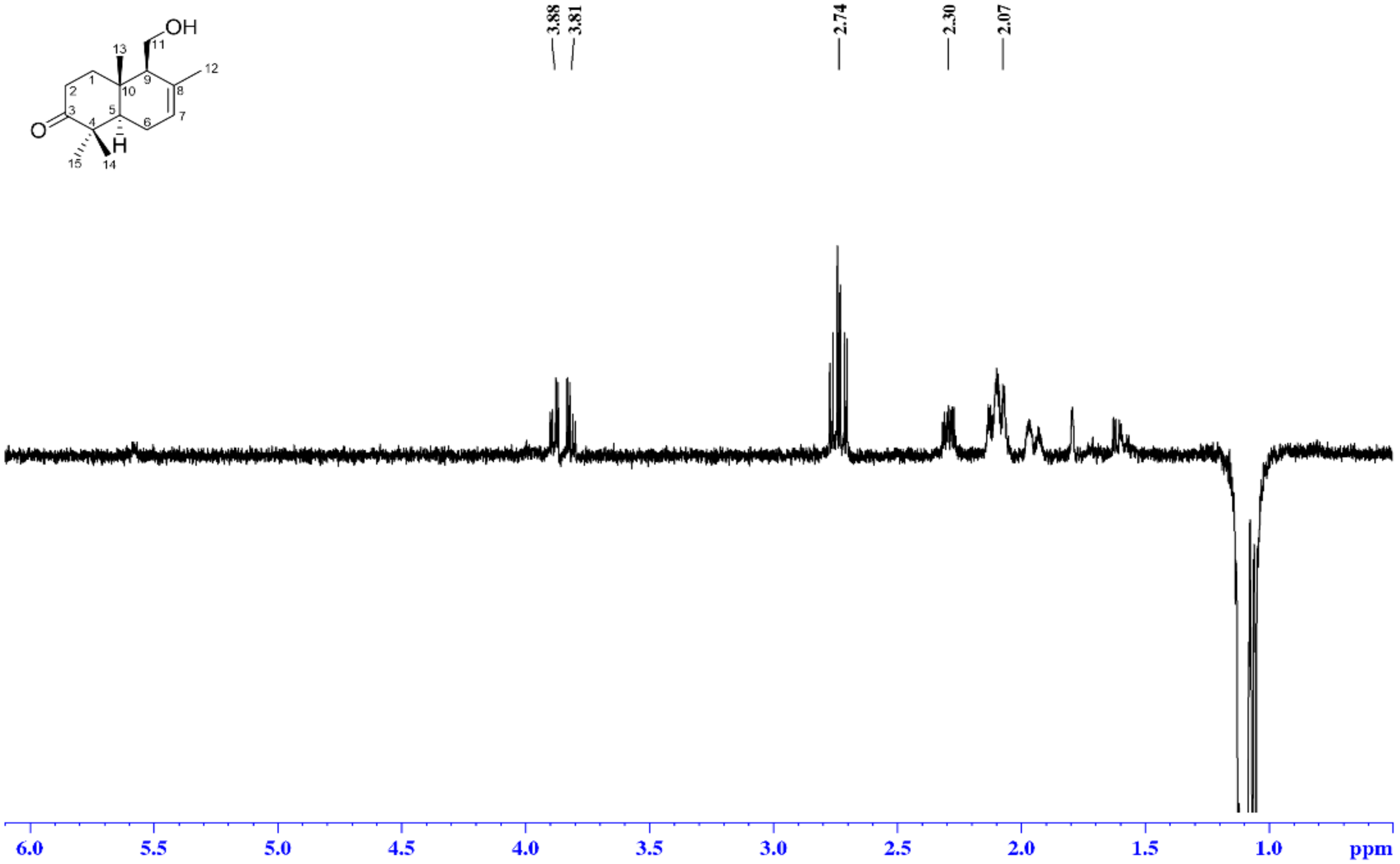

Figure NMR-S16. Selective NOE spectrum of compound **3** at 1.100 ppm ($CDCl_3$, 300 K, 500 MHz).

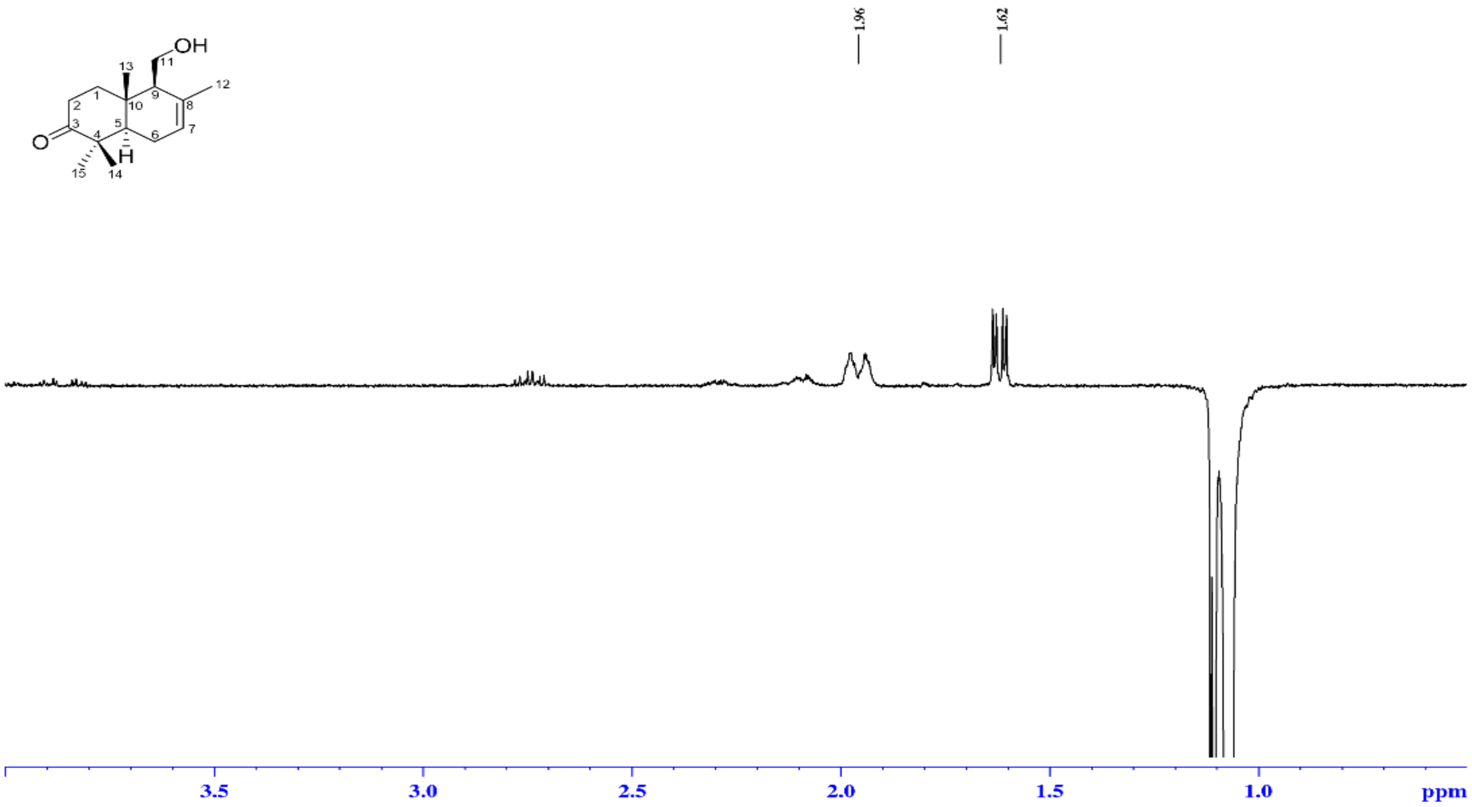

Figure NMR-S17. Selective NOE spectrum of compound **3** at 1.06 ppm ($CDCl_3$, 300 K, 500 MHz).

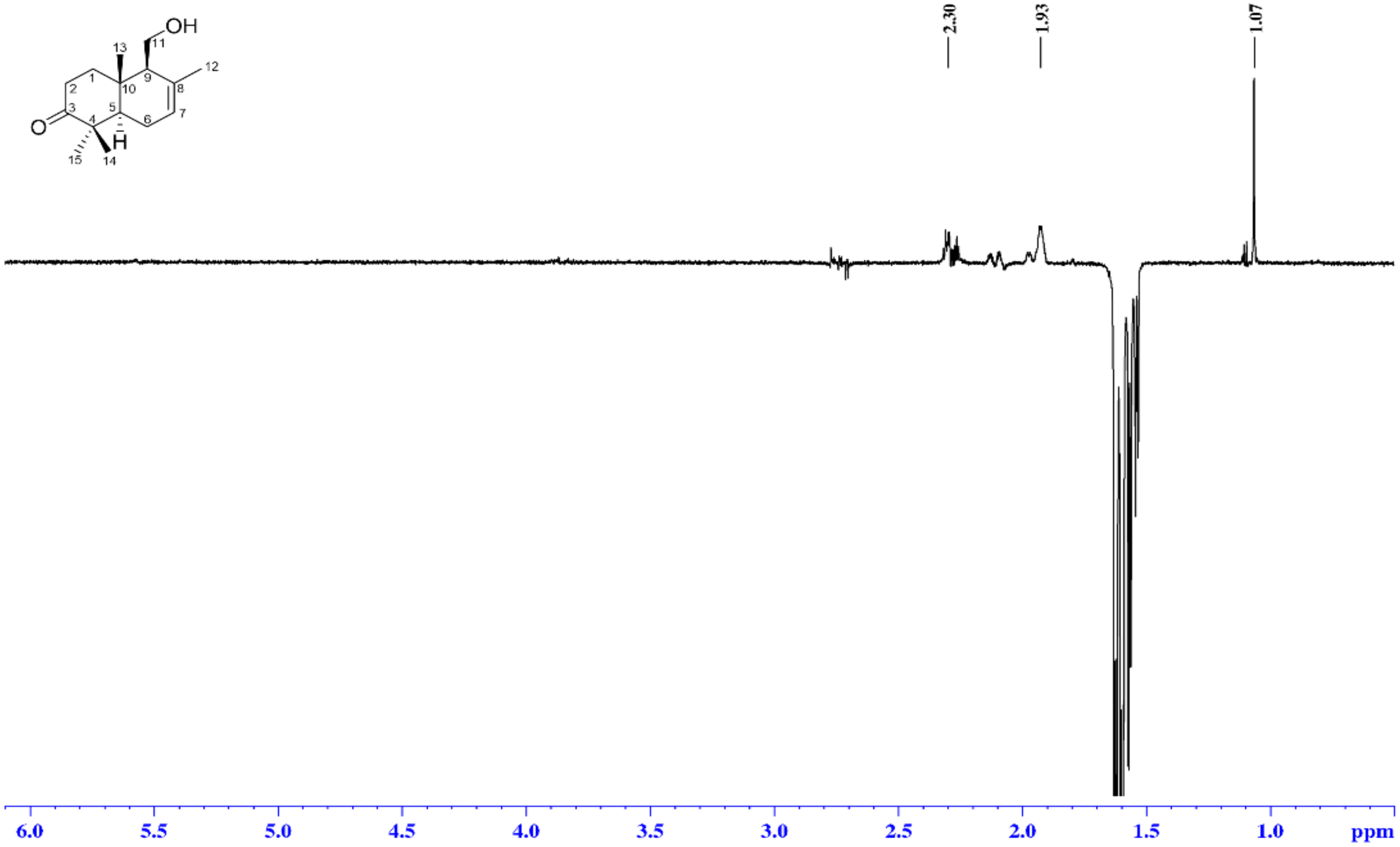

Figure NMR-S18. Selective NOE spectrum of compound **3** at 1.61 ppm ($CDCl_3$, 300 K, 500 MHz).

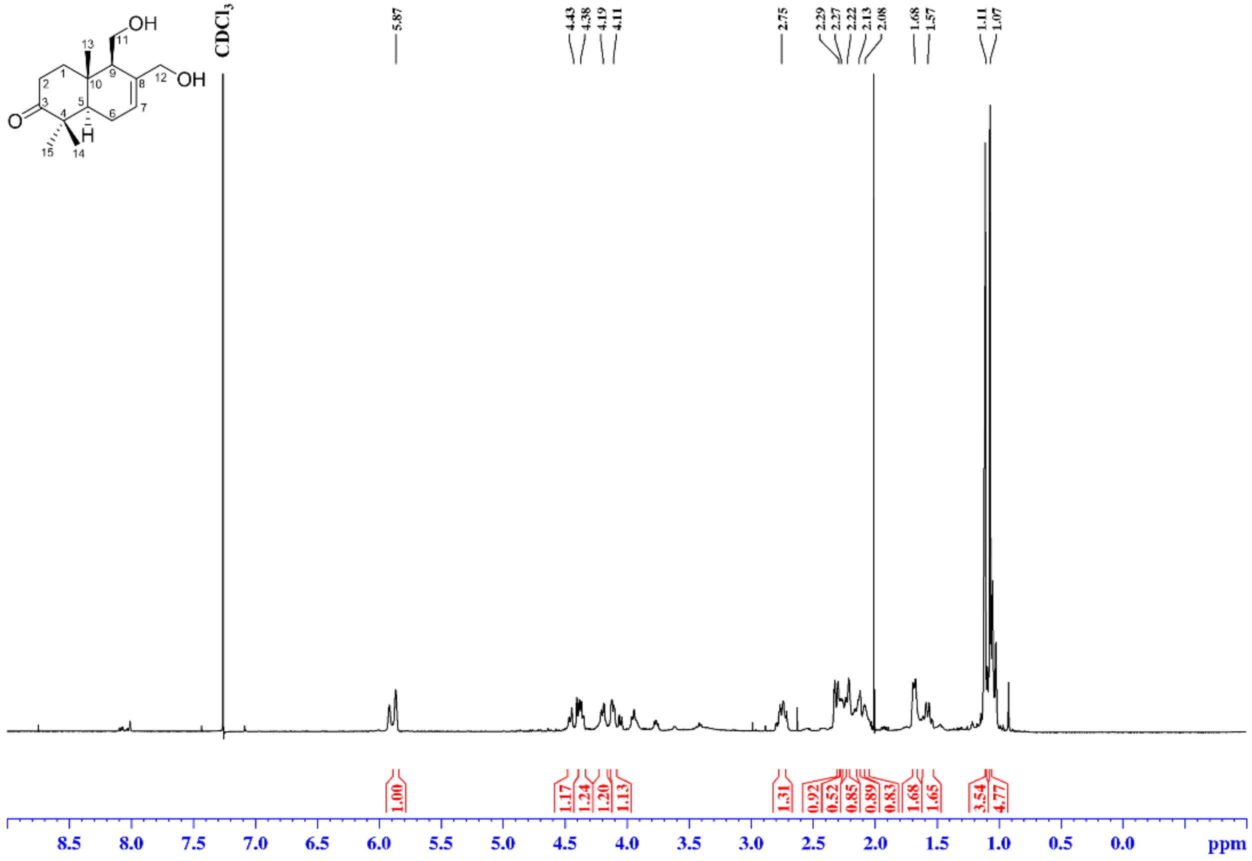

Figure NMR-S19. ^{1}H NMR spectrum of compound **4** ($CDCl_3$, 300 K, 600 MHz).

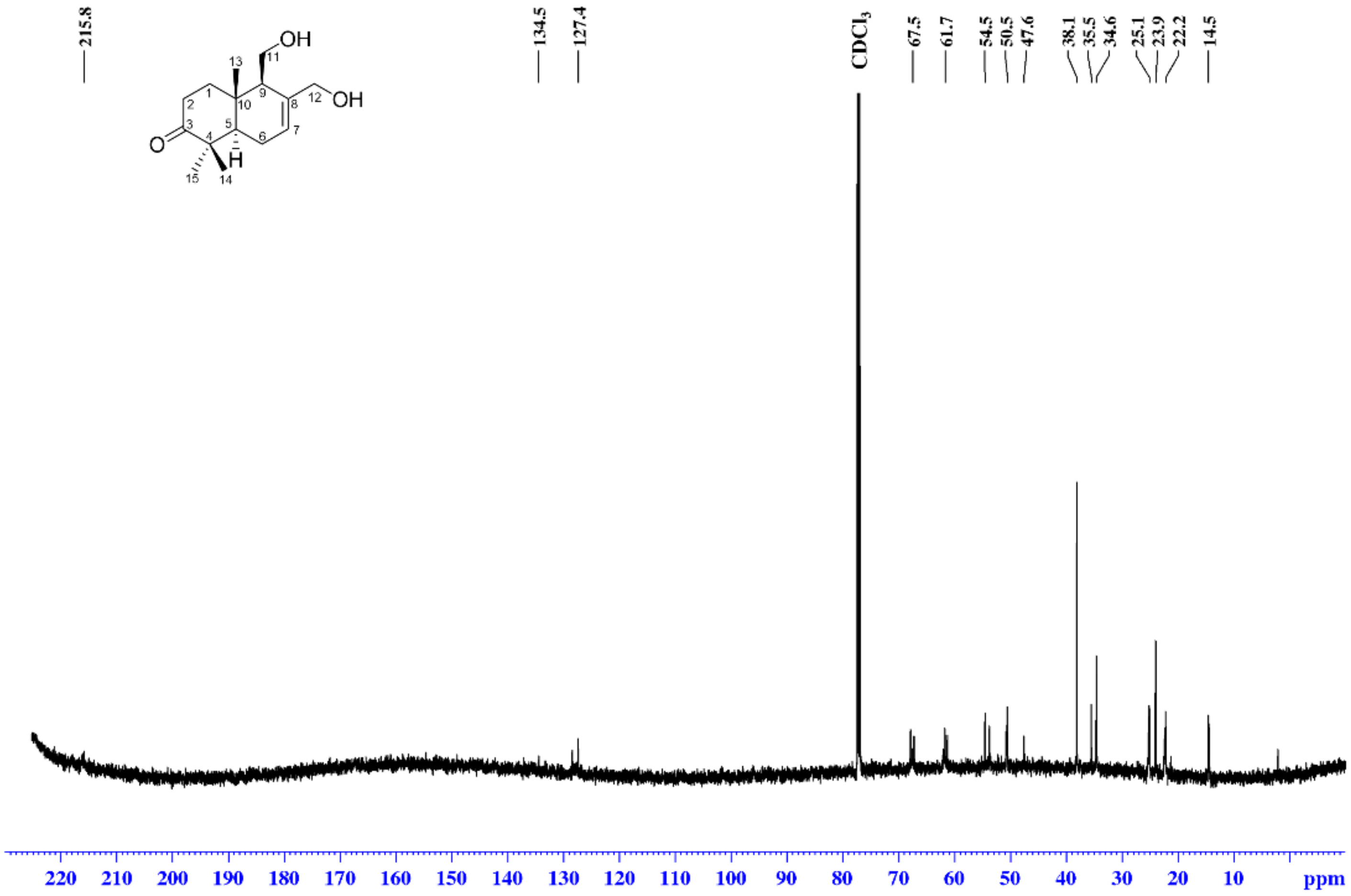

Figure NMR-S20. ^{13}C NMR spectrum of compound **4** ($CDCl_3$, 300 K, 150 MHz).

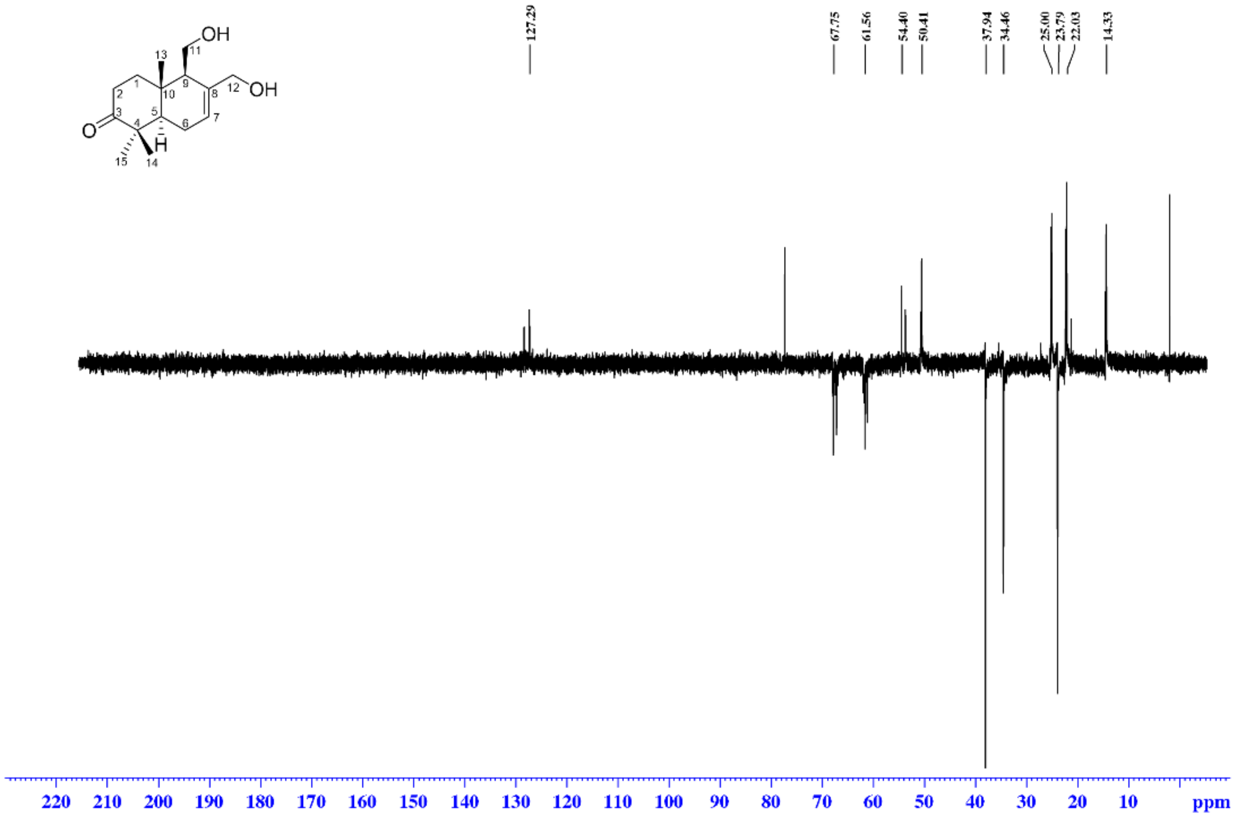

Figure NMR-S21. ^{13}C NMR DEPT spectrum of compound **4** ($CDCl_3$, 300 K, 150 MHz).

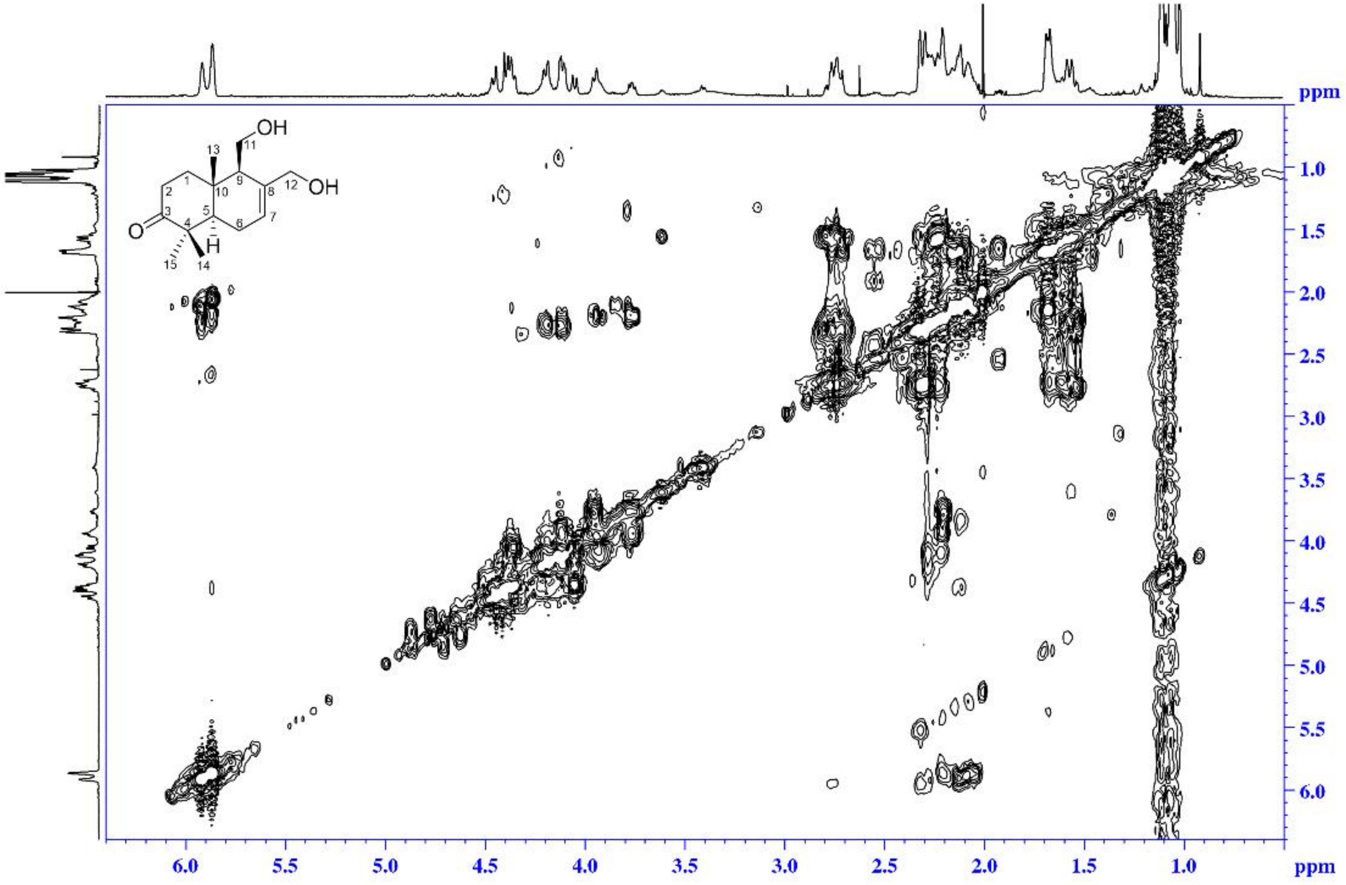

Figure NMR-S22. COSY spectrum of compound **4** ($CDCl_3$, 300 K, 600 MHz).

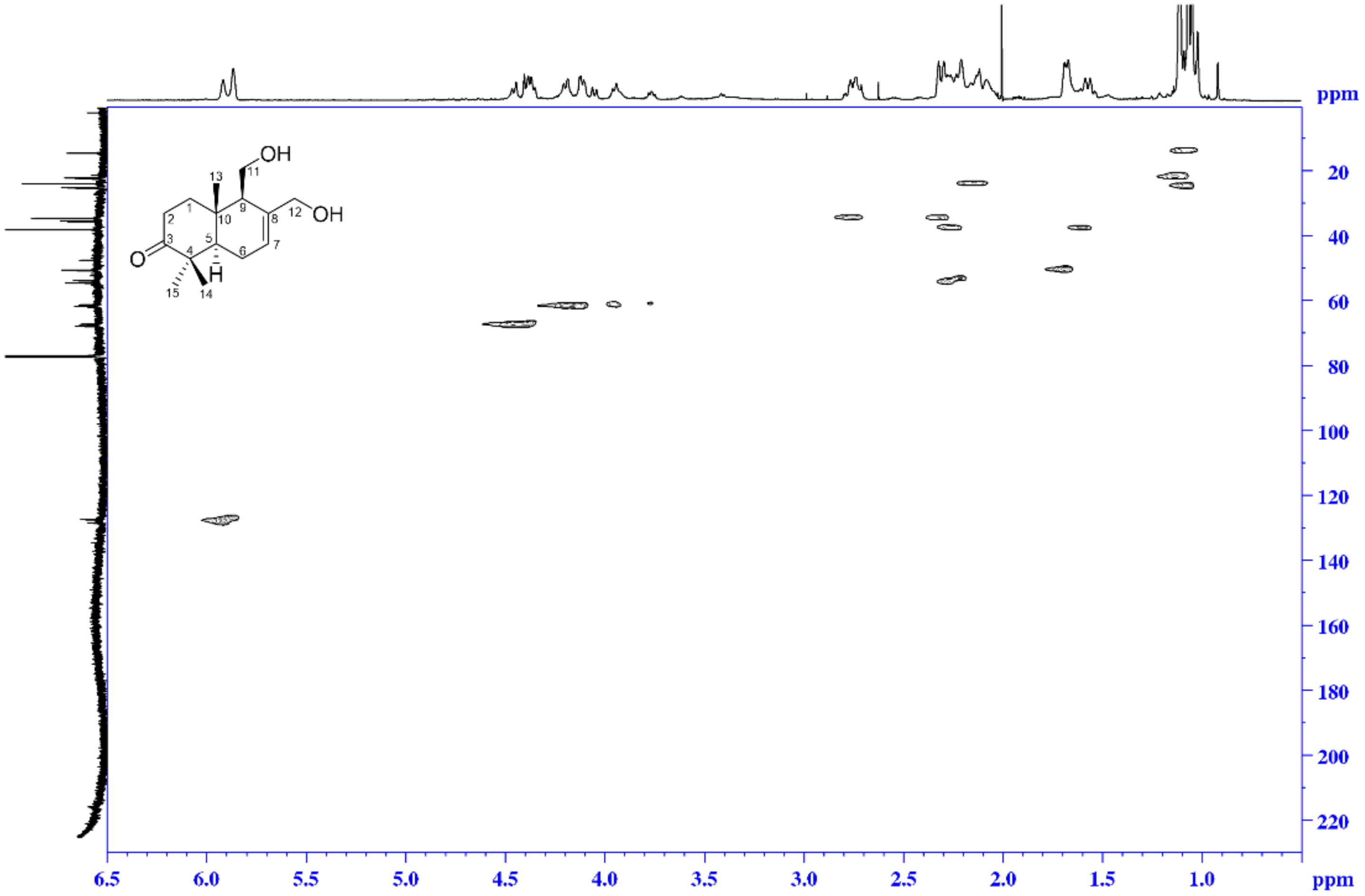

Figure NMR-S23. HSQC spectrum of compound **4** ($CDCl_3$, 300 K, 600 MHz).

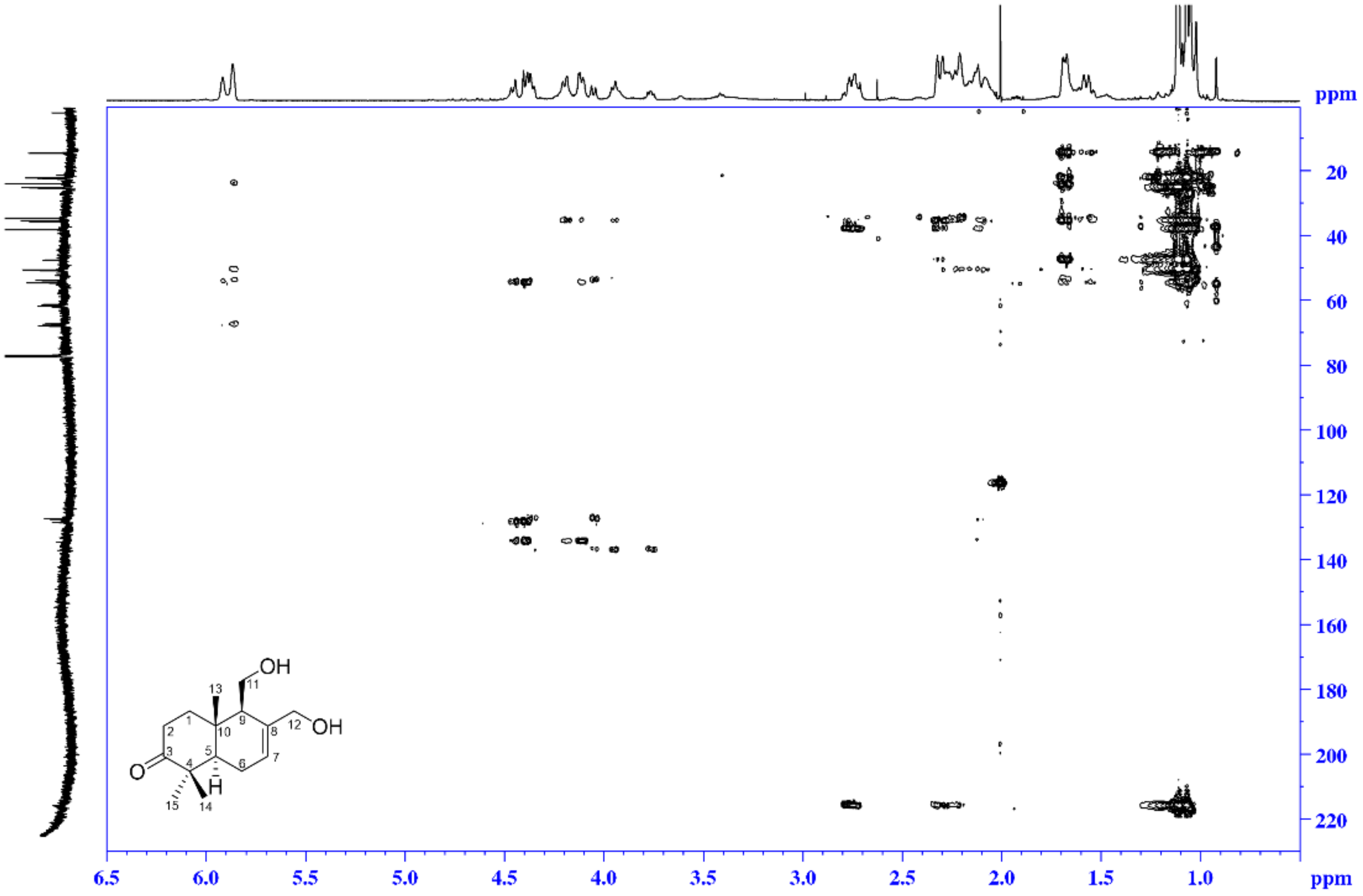

Figure NMR-S24. HMBC spectrum of compound **4** ($CDCl_3$, 300 K, 600 MHz).

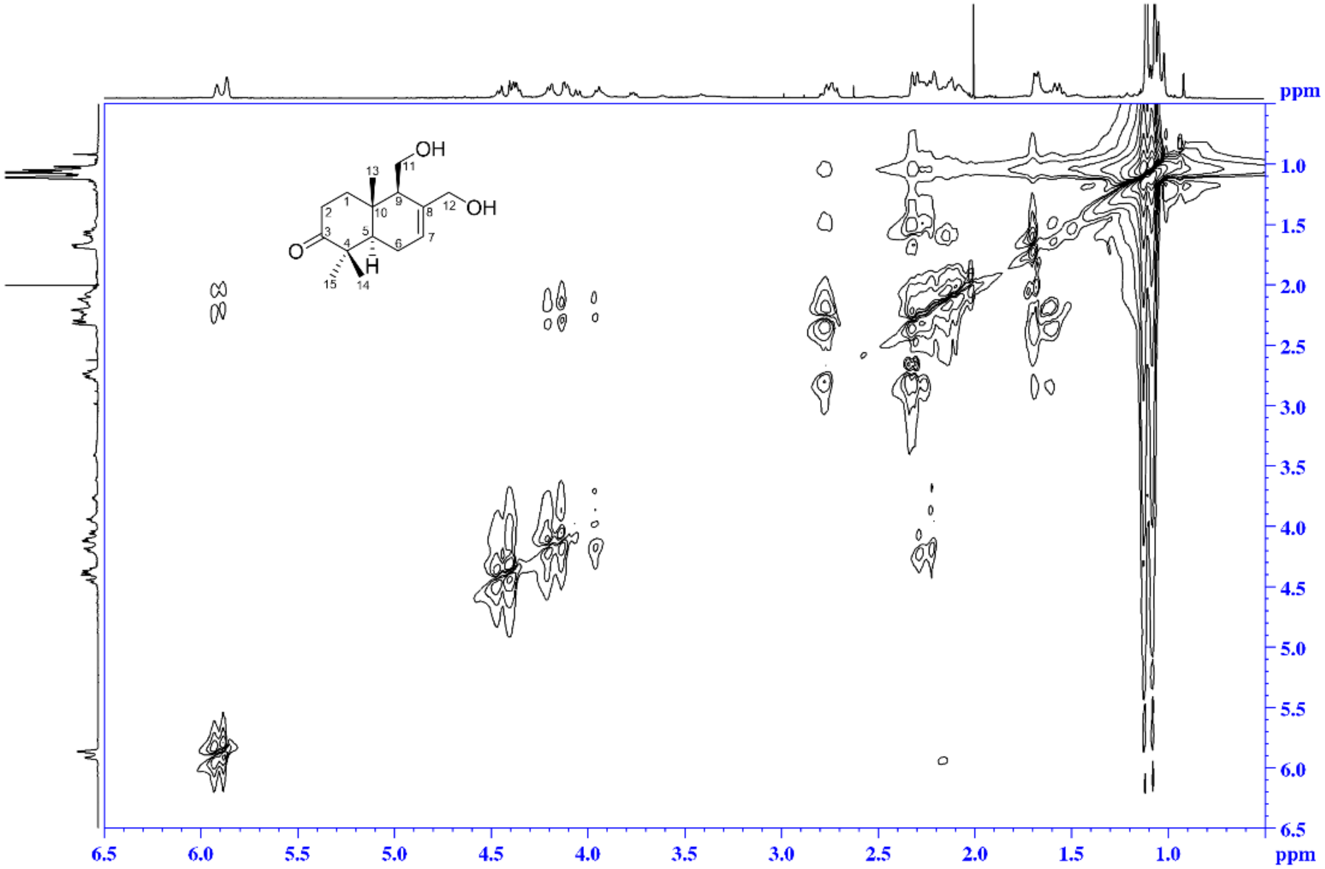

Figure NMR-S25. TOCSY spectrum of compound **4** ($CDCl_3$, 300 K, 600 MHz).

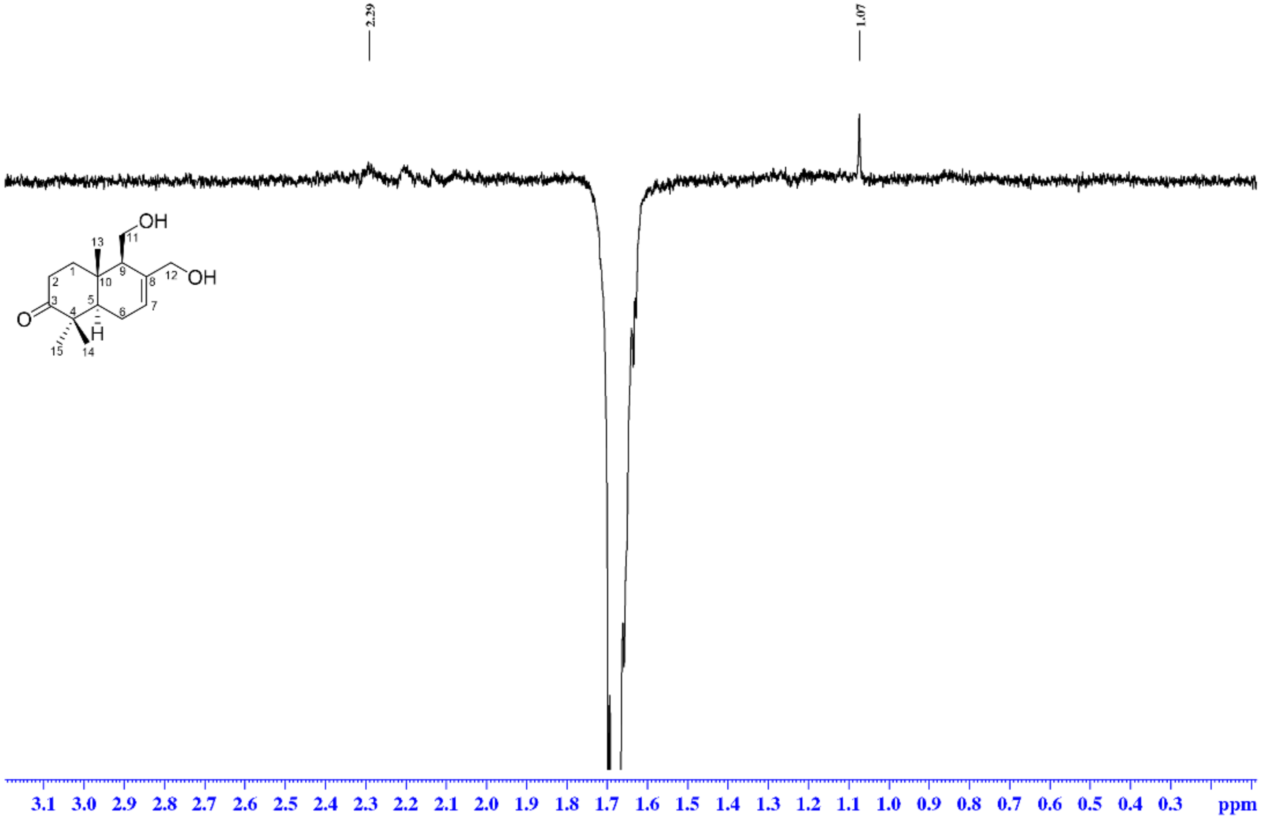

Figure NMR-S26. Selective NOE spectrum of compound **4** at 1.68 ppm ($CDCl_3$, 300 K, 600 MHz).

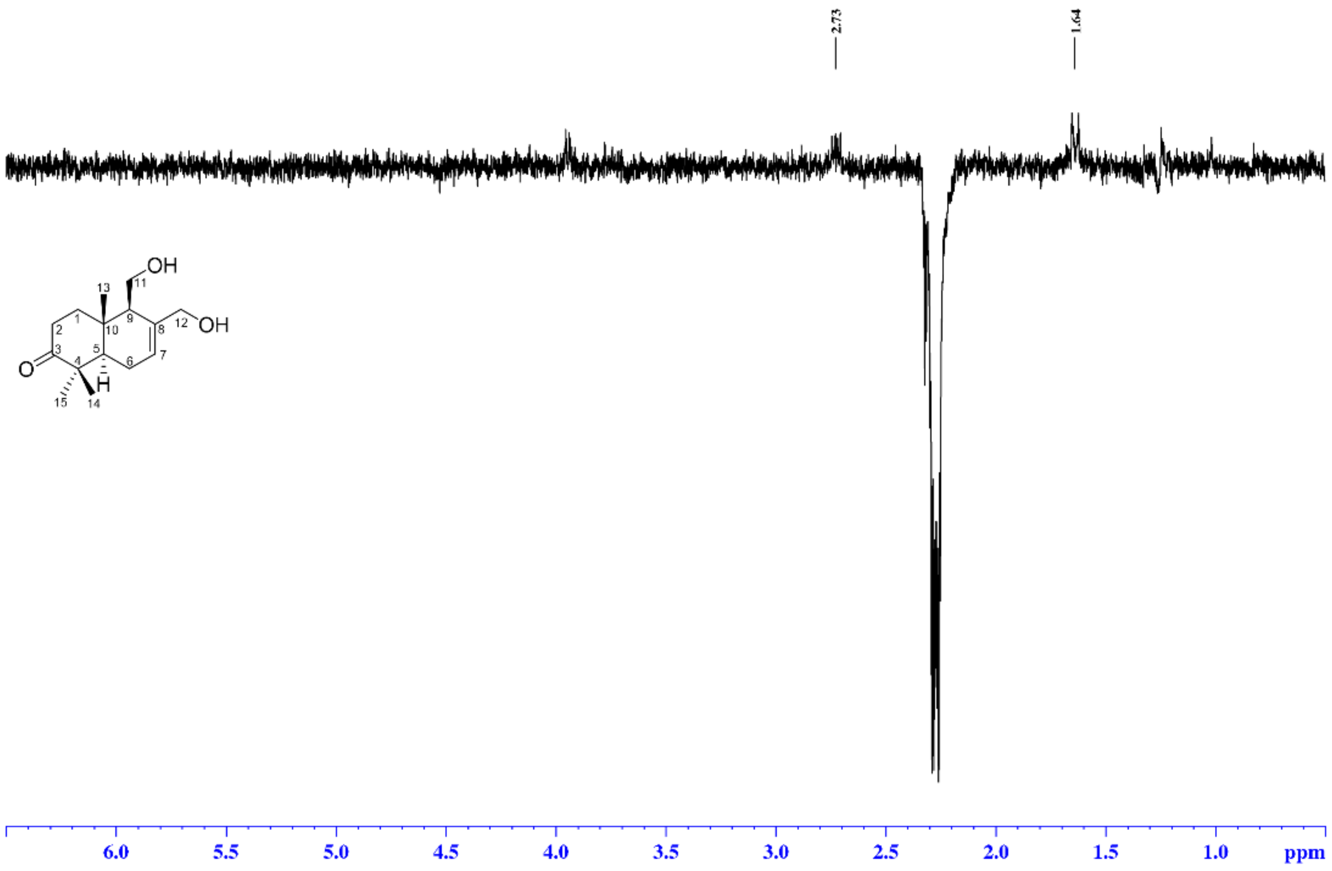

Figure NMR-S27. Selective NOE spectrum of compound **4** at 2.27 ppm ($CDCl_3$, 300 K, 600 MHz).

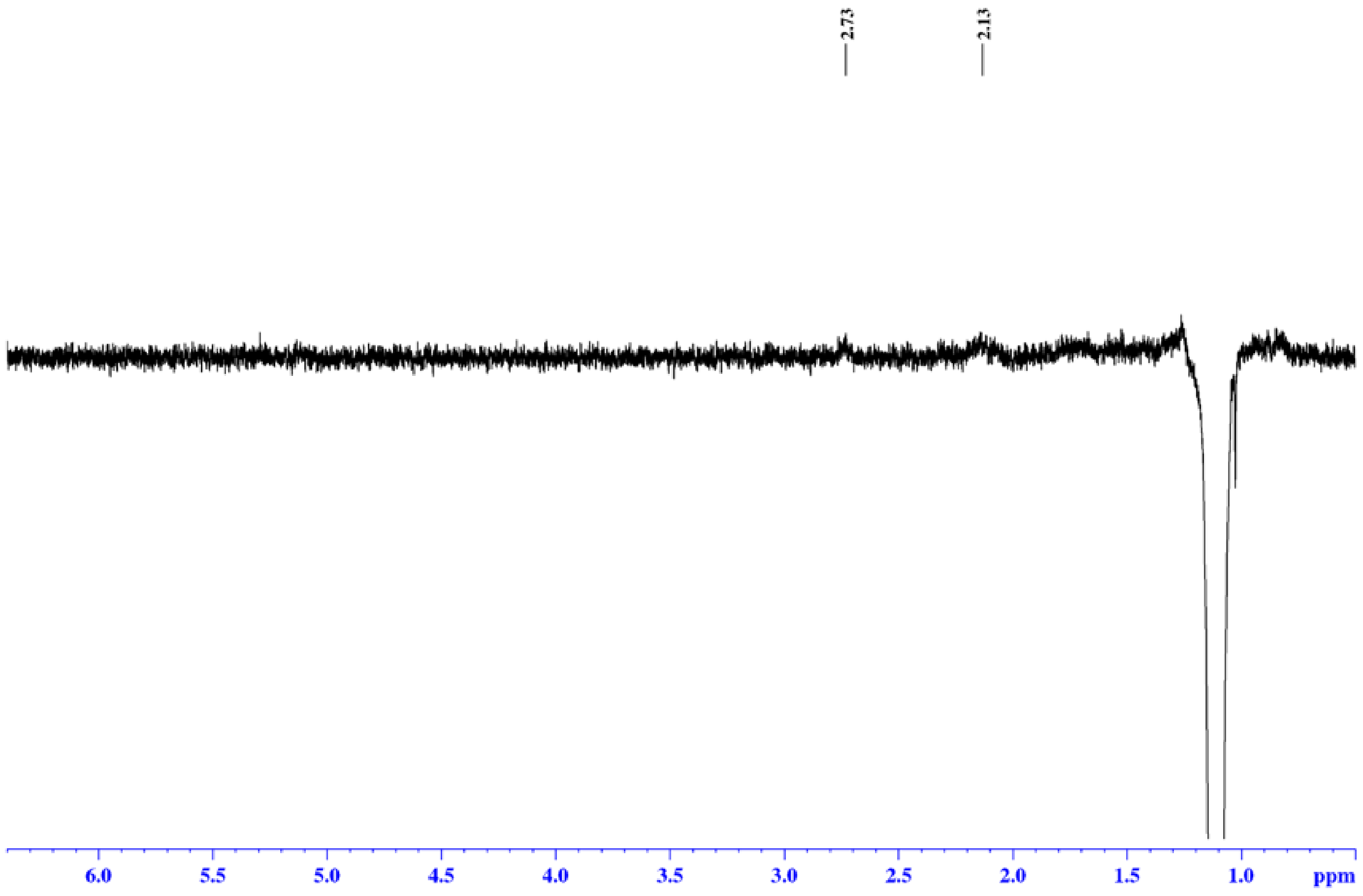

Figure NMR-S28. Selective NOE spectrum of compound **4** at 1.11 ppm ($CDCl_3$, 300 K, 600 MHz).

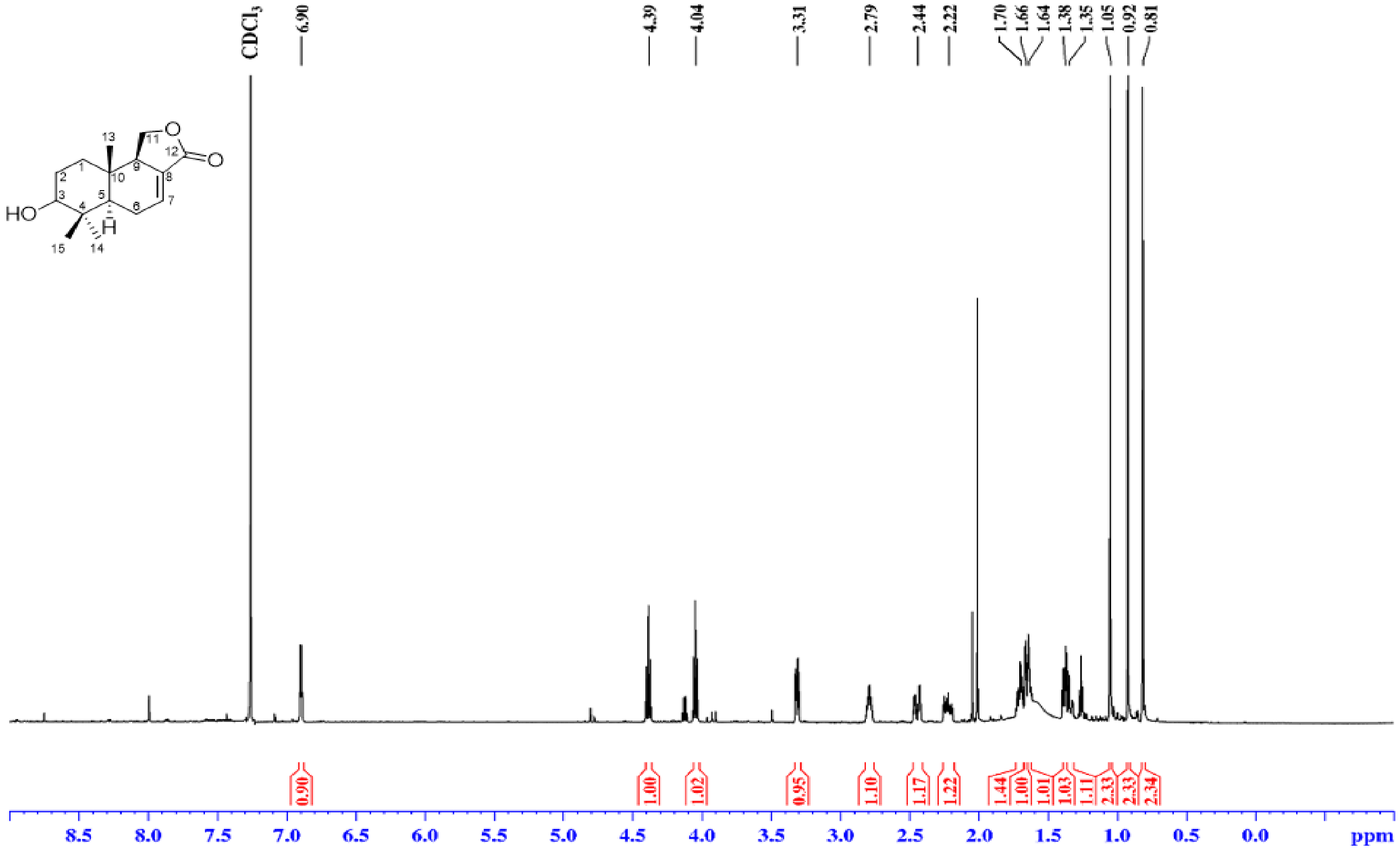

Figure NMR-S29. ^{1}H NMR spectrum of compound **5** ($CDCl_3$, 300 K, 600 MHz).

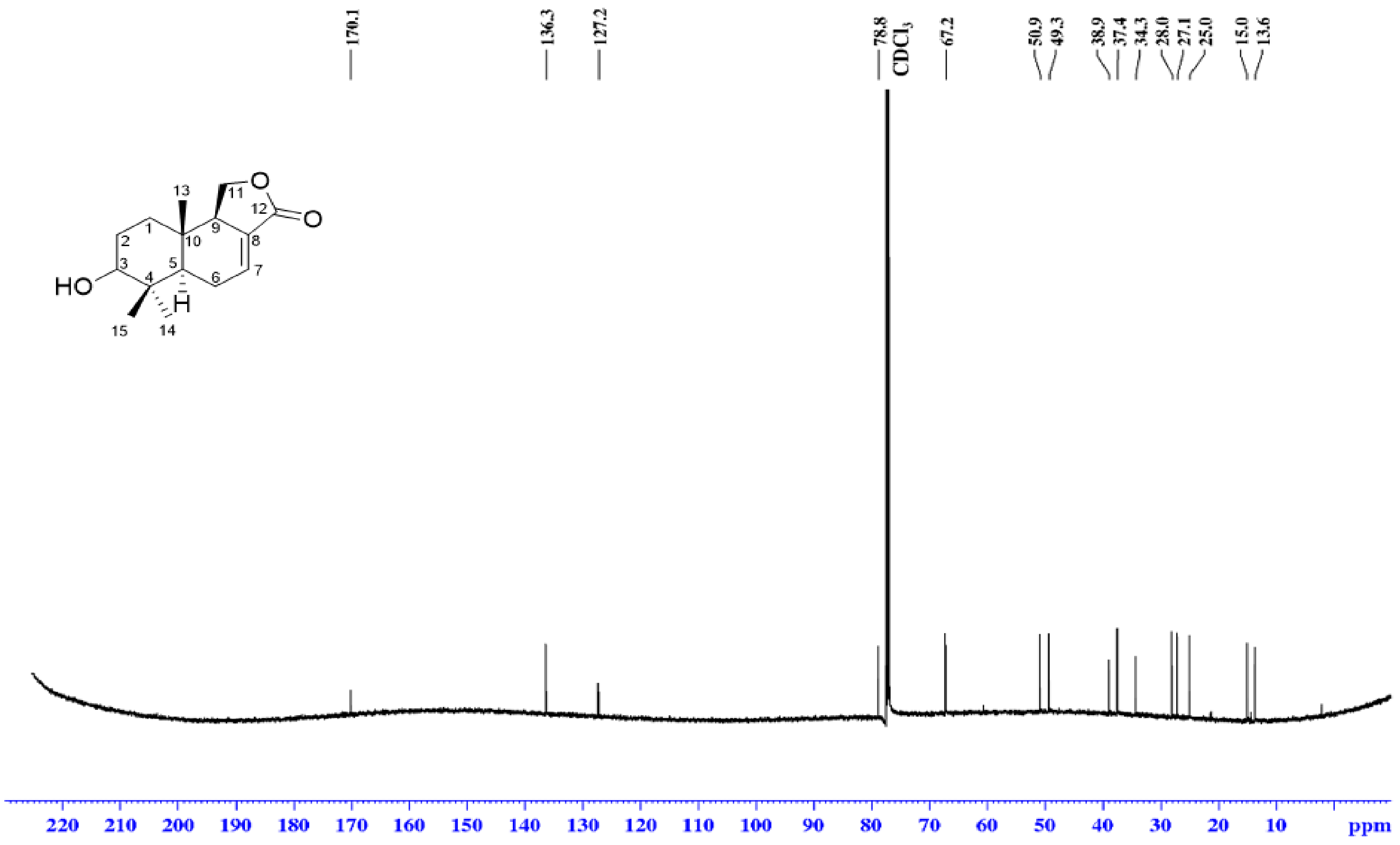

Figure NMR-S30. ^{13}C NMR spectrum of compound **5** ($CDCl_3$, 300 K, 150 MHz).

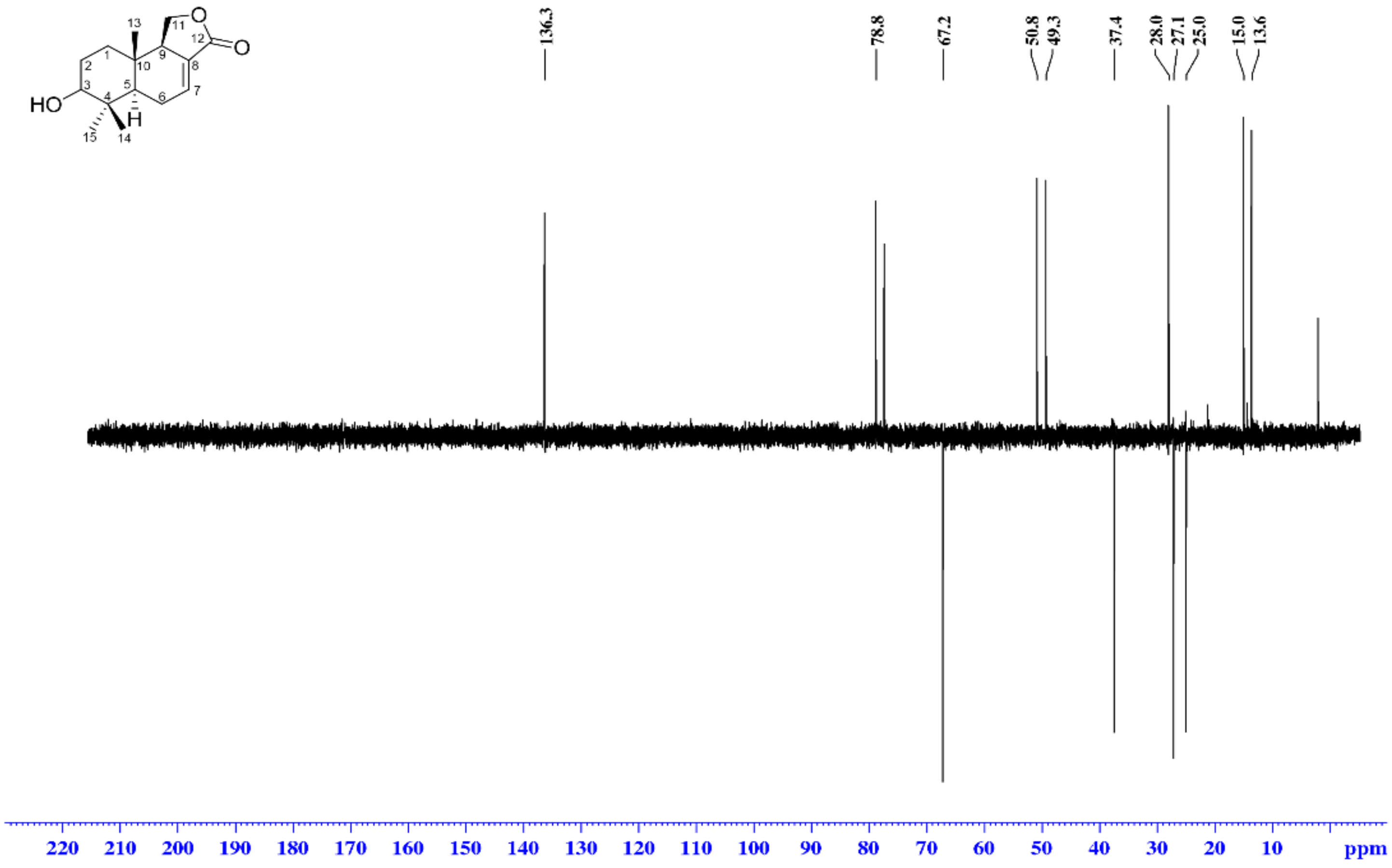

Figure NMR-S31. ^{13}C NMR DEPT spectrum of compound **5** ($CDCl_3$, 300 K, 150 MHz).

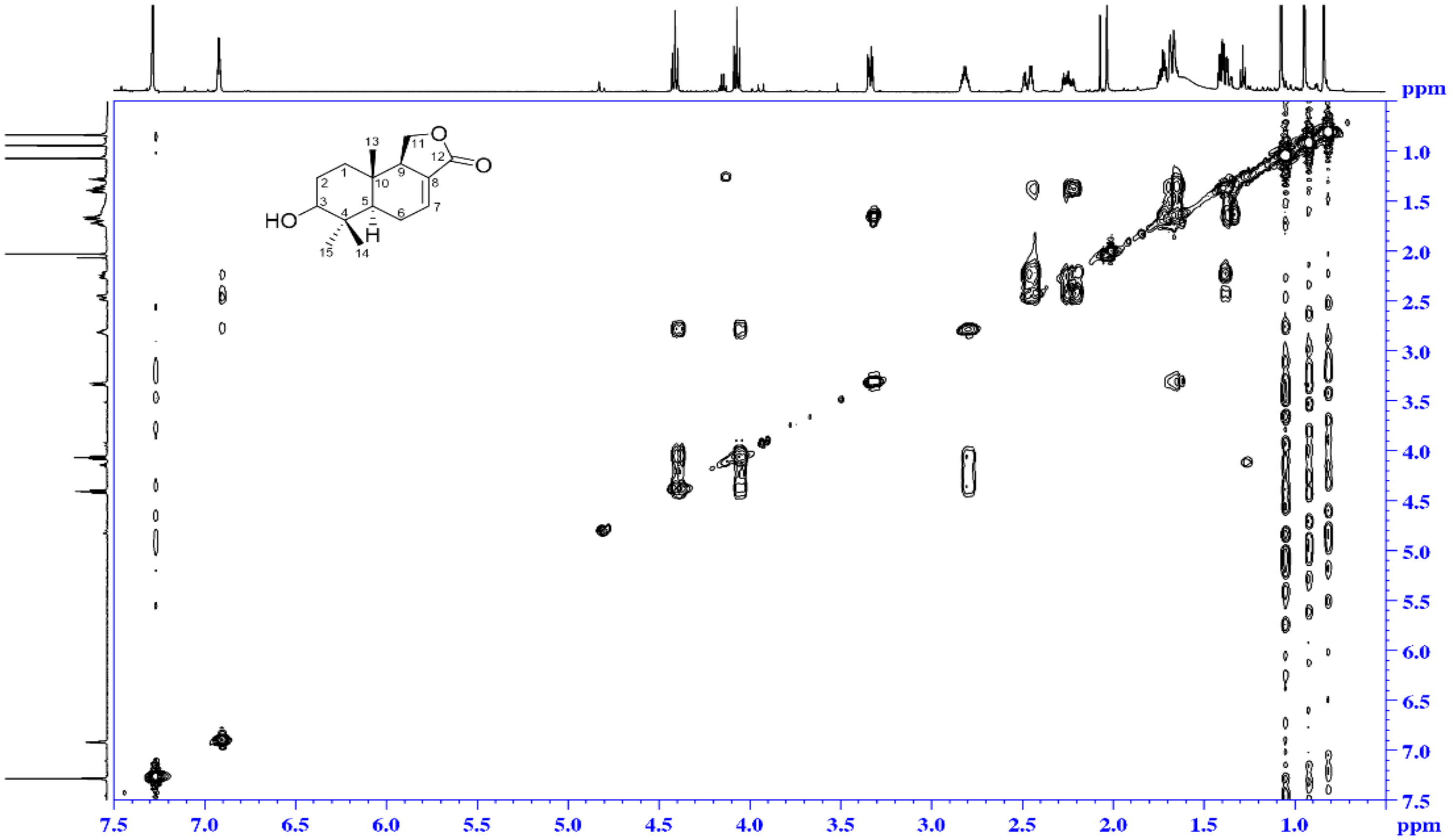

Figure NMR-S32. COSY spectrum of compound **5** ($CDCl_3$, 300 K, 600 MHz).

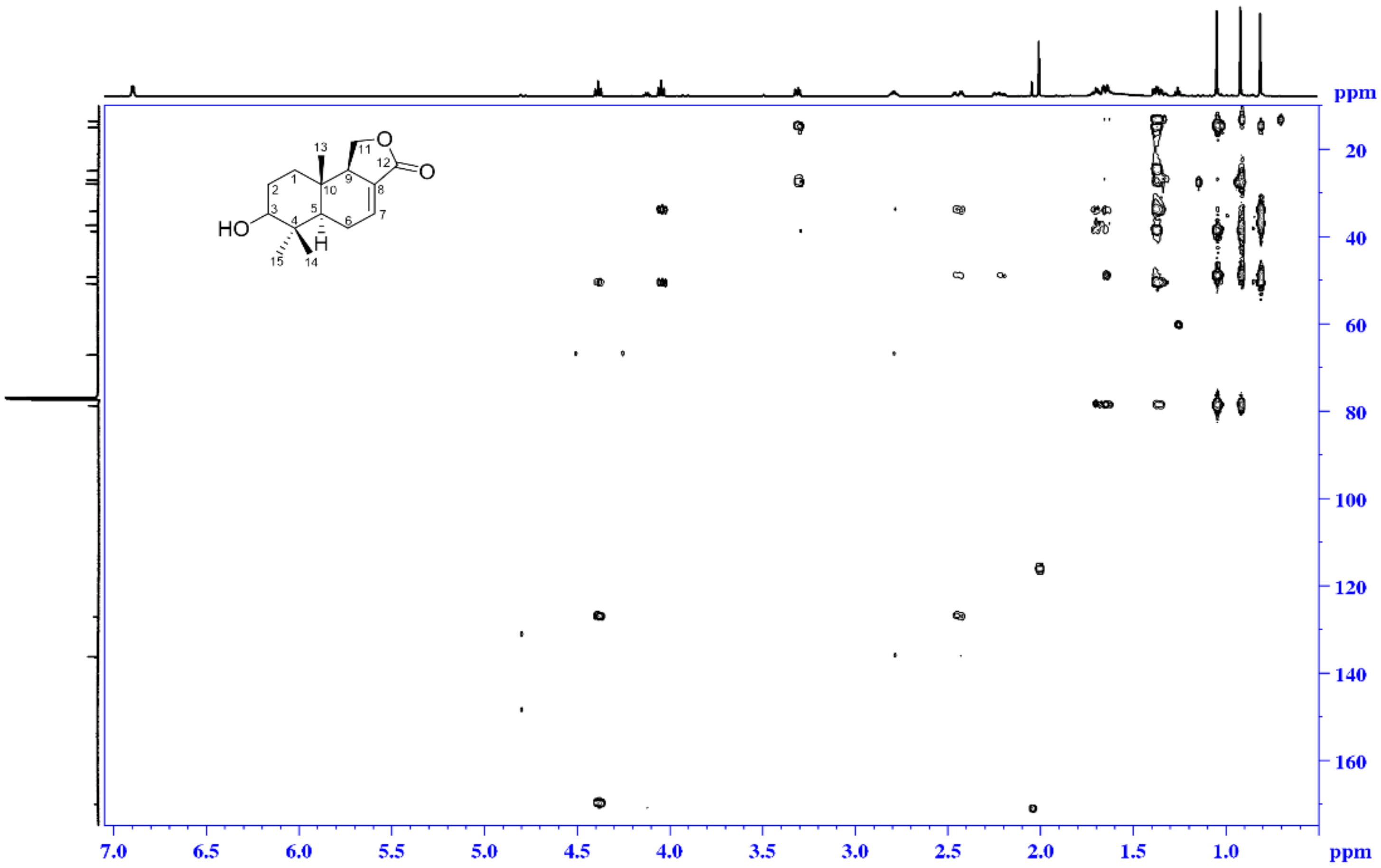

Figure NMR-S33. HSQC spectrum of compound **5** ($CDCl_3$, 300 K, 600 MHz).

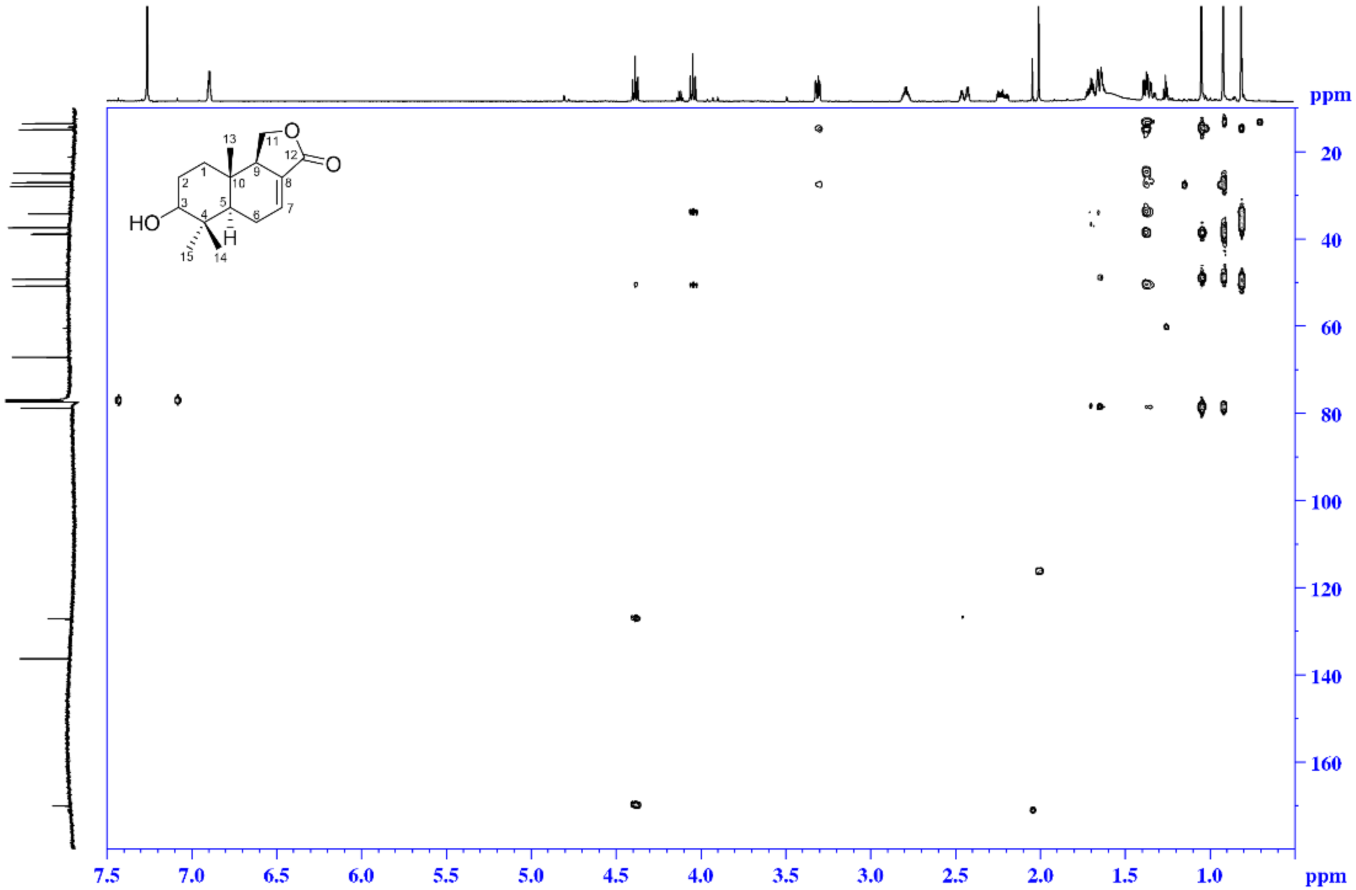

Figure NMR-S34. HMBC spectrum of compound **5** ($CDCl_3$, 300 K, 600 MHz).

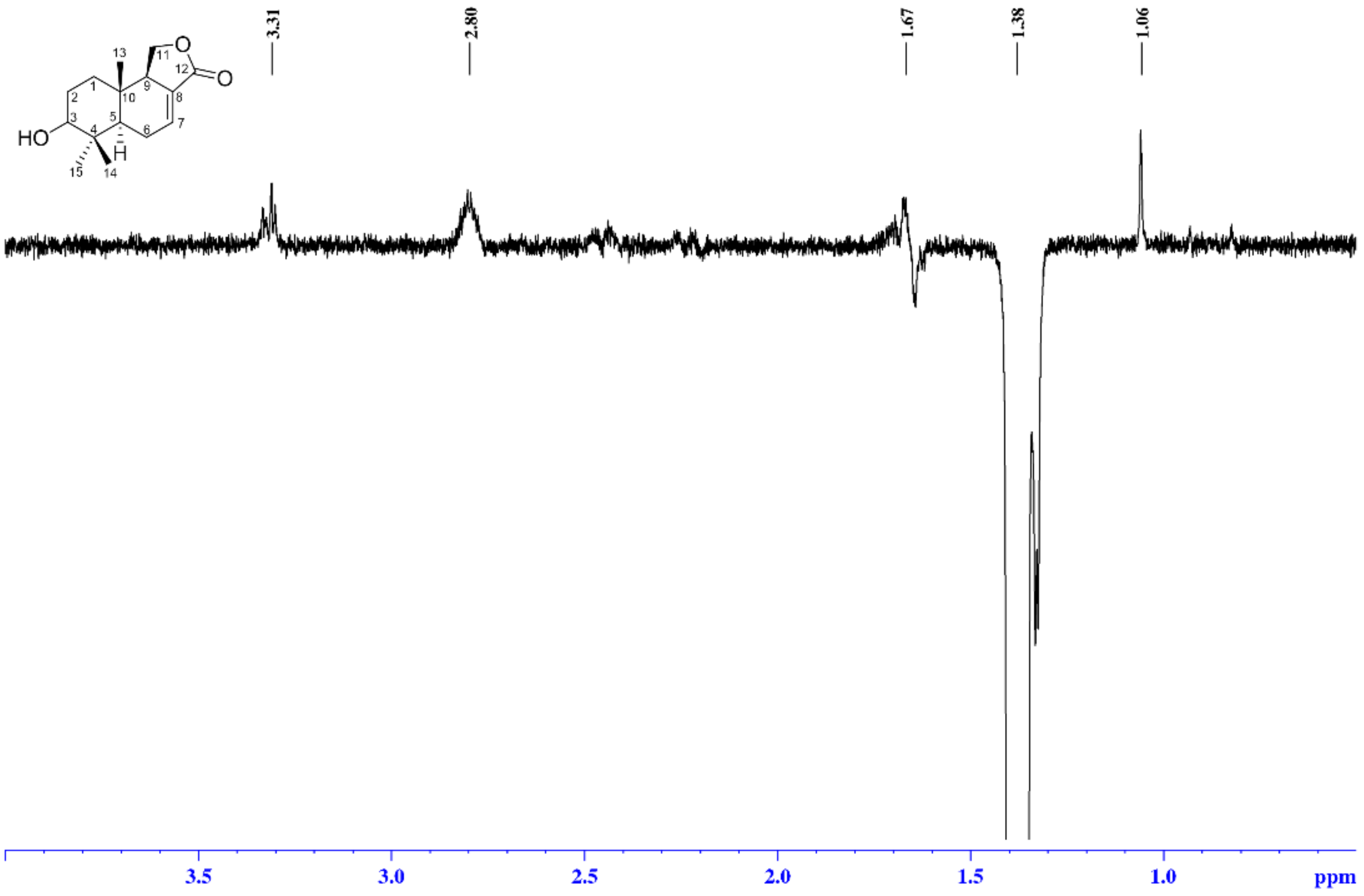

Figure NMR-S35. Selective NOE spectrum of compound **5** at 1.38 ppm ($CDCl_3$, 300 K, 500 MHz).

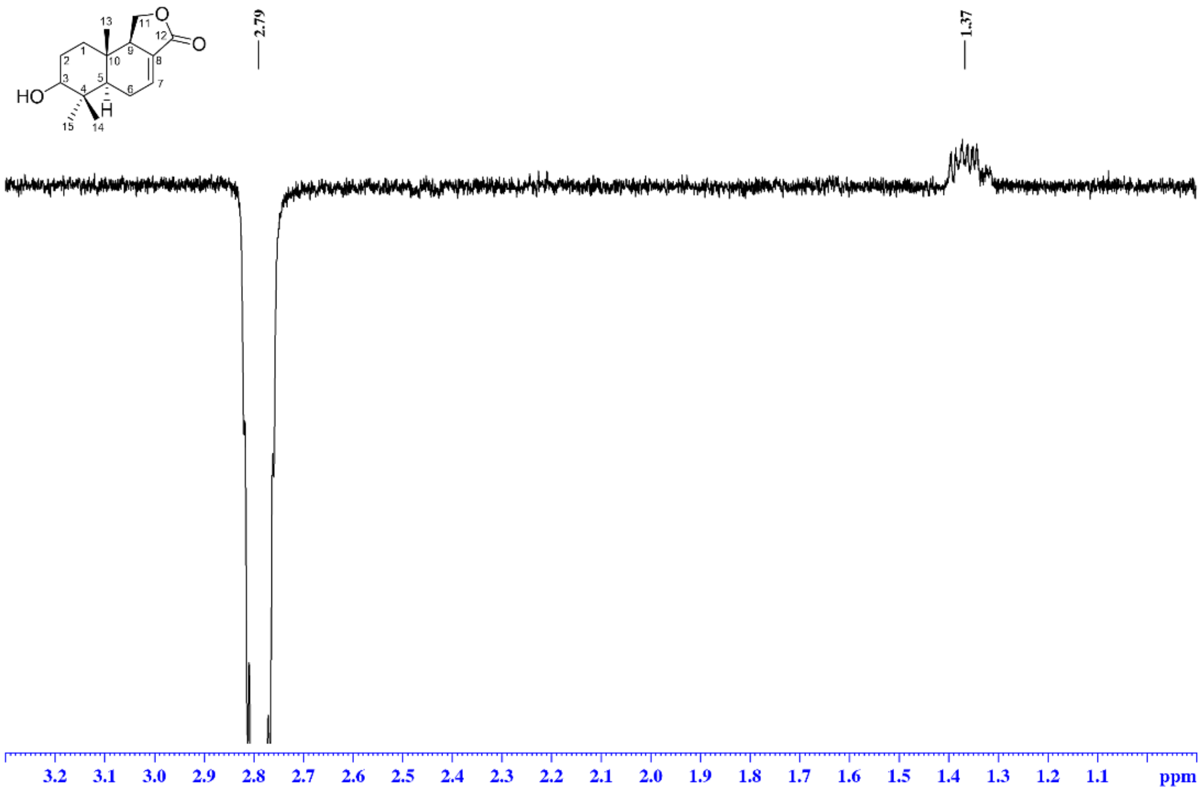

Figure NMR-S36. Selective NOE spectrum of compound **5** at 2.79 ppm ($CDCl_3$, 300 K, 500 MHz).

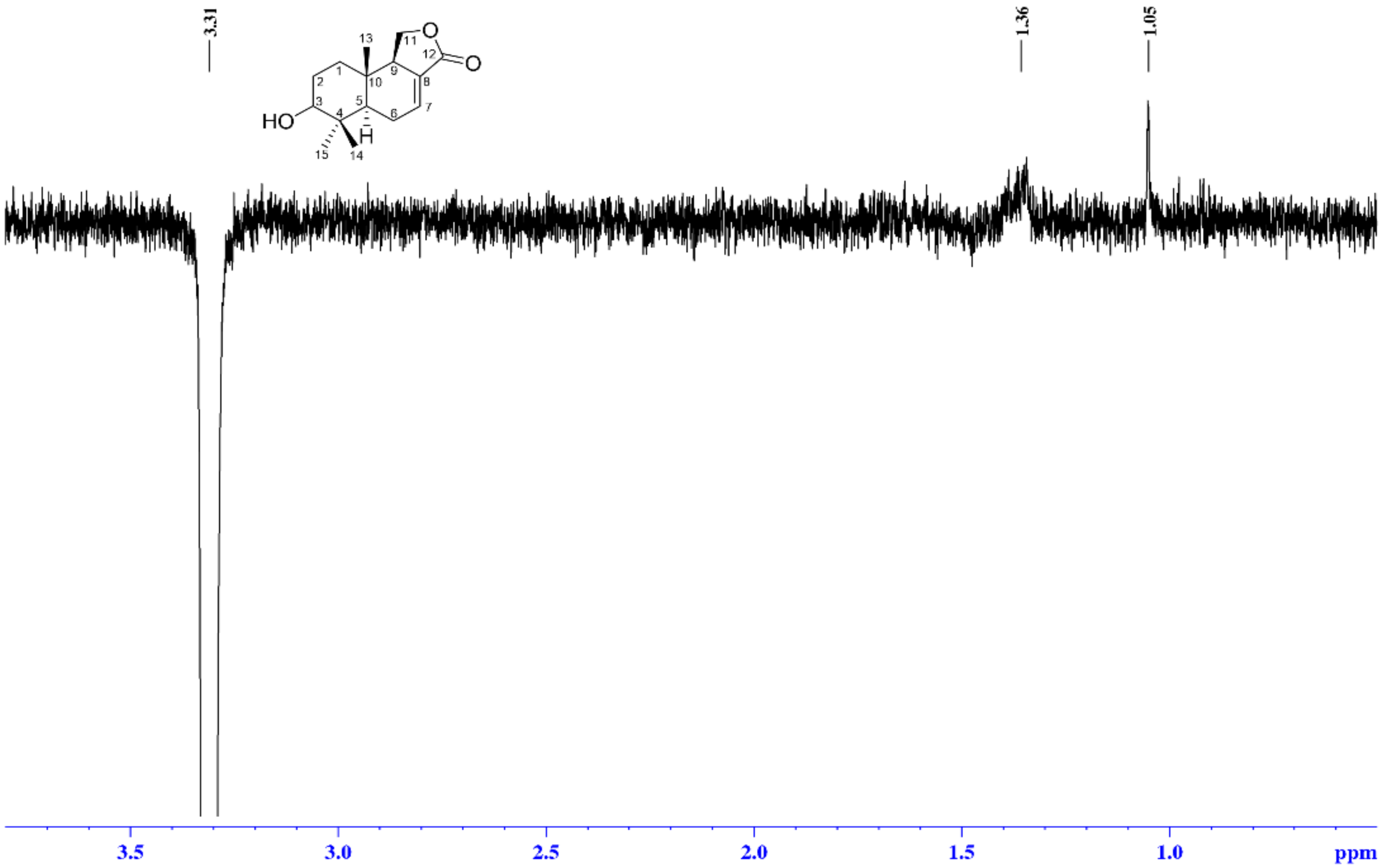

Figure NMR-S37. Selective NOE spectrum of compound **5** at 3.31 ppm ($CDCl_3$, 300 K, 500 MHz).

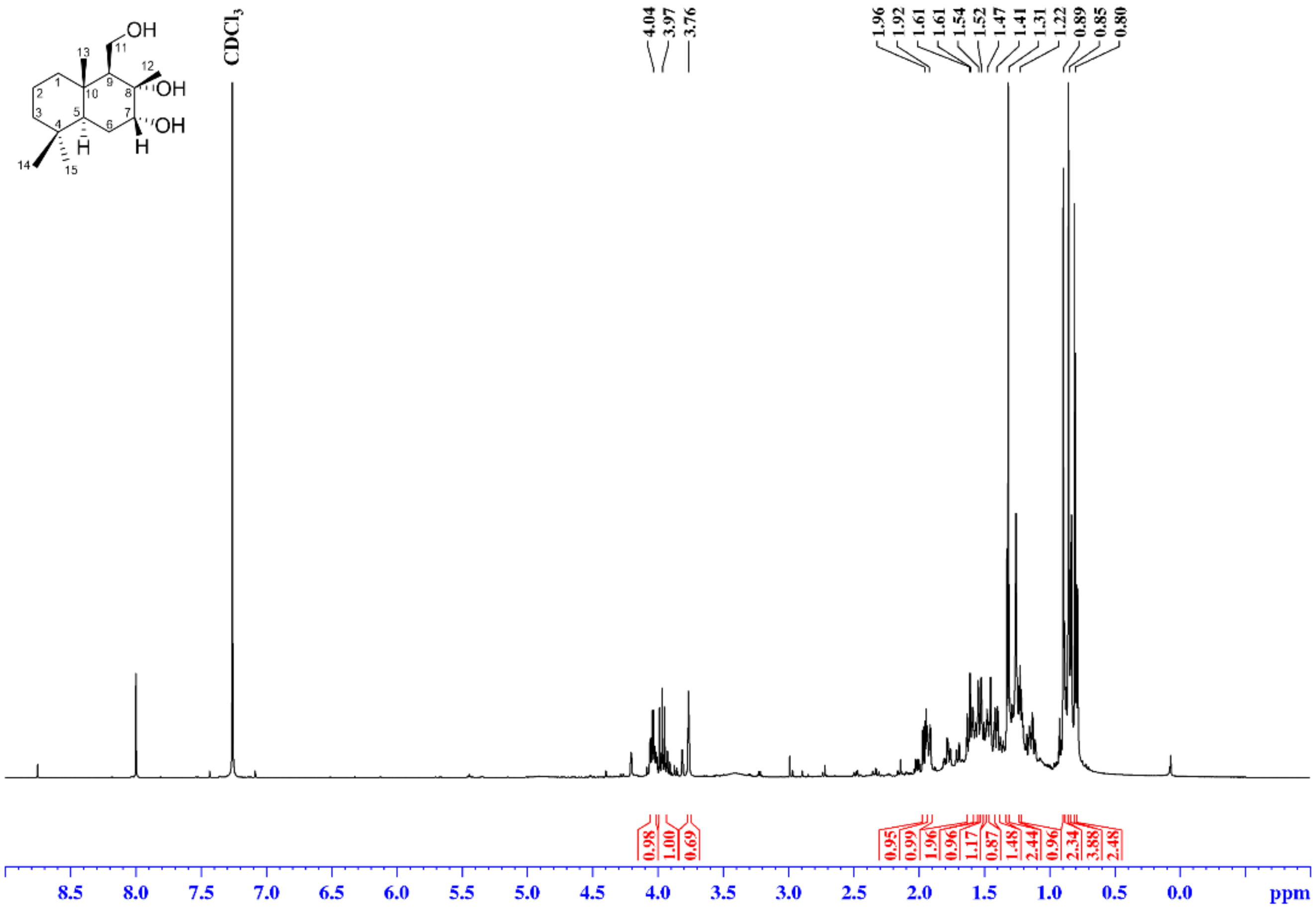

Figure NMR-S38. ^{1}H NMR spectrum of compound **6** ($CDCl_3$, 300 K, 600 MHz).

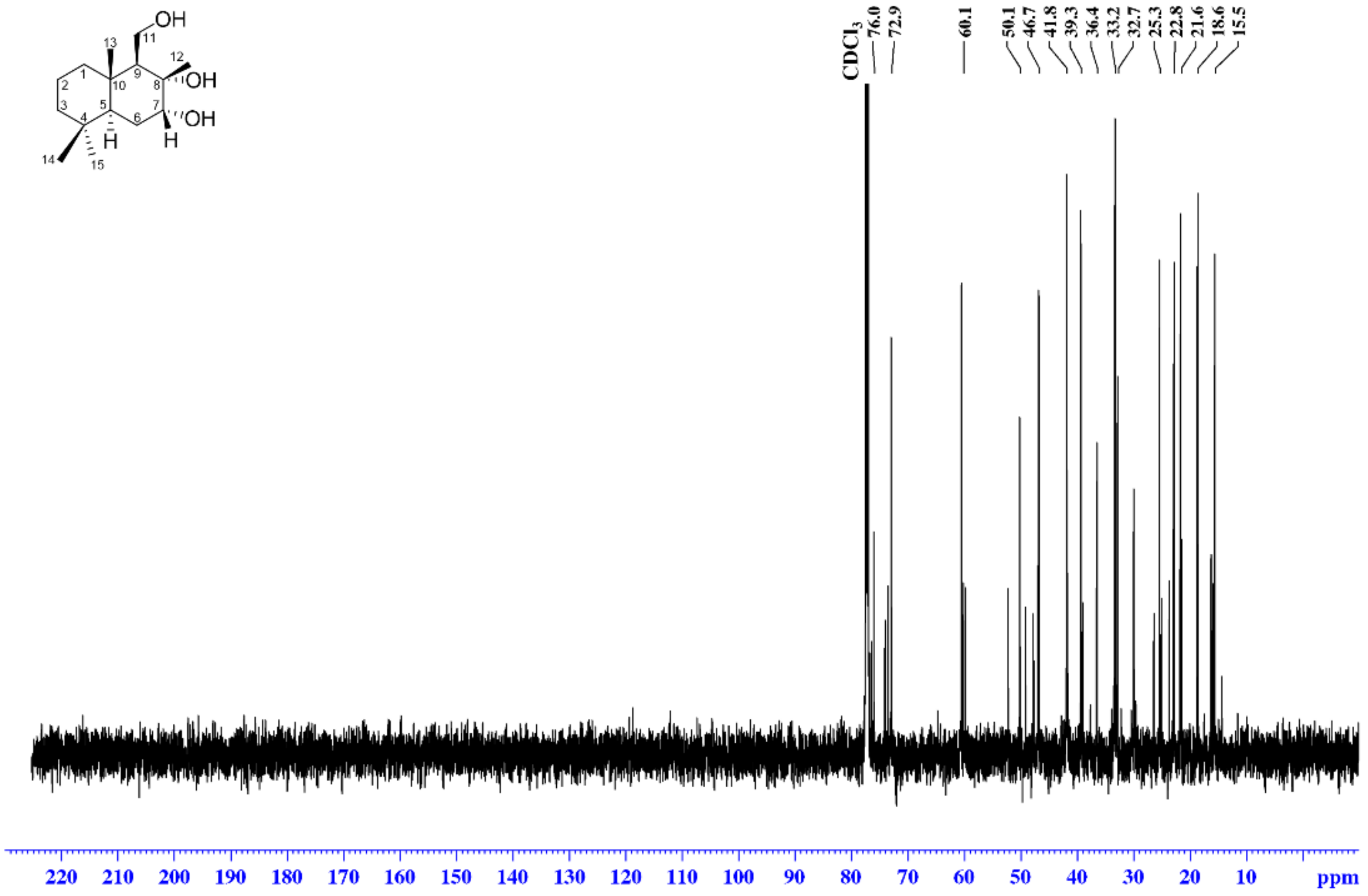

Figure NMR-S39. ^{13}C NMR spectrum of compound **6** ($CDCl_3$, 300 K, 150 MHz).

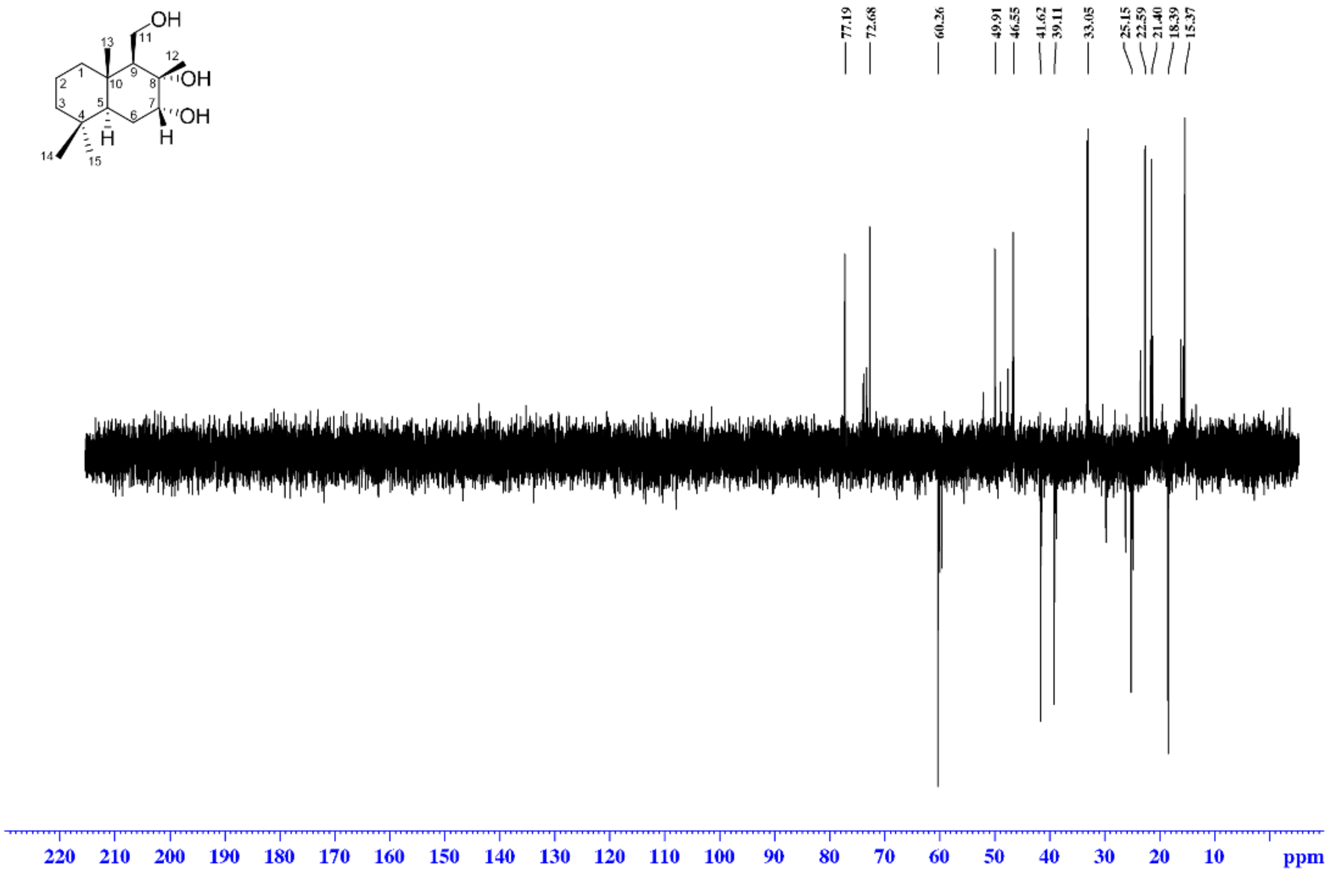

Figure NMR-S40. ^{13}C NMR DEPT spectrum of compound **6** ($CDCl_3$, 300 K, 150 MHz).

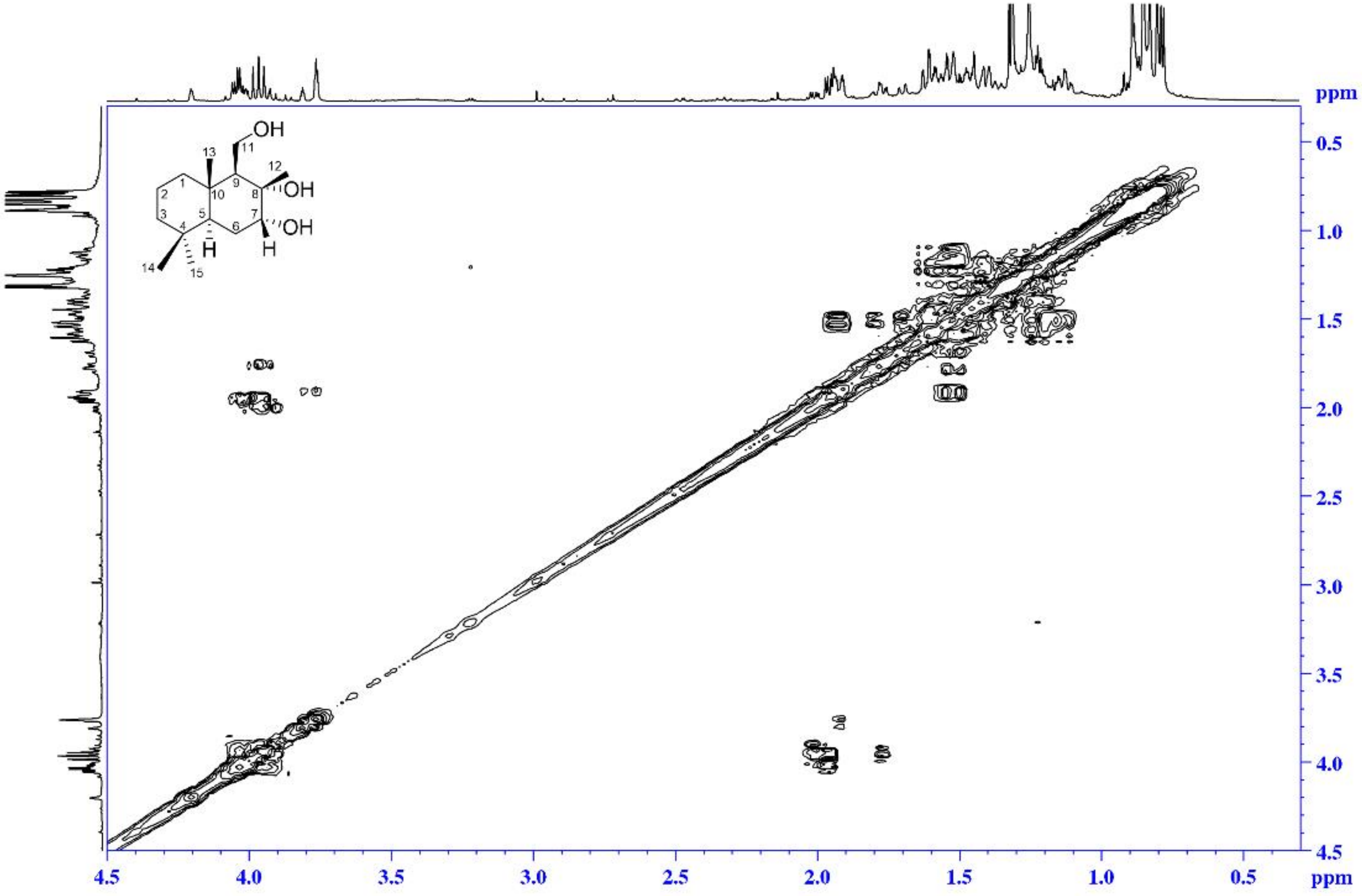

Figure NMR-S41. COSY spectrum of compound **6** ($CDCl_3$, 300 K, 600 MHz).

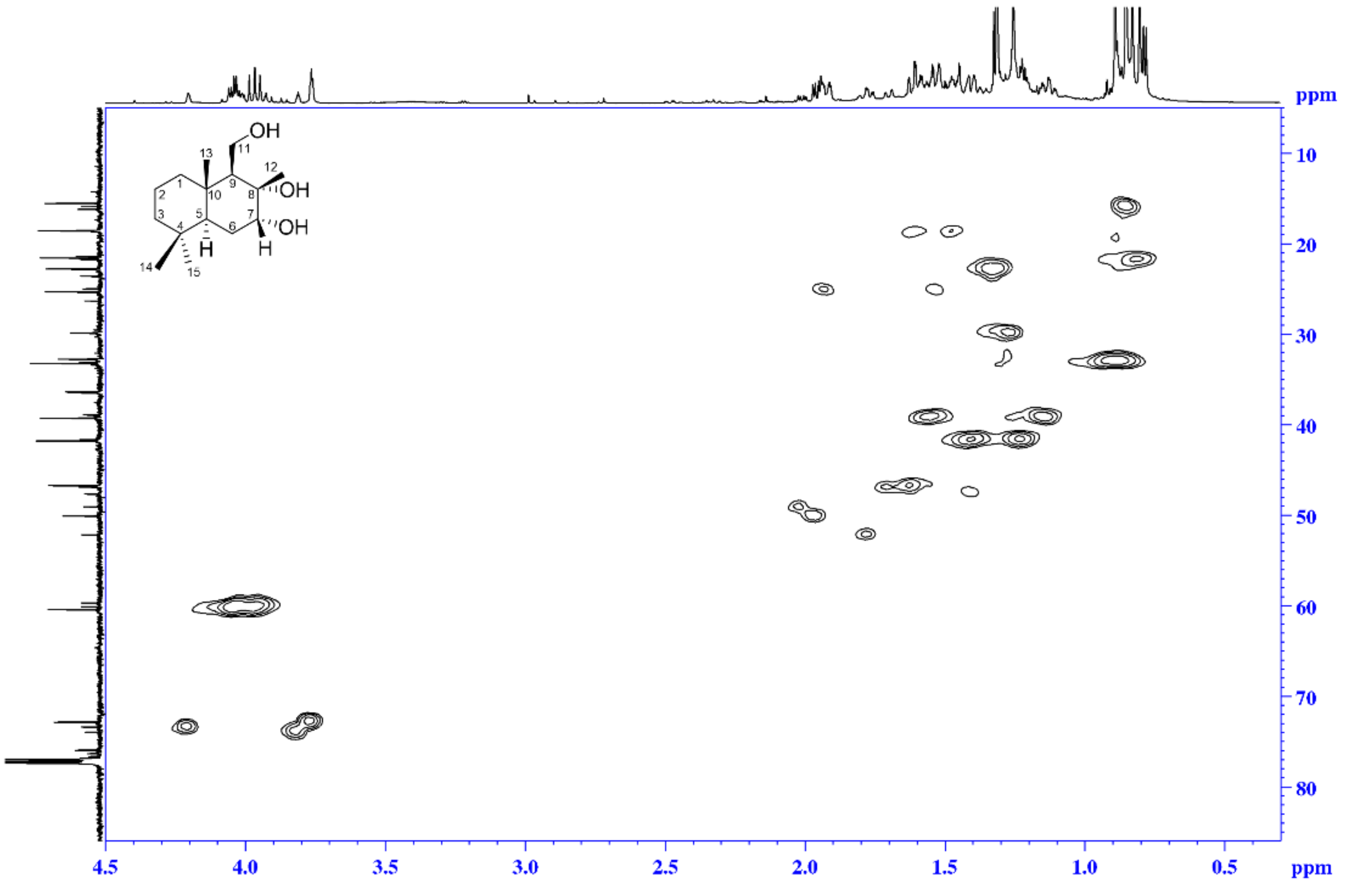

Figure NMR-S42. HSQC spectrum of compound **6** ($CDCl_3$, 300 K, 600 MHz).

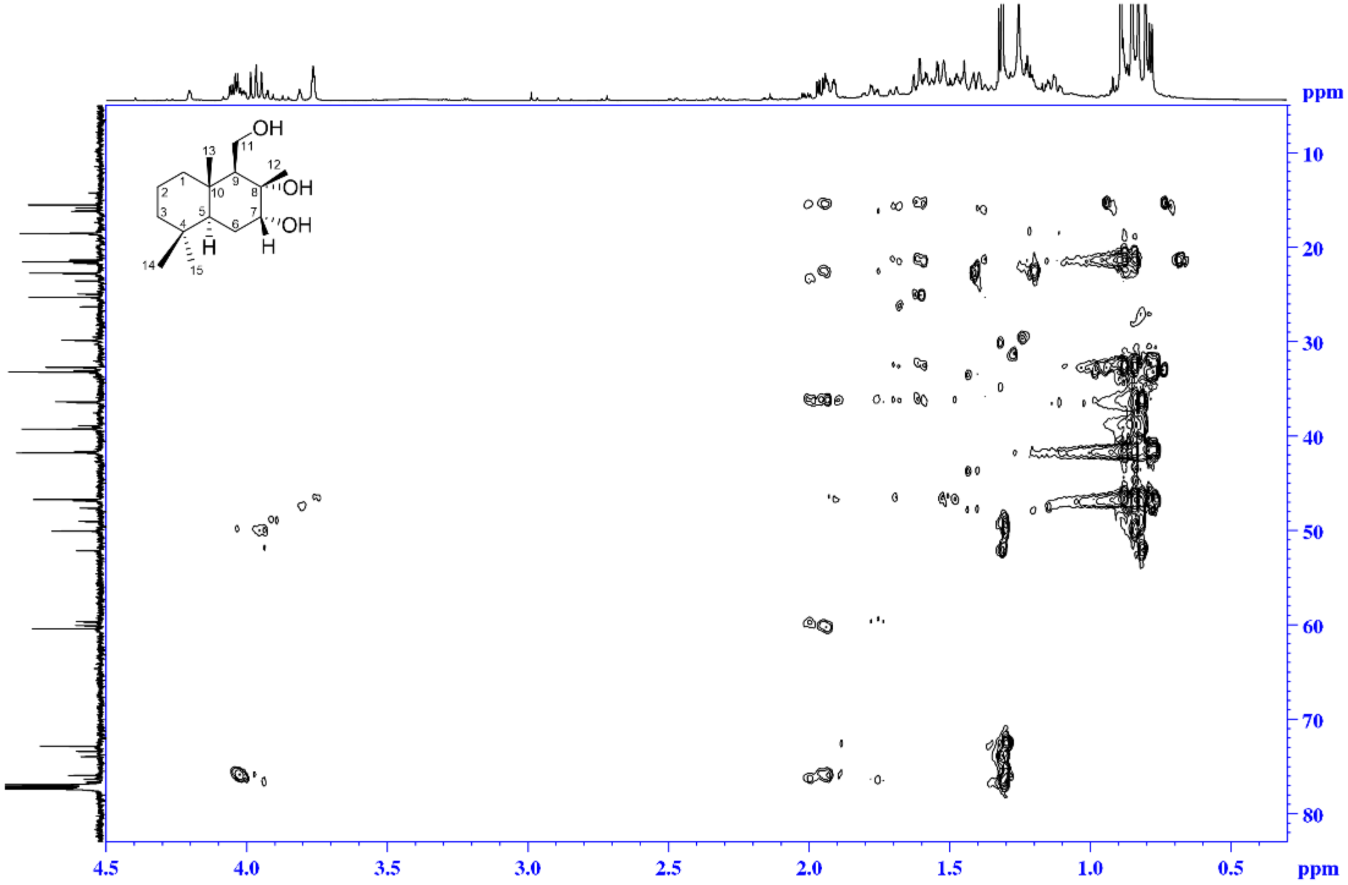

Figure NMR-S43. HMBC spectrum of compound **6** ($CDCl_3$, 300 K, 600 MHz).

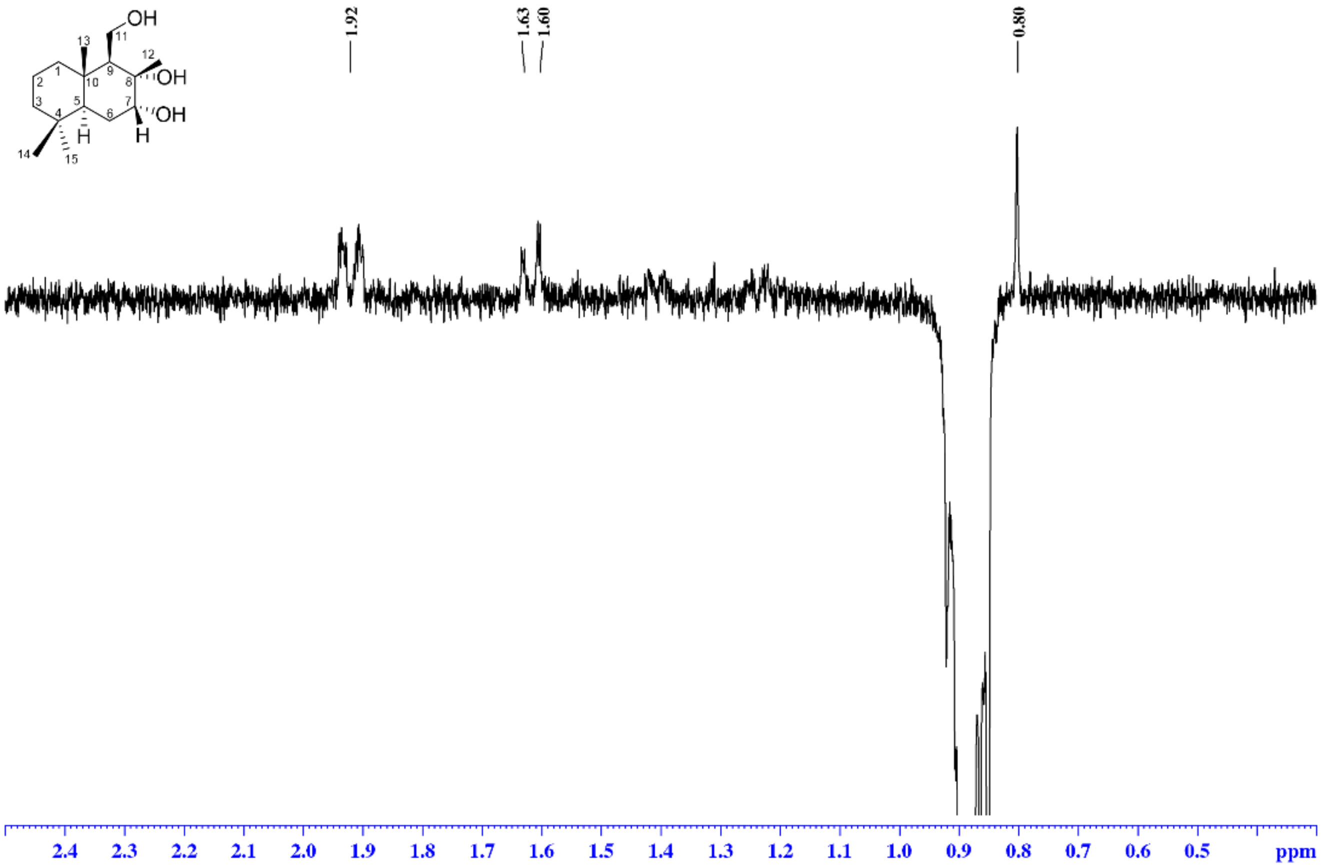

Figure NMR-S44. Selective NOE spectrum of compound **6** at 0.89 ppm ($CDCl_3$, 300 K, 500 MHz).

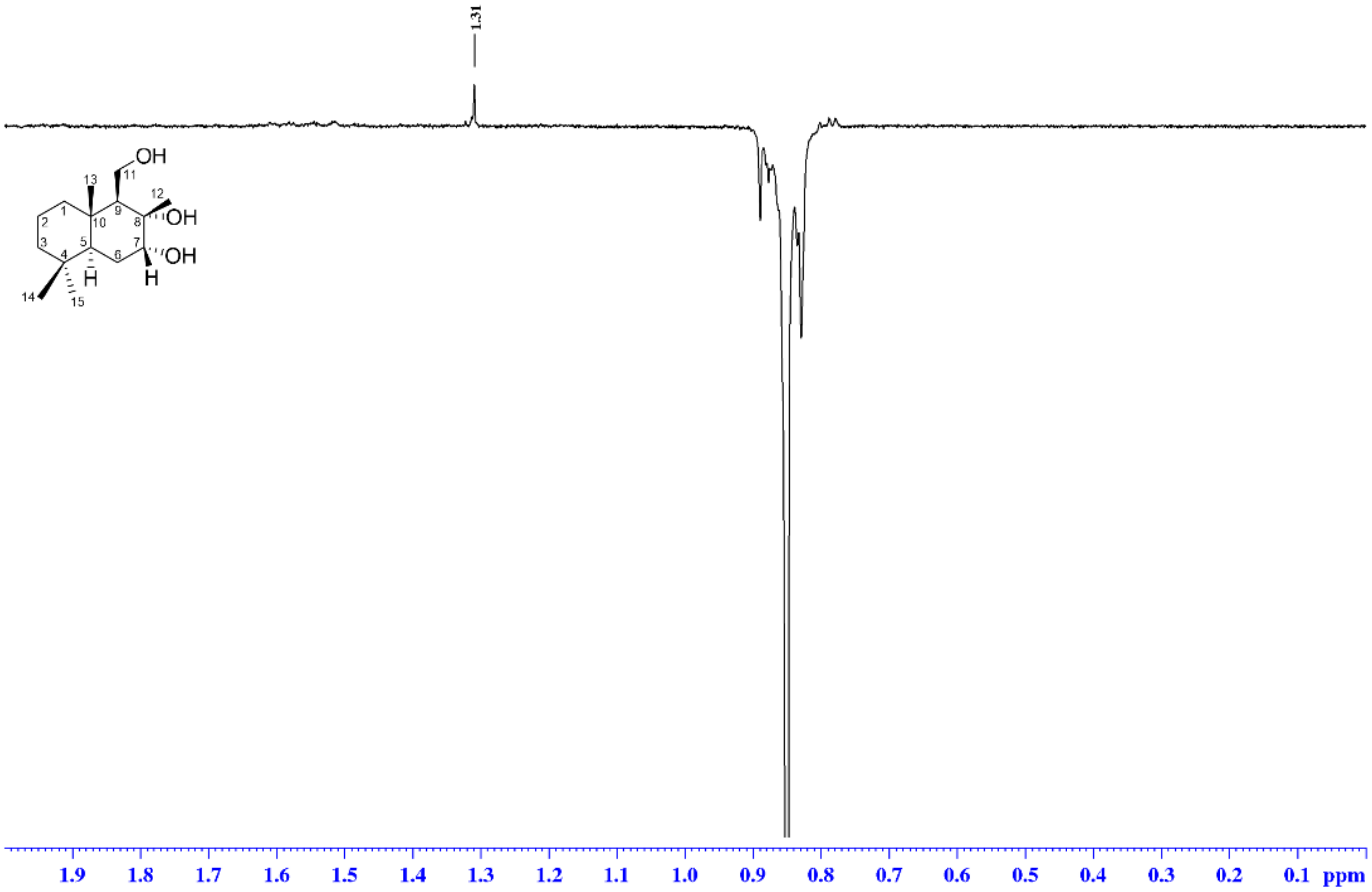

Figure NMR-S45. Selective NOE spectrum of compound **6** at 0.85 ppm ($CDCl_3$, 300 K, 500 MHz).

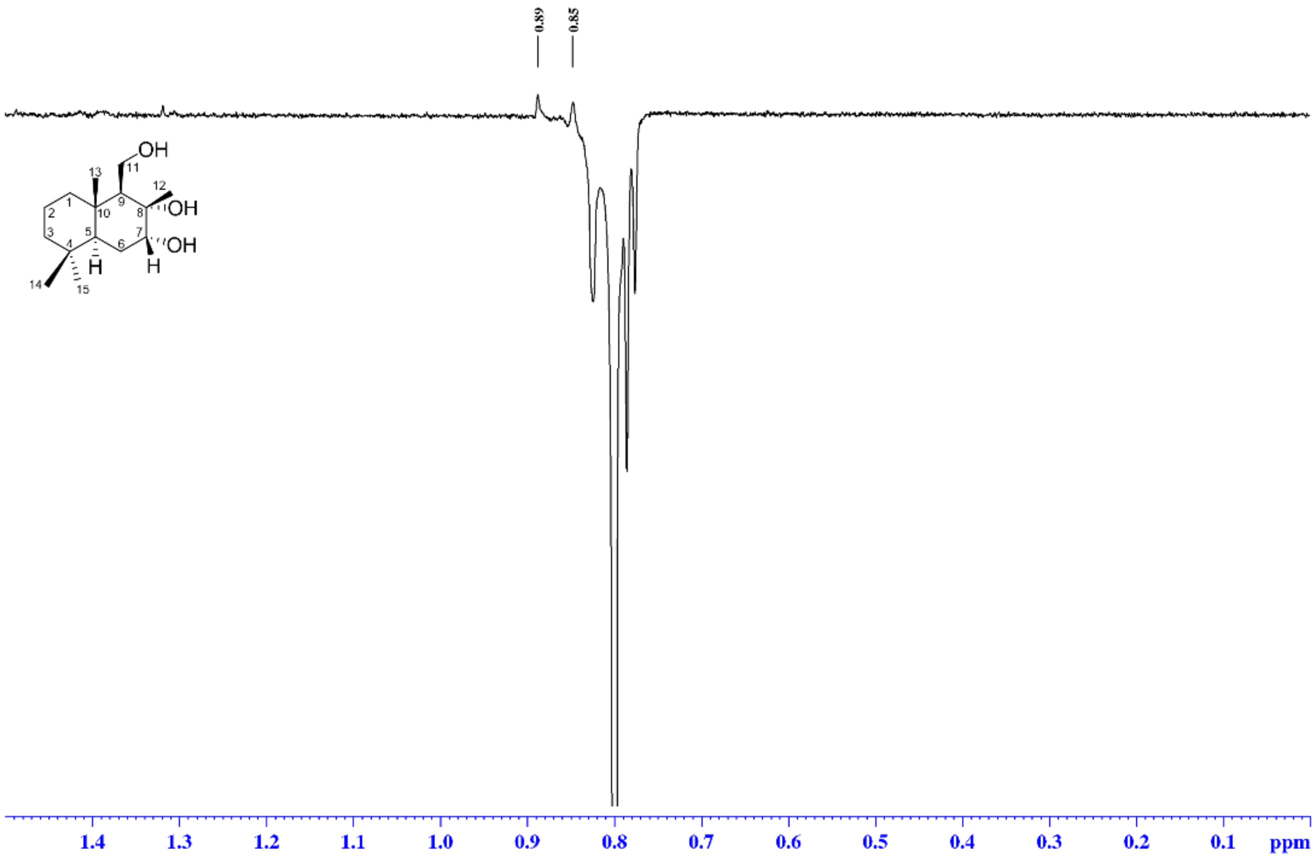

Figure NMR-S46. Selective NOE spectrum of compound **6** at 0.80 ppm ($CDCl_3$, 300 K, 500 MHz).

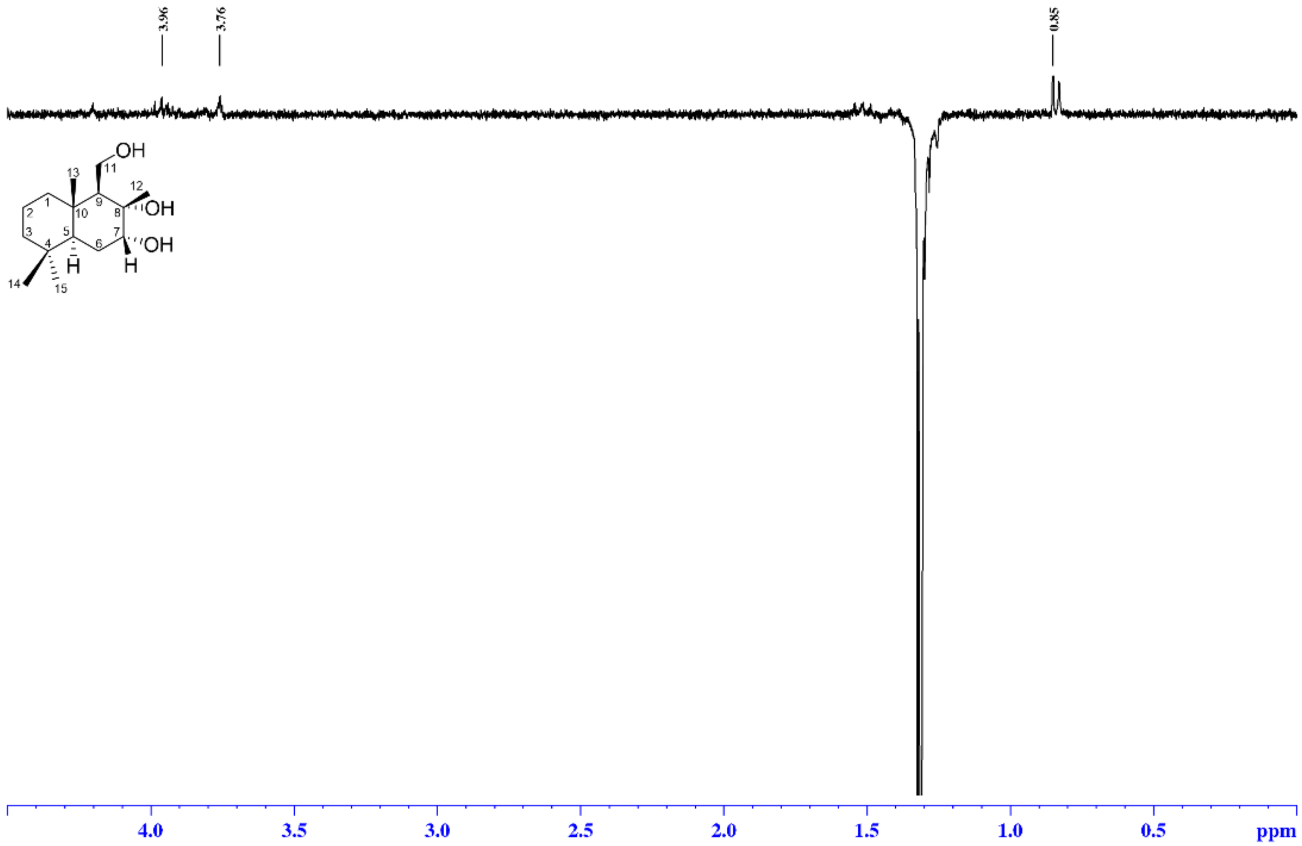

Figure NMR-S47. Selective NOE spectrum of compound **6** at 1.31 ppm ($CDCl_3$, 300 K, 500 MHz).

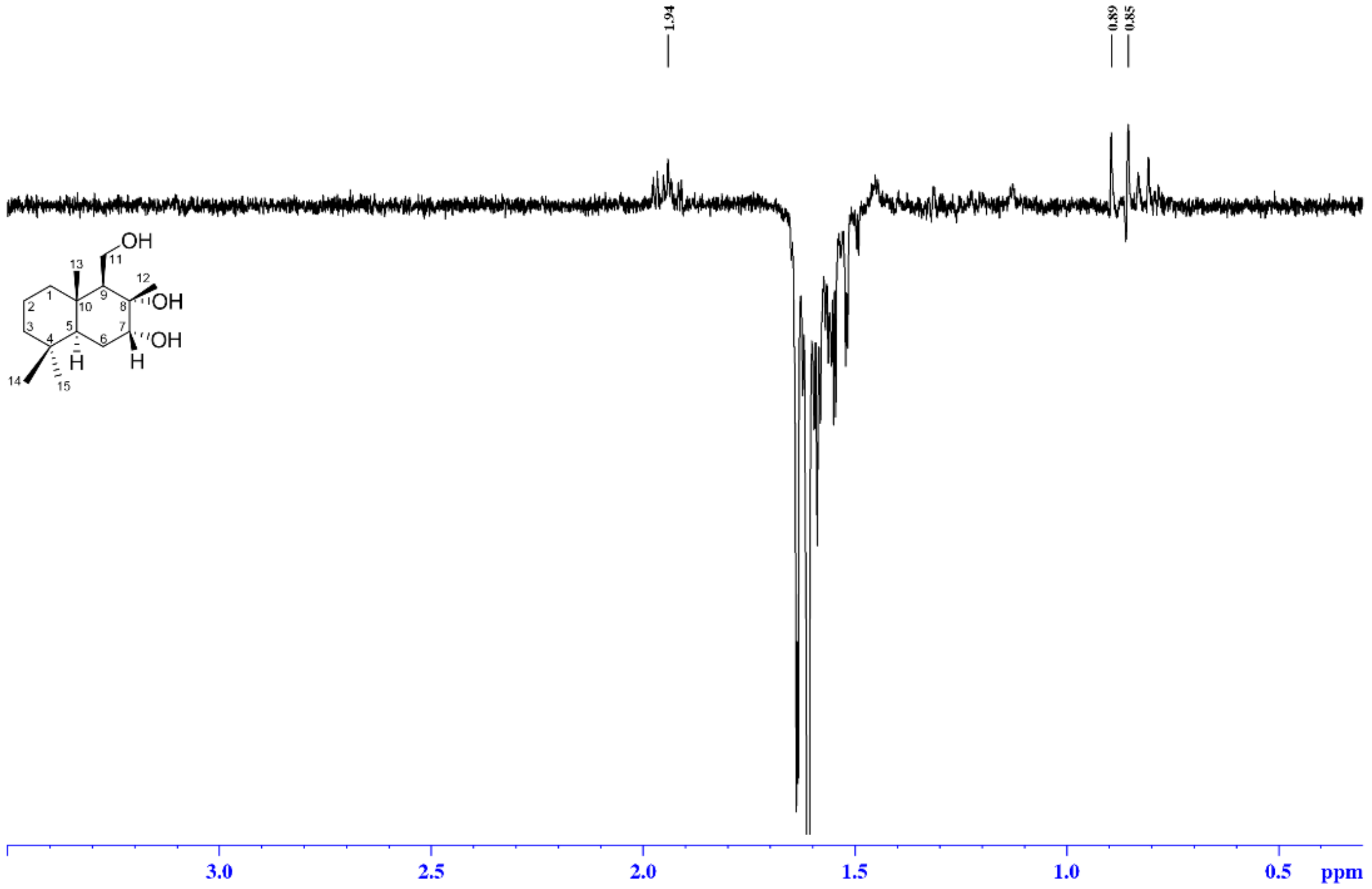

Figure NMR-S48. Selective NOE spectrum of compound **6** at 1.61 ppm ($CDCl_3$, 300 K, 500 MHz).

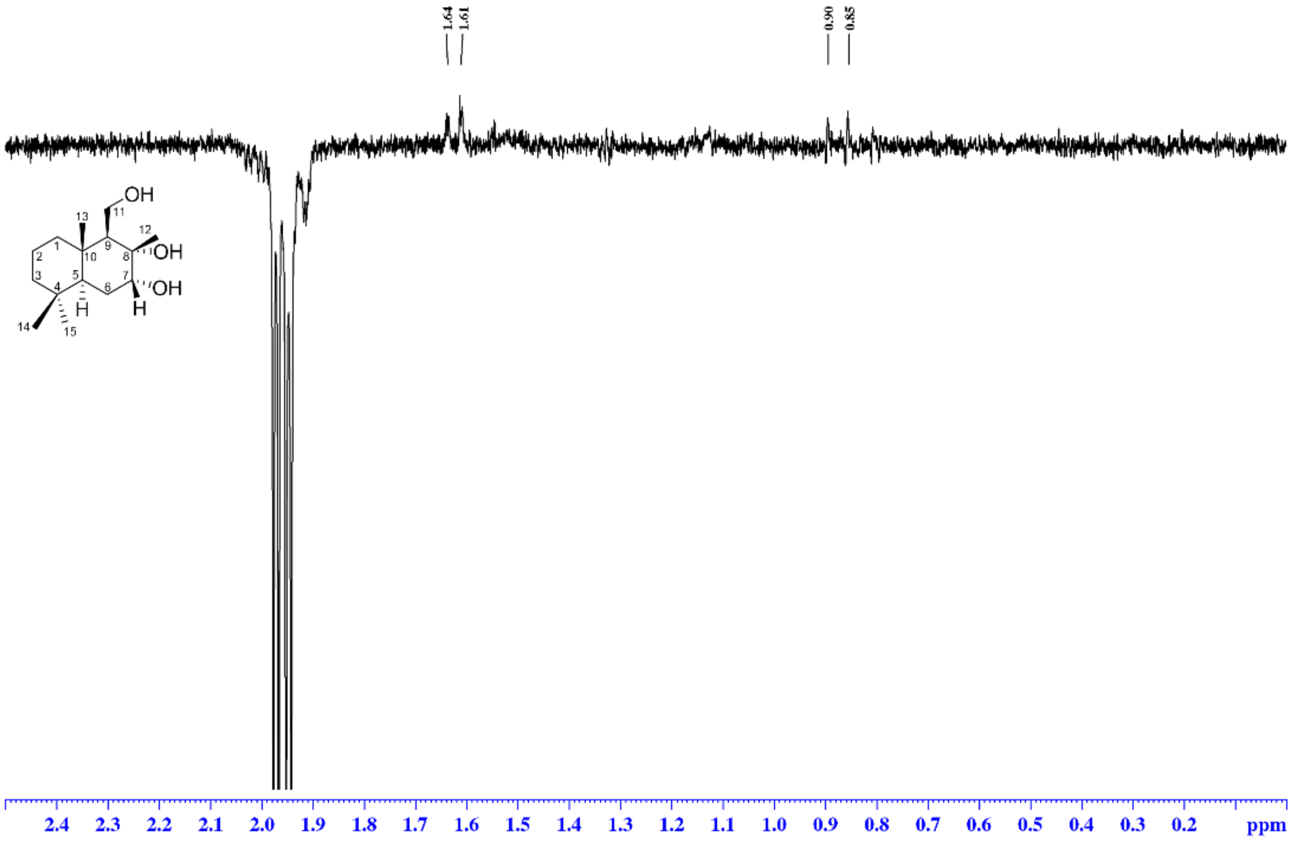

Figure NMR-S49. Selective NOE spectrum of compound **6** at 1.96 ppm ($CDCl_3$, 300 K, 500 MHz).

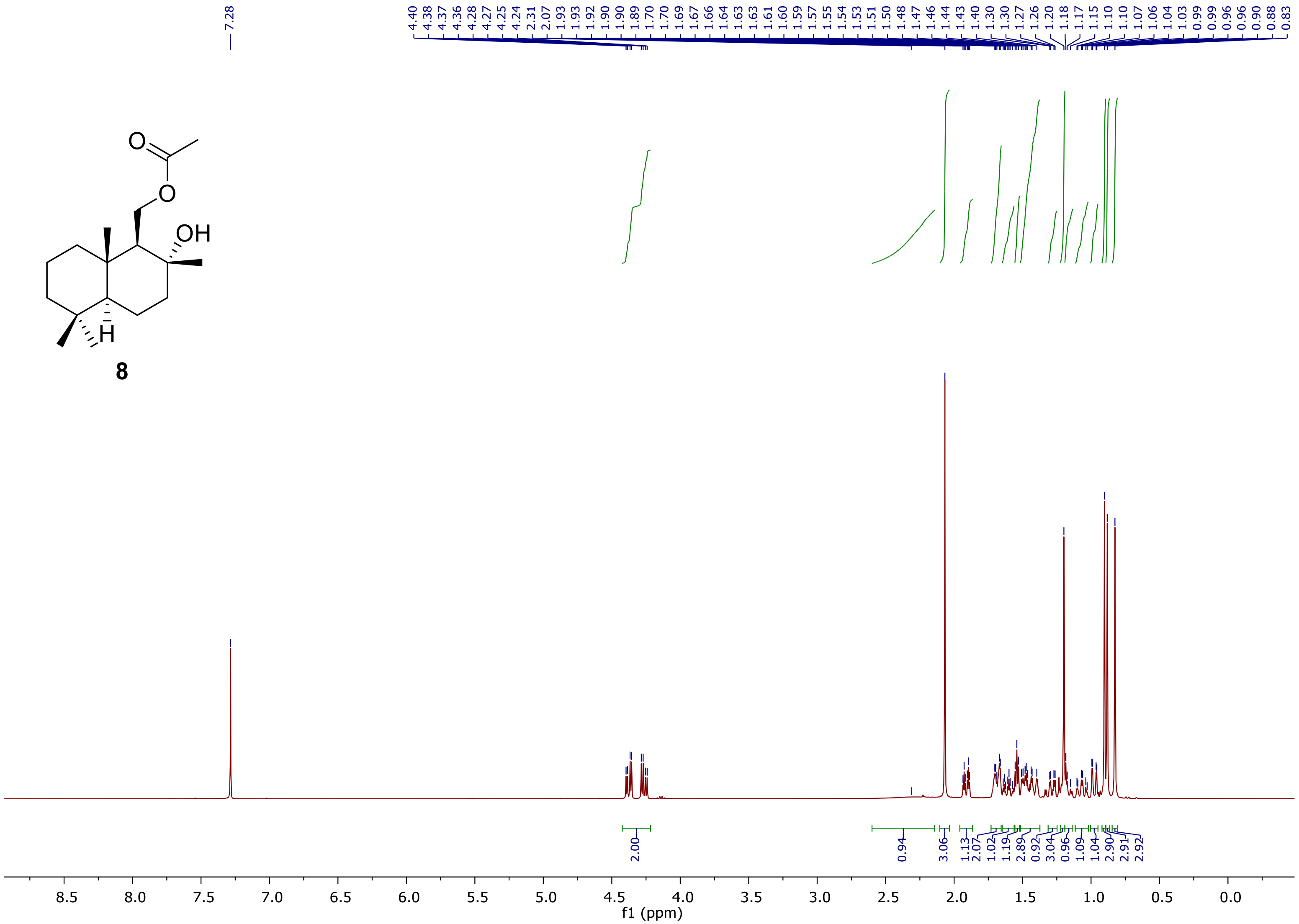

Figure NMR-S50. ^{1}H NMR spectrum of compound ((1*S*,2*R*,8a*S*)-2-hydroxy-2,5,5,8a-tetramethyldecahydronaphthalen-1-yl)methyl acetate (**8**).

Supplementary Data 1

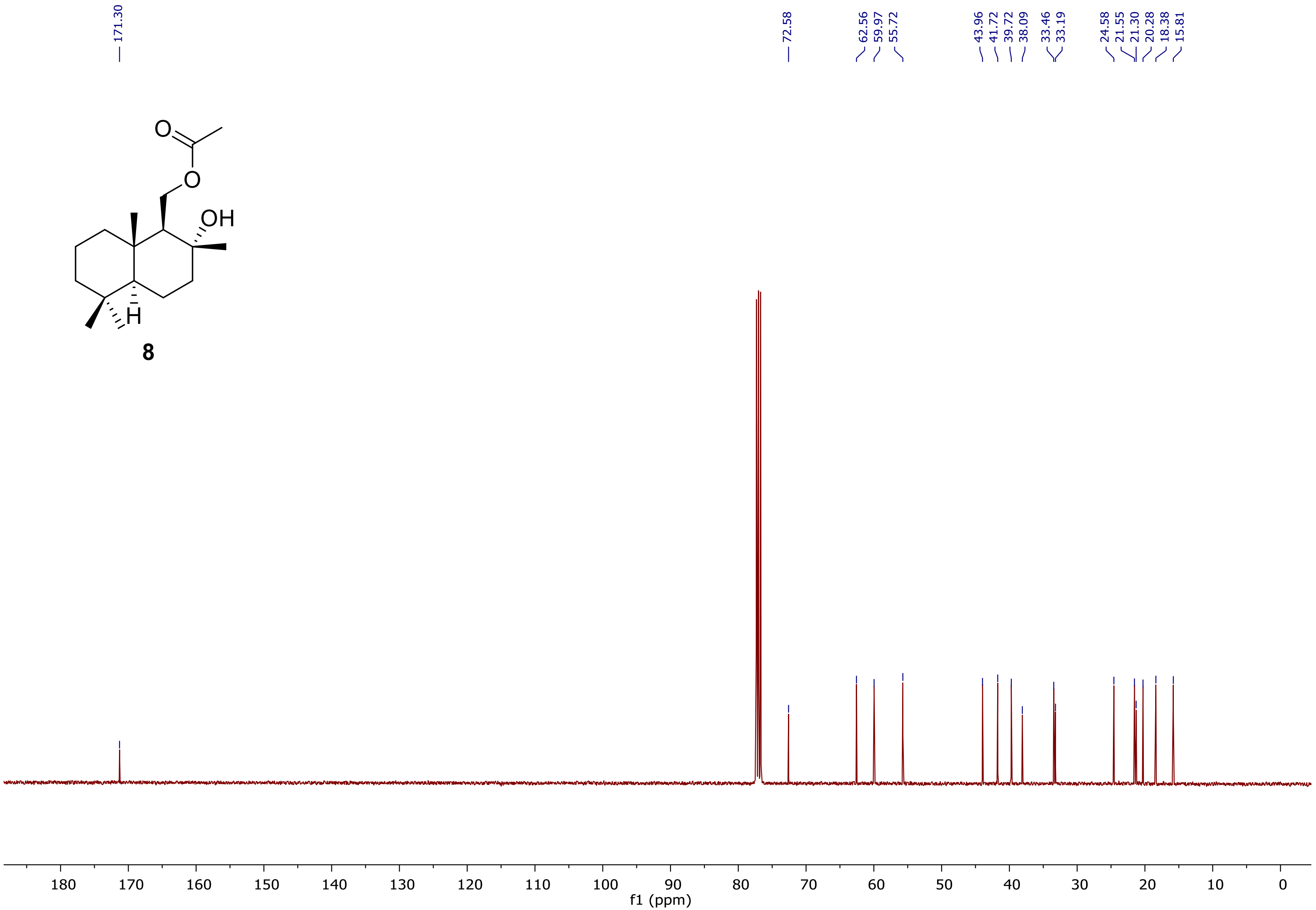

Figure NMR-S51. ^{13}C NMR spectrum of compound **8**.

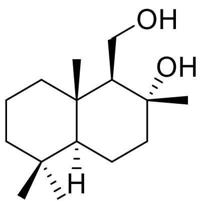

9

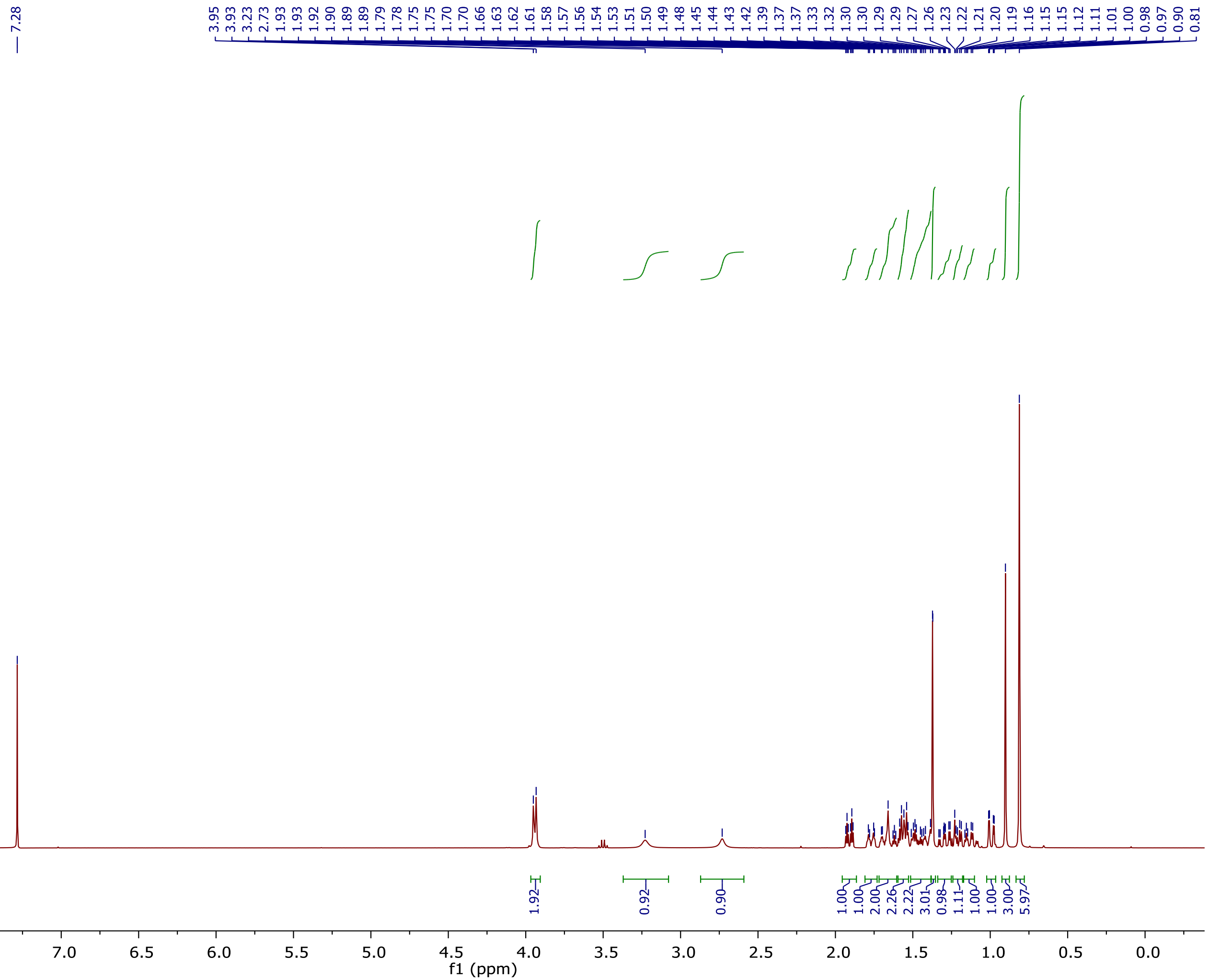

Figure NMR-S52. ^{1}H NMR spectrum of compound (1*S*,2*R*,8a*S*)-1-(hydroxymethyl)-2,5,5,8a-tetramethyldecahydronaphthalen-2-ol (**9**).

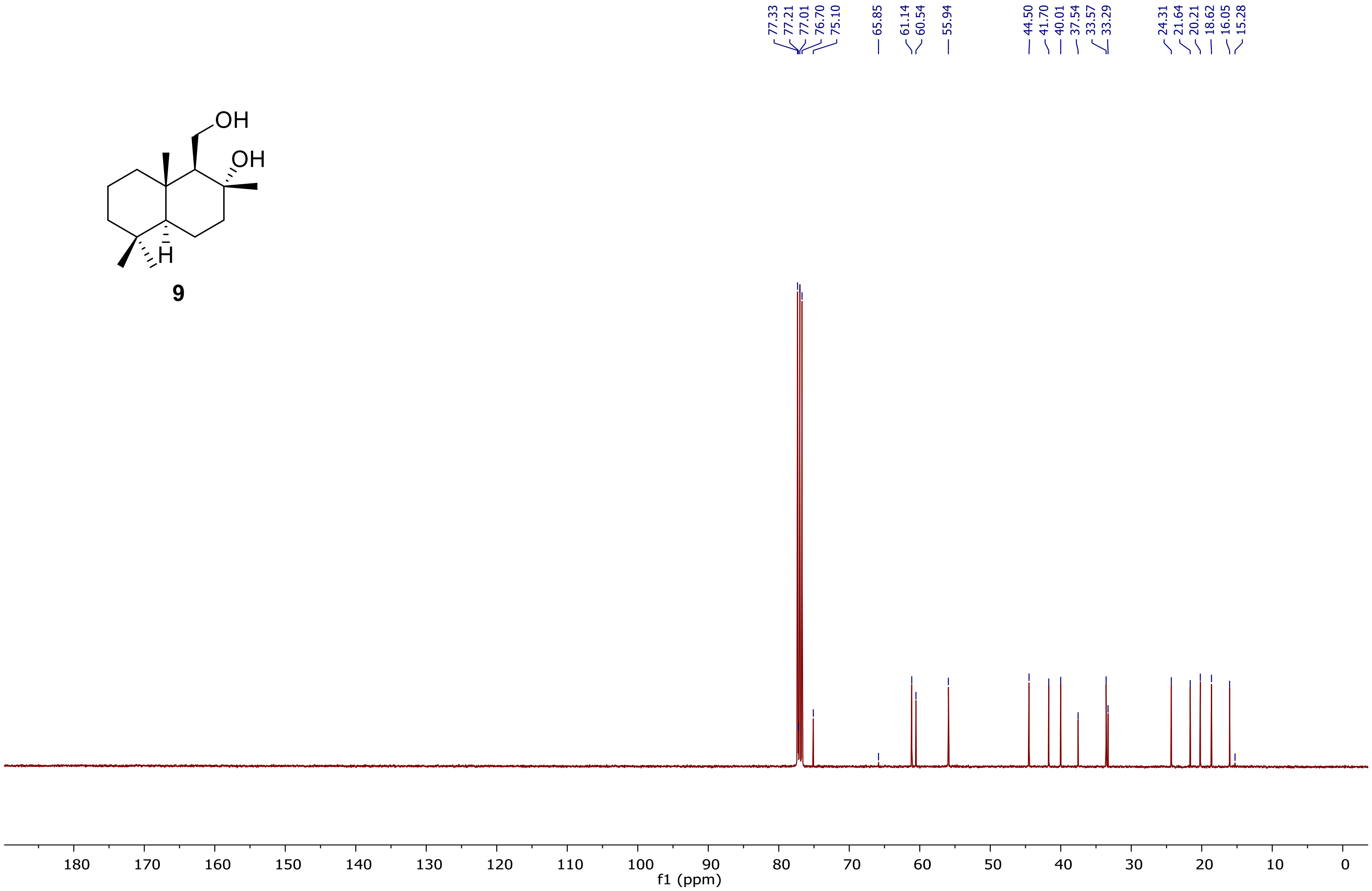

Figure NMR-S53. ^{13}C NMR spectrum of compound **9**.

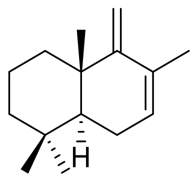

10

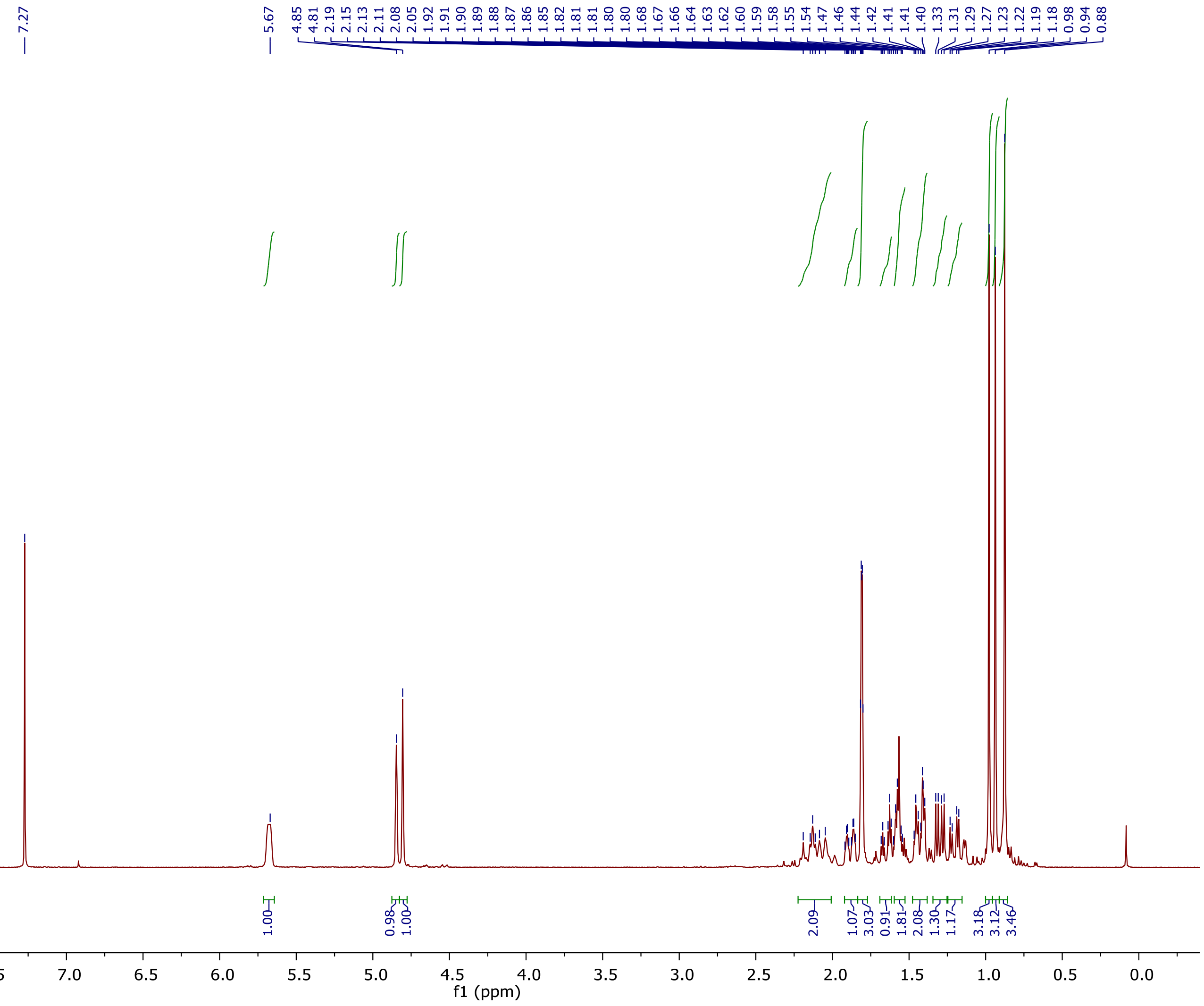

Figure NMR-S54. ^{1}H NMR spectrum of compound (4a*S*)-1,1,4a,6-tetramethyl-5-methylene-1,2,3,4,4a,5,8,8a-octahydronaphthalene (**10**).

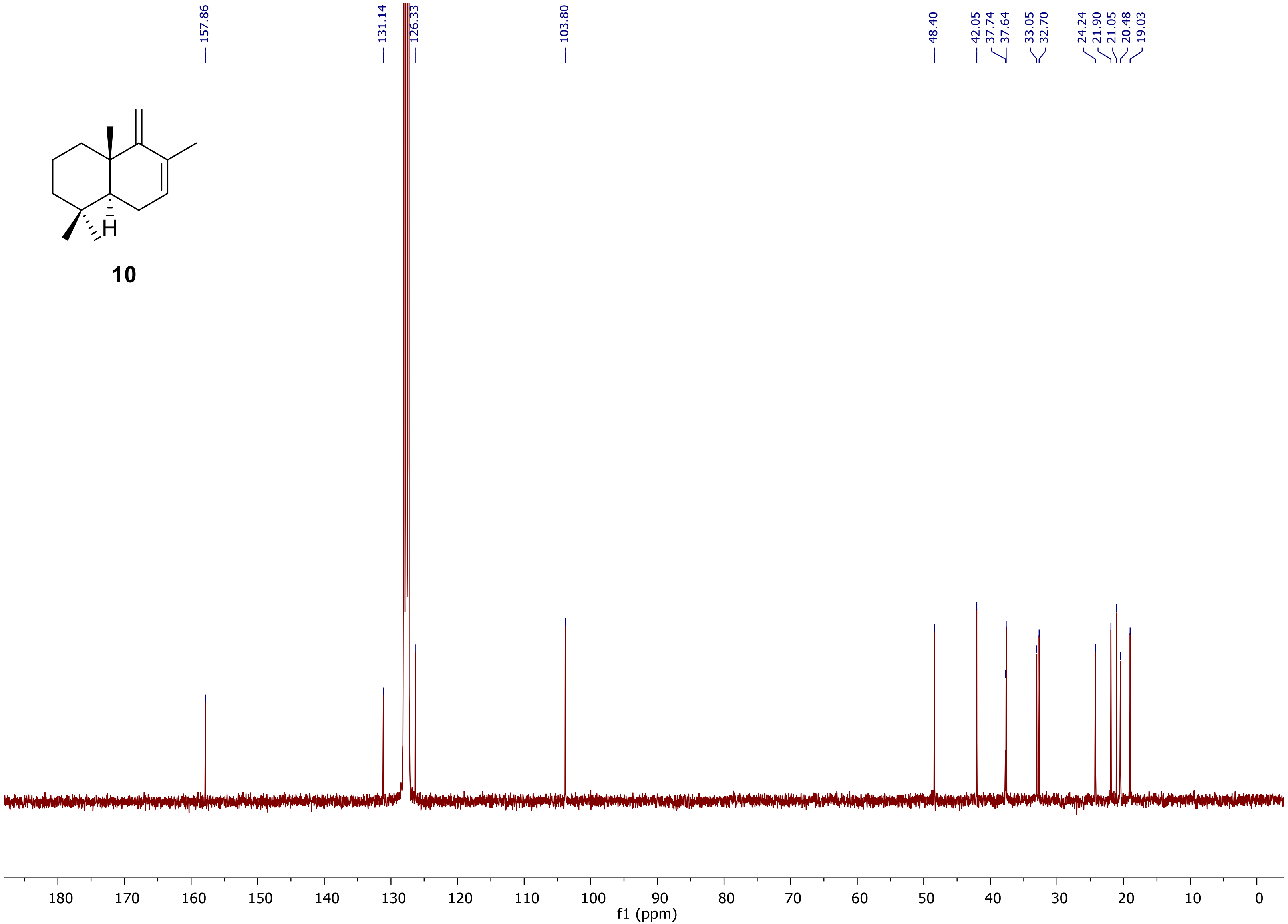

Figure NMR-S55. ^{13}C NMR spectrum of compound **10**.

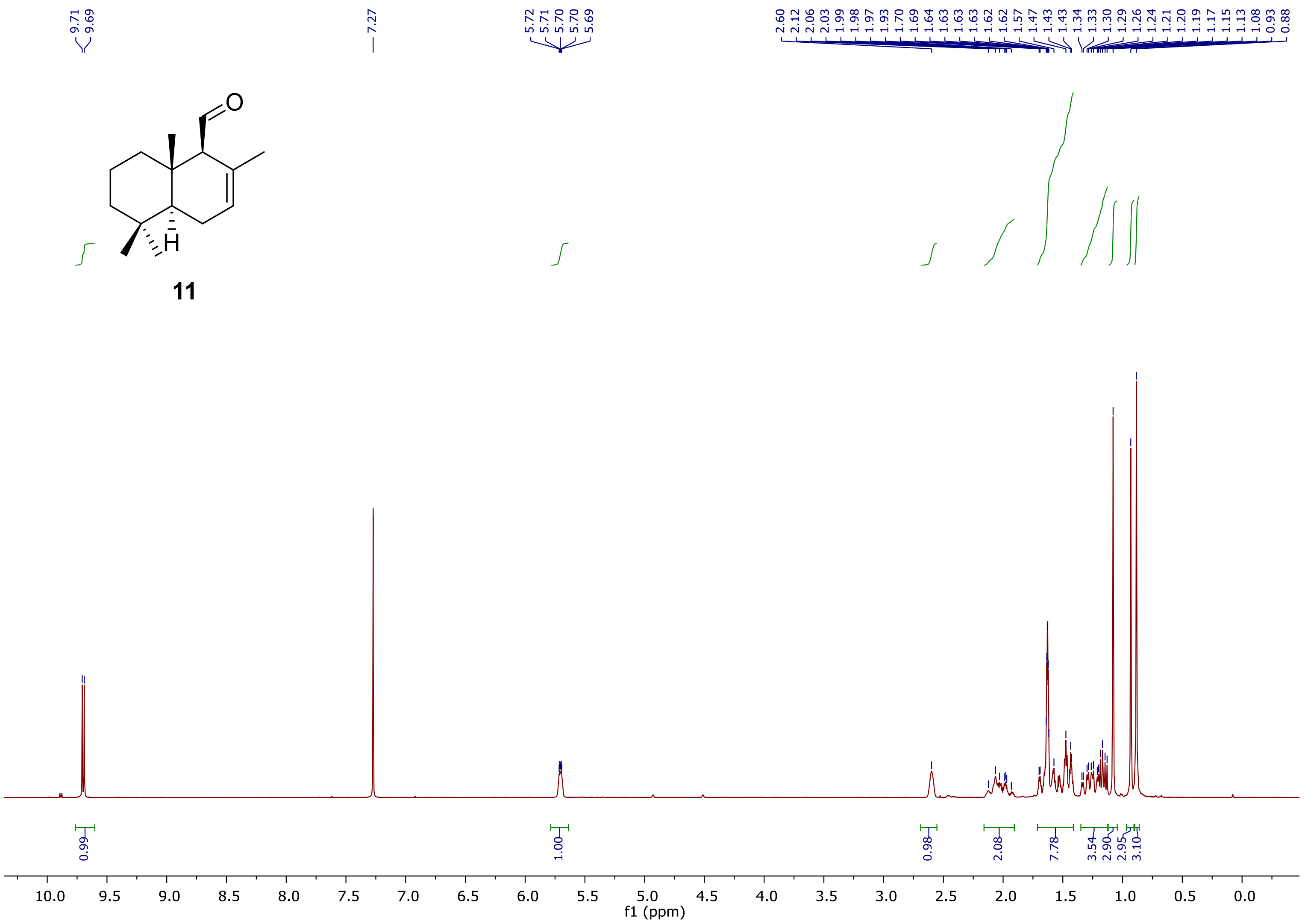

Figure NMR-S56. ^{1}H NMR spectrum of compound (1*S*,8a*S*)-2,5,5,8a-tetramethyl-1,4,4a,5,6,7,8,8a-octahydronaphthalene-1-carbaldehyde (**11**).

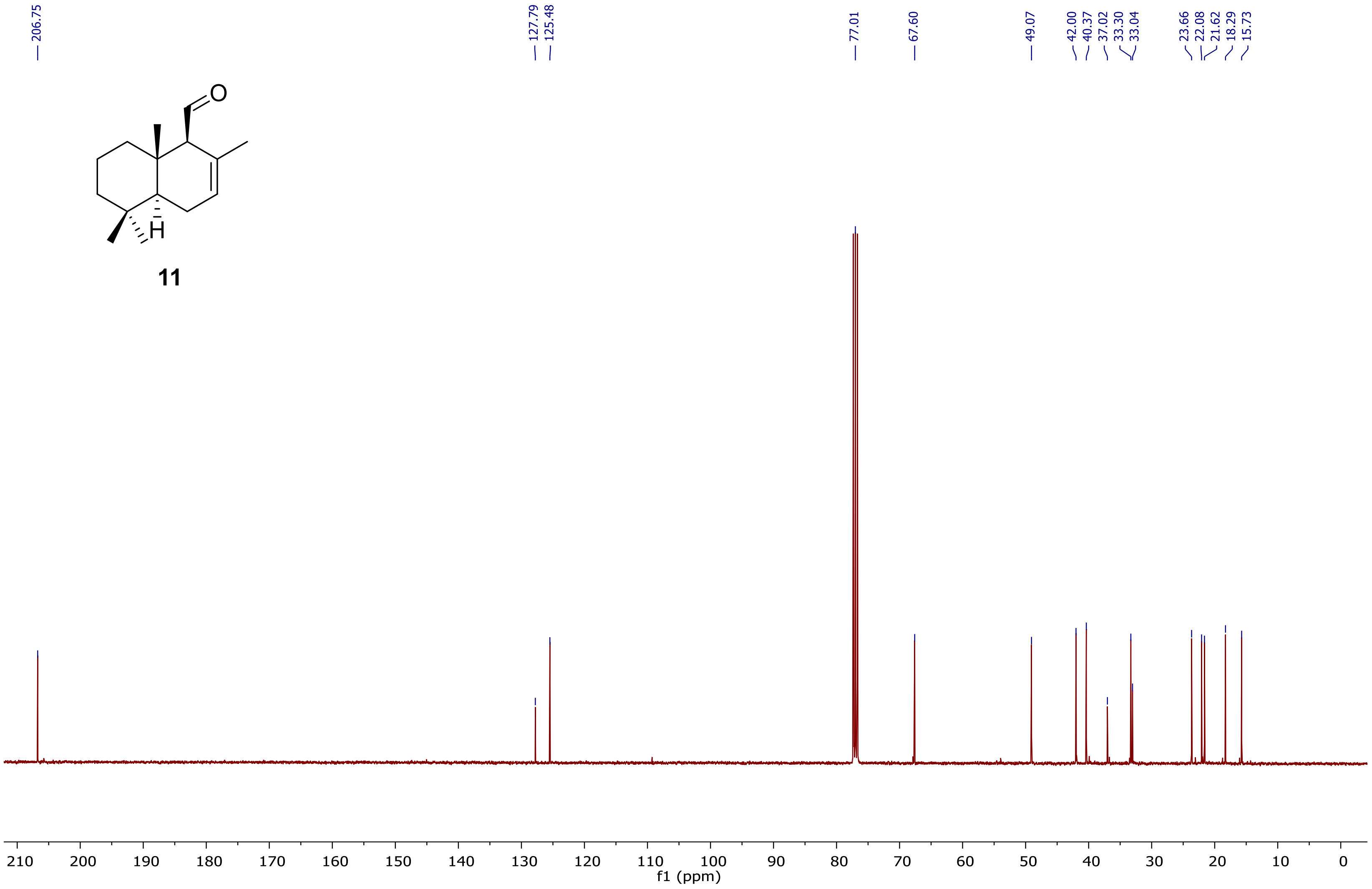

Figure NMR-S57. ^{13}C NMR spectrum of compound **11**.

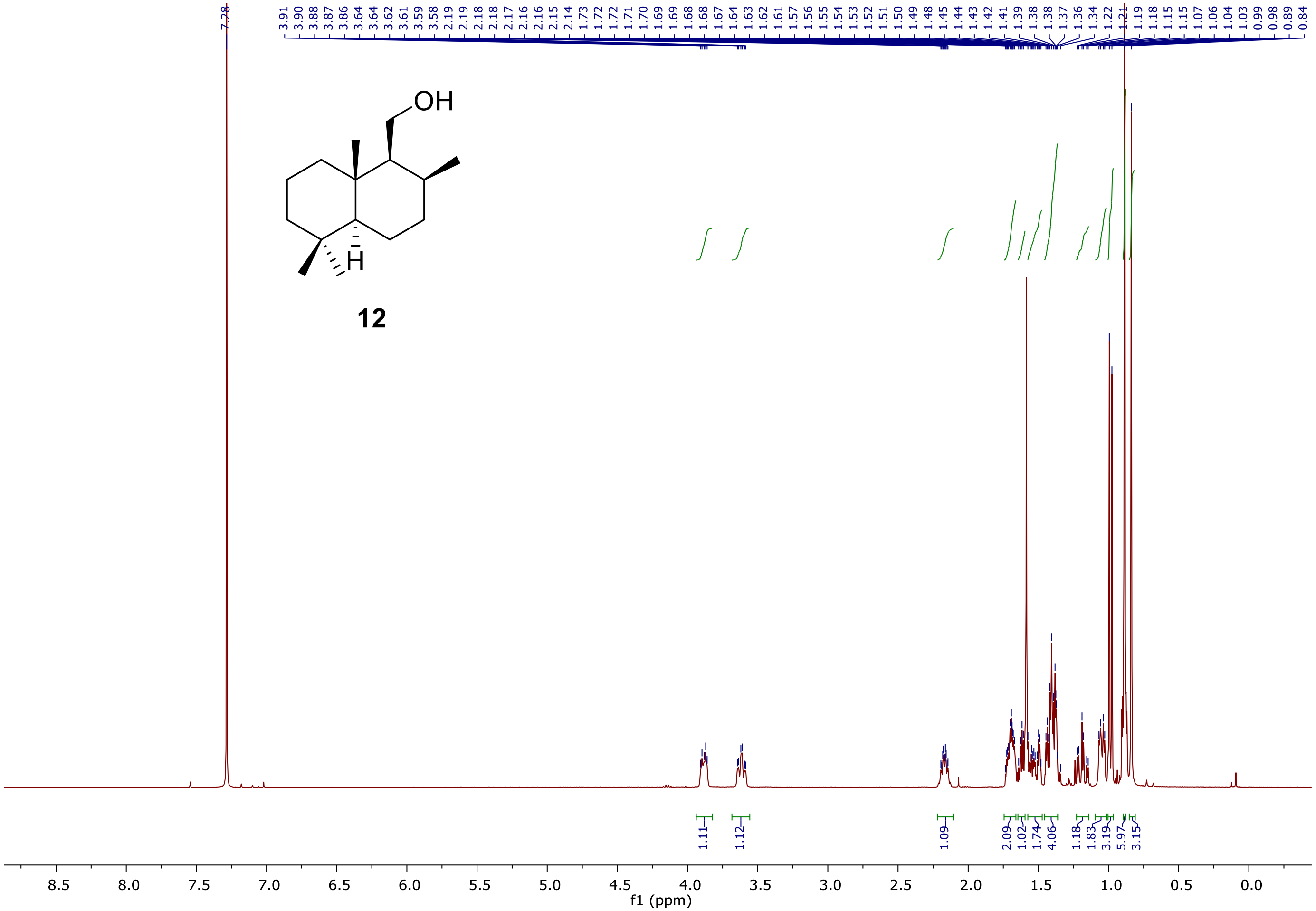

Figure NMR-S58. ^{1}H NMR spectrum of compound ((1*S*,2*S*,8a*S*)-2,5,5,8a-tetramethyldecahydronaphthalen-1-yl)methanol (**12**).

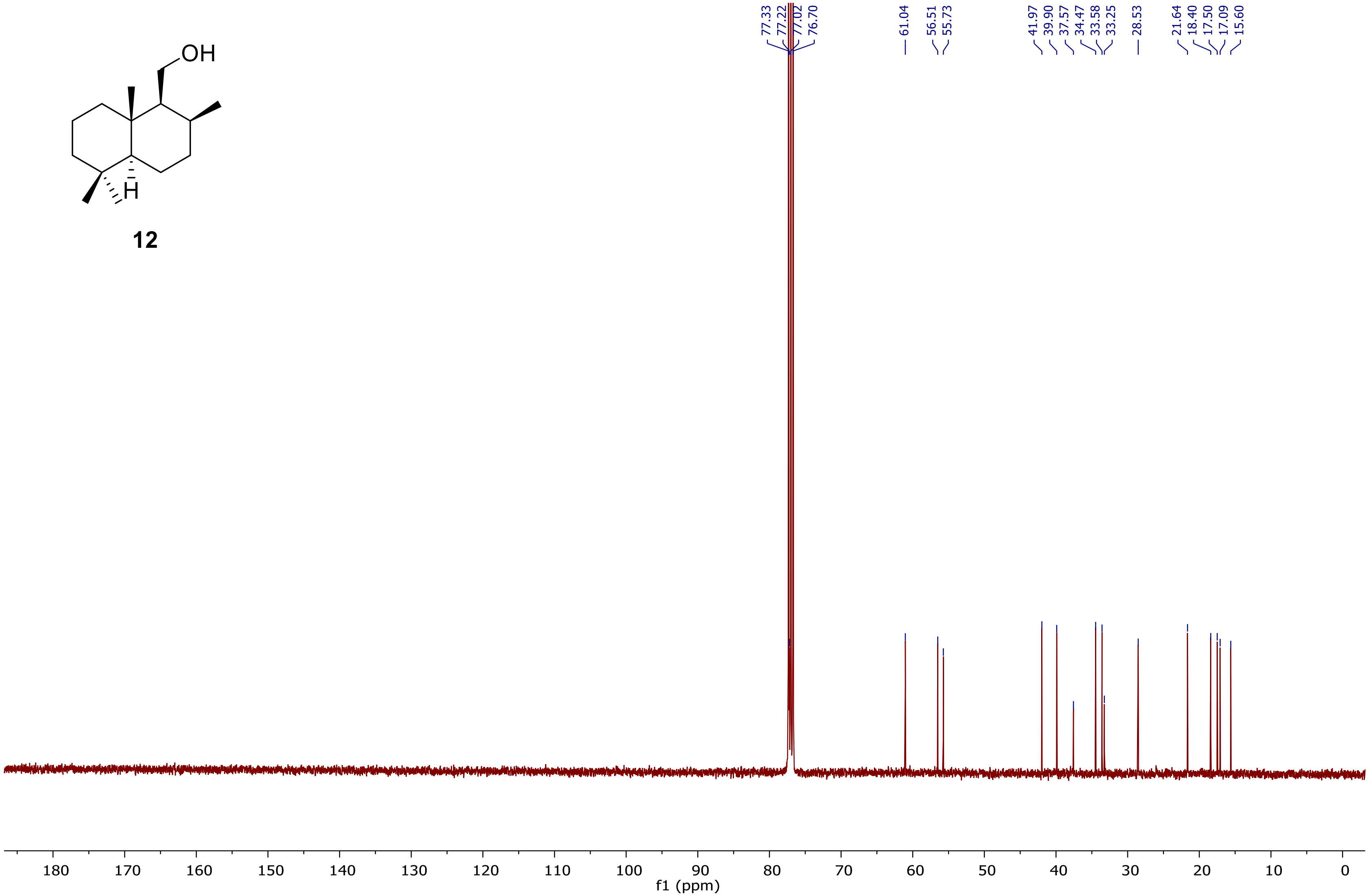

Figure NMR-S59. ^{13}C NMR spectrum of compound **12**.

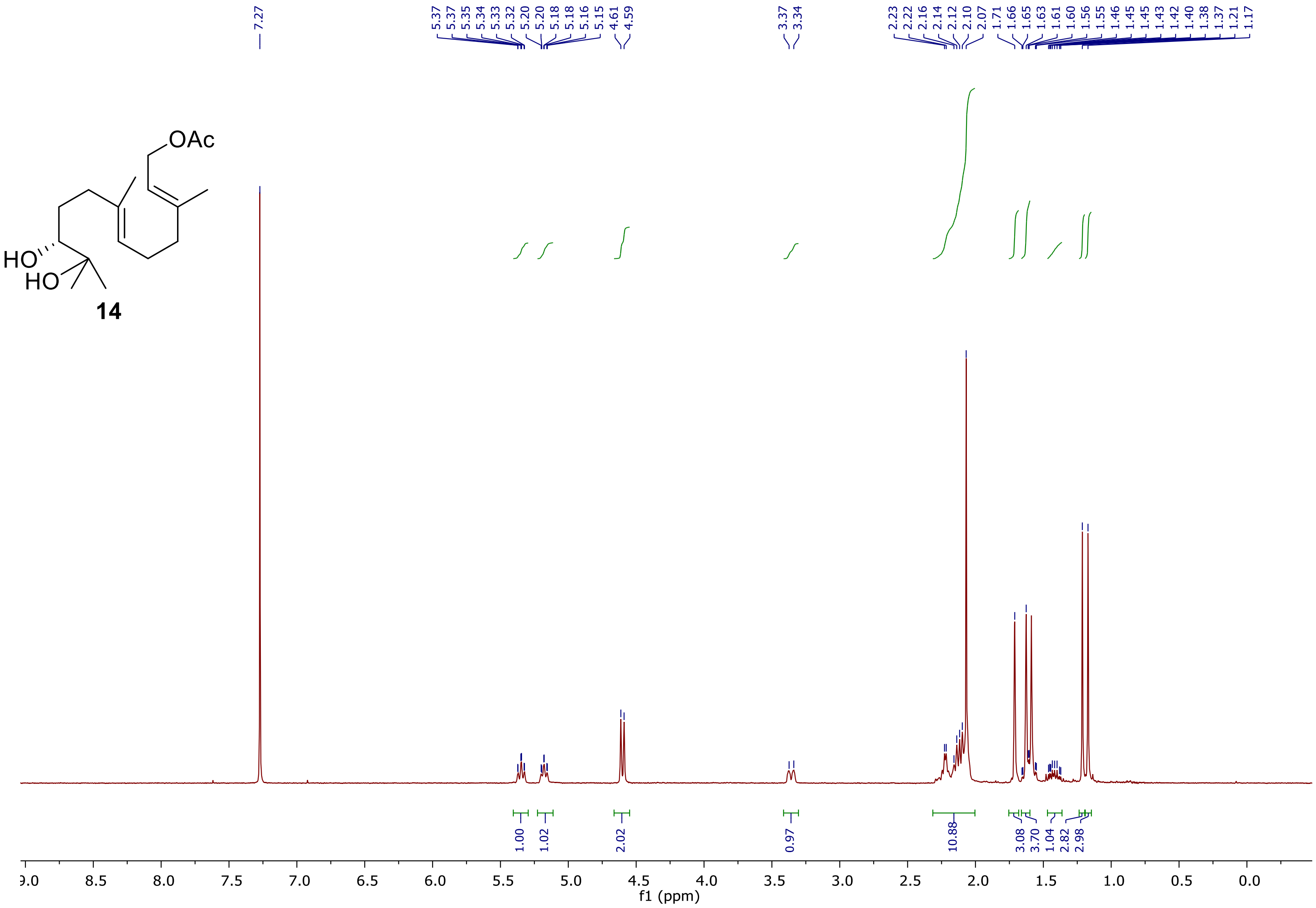

14

Figure NMR-S60. ^{1}H NMR spectrum of compound (R,1E,5E)-9,10-dihydroxy-2,6,10-trimethylundeca-1,5-dien-1-yl acetate (**14**).

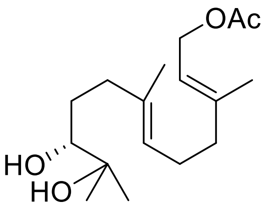

14

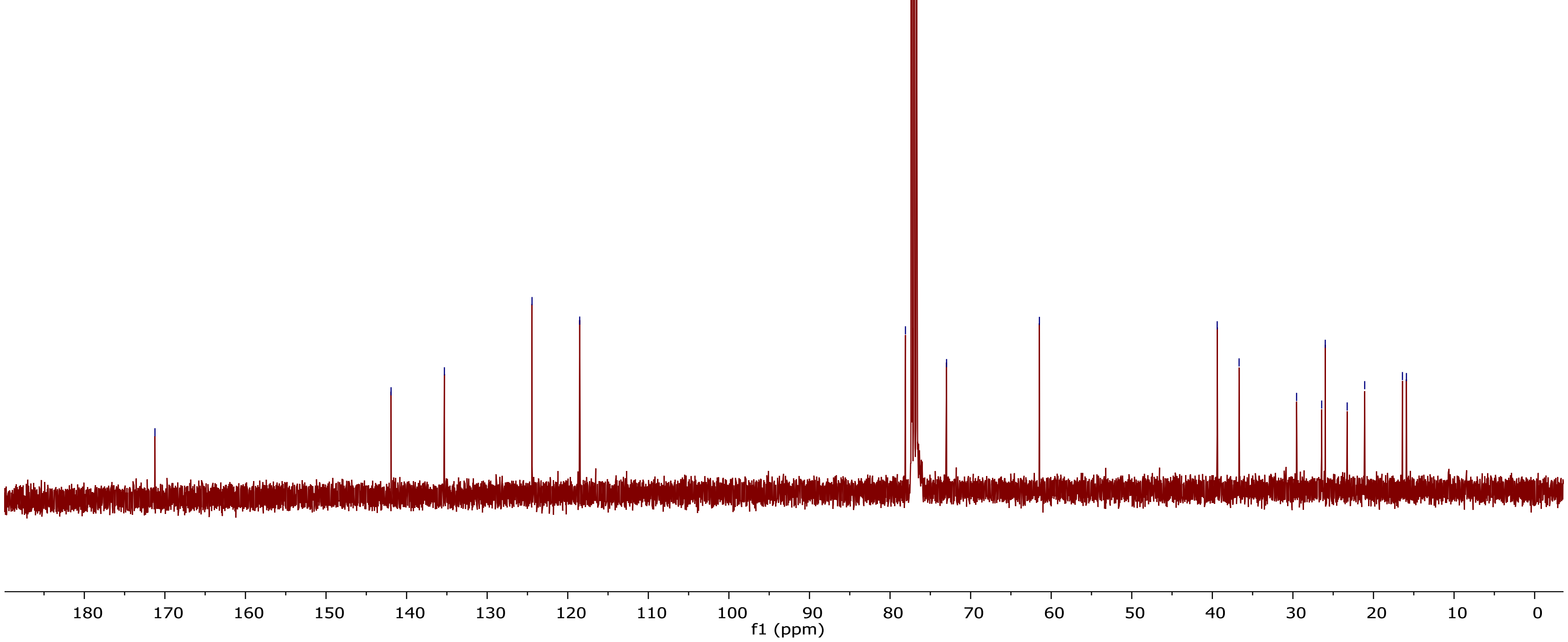

Figure NMR-S61. ^{13}C NMR spectrum of compound **14**.

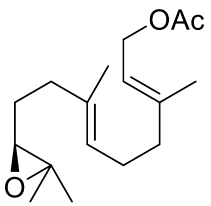

15

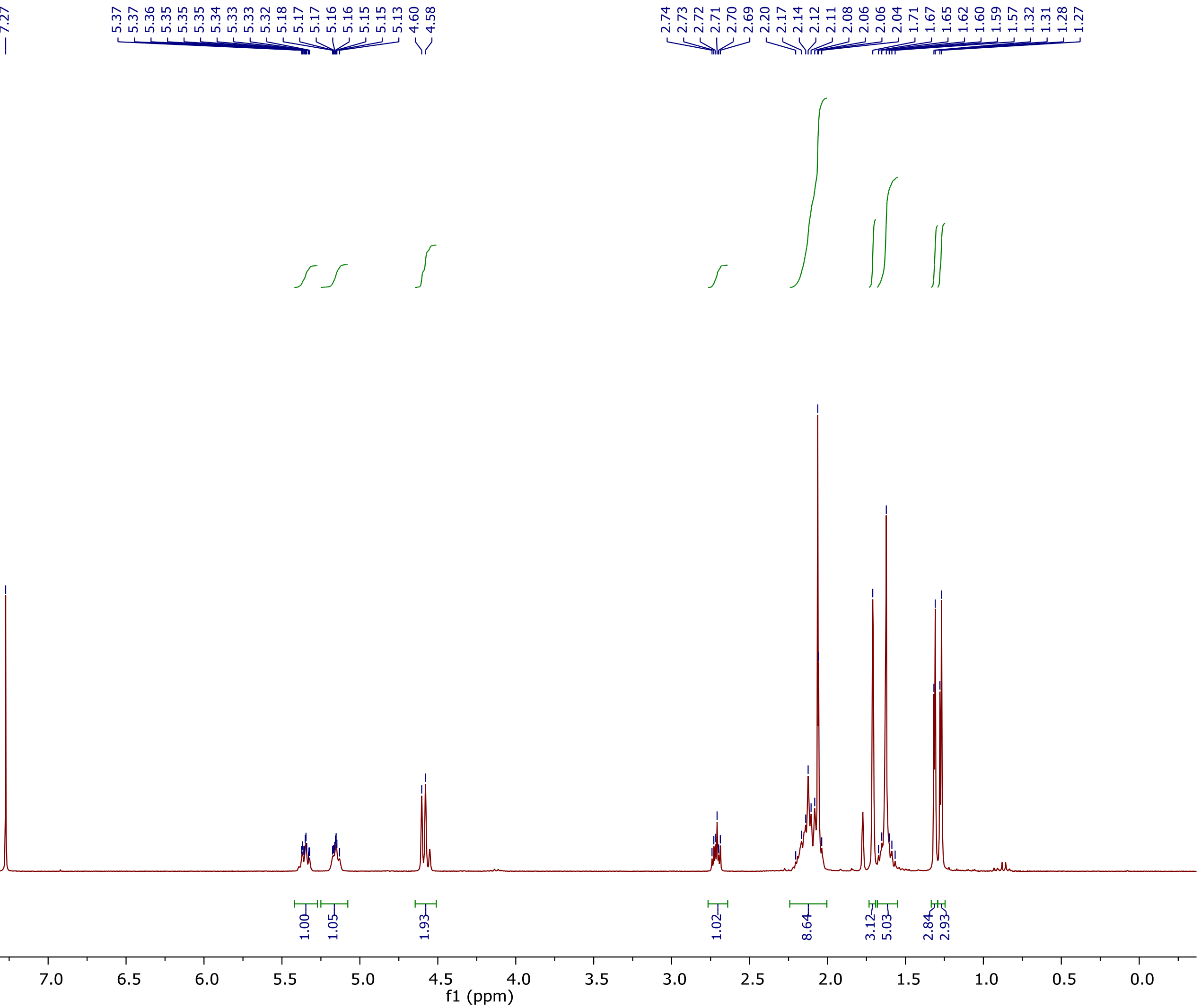

Figure NMR-S62. ^{1}H NMR spectrum of compound (2*E*,6*E*)-9-((*S*)-3,3-dimethyloxiran-2-yl)-3,7-dimethylnona-2,6-dien-1-yl acetate (**15**).

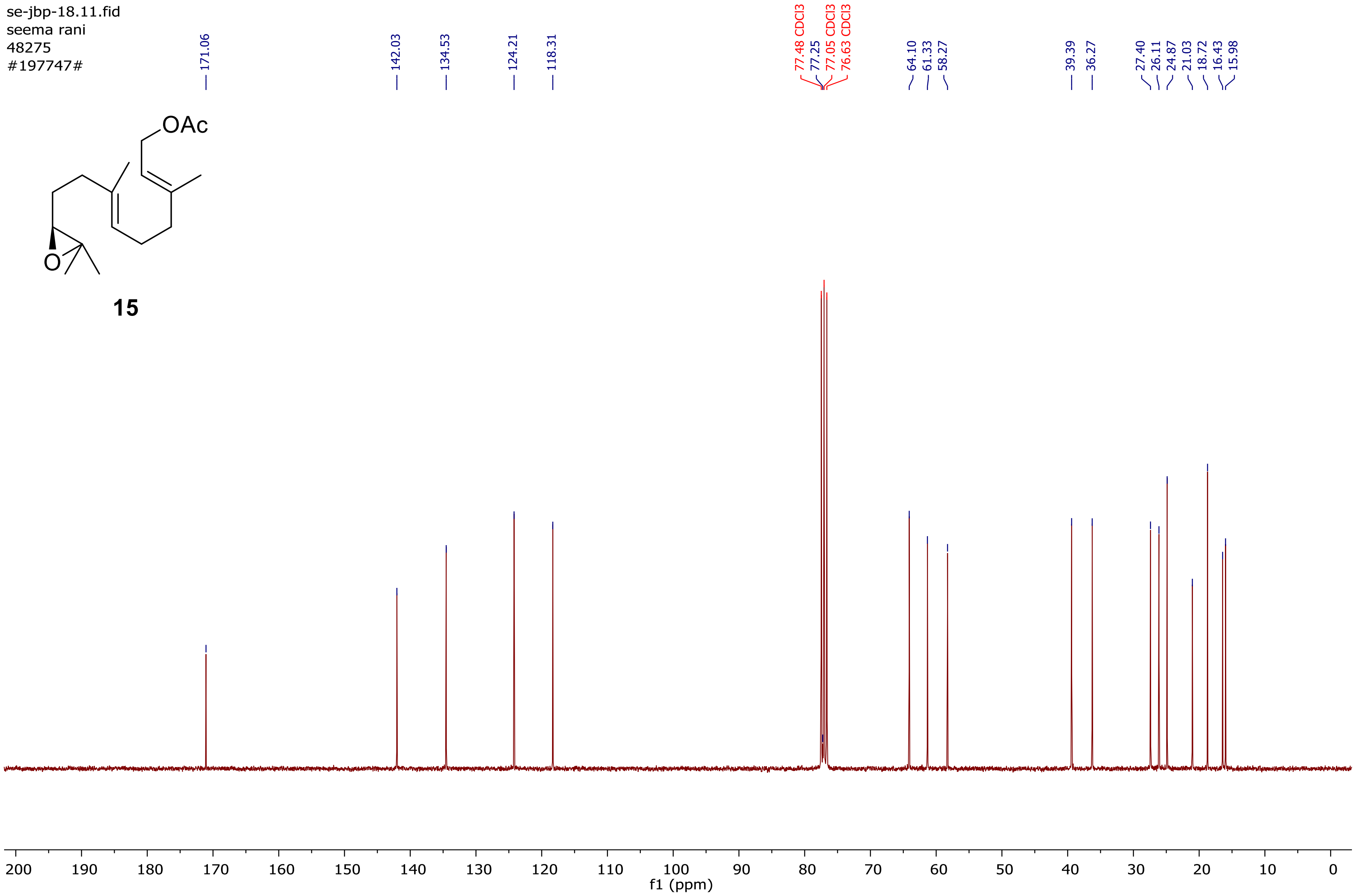

Figure NMR-S63. ^{13}C NMR spectrum of compound **15**.

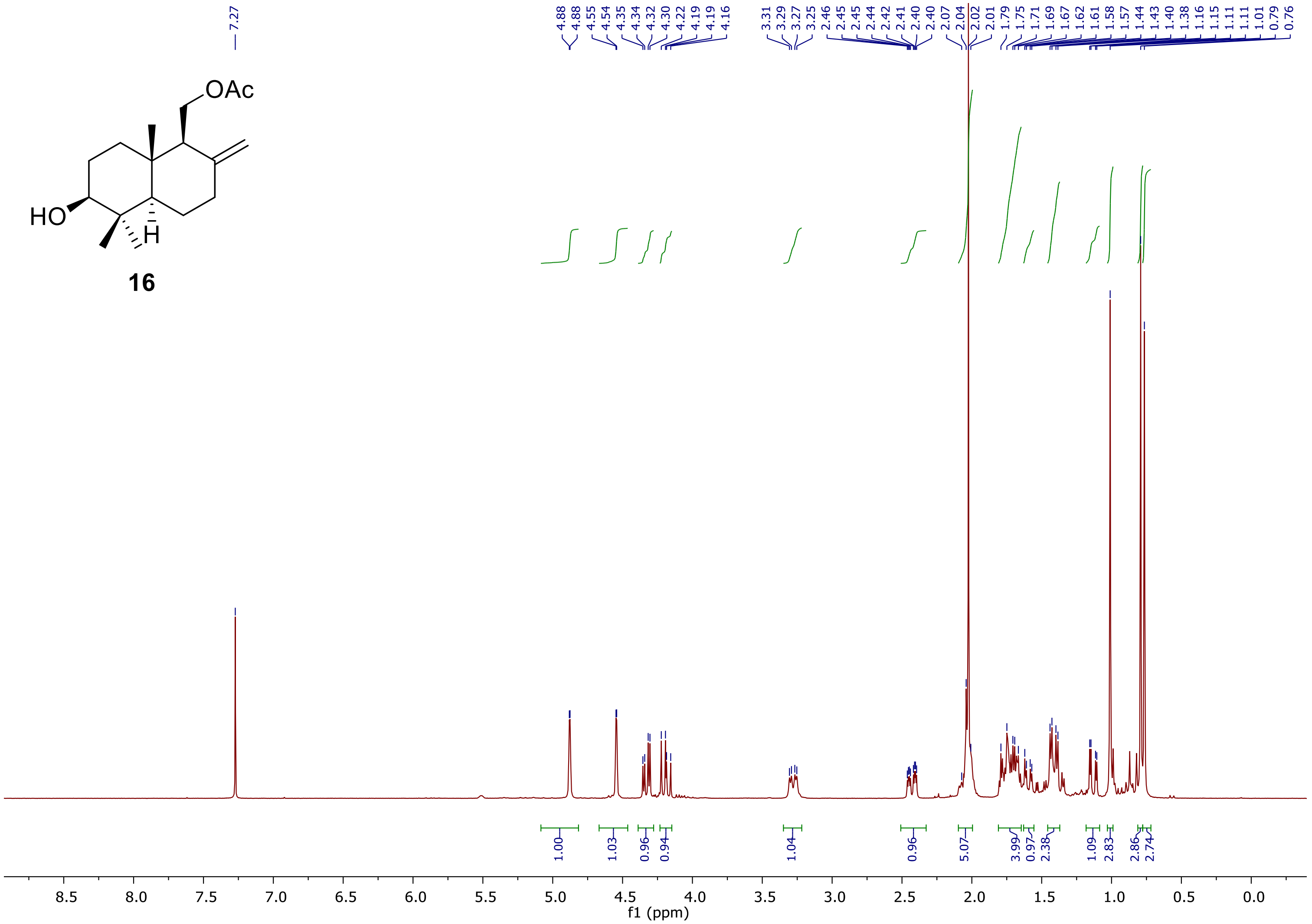

Figure NMR-S64. ^{1}H NMR spectrum of compound ((1*S*,4a*R*,6*S*,8a*S*)-6-hydroxy-5,5,8a-trimethyl-2-methylenedecahydronaphthalen-1-yl)methyl acetate (**16**).

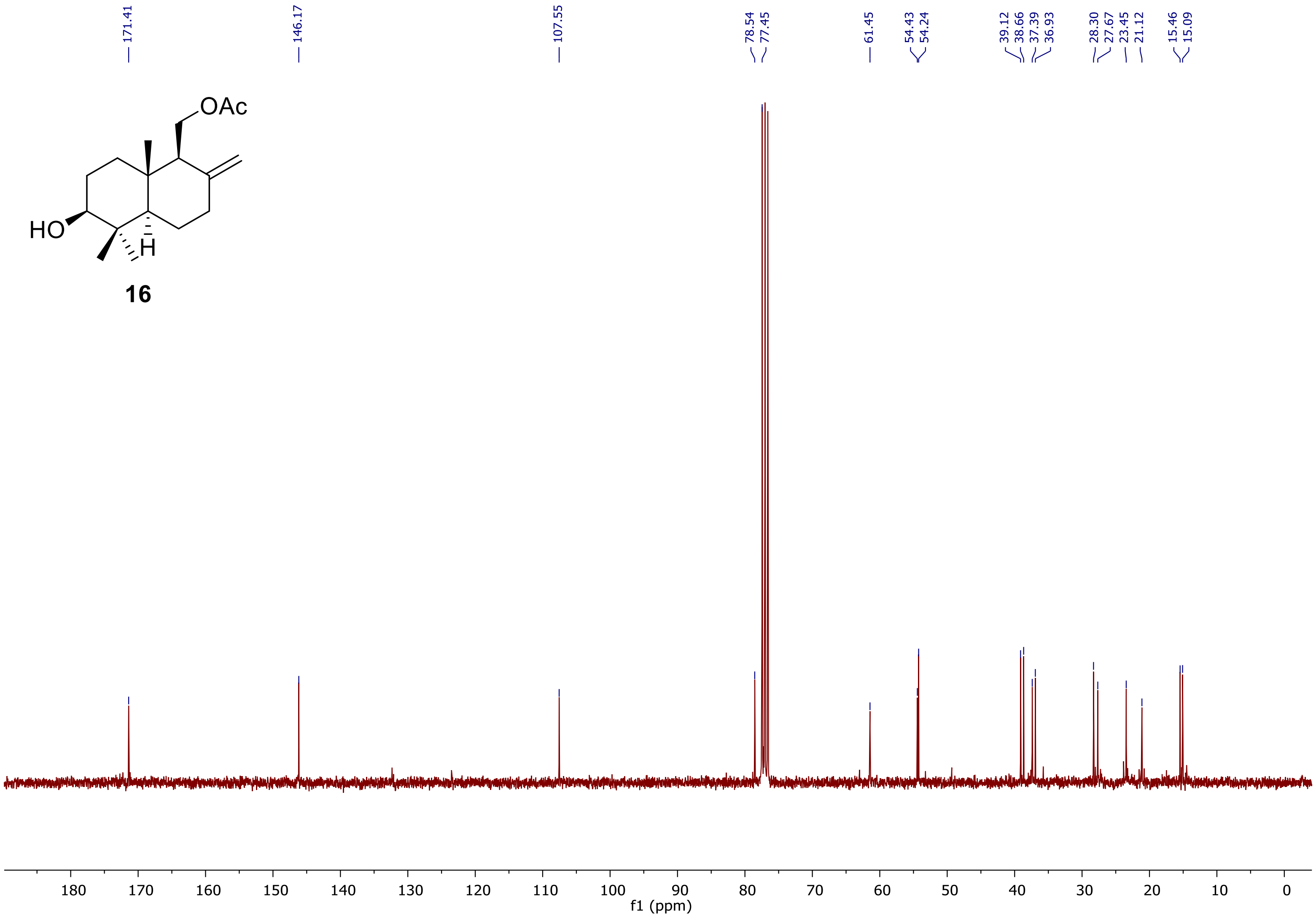

Figure NMR-S65. ^{13}C NMR spectrum of compound **16**.

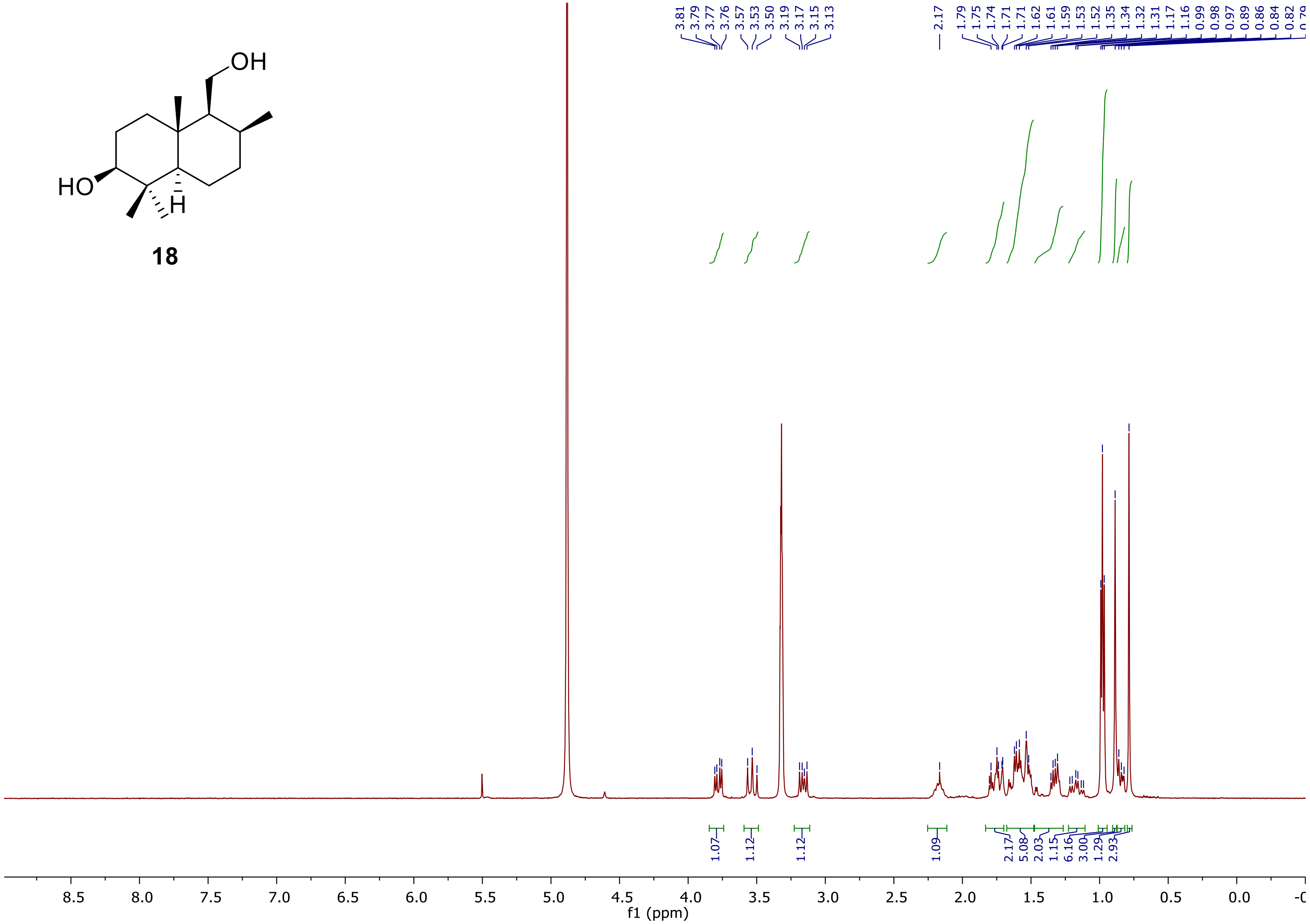

Figure NMR-S66. ^{1}H NMR spectrum of compound (2*S*,4a*S*,5*S*,6*S*,8a*R*)-5-(hydroxymethyl)-1,1,4a,6-tetramethyldecahydronaphthalen-2-ol (**18**).

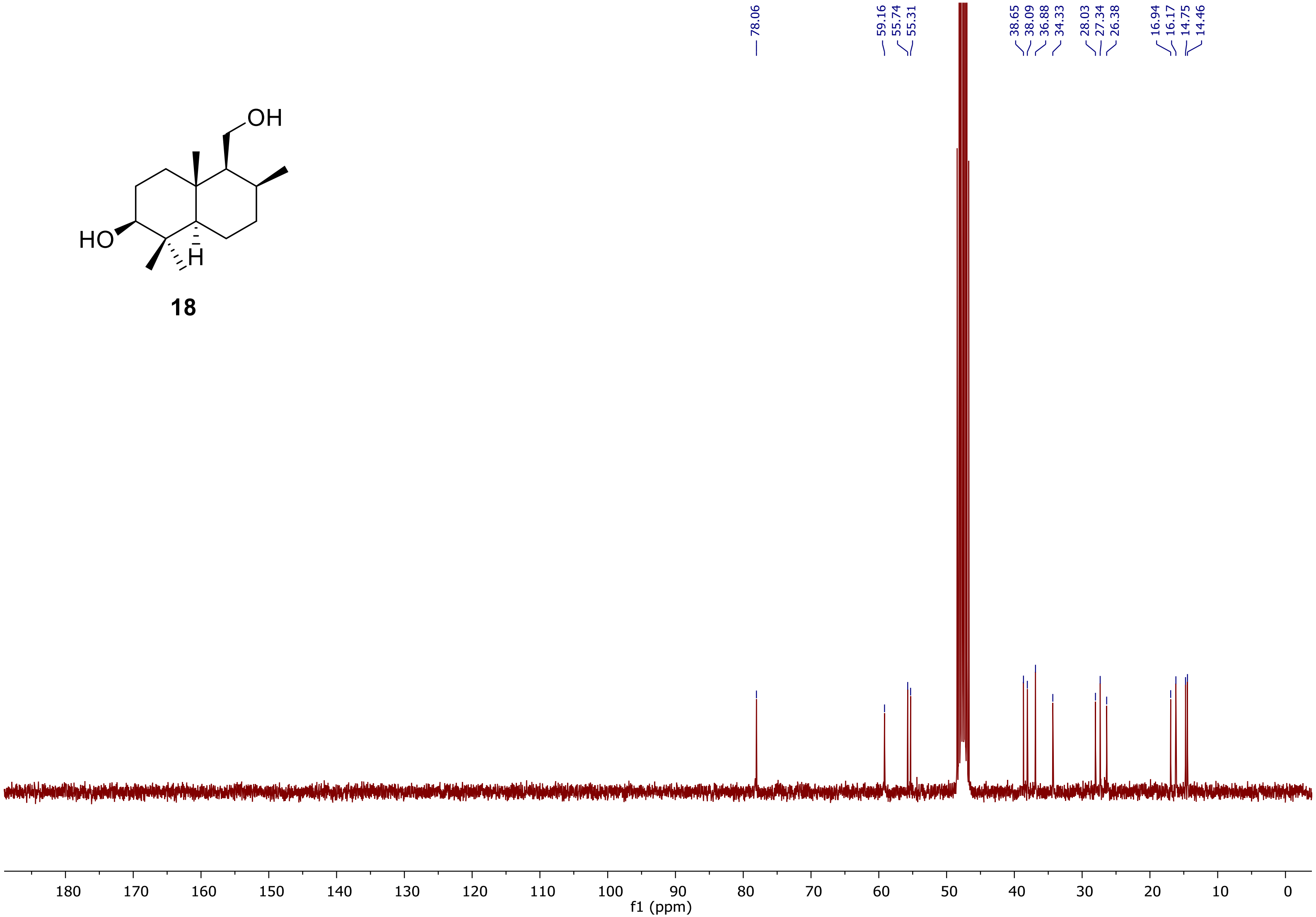

Figure NMR-S67. ^{13}C NMR spectrum of compound **18**.

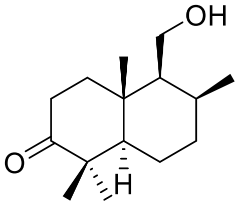

19

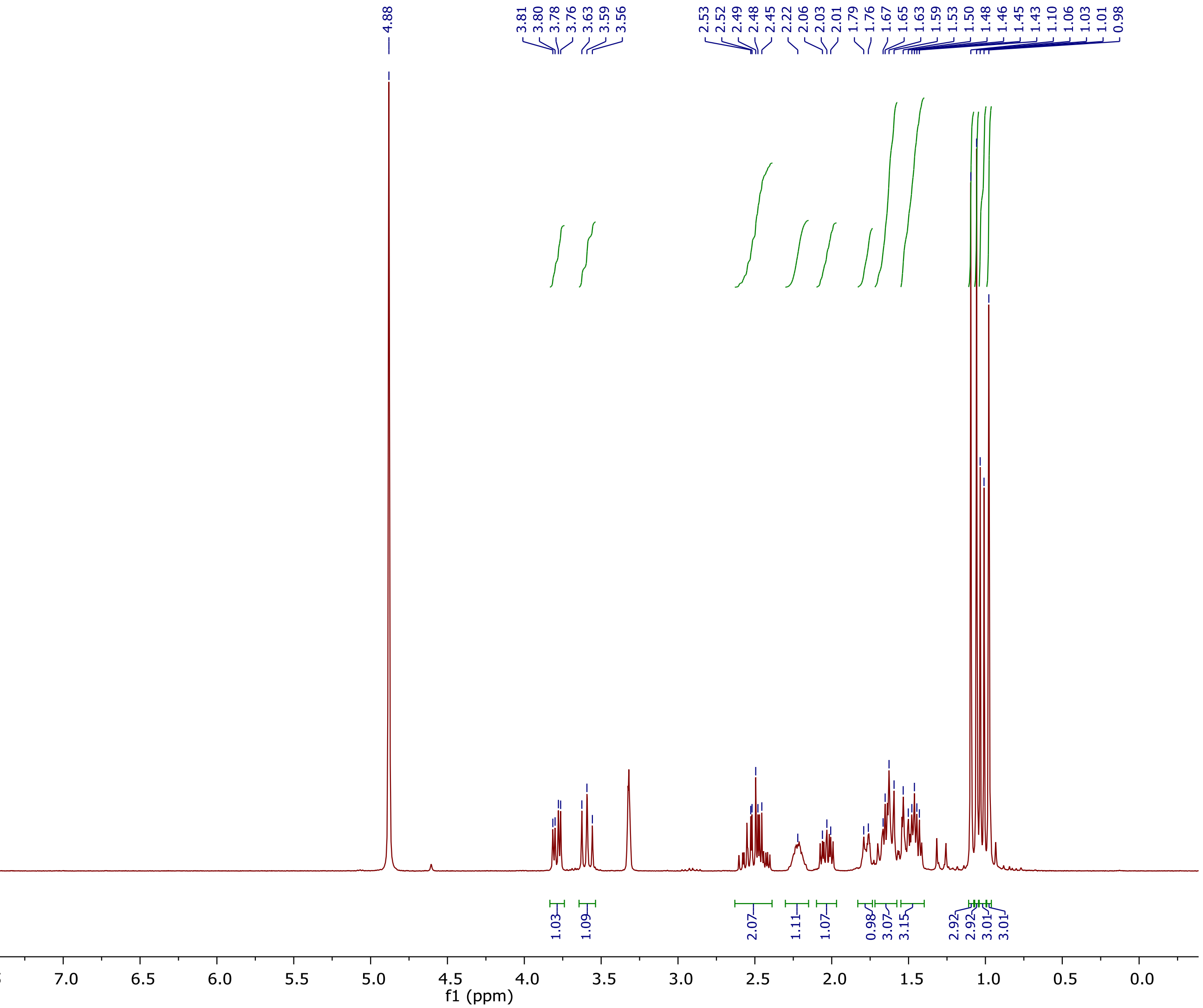

Figure NMR-S68. ^{1}H NMR spectrum of compound (4a*S*,5*S*,6*S*,8a*R*)-5-(hydroxymethyl)-1,1,4a,6-tetramethyloctahydronaphthalen-2(1H)-one (**19**).

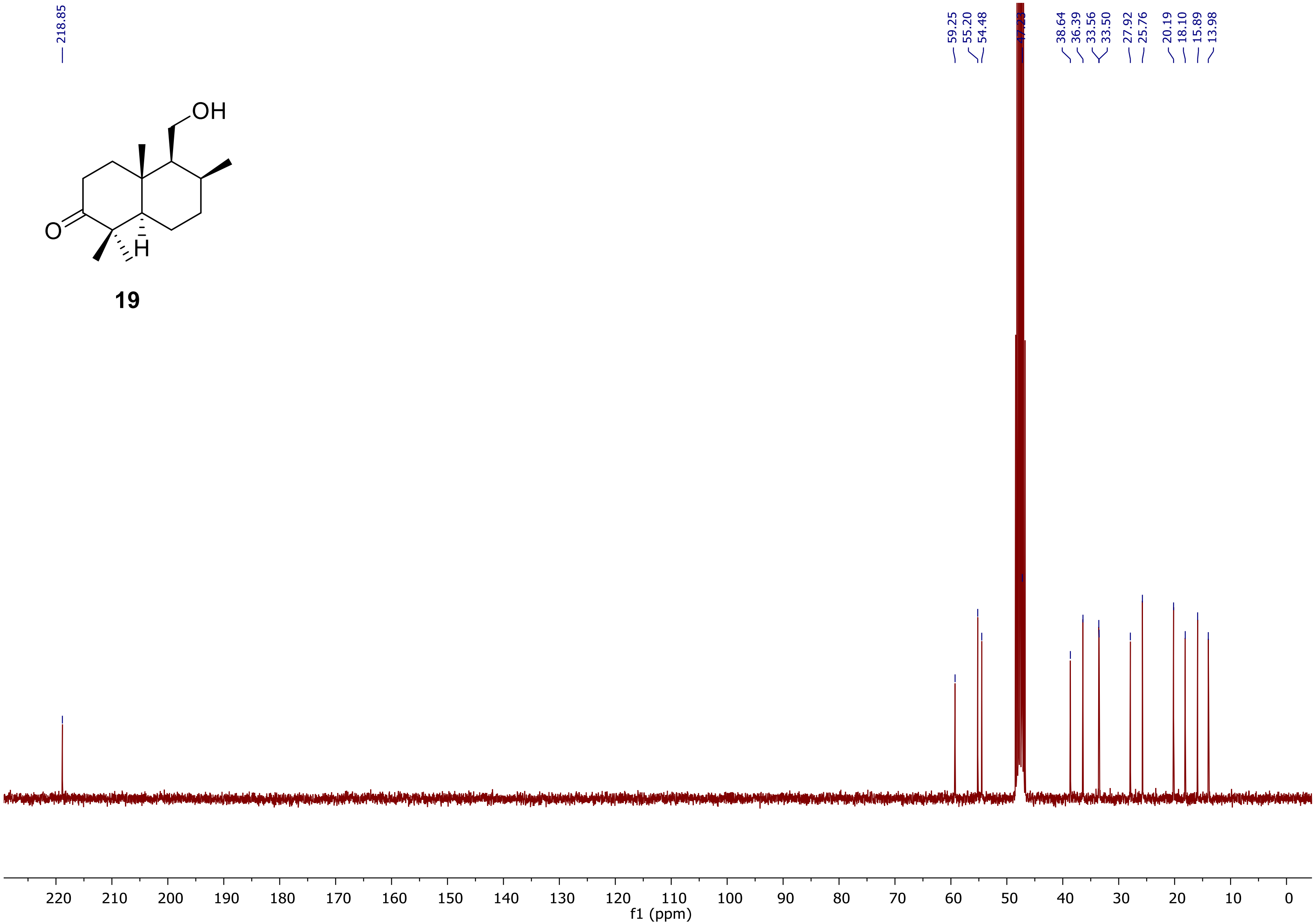

Figure NMR-S69. ^{13}C NMR spectrum of compound **19**.

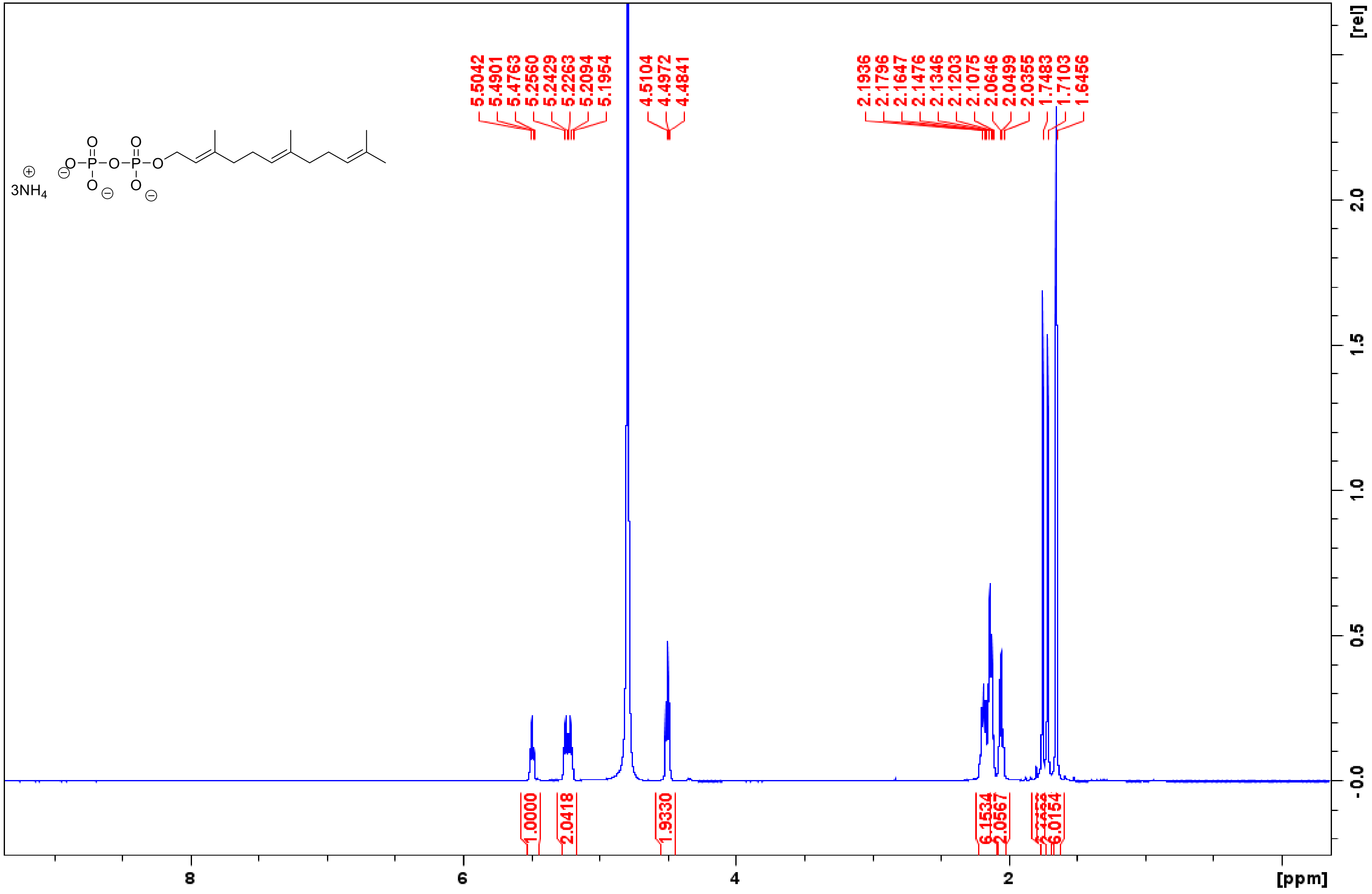

Figure NMR-S70. ^{1}H NMR spectrum of **20** (D_2O, 500 MHz).

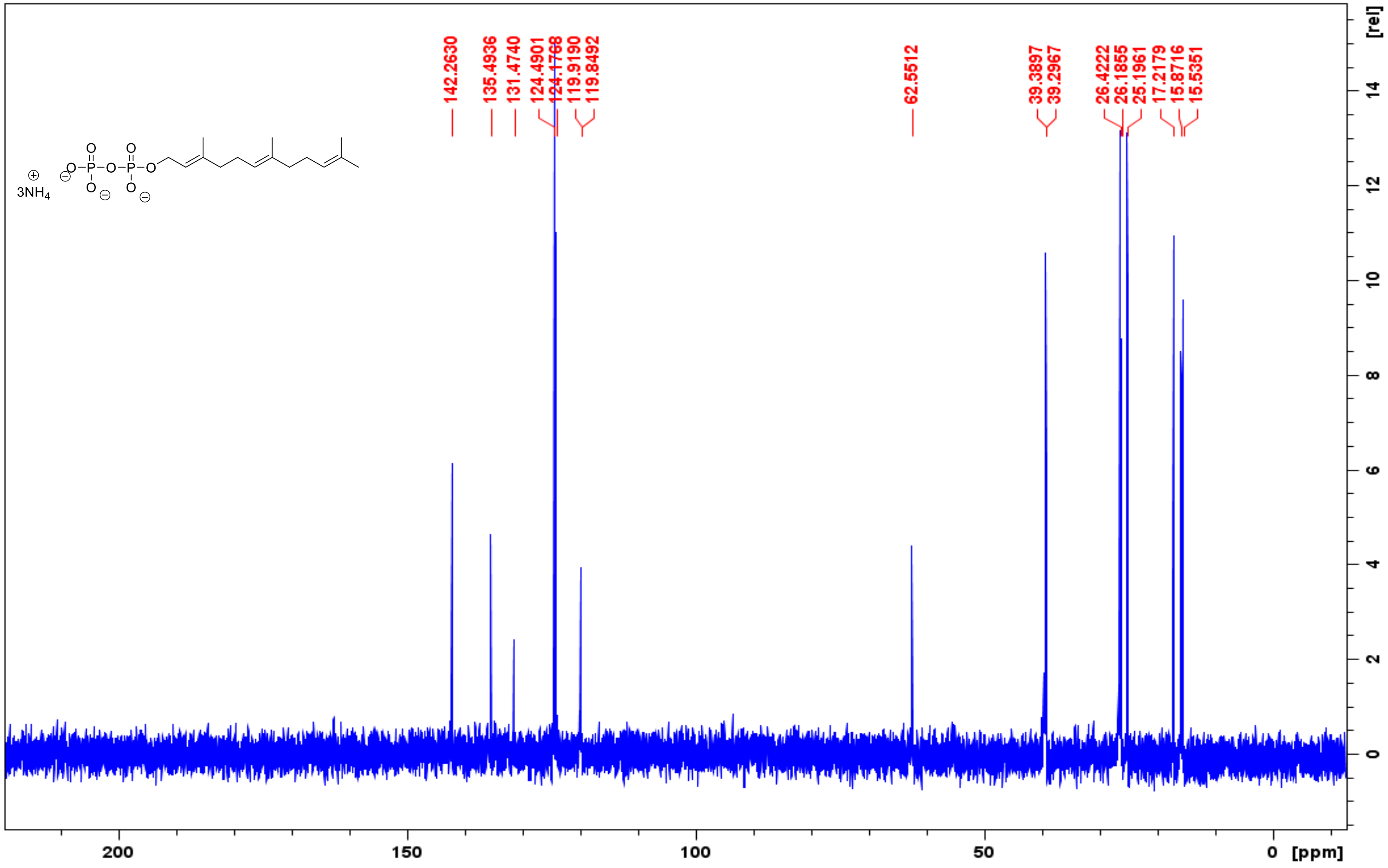

Figure NMR-S71. ^{13}C NMR spectrum of **20** (D_2O, 125 MHz).

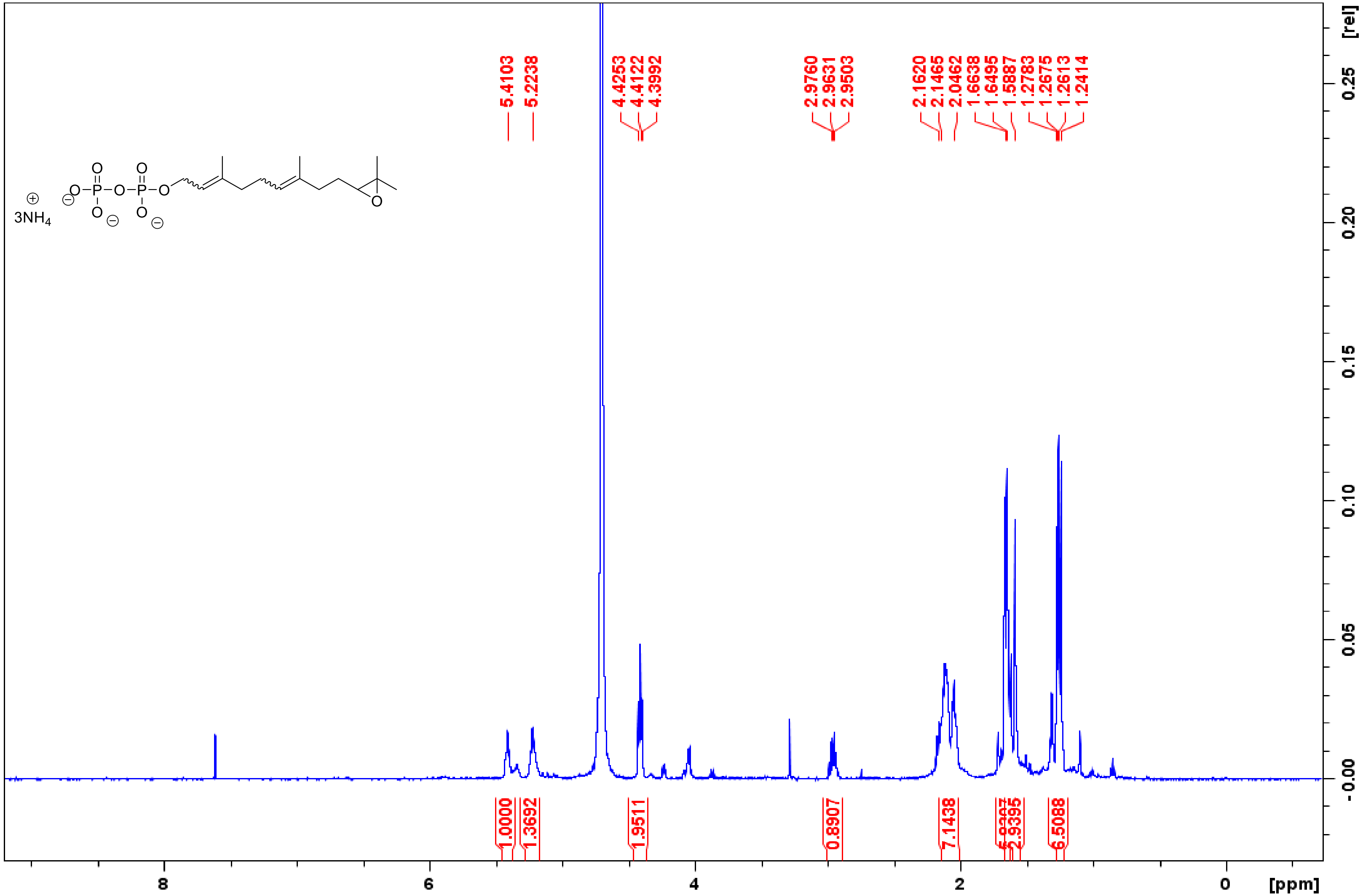

Figure NMR-S72. ^{1}H NMR spectrum of **21** (D_2O, 500 MHz).

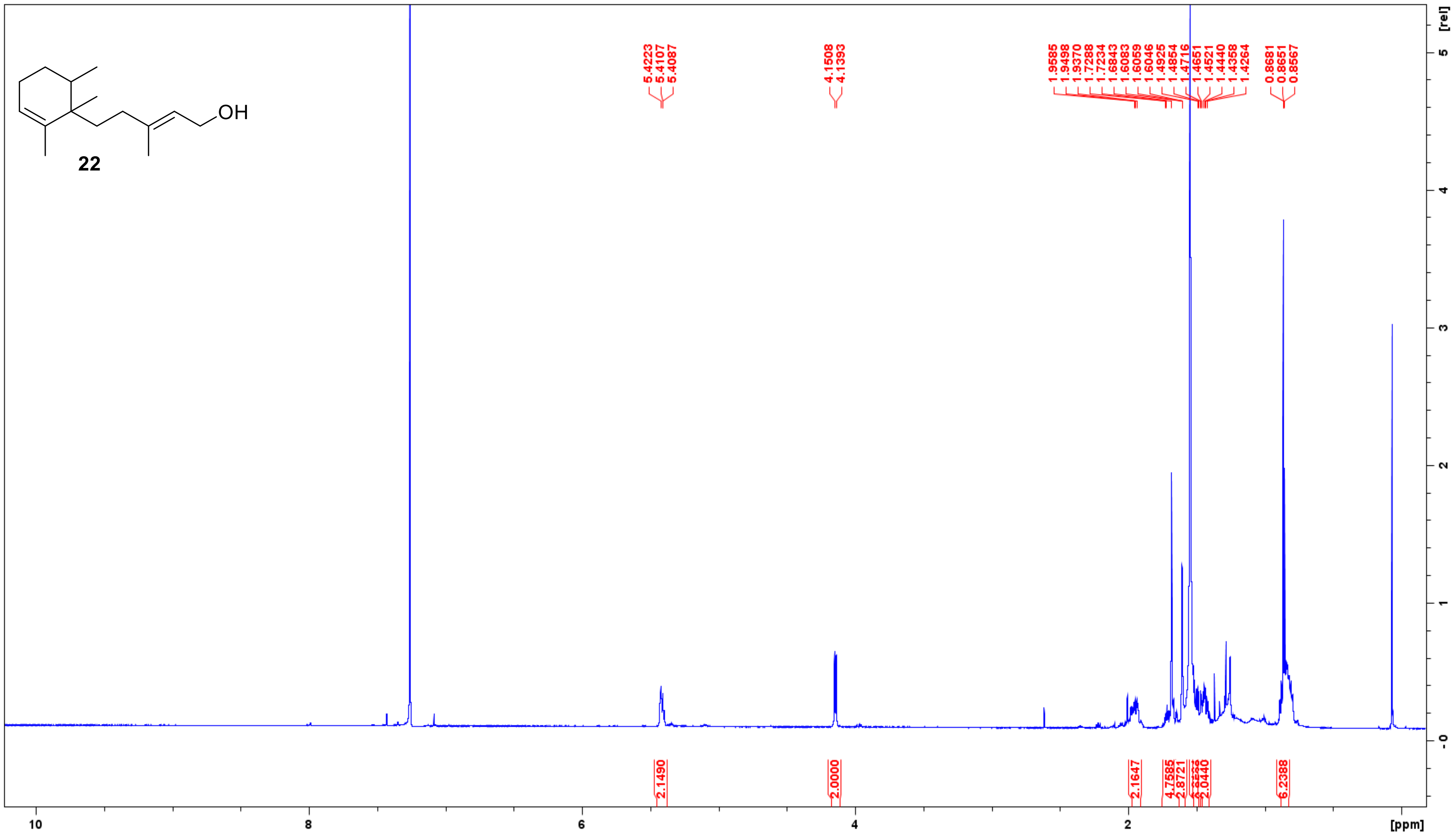

Figure NMR-S73. ^{1}H NMR spectrum of compound **22** ($CDCl_3$, 300 K, 600 MHz).

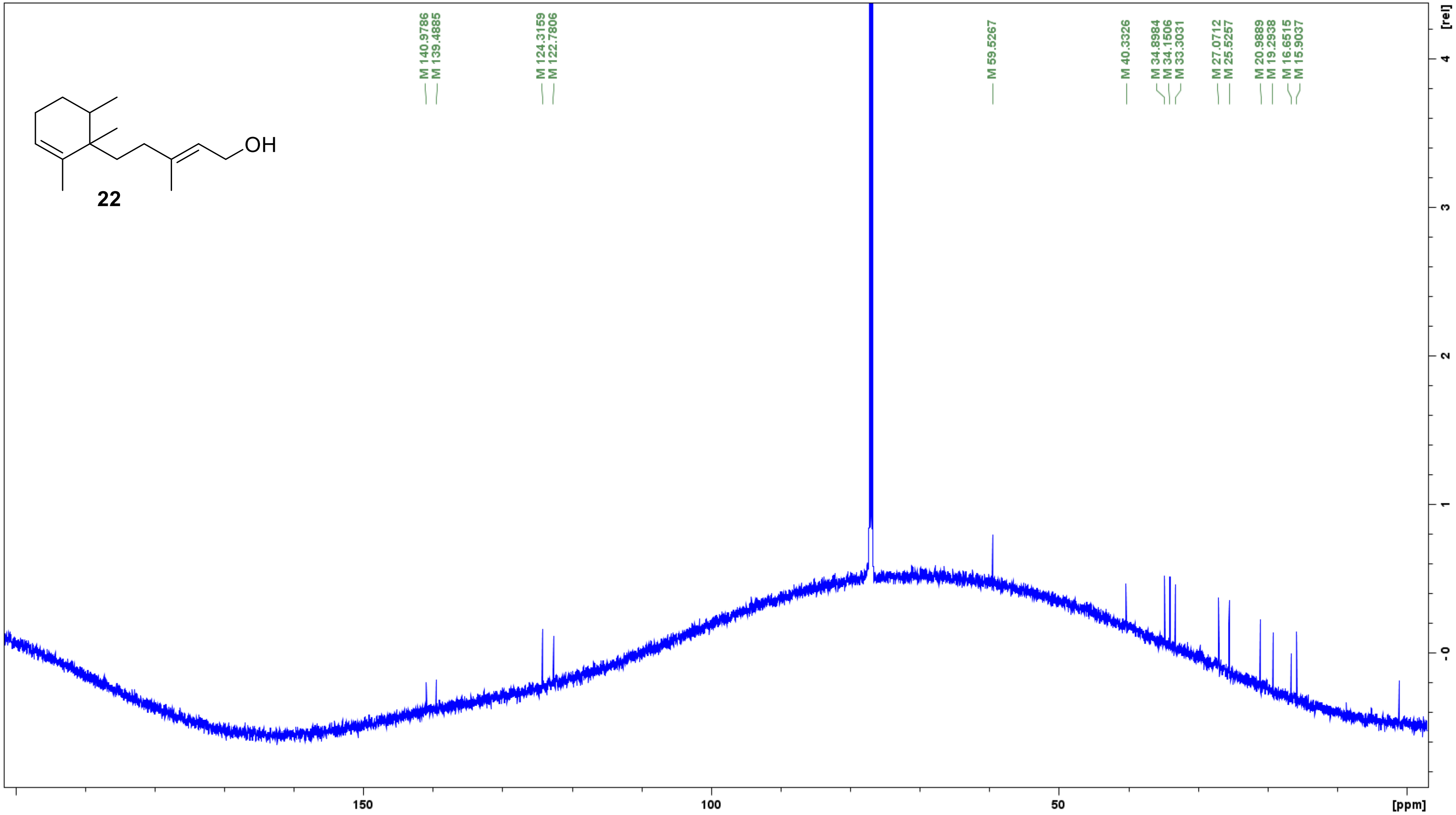

Figure NMR-S74. ^{13}C NMR spectrum of compound **22**.

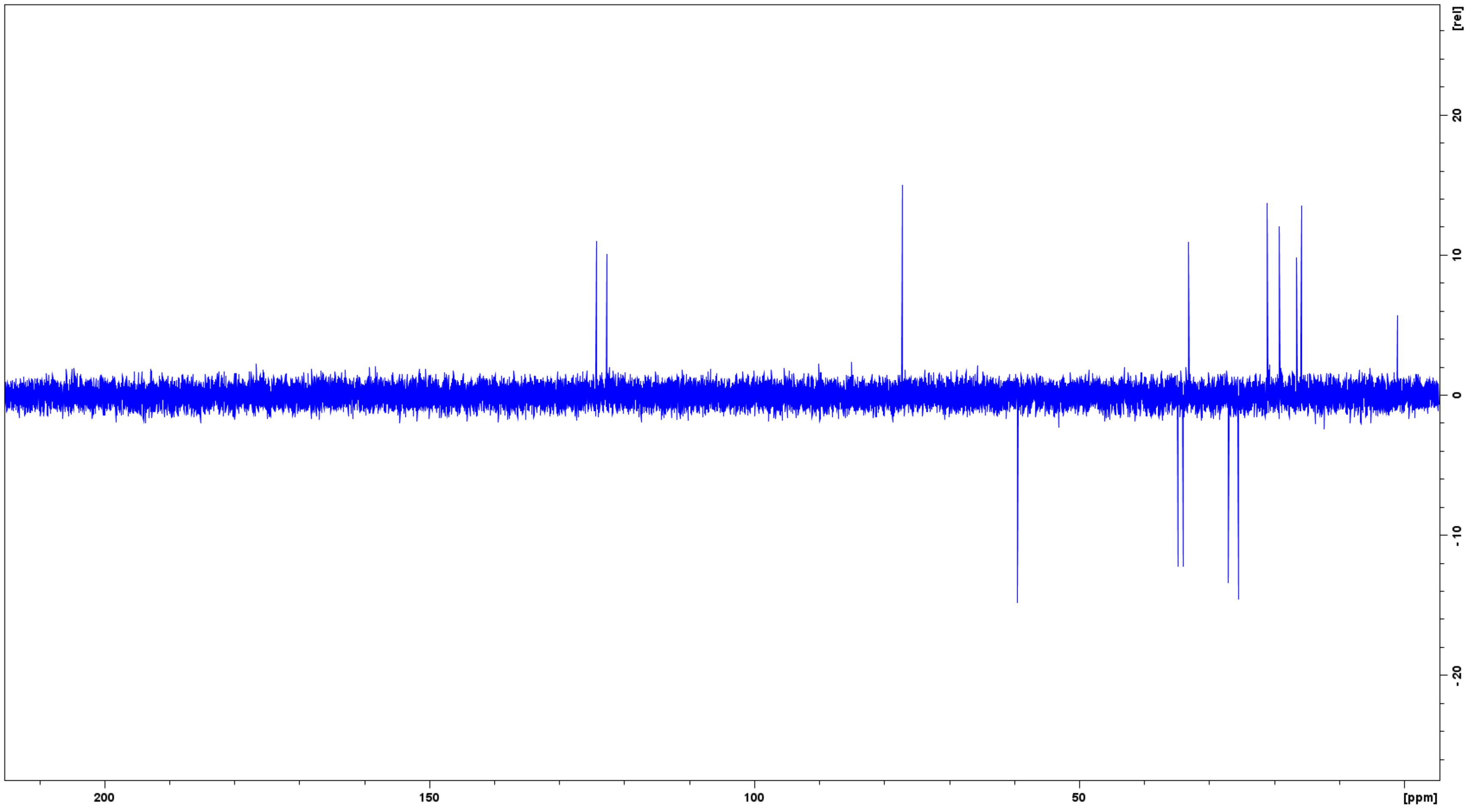

Figure NMR-S75. ^{13}C NMR DEPT spectrum of compound **22** ($CDCl_3$, 300 K, 150 MHz).

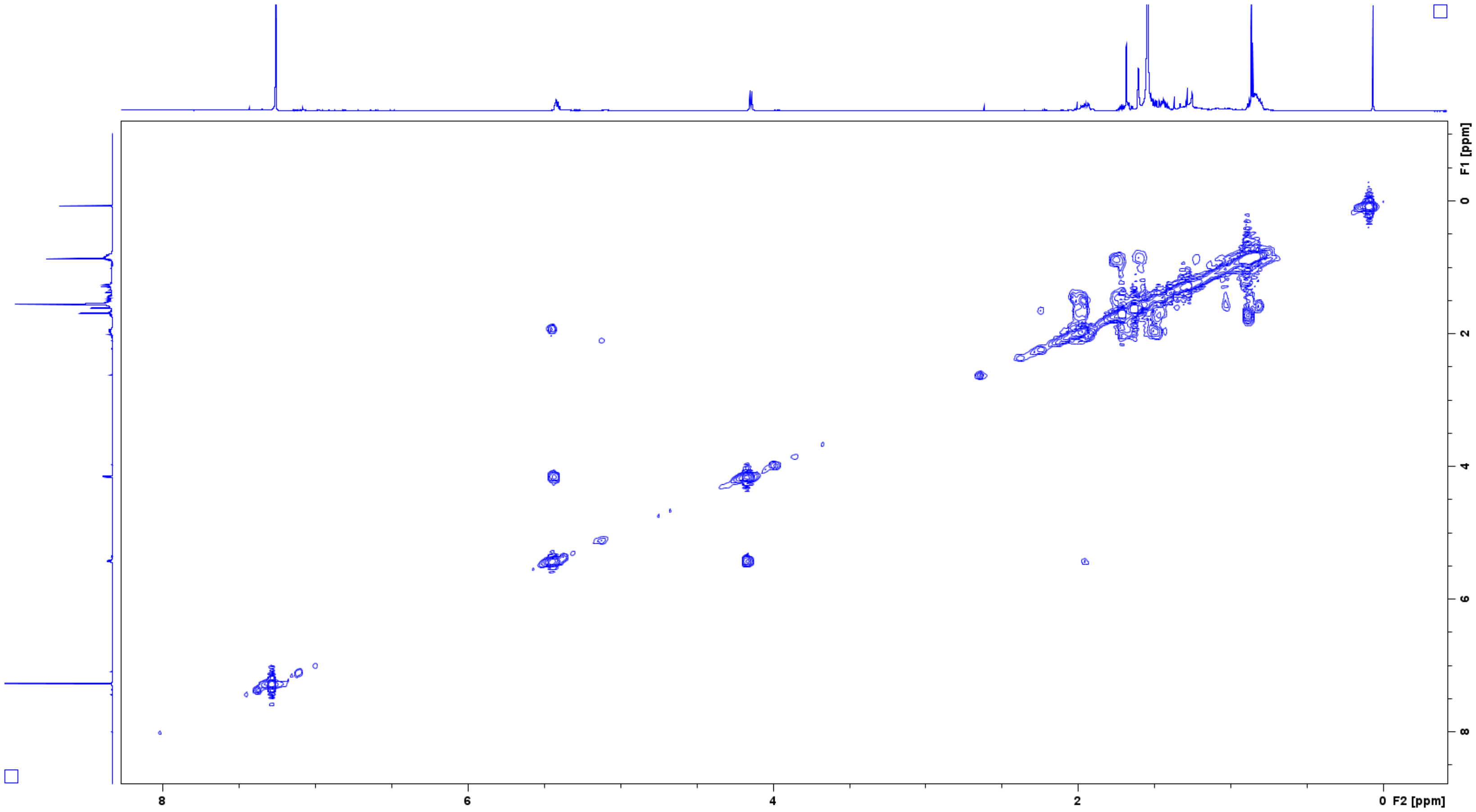

Figure NMR-S76. COSY spectrum of compound **22** ($CDCl_3$, 300 K, 600 MHz).

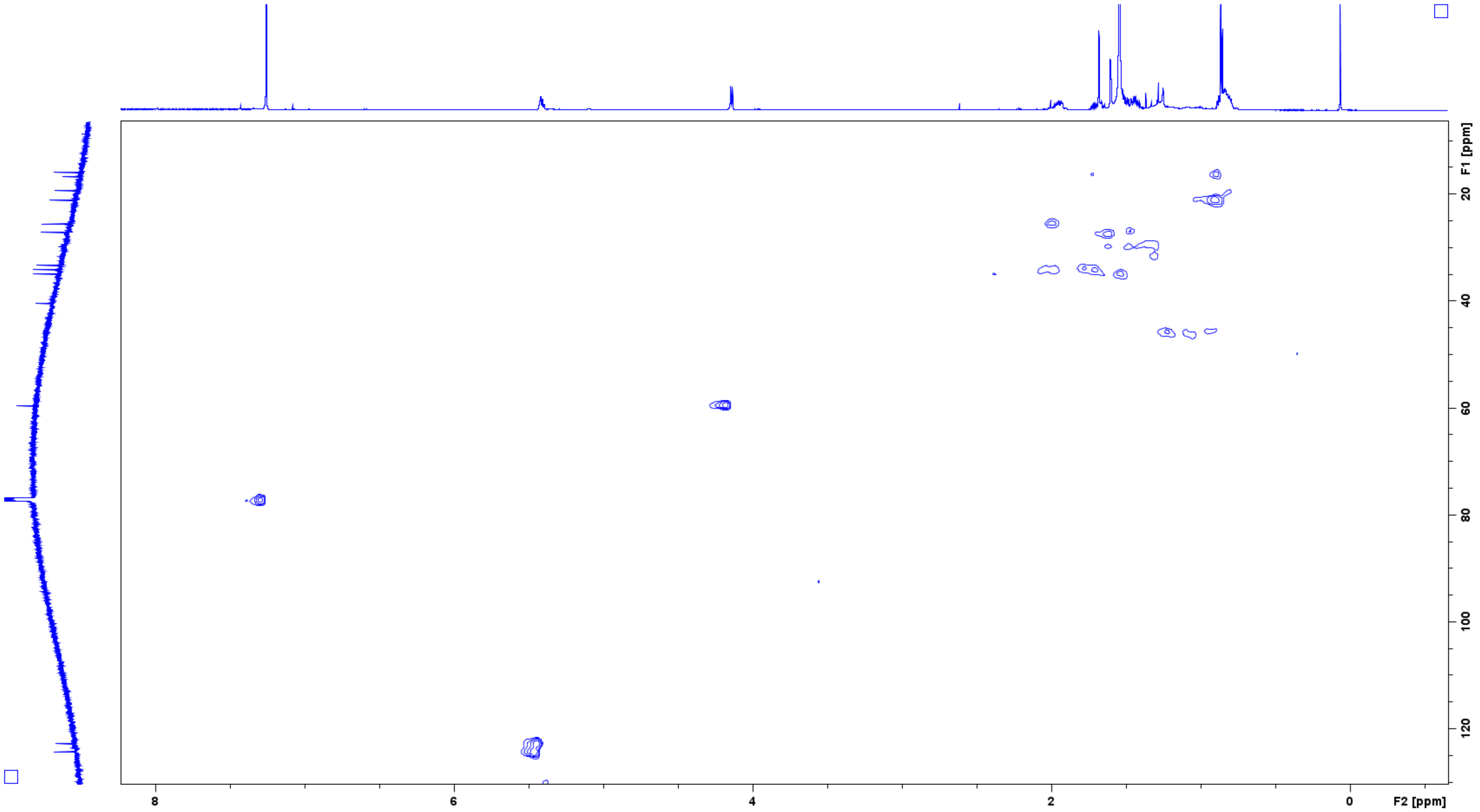

Figure NMR-S77. HSQC spectrum of compound **22** ($CDCl_3$, 300 K, 600 MHz).

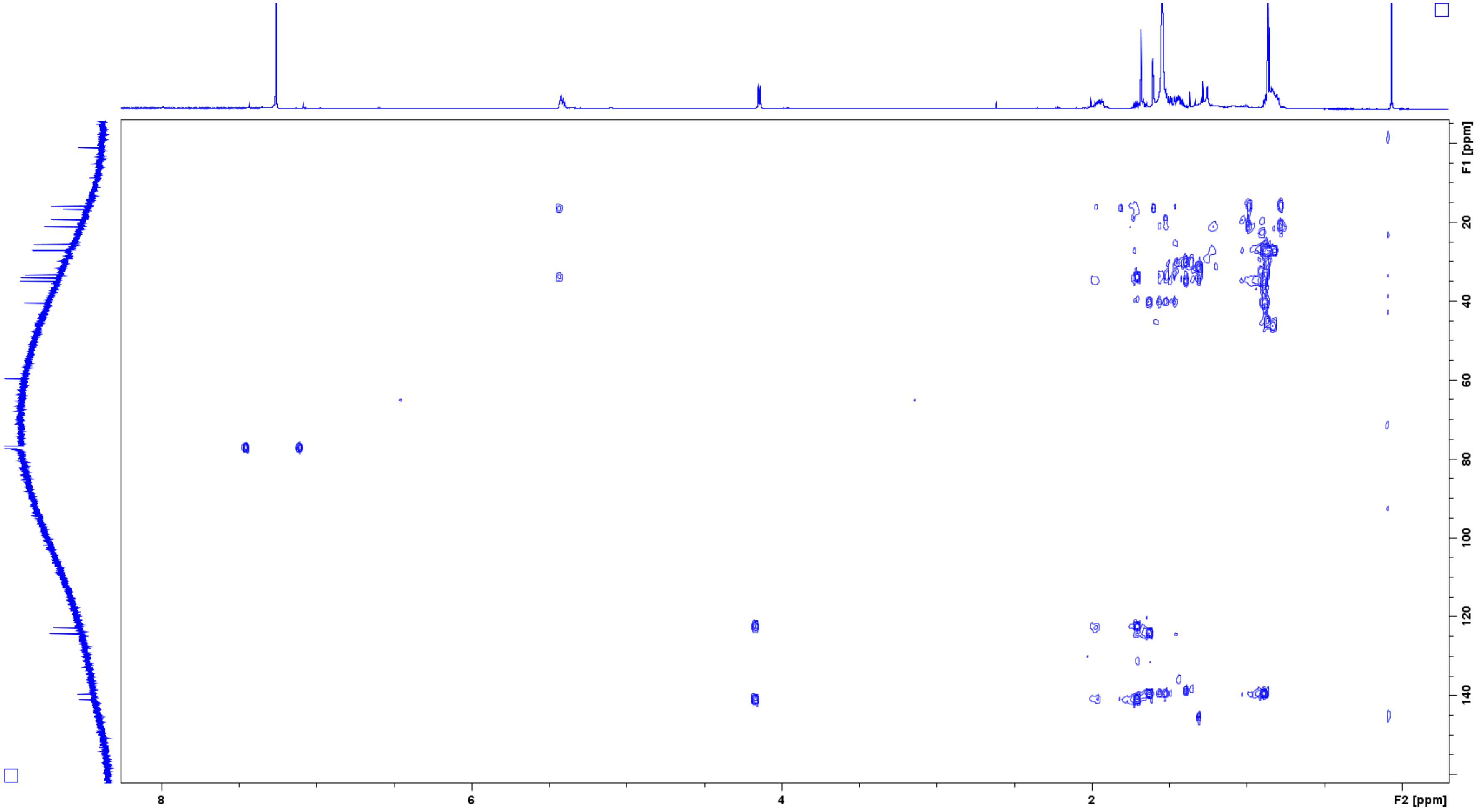

Figure NMR-S78. HMBC spectrum of compound **22** ($CDCl_3$, 300 K, 600 MHz).

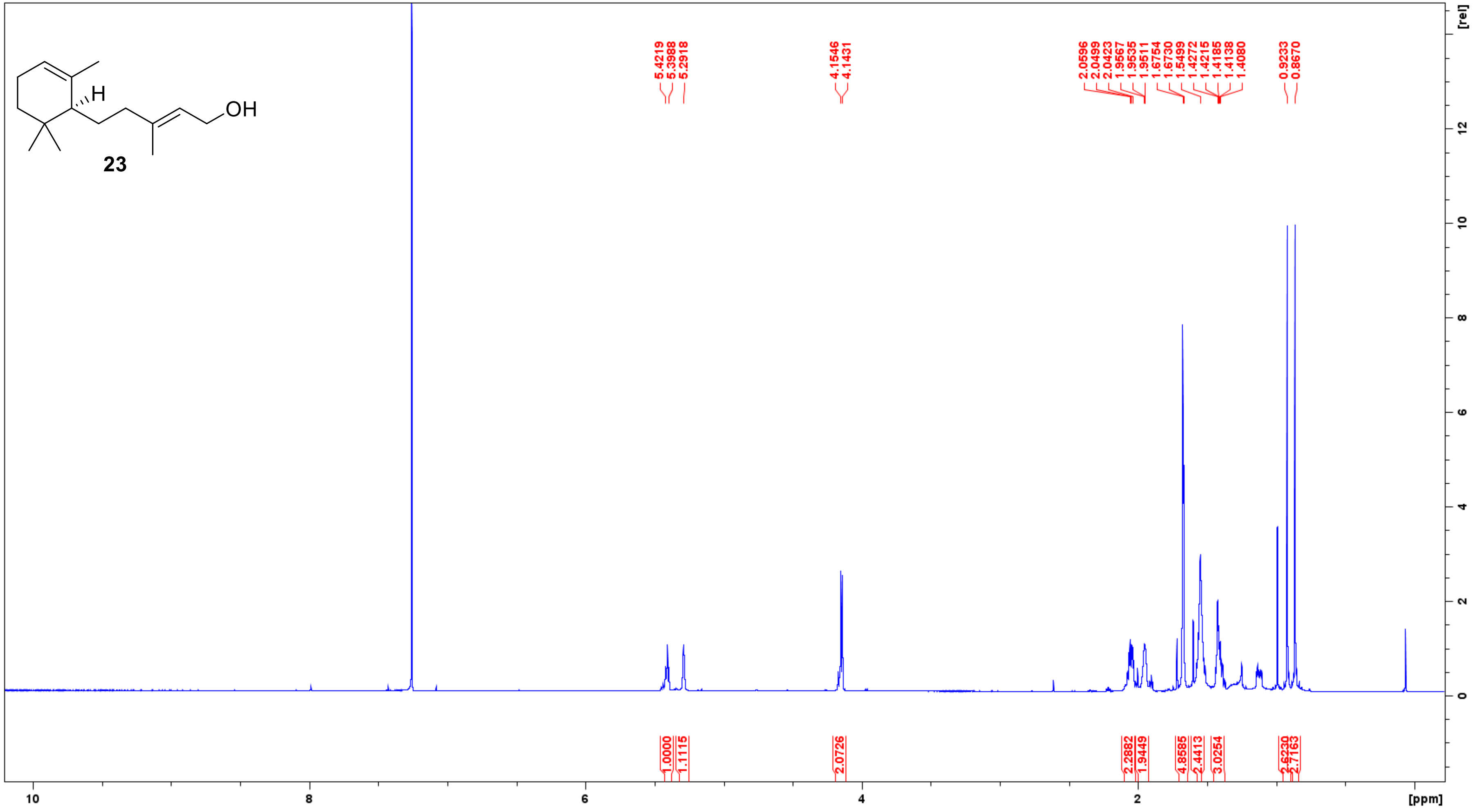

Figure NMR-S79. ^{1}H NMR spectrum of compound **23** ($CDCl_3$, 300 K, 600 MHz).

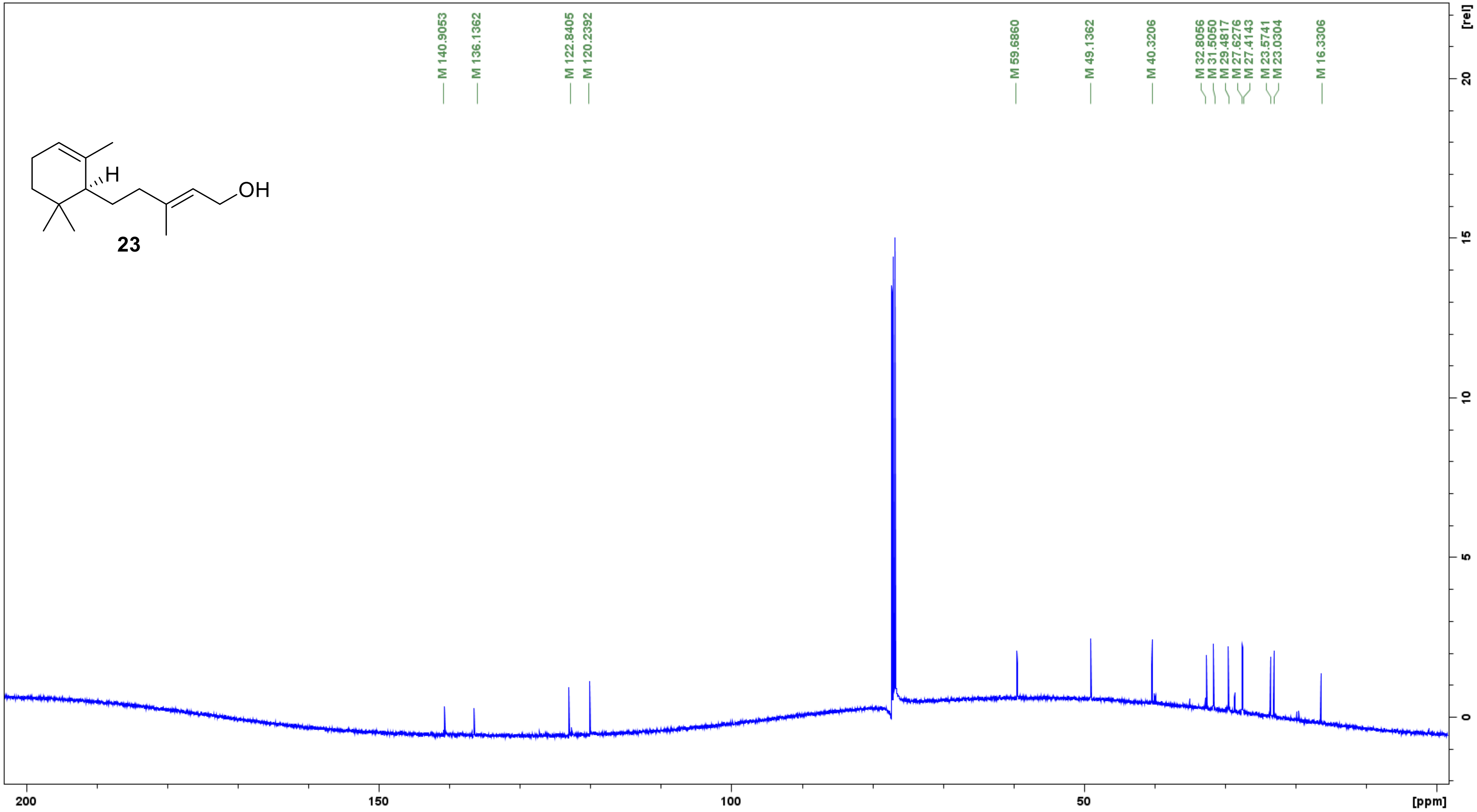

Figure NMR-S80. ^{13}C NMR spectrum of compound **23**.

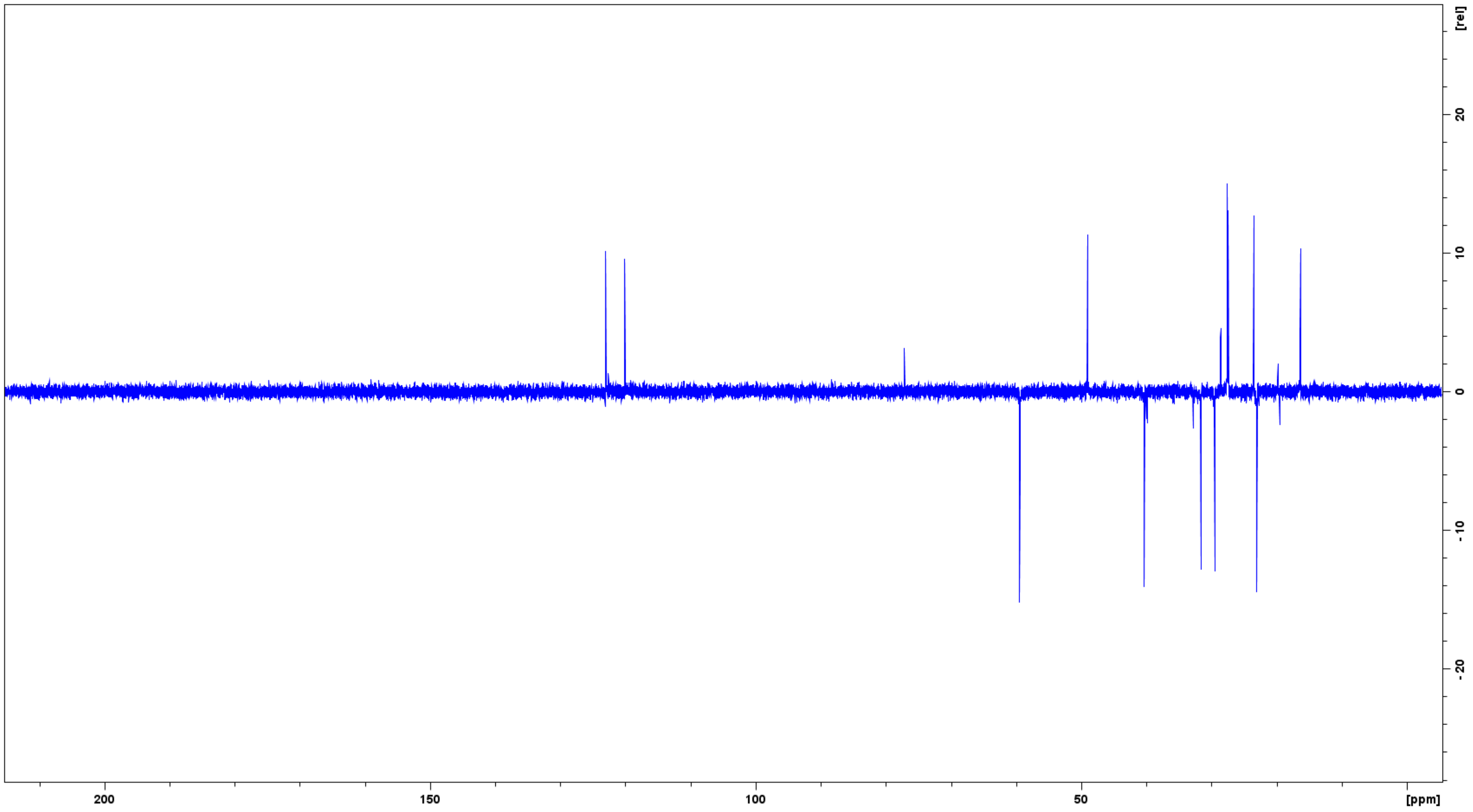

Figure NMR-S81. ^{13}C NMR DEPT spectrum of compound **23** ($CDCl_3$, 300 K, 150 MHz).

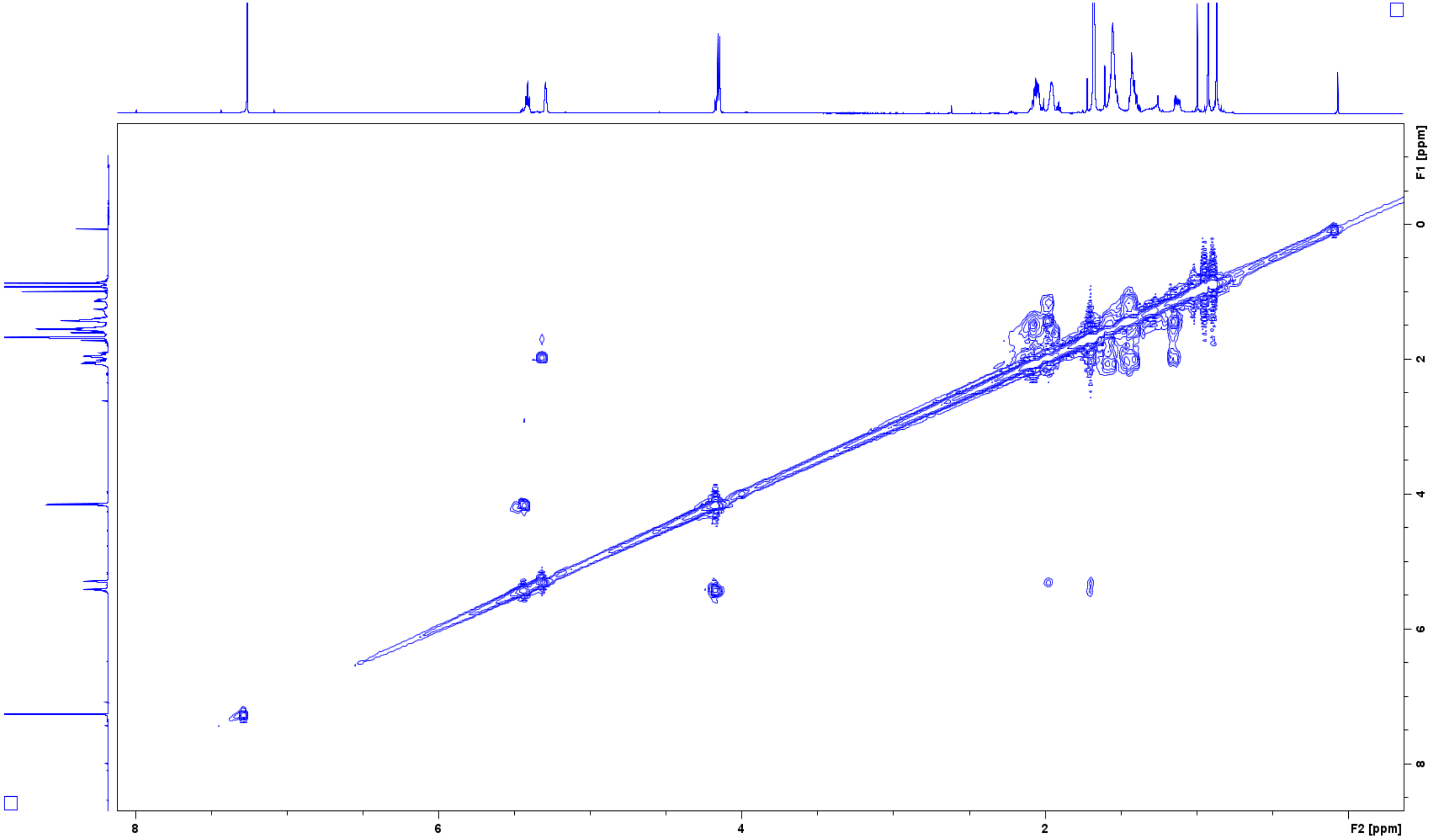

Figure NMR-S82. COSY spectrum of compound **23** ($CDCl_3$, 300 K, 600 MHz).

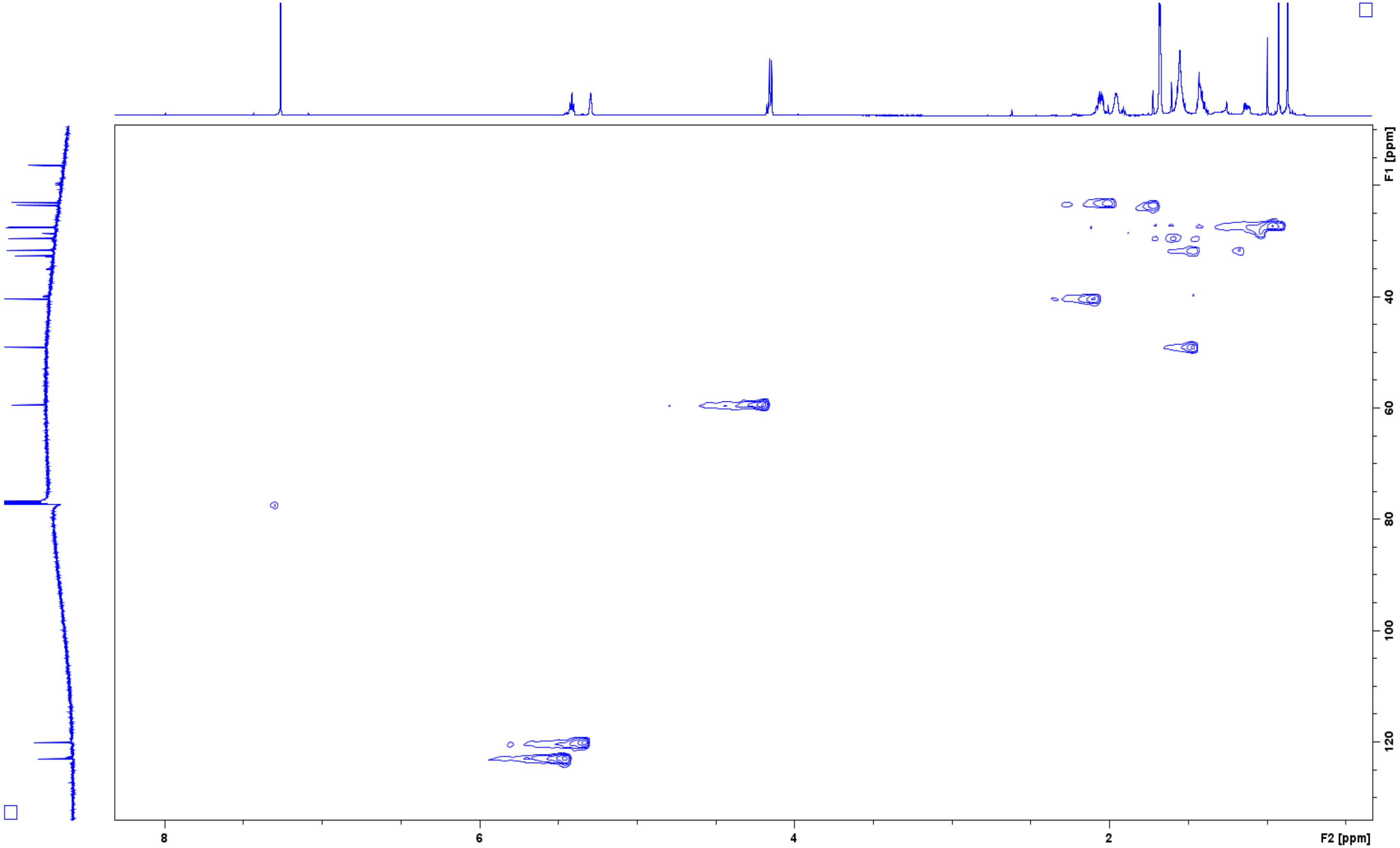

Figure NMR-S83. HSQC spectrum of compound **23** ($CDCl_3$, 300 K, 600 MHz).

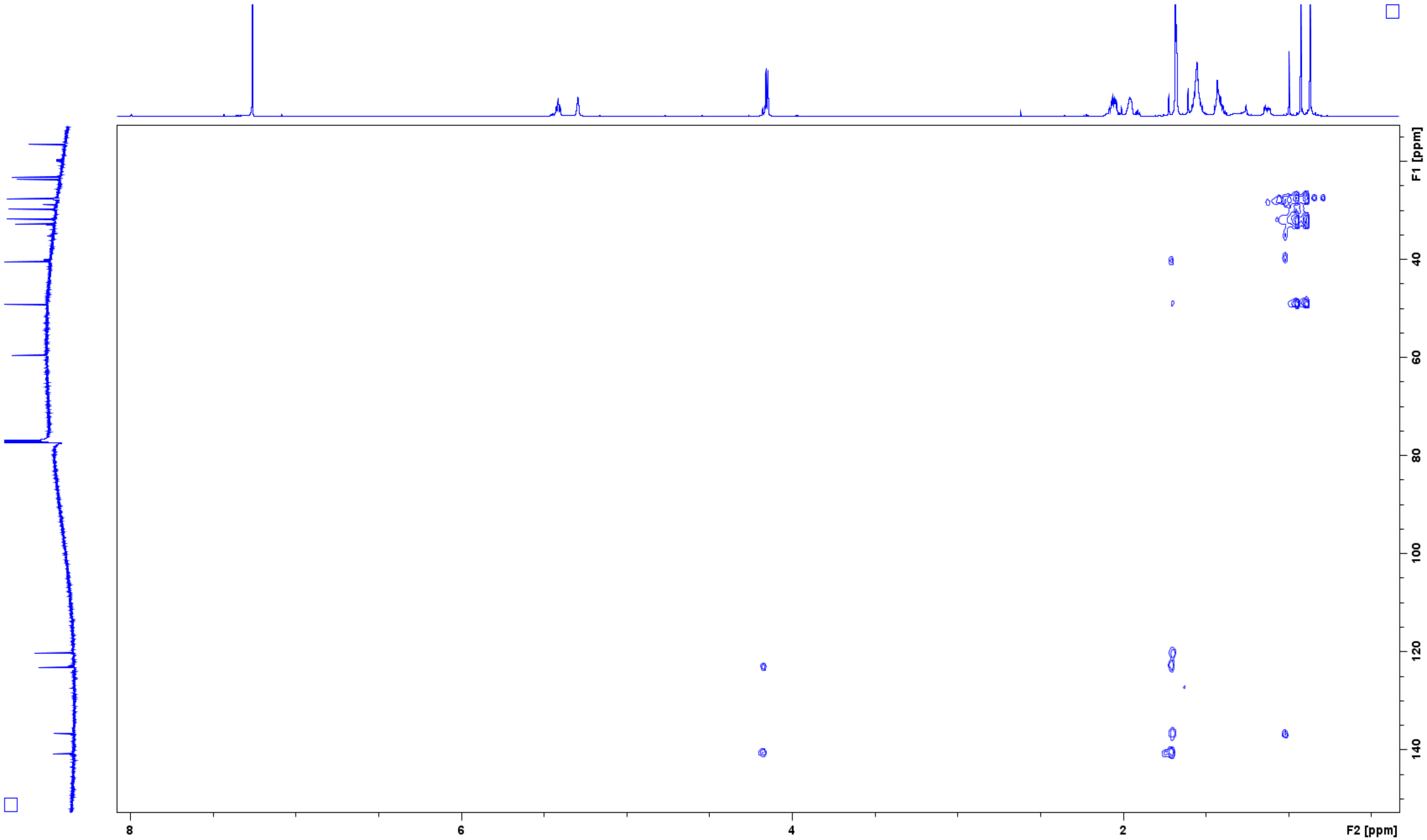

Figure NMR-S84. HMBC spectrum of compound **23** ($CDCl_3$, 300 K, 600 MHz).

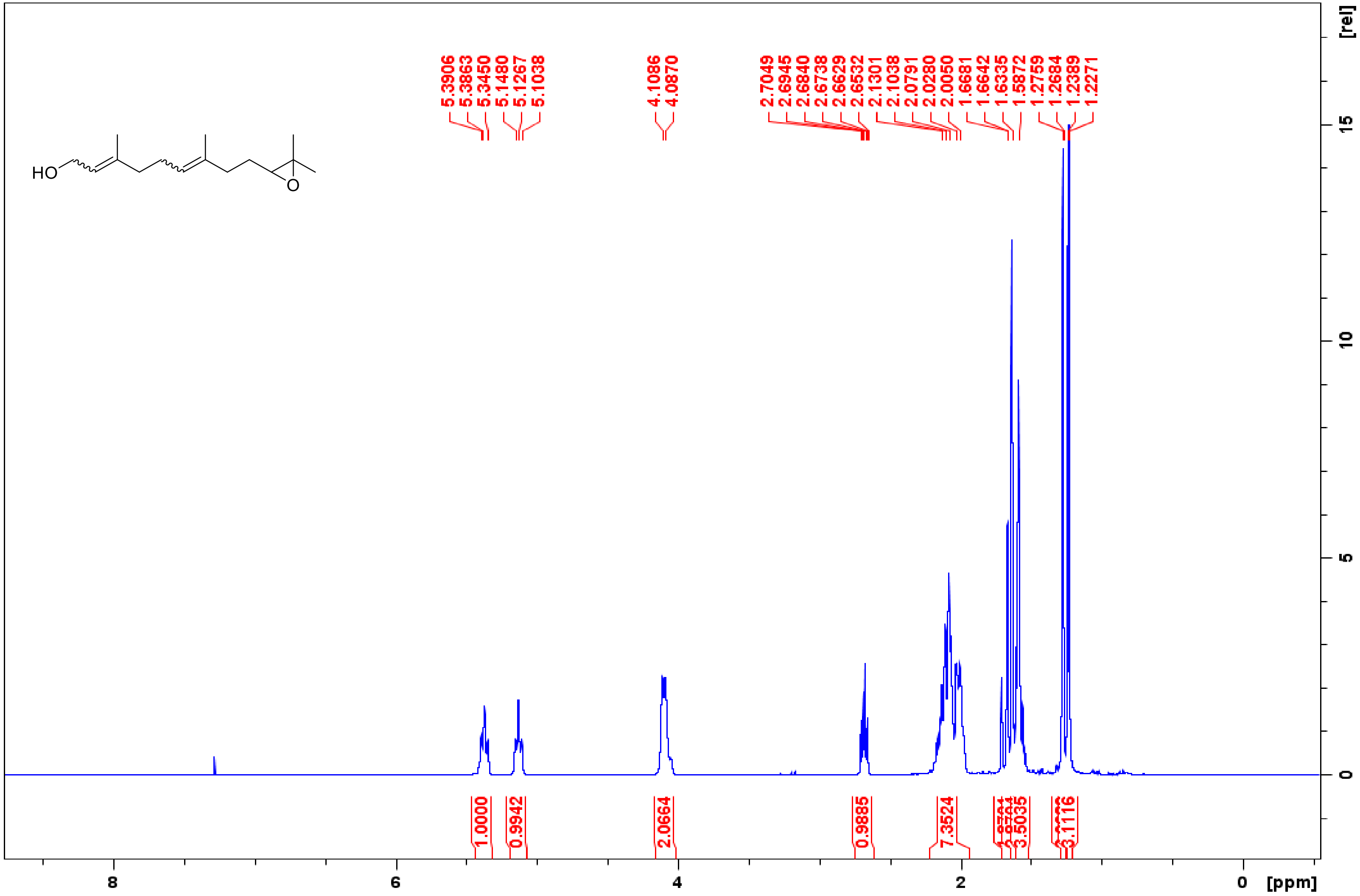

Figure NMR-S85. ^{1}H NMR spectrum of **25** ($CDCl_3$, 300 MHz).

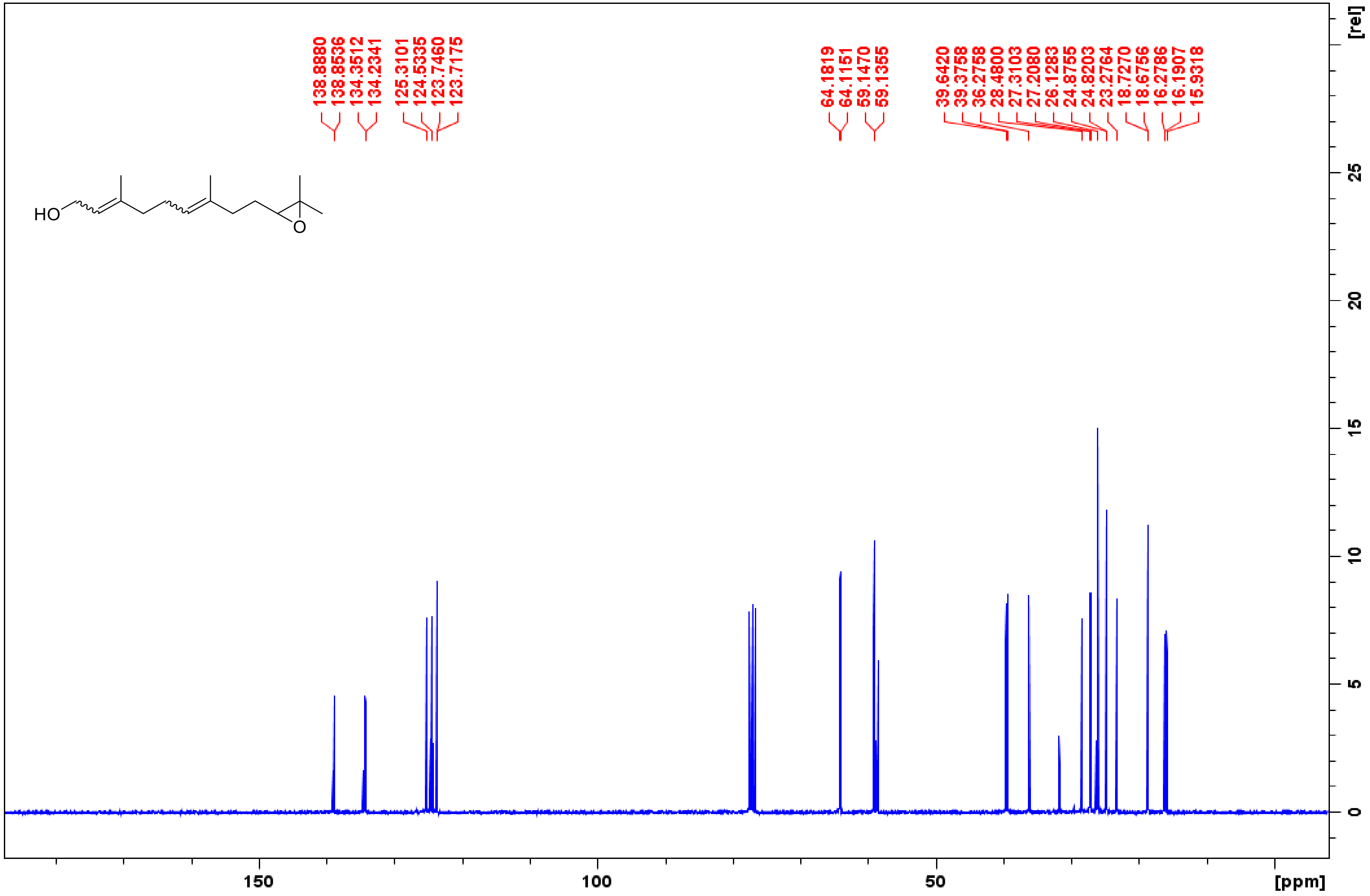

Figure NMR-S86. ^{13}C NMR spectrum of **25** ($CDCl_3$, 75 MHz).

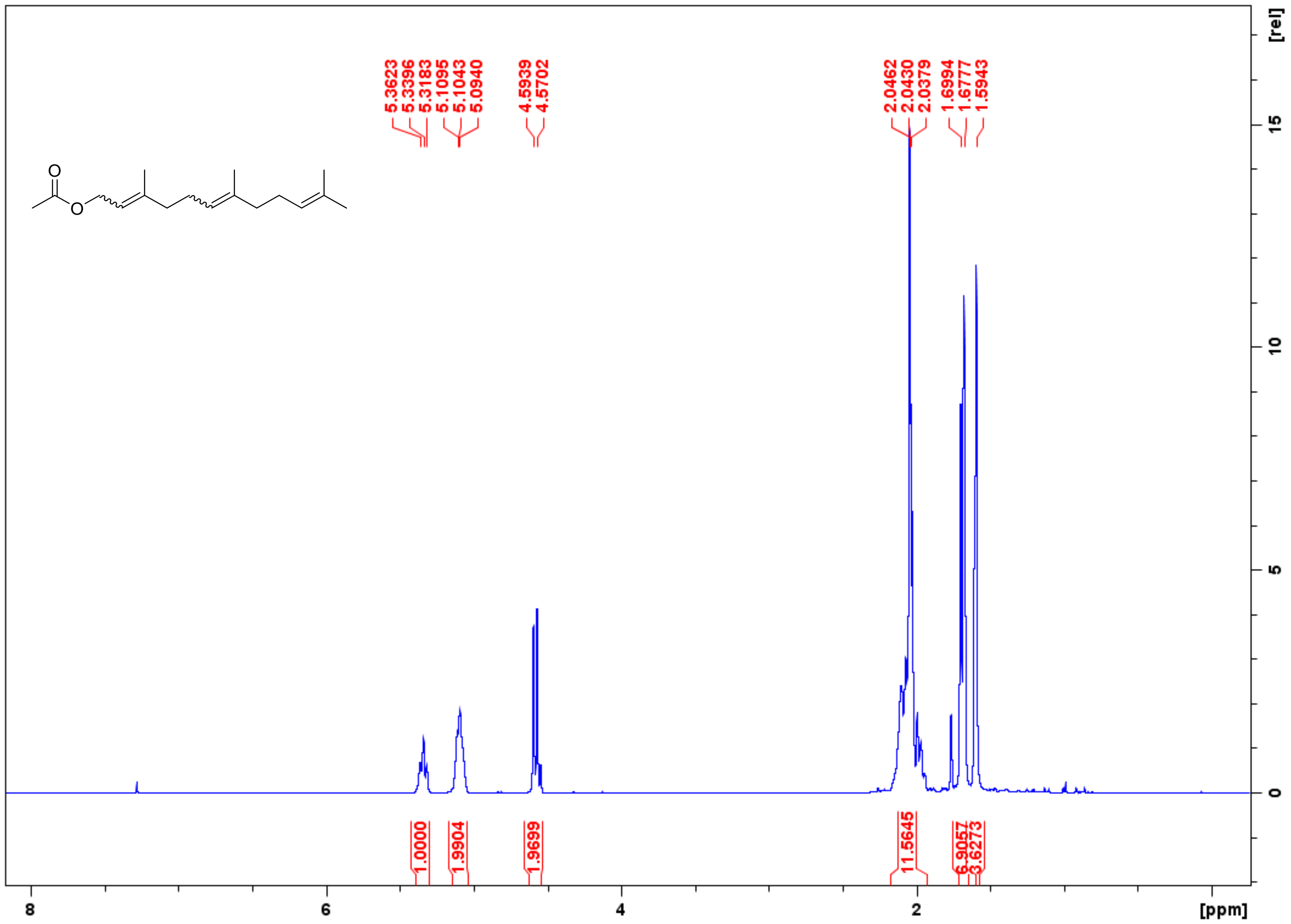

Figure NMR-S87. ^{1}H NMR spectrum of **27** ($CDCl_3$, 300 MHz).

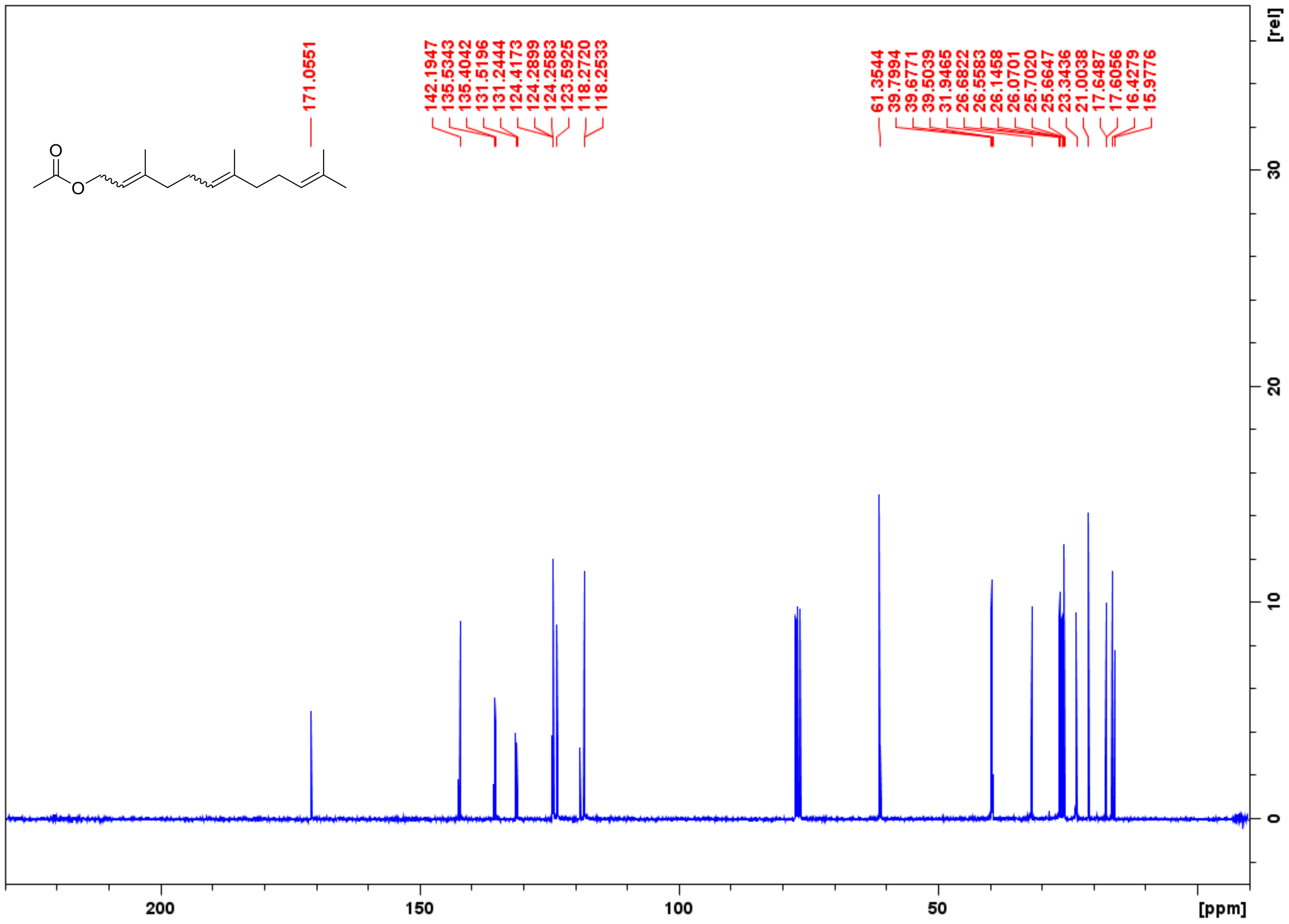

Figure NMR-S88. ^{13}C NMR spectrum of **27** ($CDCl_3$, 75 MHz).

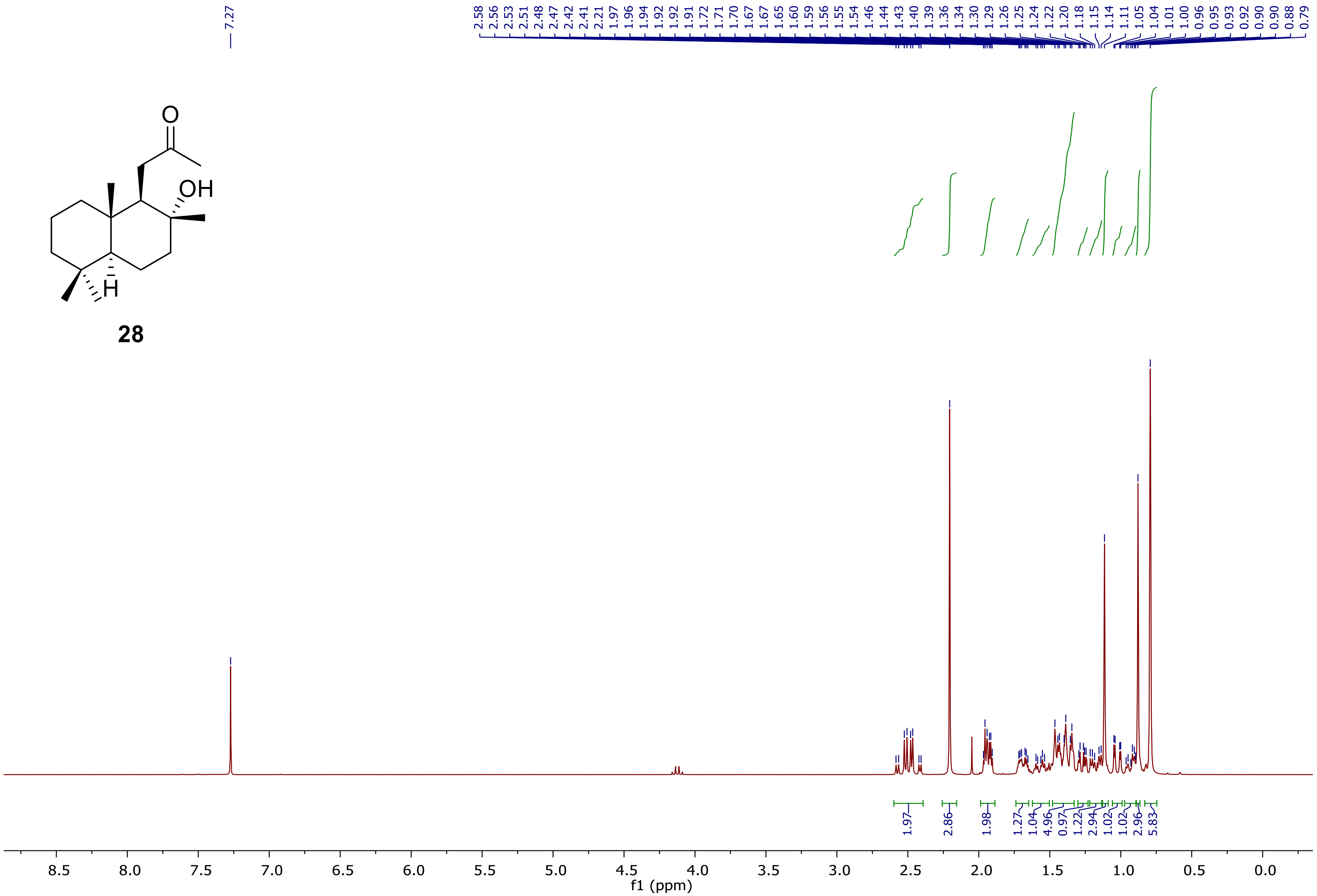

Figure NMR-S89. ^{1}H NMR spectrum of compound 1-((1*R*,2*R*,8a*S*)-2-hydroxy-2,5,5,8a-tetramethyldecahydronaphthalen-1-yl)propan-2-one (**28**).

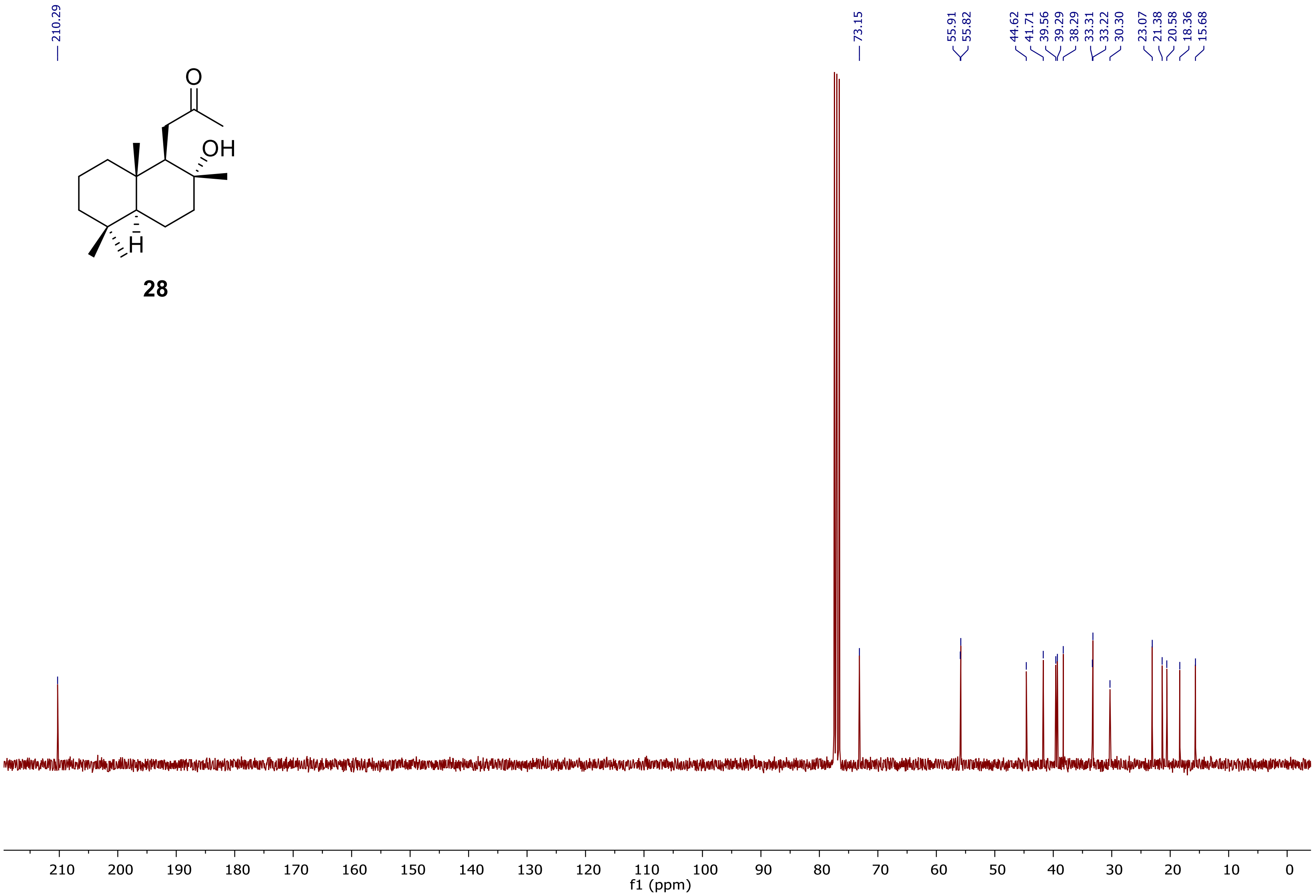

Figure NMR-S90. ^{13}C NMR spectrum of compound **28**.

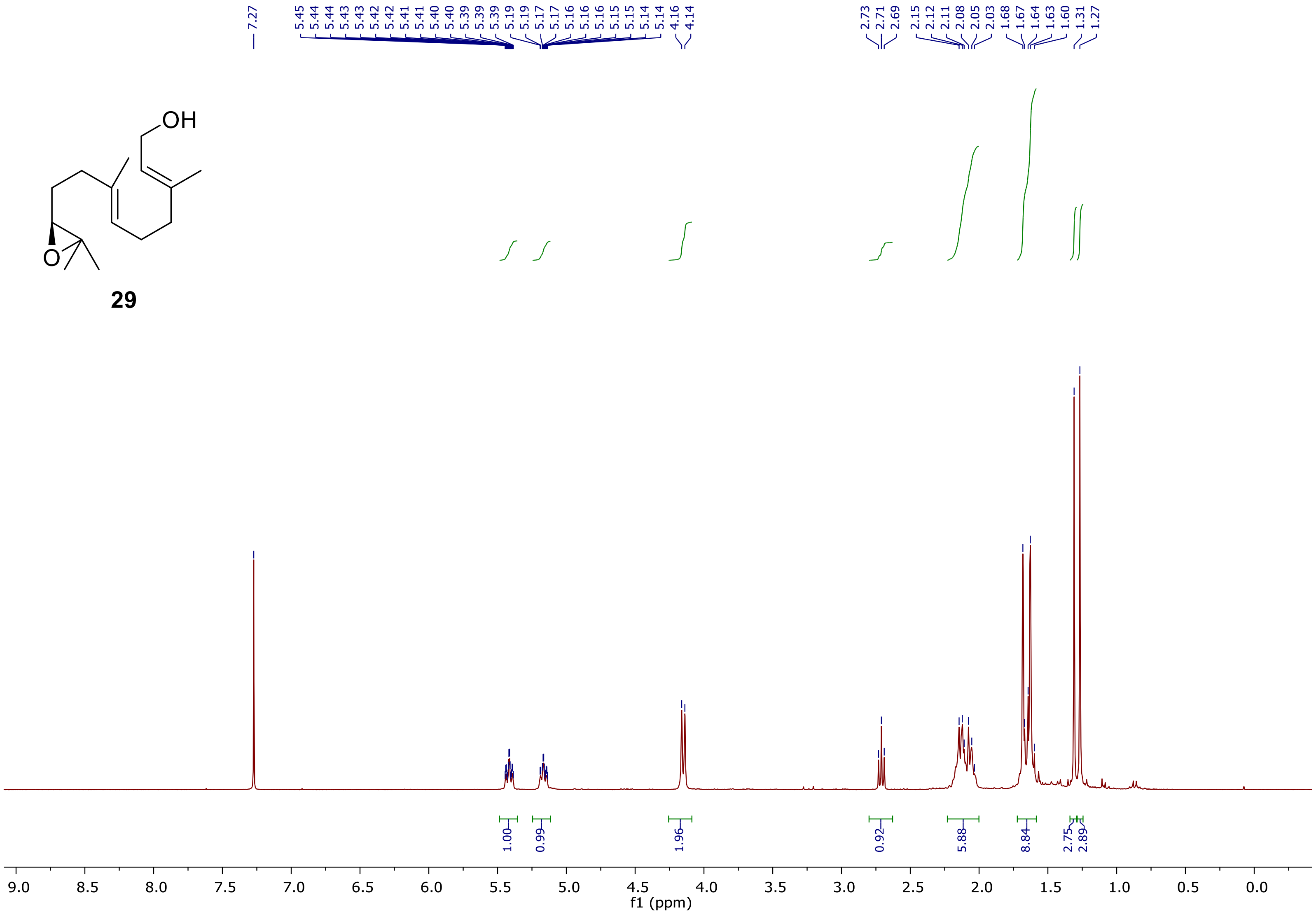

Figure NMR-S91. ^{1}H NMR spectrum of compound (2*E*,6*E*)-9-((*S*)-3,3-dimethyloxiran-2-yl)-3,7-dimethylnona-2,6-dien-1-ol (**29**).

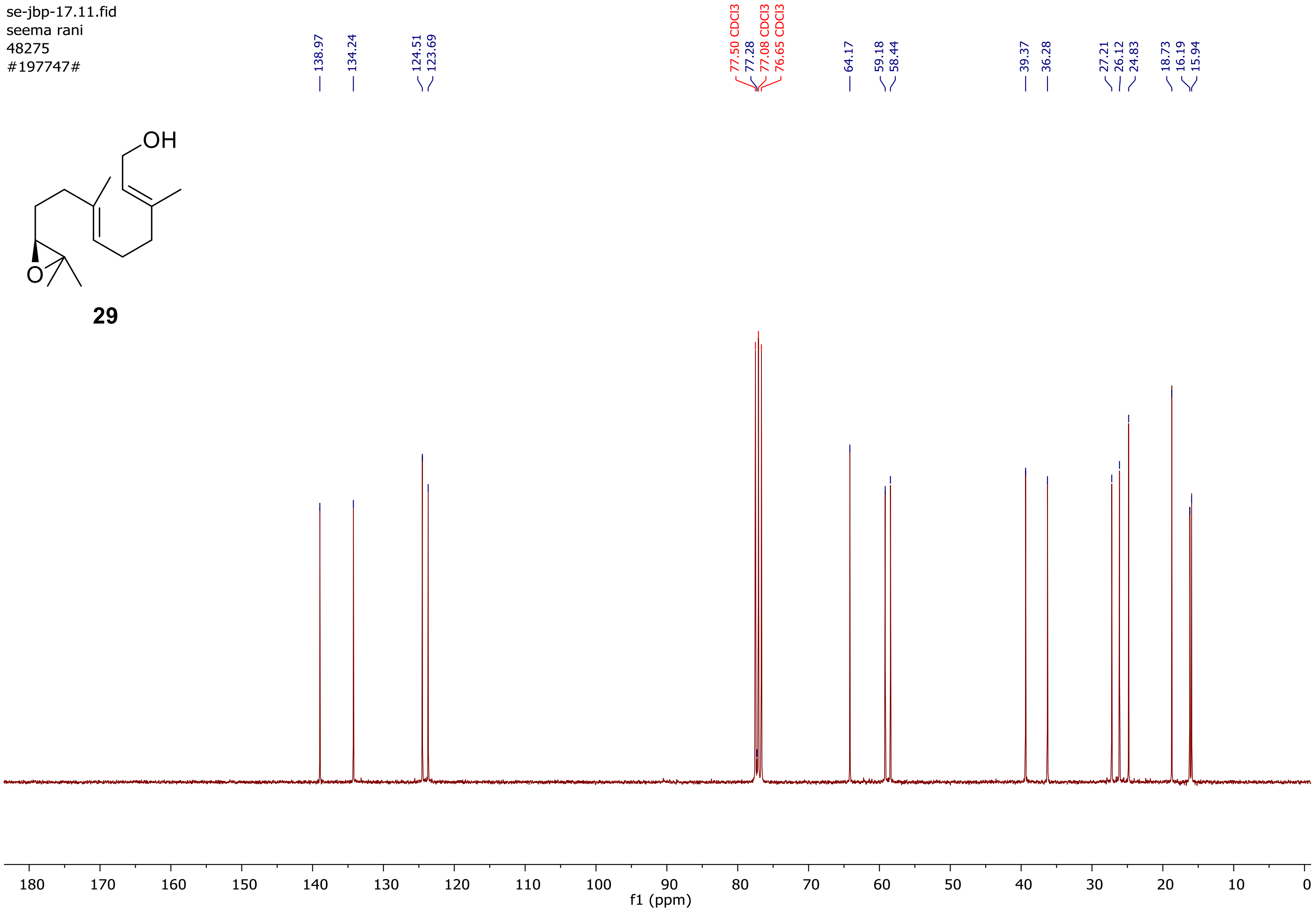

Figure NMR-S92. ^{13}C NMR spectrum of compound **29**.
